# Artificial Chiral Trinuclear Zn Catalysts: Design, Self-Assembly and Unprecedented Efficiency in Asymmetric Hydroboration of Ketones

**DOI:** 10.1021/acscentsci.5c01067

**Published:** 2025-08-12

**Authors:** Jingxi He, Shuxin Jiang, Yu Qiu, Yingchao Liu, Kuiling Ding, Xiaoming Wang

**Affiliations:** † State Key Laboratory of Organometallic Chemistry and Shanghai Hongkong Joint Laboratory in Chemical Synthesis, Shanghai Institute of Organic Chemistry, University of Chinese Academy of Sciences, Chinese Academy of Sciences, 345 Lingling Road, Shanghai 200032, China; ‡ Frontier Science Center for Transformative Molecules, School of Chemistry and Chemical Engineering, 12474 Shanghai Jiao Tong University, 800 Dongchuan Road, Shanghai 200240, China; § School of Chemistry and Materials Science, Hangzhou Institute for Advanced Study, University of Chinese Academy of Sciences, 1 Sub-lane Xiangshan, Hangzhou 310024, China; ∥ School of Chemistry and Chemical Engineering, Henan Normal University, Xinxiang 453007, China

## Abstract

The development of artificial catalysts with efficiency that can rival those of Nature’s enzymes represents one of the foremost yet challenging goals in homogeneous metal catalysis. Inspired by the exceptional performance of metalloenzymes, the design and development of highly efficient bi/multinuclear catalysts via judicious ligand design, by taking advantage of the cooperative action of the proximal catalytic sites, has attracted great attention. Herein, we report the self-assembly of a chiral hexadentate BINOL-dipyox ligand with zinc acetate into a well-defined trinuclear zinc complex, which demonstrated ultrahigh catalytic productivity in the enantioselective hydroboration of ketones with an unprecedented turnover number (TON) of 19,400 at an extremely low catalyst loading (0.005 mol %). Mechanistic investigations reveal that a cooperative Lewis acid activation mode is operating in the catalytic process, hence, underscoring the unique advantages of the trinuclear architecture.

## Graphical abstract

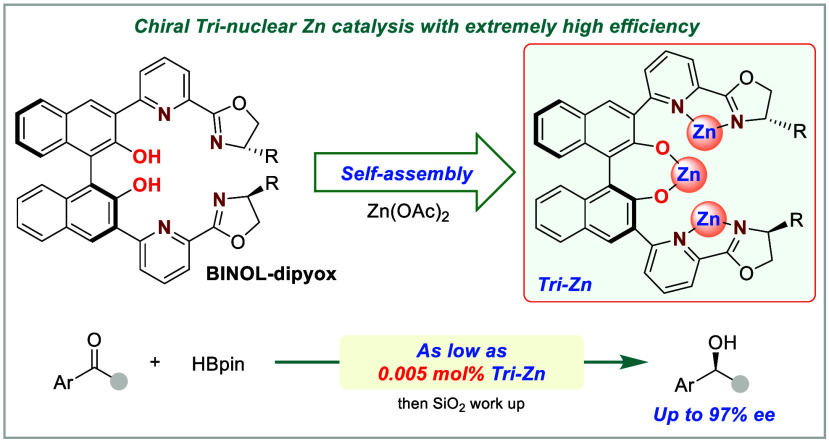

## I Introduction

The development of catalysts with extremely high efficiency is of paramount importance in catalytic synthetic chemistry, and numerous paradigms for efficient catalysts can be found in enzymes,^1–8^ with spatially organized multifunctional active sites for exceptional selectivity and efficiency in biological transformations. In this context, there are increasing studies on the development of bi/multinuclear metallic catalysts that mimics the metalloenzymes’ activation sites—where cooperative interactions between adjacent metal centers, leading to catalysts with significantly improved efficiency and selectivity as compared to their mononuclear analogues.^9–21^ For instance, zinc was known to play critical roles in biological processes involving metalloenzymes^22^ such as phosphotriesterase^23–25^ and P1 nuclease,^26–28^ containing two or three zinc ions at their active sites (Figure 1a), respectively. These have inspired the development of some of the most successful bi/multinuclear zinc catalysts to date,^29–46^ such as Trost’s dinuclear zinc-ProPhenol catalysts^47–51^ and Shibasaki’s zinc/Linked-BINOL catalysts.^52–56^ Despite these significant advancements, however, the development of highly active chiral zinc catalysts that are capable of simultaneously achieving robust catalytic efficiency and precise enantioselective control remains a critical challenge, especially for chiral tri-Zn catalysts. A key to addressing this challenge is the strategic design of multidentate ligands, which can keep the transition metal ions spatially well-organized within catalytic frameworks.

**Figure 1:**
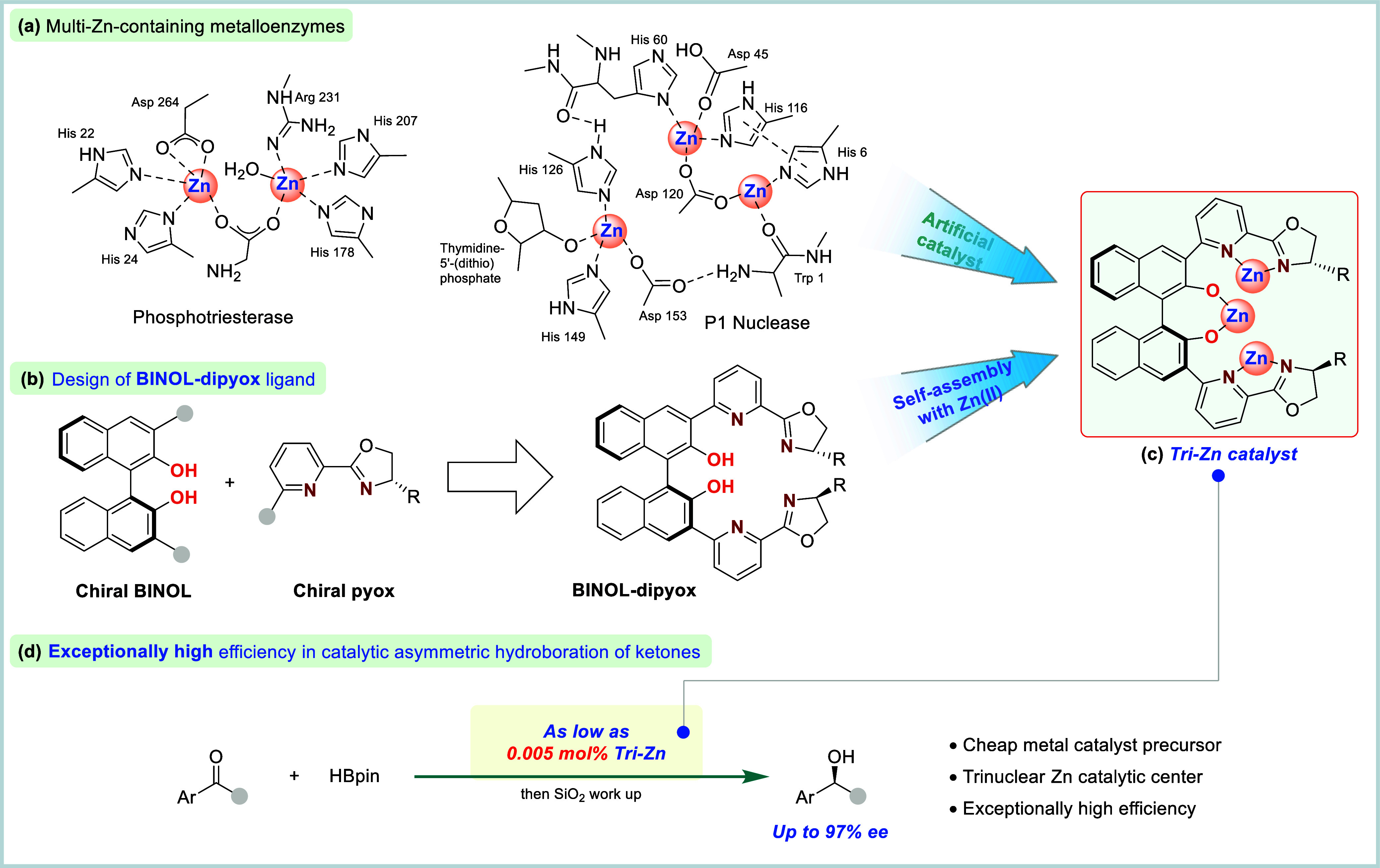
Zinc-containing metalloenzymes and artificial chiral trinuclear Zn catalysts. (a) Multi-Zn-containing metalloenzymes. (b) Design of the BINOL-dipyox ligand. (c) Tri-Zn catalyst. (d) Exceptionally high efficiency in catalytic hydroboration of ketones.

Among the numerous chiral ligands developed so far, BINOL stands out for asymmetric catalysis due to its cost-effectiveness, structural tunability, and stereochemical rigidity. Particularly, the ligand is modifiable at the 3,3′-positions with donor or steric groups, rendering it a highly attractive feature for diversification.^57–72^ It is also worthy to note that chiral pyridine-oxazolines (pyox) have proven to be a class of highly effective ligands for transition metal catalyzed asymmetric reactions.^73,74^ We envisioned that by integrating BINOL with the pyox modules into a single hybrid ligand framework, the resulting chiral N, N, O, O, N, N-hexadentate ligands may be capable of forming di/multinuclear catalytic systems to address synthetic challenges in high efficiency (Figure 1b).

Transition-metal-catalyzed asymmetric hydroboration of ketones is a pivotal strategy for constructing chiral alcohols.^75–80^ Nevertheless, studies on the Zn-catalyzed enantioselective variants of this reaction remain rare,^81–84^ despite zinc catalysts’ unique advantages of low cost, natural abundance, and reduced toxicity.^85–89^ Herein, we report the rational design and synthesis of a novel chiral BINOL-dipyox ligand, which assembles with the zinc salt to form a well-defined trinuclear zinc complex (Figure 1c). This tri-Zn system demonstrated an unprecedented efficiency (as low as 0.005 mol %) in the asymmetric hydroboration of ketones, providing access to various chiral alcohols with high enantioselectivities (Figure 1d). Mechanistic studies suggested that the extraordinary activity—far exceeding conventional mononuclear Zn systems—might originate from synergistic interactions of two reactants within the trinuclear core. This work not only establishes a new benchmark for low-loading zinc catalysis but also provides a blueprint for engineering multinuclear architectures to amplify cooperative effects in asymmetric transformations.

## II Results and Discussion

### 1 Ligand Synthesis and Self-Assembly of the Tri-Zn Complexes

As illustrated in Figure 2a, the chiral BINOL-dipyox ligand **L1** was synthesized concisely through a four-step sequence. The preparation commenced with commercially available (*S*)-2,2′-bis­(methoxymethoxy)-1,1′-binaphthalene (**S**_**1**_), which was converted into diboronate derivative **S**_**2**_ in 87% isolated yield by treatment with ^*n*^BuLi and ^*i*^PrO-BPin. Concurrently, 6-bromo-2-pyridinecarboxylic acid **S**_**3**_ was condensed with chiral aminoalcohol **S**_**4**_ under amide coupling conditions to furnish 6-bromo-2-pyridinecarboxamide **S**_**5**_ in 76% yield. Subsequently, a palladium-catalyzed Suzuki–Miyaura cross-coupling between **S**_**2**_ and **S**_**5**_ delivered the bis-pyridyl intermediate **S**_**6**_ bearing the key BINOL-dipy framework in 93% yield. Selective removal of the methoxymethyl (MOM) groups on **S**_**6**_ followed by treatment of the deprotected intermediate with DAST (diethylaminosulfur trifluoride) effectively promoted the oxazoline ring formation, ultimately affording the target BINOL-dipyox ligand **L1** in 60% overall yield for the two steps. A series of BINOL-dipyox ligands **L2**-**L7** were also successfully synthesized by following a similar procedure.

**Figure 2:**
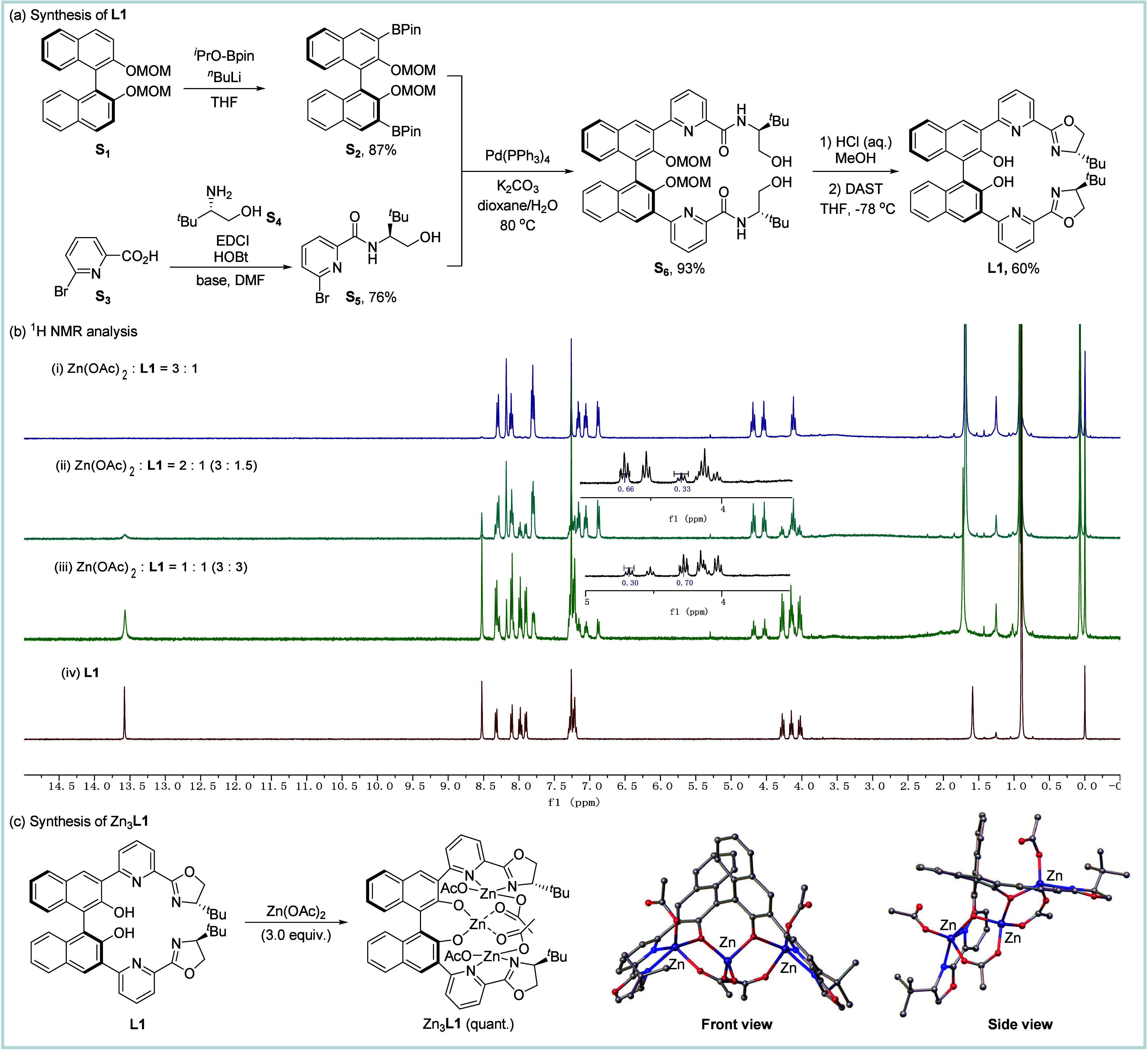
Synthesis of the ligand and self-assembly of the tri-Zn complex. (a) Synthesis of **L1**. (b) ^1^H NMR analysis. (c) Synthesis of Zn_3_ **L1**.

With these multidentate ligands in hand, their coordination propensity was evaluated by treating **L1** with varying amounts of zinc acetate (1 to 3 equiv) under ambient conditions to probe into the preferential formation of mono-, bi-, or trinuclear complexes. ^1^H NMR analysis of the reaction mixtures revealed signals with a pronounced downfield shift for the oxazoline protons (Figure 2b), which could be attributed to zinc coordination. Intriguingly, irrespective of the molar ratios of Zn-to-ligand (1:1, 2:1, or 3:1) in the system, the dominant ligated species formed in the mixtures exhibited identical spectral signatures at 4.12, 4.53, and 4.68 ppm, suggesting that the ligand is prone to adopt such a specific coordination architecture with the Zn­(II) ions. The ^1^H NMR spectrum for the 3:1 Zn-to-ligand mixture revealed a set of signals with chemical shifts distinct from those of **L1**, suggesting an exclusive formation of Zn_3_ **L1**-type species in this system. In contrast, the ^1^H NMR spectra for both the 2:1 and 1:1 Zn-to-ligand mixtures showed the same signals as the 3:1 mixture, along with the signals that are characteristic of the free ligand. These observations indicated that hexadentate **L1** has a strong propensity to undergo selective self-assembly with Zn­(II) ions, leading to the preferential formation of a trinuclear complex (Zn_3_ **L1**). The metal-to-ligand stoichiometry appears to have little effect under these conditions, as no alternative complexes (e.g., Zn_1_ **L1** or Zn_2_ **L1**) were detected. Single-crystal X-ray diffraction analysis of the trinuclear complex (Figure 2c) unambiguously established its Zn_3_ **L1** moiety in the structure. The complex features three Zn­(II) centers coordinated with distinct donor atoms in **L1** and bridged by two acetate ions. While the outer two Zn ions are bound by two pyridine-oxazoline moieties, respectively, the central Zn is coordinated by the two BINOL-derived oxygen atoms. Notably, the axial chirality of the BINOL scaffold induces a pronounced helical distortion (torsion angle = −100.7°), creating a sterically accommodating cavity that stabilizes the trimetallic core. Two acetate ligands adopt μ_2_-bridging modes, completing the tetrahedral geometry at each Zn­(II) center.

### 2 Reaction Development and Unprecedented Efficiency

4-Acetylbiphenyl was initially selected as the standard substrate for Zn-catalyzed asymmetric hydroboration using pinacolborane (HBpin) as the hydroboration reagent (Table 1). The reaction proceeded smoothly in THF at room temperature using (*S*, *S*, *S*)-**L1** (2.0 mol %)/Zn­(OAc)_2_ (6.0 mol %) as the chiral catalyst, giving the desired alcohol **2a** in 99% yield with 90% enantiomeric excess (*ee*), thus demonstrating the feasibility of using trinuclear Zn for the catalytic asymmetric hydroboration of ketones (Entry 1). Notably, when using (*R*, *S*, *S*)-**L1** in the reaction, wherein the chirality of the oxazoline moiety was maintained while inverting the chirality of the BINOL scaffold, the *enantiomer of* **2a** was obtained with a slightly decreased *ee* value of 80% (Entry 2), suggesting that the chirality of BINOL backbone might play a key role in dictating the facial attack of the hydride species on the ligated carbonyl group. Using (*R*, *R*, *R*)-**L1** also afforded the enantiomer isomer in 99% yield with 90% *ee* (Entry 3). When the ^*t*^Bu group on the oxazoline moiety was replaced by Bn ((*S*, *S*, *S*)-**L2**), the catalyst exhibited slightly lower activity, and the reaction gave the desired product in 92% yield with 89% *ee* (Entry 4). Altering the chirality of the oxazoline moiety to *R* (using (*S*, *R*, *R*)-**L2**) led to a further minor decrease in both the product yield and *ee* value (Entry 5), though the absolute configuration of the product remained unchanged, further attesting that the chiral induction of the catalysis is likely to be dominated by the absolute configuration of the BINOL moiety. These results (entries 1–5) indicate that the chirality of the BINOL unit in the ligand is critical as it determines the configuration of the product, while the chirality of the oxazoline moiety plays a secondary role in modulating the *ee* value. These results also indicate that the ligand with a *S*-configured BINOL skeleton and a *S*-configured oxazoline moiety is chirally matched for the current reaction. Therefore, (*S*, *S*, *S*)-ligands (**L3**-**L6**) with different substituents on the oxazoline moiety were further investigated, and the ligands with naphthalenemethyl, ethyl, and isopropyl afforded the same results as that of ^*t*^Bu (entries 1, 6–8). The reaction using (*S*, *S*, *S*)-**L6** with a phenyl group on the oxazoline led to a slightly lower yield (88%) and *ee* (88%) (Entry 9). Reducing the reaction temperature to 10 °C increased the ee to 92% using **L1** (Entry 10). To elucidate the catalytic role of the chiral hexadentate ligand, systematic ligand modification studies were conducted. Primary modification involved methyl-protection of the phenolic hydroxyl group in the BINOL-dipyox ligand. This protection resulted in almost complete loss of stereochemical control, yielding the racemic product with significantly declined efficiency (entry 11, 34% yield). Furthermore, the reaction using monofunctionalized BINOL-pyox **L8** demonstrated poor catalytic performance, achieving only 16% yield with 16% *ee* (Entry 12). These results prompted us to hypothesize that the zinc ion coordinated to the phenolic hydroxyl group might play a crucial role in activating ketone carbonyl for the stereodiscriminated attack by the boronate hydride. Notably, control experiments employing BINOL, pyridine-oxazoline, or their ligand mixtures (entries 13–15) only demonstrated poor catalytic performance (<6% *ee*), which are in starking contrast with the superior efficiency of the BINOL-dipyox (90% *ee*). Such a dramatic difference further consolidates the advantages of the rationally designed chiral polydentate ligands in achieving enantioselective hydroboration reactions of ketones. Interestingly, using the *pre*-prepared tri-Zn complex Zn_3_ **L1** directly as the catalyst (2.0 mol %) achieved full conversion and gave the desired product **2a** with a slightly lower *ee* (84%). However, when KOAc (1.0 or 10 mol %) was added to the tri-Zn complex catalyzed reaction, the *ee* was improved to match that of the *in situ*-formed tri-Zn system (90%), suggesting that the OAc^–^ anion generated during the self-assembly of Zn­(OAc)_2_ and **L1** may play a role in chiral control.

**Table 1:**
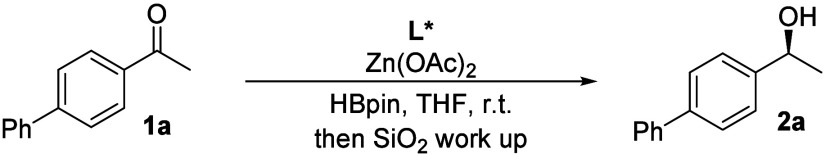
Screening of the Ligands^b^

**Entry**	L*	**Conv. of 1a (%)** ^c^	**Yield of 2a (%)** ^d^	***ee*** **(%)** ^e^
1	(*S*, *S*, *S*)-**L1**	>99	99	90 (*S*)
2	(*R*, *S*, *S*)-**L1**	>99	99	–80 (*R*)
3	(*R*, *R*, *R*)-**L1**	>99	99	–90 (*R*)
4	(*S*, *S*, *S*)-**L2**	93	92	89 (*S*)
5	(*S*, *R*, *R*)-**L2**	90	89	88 (*S*)
6	(*S*, *S*, *S*)-**L3**	>99	99	90 (*S*)
7	(*S*, *S*, *S*)-**L4**	>99	99	90 (*S*)
8	(*S*, *S*, *S*)-**L5**	>99	99	90 (*S*)
9	(*S*, *S*, *S*)-**L6**	89	88	88 (*S*)
10^f^	(*S*, *S*, *S*)-**L1**	99	99	92 (*S*)
11[Table-fn t1fn7]	(*S*, *S*, *S*)-**L7**	45	34	*rac*.
12[Table-fn t1fn7]	**L8**	24	16	16 (*S*)
13[Table-fn t1fn7]	**L9**	93	90	*rac*.
14[Table-fn t1fn7]	**L10**	17	15	6 (*S*)
15[Table-fn t1fn7]	**L9 + L10**	22	17	4 (*S*)

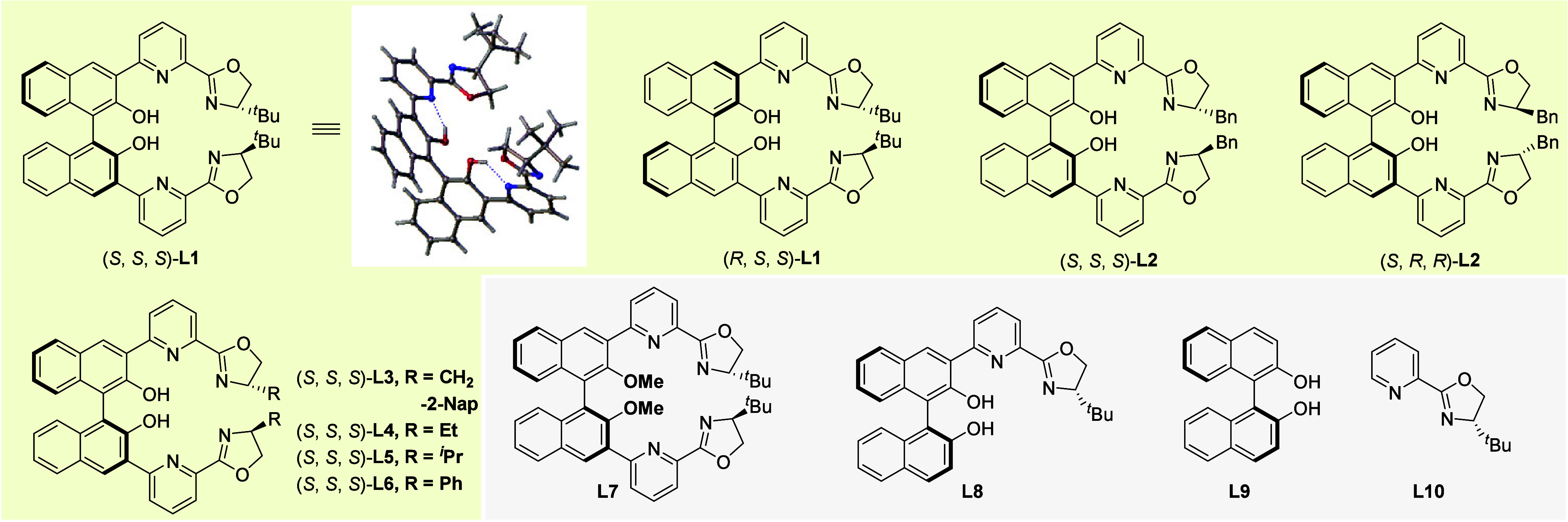

a Reaction conditions: **1a** (0.1 mmol), HBpin (2.0 equiv), L* (2 mol %), Zn­(OAc)_2_ (6 mol %), THF, r.t., 4 h.

b The conversion of **1a** was determined by GC using *n*-decane as the internal standard.

c Isolated yield.

d The *ee* values of **2a** were determined by chiral HPLC

e 10 °C, 24 h.

f L* (6 mol %)

During the optimization of the reaction conditions, it was found that the catalyst was highly active, and the reactant **1a** could be fully consumed in a short time (4 h). Therefore, the reactions of **1a** and HBpin were carried out under gradually reduced catalyst loadings (Table 2). A survey of **L1** with loadings ranging from 2 to 0.005 mol % revealed that the reaction still proceeds smoothly at an extremely low catalyst loading (0.005 mol % Zn_3_/**L1)**, affording the product in 97% yield with 90% *ee* (Entry 6). Further reducing the catalyst loading to 0.002 mol %, the reaction product was still obtained in excellent yield (93%), albeit with a slightly lower *ee* (80%, Entry 7). However, without a chiral ligand, 0.015 mol % Zn­(OAc)_2_ exhibited some catalytic activity in the reaction, albeit with low efficiency, indicating a ligand-accelerated catalysis in this system (entry 9). These results demonstrated the robust catalytic performance of the BINOL-dipyox-Zn_3_ catalyst in the reaction, particularly at very low catalyst loadings. Notably, kinetic studies showed that the reaction rate decreased concomitantly with a reduction in the Zn­(OAc)_2_ (0.1*x* mol %) to **L1** (0.1 mol %) ratio (*x* = 3, 2, 1), suggesting that a 3:1 ratio of Zn­(OAc)_2_-to-**L1** is crucial for achieving an efficient and highly enantioselective asymmetric hydroboration of ketones (for details, see the ).

**Table 2:**
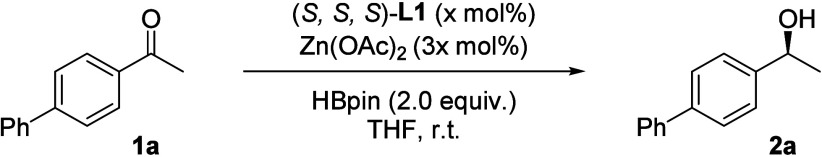
Optimizing the Reaction Conditions^a^

**Entry**	**L1** **(mol %)**	**Time (h)**	**Conv. (%)** ^b^	***ee*** **(%)** ^c^	**TON**
1	2	4	>99	90	-
2	1	4	>99	90	-
3	0.1	6	>99	90	-
4^d^	0.1	24	99 (98)^e^	92	-
5	0.01	12	>99	90	-
6	0.005	24	99 (97)^e^	90	19,400
7	0.002	48	99 (93)^e^	80	46,500
8	0.001	48	50	79	-
9^f^	-	24	22	*rac*.	

a Reaction conditions: **1a** (0.1–200 mmol), (*S, S, S*)-**L1** (0.002 mmol), Zn­(OAc)_2_ (0.006 mmol), THF (1–270 mL), r.t.

b The conversion of **1a** was determined by GC using *n*-decane as the internal standard.

c The *ee* values of **2a** were determined by chiral HPLC.

d 0 °C.

e Isolated yield in parentheses.

f Zn­(OAc)_2_ (0.015 mol %), without a chiral ligand.

To further extend the generality of the BINOL-dipyox-Zn_3_ catalyst in asymmetric hydroboration, we investigated the scope of ketones using 0.1 mol % Zn_3_/**L1** as the catalyst (Figure 3). Remarkably, this catalytic system demonstrates exceptional generality across a diverse range of ketones. Both electron-donating (-Ph, -Me, -OMe, -OEt) and electron-withdrawing substituents (-F, -Cl, -Br, -CF_3_, -NO_2_) on the ketones were tolerated, delivering the corresponding chiral alcohol products **2a**-**2s** with high efficiency (80–99% yields) and good to excellent stereocontrol (80–97% *ee*). The (hetero)­aromatic ketones also worked well in the protocol, and the reactions of naphthyl derivatives (**2t**, **2u**: 96–99% yields, 85% ee and 80% *ee*), thiophene-containing substrates (**2v**, **2w**: 92–93% yields, 84–92% *ee*), and 1,3-benzodioxole analogues (**2x**; 99% yield, 91% *ee*) proceeded smoothly. It is noteworthy that the reactions of sterically demanding fused aromatic rings afforded their corresponding products [i.e., with five- (**2y**-**2z**), six- (**2aa**-**2ad**), and seven-membered (**2ae**) systems] in excellent yields (90–99%) with high enantioselectivities (86–97% *ee*). Furthermore, diverse heterocyclic substrates such as 5,6,7,8-tetrahydroquinolin-8-one, thiochroman-4-one, and 4-chromanone proved to be amenable to the procedure, affording the corresponding chiral alcohols **2af**-**2ah** with remarkable efficiency (83–97% yields) and good stereochemical control (82–96% *ee*). In addition, 4-chlorobutyrophenones performed quite well in the reaction, providing the corresponding chlorohydrin products **2ai** and **2aj** in excellent yields (both 96%) and *ee* values (96% and 88%), respectively.

**Figure 3:**
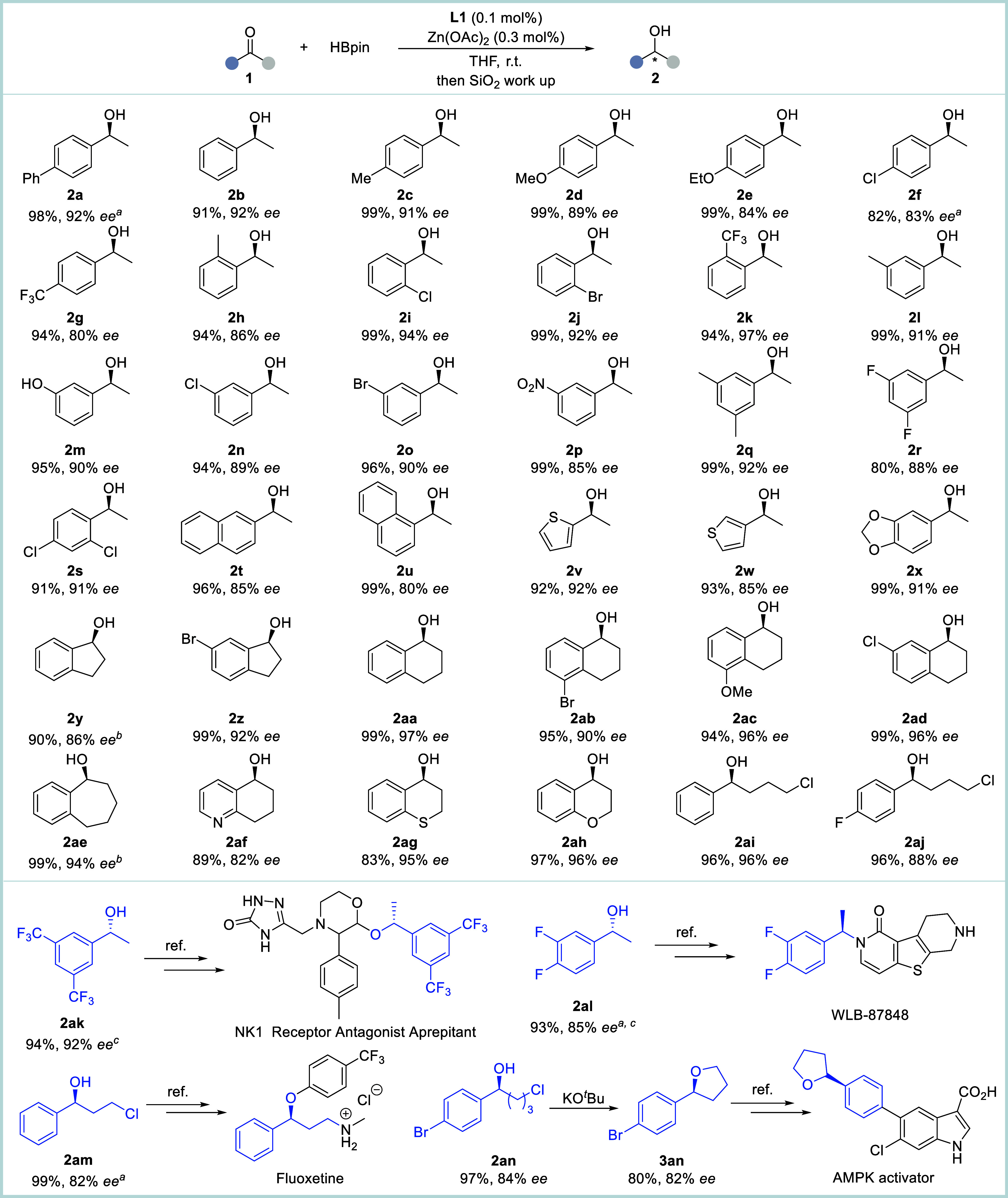
Substrate scope. Reaction conditions: **1** (2 mmol), (*S, S, S*)-**L1** (0.1 mol %), Zn­(OAc)_2_ (0.3 mol %), rt, 12 h. ^*a*^ 0 °C, 24 h. ^*b*^ **1** (0.1 mmol), (*S, S, S*)-**L1** (2 mol %), Zn­(OAc)_2_ (6 mol %), r.t., 4 h. ^*c*^ (*R, R, R*)-**L1** (0.1 mol %) was used. Isolated yield.

Notably, the chiral alcohols **2ak**-**2am** represent key synthetic intermediates for several clinically important agents, including the NK1 receptor antagonist aprepitant,^90^ σ1 receptor antagonist WLB-87848 ^91^ and the antidepressants fluoxetine,^92^ respectively, were also obtained in high yields with good to excellent enantioselectivities using the protocol (93–99% yields, 82–92% *ee*). Furthermore, chiral alcohol **2an** can be synthesized in 97% yield with 84% *ee*. This intermediate underwent cyclization to generate chiral tetrahydrofuran **3an** smoothly, which serves as a strategic precursor for accessing AMP-activated protein kinase (AMPK) activators.^93^

### 3 Mechanistic Studies

Two distinct mechanistic pathways have been proposed for zinc-catalyzed hydroboration of ketones, i.e., (i) Lewis acid activation of the ketone by carbonyl coordination to the Zn center,^84,94,95^ and (ii) *in situ* formation of zinc-hydride species as the catalytically active intermediates.^96–100^ To elucidate the operating mechanism of the current catalytic system, we conducted a series of mechanistic investigations, including reaction kinetic order studies using acetophenone (**1b**), Hammett analysis and the nonlinear effects (Figure 4). The initial rate of the reaction exhibited a linear dependence on the catalyst concentration, suggesting that the trinuclear Zn core is most likely to be involved in the rate-limiting step and to remain largely intact over the entire course of catalysis (Figure 4a). Furthermore, a linear correlation between the reaction rate and HBpin concentration was also observed, indicating that a molecule of boronate hydride should also be involved in the turnover-limiting step. In contrast, variation in acetophenone **1b** concentration exhibited a negligible impact on initial rate, indicating that the ketone is unlikely to be involved in the rate-determining step. Next, Hammett analysis was carried out on *para*-substituted acetophenone **1**, to probe the electronic effect of the ketone on the rate of the reaction. This analysis discloses a positive ρ value (1.31) of the slope, showing that the reaction is more favorable in the presence of an electron-withdrawing group on the ketones (Figure 4b). Integration of the Hammett study and the observed zero-order kinetics of acetophenone suggests that acetophenone may coordinate with the catalyst, forming a pre-equilibrium before the rate-determining step. These findings may provide mechanistic evidence for HBpin mediated nucleophilic addition to a zinc-polarized carbonyl intermediate, wherein the Lewis acidic Zn center activates the ketone substrate, rendering the electrophilic carbon susceptible to borane attack. This is also consistent with first order for Zn_3_ **L1** and HBpin, but zero order for ketone **1b** in the reaction since **1b** has been coordinated with Zn_3_ **L1**.

**Figure 4:**
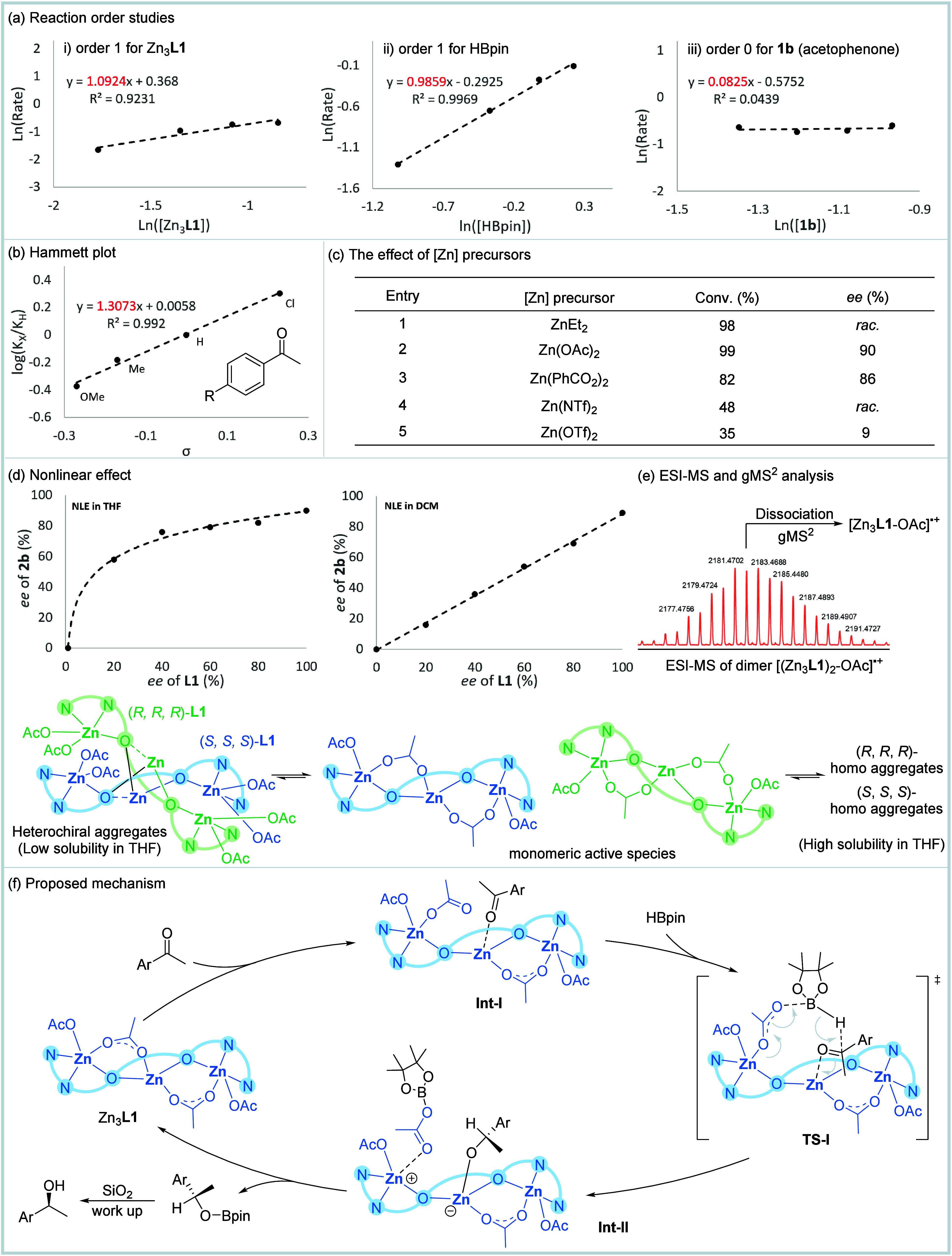
Mechanistic studies. (a) Reaction order studies. (b) Hammett plot. (c) The effect of [Zn] precursors. (d) Nonlinear effect. (e) ESI-MS and gMS^2^ analysis. (f) Proposed mechanism.

To elucidate the potential involvement of Zn–H intermediates in the current catalytic system, the Zn_3_ **L1** complex (1.0 equiv) was mixed with HBpin (3.0 equiv) in CDCl_3_. Notably, ^1^H NMR analysis revealed characteristic Zn–H vibrational signals (δ 4.6 ppm, consistent with literature reports^96,101,102^ ). Given that Et_2_Zn serves as a conventional Zn precursor for generating Zn–H species in hydroboration reactions,^103–105^ Zn­(OAc)_2_ was replaced with Et_2_Zn under the standard conditions. However, this modification yielded only racemic product in 98% yield (Figure 4c), suggesting that while trace Zn–H species might form, they likely do not contribute to the catalytic cycle. Further investigations into anionic effects using alternative Zn precursors revealed carboxylate anions (e.g., ^–^OAc, PhCO_2_^–^) to be critical for achieving high activity and *ee*. In contrast, Zn­(NTf)_2_, Zn­(OTf)_2_, and ZnBr_2_ resulted in markedly reduced conversion and *ee* values. Collectively, these results using different Zn precursors highlight the essential role of acetate anions in both catalytic efficiency and chiral induction and also indicate that Zn–H species is less likely to be active in the current reaction.

The nonlinear effect (NLE) for the Zn_3_ **L1** catalyzed hydroboration of ketones was also investigated with (*S*, *S*, *S*)-**L1** in different enantiomeric purities in THF. The relationship between the *ee* values of product **2b** and ligand **L1** showed that an obvious positive nonlinear effect existed (Figure 4d). This observation suggested that oligomeric aggregates may exist in the reaction system.^106–110^ Notably, we observed a distinct solubility difference between these diastereomeric complexes, and the heterochiral aggregate shows poor solubility in the reaction solvent (THF) compared with its homochiral counterparts. Particularly, combinations of Zn­(OAc)_2_ with (*S, S, S*)-**L1** at varying enantiopurities are completely soluble in CH_2_Cl_2_. Corresponding NLE experiments conducted in CH_2_Cl_2_ revealed a linear correlation, indicating that the observed positive nonlinear effect in THF most likely originates from the poor solubility of heterochiral aggregates in that reaction solvent. The reaction mixture (1:1) of (*S*, *S*, *S*)-Zn_3_ **L1** and (*R*, *R*, *R*)-Zn_3_ **L1** complexes was analyzed by ESI-MS (Figure 4e). The MS peak of dimer species at *m*/*z* 2183.4688 (*m*/*z* calcd for [(Zn_3_ **L1**)_2_–OAc]^+^ 2183.2686) was observed, which was subsequently analyzed by gMS^2^, and the MS peak of monomer at *m*/*z* 1061.2119 (*m*/*z* calcd for [Zn_3_ **L1**–OAc]^+^ 1061.1323) was further observed. Combined with the reaction order studies (first order on tri-Zn), the above results suggested that monomeric complex Zn_3_ **L1** most likely functions as the active and effective catalytic intermediate, and limited dimerization or oligomerization may also occur between tri-Zn species to form out-cycle species.

Based on the above results, a possible mechanism for the process is proposed in Figure 4f. The catalytic cycle commences with the coordination of the carbonyl substrate to Zn_3_ **L1**, forming intermediate **Int-I**. Subsequent bimolecular interaction with HBpin proceeds through a concerted transition state (TS-I) to form **Int-II** that exhibits a first-order kinetic dependence on both Zn_3_ **L1** and HBpin concentrations. This step involves a process where zinc-mediated polarization of the carbonyl group, significantly enhancing its electrophilicity, and simultaneous B–H bond activation in HBpin through its interaction to the acetate of Zn_3_ **L1**. The third Zn (II) might maintain the structural stability of the chiral catalyst. The reaction order studies and Hammett plot indicated that this step, the addition of hydride from HBPin to ketone, is most likely the rate-determining step. Then, dissociation of **Int-II** regenerates the active catalyst Zn_3_ **L1** while releasing the [O-Bpin] species. After the reaction, silicate workup converts the [O-Bpin] species into the corresponding chiral alcohol product. While experimental mechanistic investigations suggest the proposed mechanism as the dominant pathway, the intricate nature of the trinuclear catalytic system permits the existence of alternative routes that cannot be ruled out. It is proposed that the intrinsic origin of the unprecedented activity in the asymmetric hydroboration of ketones lies in the synergistic activation of dual substrates by the trinuclear zinc centers and the confined reaction microenvironment within the self-assembled architecture.

## III Summary and Conclusions

In summary, we have developed a class of self-assembled trinuclear chiral zinc complexes using earth-abundant Zn­(II) ions and rationally designed chiral BINOL-dipyox ligands. The chiral tri-Zn system demonstrated unparalleled catalytic efficiency in the asymmetric hydroboration of ketones, achieving a turnover number (TON) of 19,400 with a catalyst loading of 0.005 mol %, which is unprecedented by using conventional mononuclear Zn systems. This work not only establishes tri-Zn as a benchmark for sustainable, high-performance asymmetric catalysis but also underscores the transformative potential of multinuclear metal assemblies. Further detailed mechanistic investigations and applications of this tri-Zn system are underway in our laboratory.

## Supplementary Material











## Notes

Experimental procedures, complete characterization data, and copies of ^1^H, ^13^C, and ^19^F NMR spectra and HPLC chromatograms (PDF)
CheckCIF/PLATON report (PDF)
CheckCIF/PLATON report (PDF)
X-ray data (CIF)
X-ray data (CIF)
