# Supplementary material for: Artificial Chiral Trinuclear Zn Catalysts: Design, Self-Assembly and Unprecedented Efficiency in Asymmetric Hydroboration of Ketones
Source: ACS Cent Sci. 2025 Aug 12;11(9):1773–83. doi: 10.1021/acscentsci.5c01067 (PMC12464761; doi:10.1021/acscentsci.5c01067)
Supplement: Supplementary file 1 [file oc5c01067_si_001.pdf]

Supporting information

Artificial Chiral Trinuclear Zn Catalysts: Design, Self-Assembly and Unprecedented Efficiency in Asymmetric Hydroboration of Ketones

Jingxi He,[a] Shuxin Jiang,[a] Yu Qiu,[a] Yingchao Liu,[a] Kuiling Ding,*[a,b] Xiaoming Wang*[a,c,d]

[a] State Key Laboratory of Organometallic Chemistry and Shanghai Hongkong Joint Laboratory in Chemical Synthesis, Shanghai Institute of Organic Chemistry, University of Chinese Academy of Sciences, Chinese Academy of Sciences, 345 Lingling Road, Shanghai 200032, China.

[b] Frontier Science Center for Transformative Molecules, School of Chemistry and Chemical Engineering, Shanghai Jiao Tong University, 800 Dongchuan Road, Shanghai 200240, China.

[c] School of Chemistry and Materials Science, Hangzhou Institute for Advanced Study, University of Chinese Academy of Sciences, 1 Sublane Xiangshan, Hangzhou 310024, China.

[d] School of Chemistry and Chemical Engineering, Henan Normal University, Xinxiang 453007, China.

Email: kding@sioc.ac.cn (K. D.); xiaoming@sioc.ac.cn (X. W.)

Table of Contents

1. General information 3

2. Experimental data 4

2.1 Synthesis of (*S, S, S*)-**L1**. 4

2.2 Optimization of the reaction conditions (Table S1-S4) 13

2.3 General Procedure for asymmetric hydroboration of ketones. 16

2.4 Kinetic studies. 35

3. X-ray crystallographic 51

3.1 X-ray Crystallographic for (*S, S, S*)-**L1** 51

3.2 X-ray Crystallographic for Zn_3**L1** 54

4. References 56

5. NMR spectra 58

6. HPLC chromatograms 115

1. General information

Unless otherwise noted below, commercially available reagents were used throughout without further purification, and all the reactions were carried out under an argon atmosphere condition and glassed were dried in an oven before use. Dry solvents were purchased and stored with molecular sieves in an atmosphere of argon. Column chromatography was performed using 200-300 mesh silica gel. ^{1}H, ^{13}C, ^{19}F NMR spectra were recorded on Varian 400 MHz, Agilent 400 MHz, Agilent 500 MHz, Bruker 400 MHz spectrometers. Chemical shifts (*δ* values) were reported in ppm related to internal TMS (^{1}H NMR: 0.000 ppm) or $CDCl_3$ (^{1}H NMR: 7.260 ppm), respectively. The following abbreviations (or combination thereof) were used to explain the multiplicities: s = singlet, d = doublet, t = triplet, q = quartet, m = multiplet, br = broad. HRMS (ESI) were determined on Agilent 6224 TOF LC/MS. HPLC analyses was carried out with Agilent 1260 Infinity on a UV spectrophotometric detector (Agilent) and JASCO 2089 liquid chromatograph. Optical rotations were determined using a Rudolph Autopol I polarimeter. The IR spectra were measured on a BRUKER TENSOR 27 FT-IR spectrometer.

2. Experimental Data

2.1 Synthesis of (*S, S, S*)-L1.

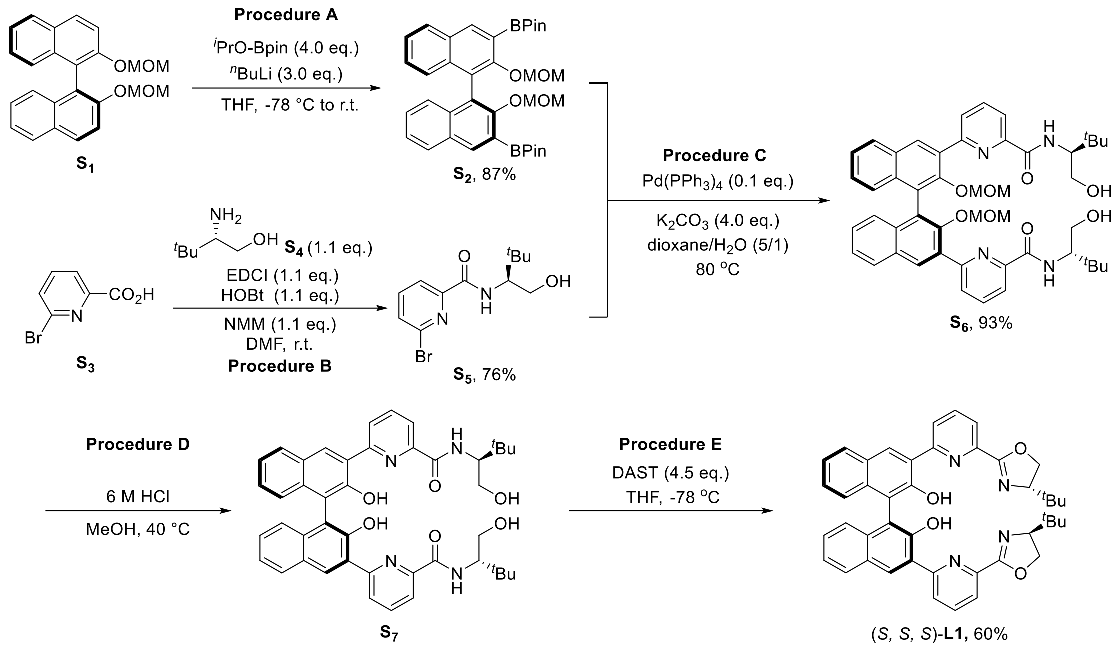

Procedure A: According to the reported procedure[1], to a flame-dried Schlenk flask equipped with a magnetic stir bar, containing (*S*)-2,2'-bis(methoxymethoxy)-1,1'-binaphthyl **S1** (7.48 g, 20.0 mmol) in dry THF (80 mL) was added ^{n}BuLi (2.5 M in *n*-hexane, 24.0 mL, 60.0 mmol) at 0 °C under argon. The reaction mixture was stirred at room temperature for 3 h, then the resulting light brown suspension was cooled to -78 °C, and 2-isopropoxy-4,4,5,5-tetramethyl -1,3,2-dioxaborolane (^{i}PrO-BPin) (16.6 mL, 80.0 mmol) was added over 10 min. The solution was allowed to warm to room temperature and left stirring overnight. The reaction mixture was quenched with NH_4Cl aqueous solution and extracted with ethyl acetate. The combined organic layer was washed with brine and dried with anhydrous Na_2SO_4. After removal of the solvent under reduced pressure, the residue was purified by silica gel column chromatography (petroleum ether/ethyl acetate, 5/1) to give target compound **S2** (10.86 g) as white solid in 87% yield.

Procedure B: To a flame-dried round bottom flask equipped with a magnetic stir bar was added 6-bromopicolinic acid **S3** (2.02 g, 10 mmol), L-*tert*-Leucine **S4** (1.29 g, 1.1 equiv), *n*-(3-dimethylaminopropyl)-*n'*-ethylcarbodiimide hydrochloride (EDCI) (2.1 g,

1.1 equiv), 1-Hydroxybenzotriazole (HOBt) (1.49 g, 1.1 equiv) and *N*-Methyl morpholine (NMM) (1.2 mL, 1.1 equiv) in DMF (10 mL). The reaction mixture was stirred at room temperature for 4 h. After completion, ethyl acetate was added and the mixture was washed with water for 5 times. The combined organic layer was washed with brine and dried with anhydrous Na_2SO_4. After removal of the solvent under reduced pressure, the residue was purified by silica gel column chromatography (petroleum ether/ethyl acetate, 1/1) to give target compound **S5** (2.30 g) as white solid in 76% yield.

Procedure C: To a flame-dried Schlenk flask equipped with a magnetic stir bar, was added **S2** (1.25 g, 2.0 mmol), **S5** (1.26 g, 2.1 equiv), $Pd(PPh_3)_4$ (0.23 g, 10 mol%), anhydrous K_2CO_3 (1.10 g, 4.0 equiv) and 1,4-dioxane/H_2O (20 mL / 4 mL) under argon. The reaction mixture was stirred at 80 °C overnight. The reaction mixture was quenched with water and extracted with DCM. The combined organic layer was washed with brine and dried with anhydrous Na_2SO_4. After removal of the solvent under reduced pressure, the residue was purified by silica gel column chromatography (DCM/MeOH, 100/1) to give target compound **S6** (1.51 g) as white solid in 93% yield.

Procedure D: To a flame-dried round bottom flask equipped with a magnetic stir bar was added **S6** (1.08 g, 1.33 mmol) and MeOH (10 mL). Then HCl (1.5 mL, 6 M) was added dropwise. The reaction mixture was stirred at 40 °C for 4 h. After completion, the reaction mixture was quenched with $NaHCO_3$ aqueous solution and extracted with DCM. The combined organic layer was washed with brine and dried with anhydrous Na_2SO_4. After removal of the solvent under reduced pressure, the crude product **S7** (1.04 g) was obtained as yellow solid.

Procedure E: To a flame-dried Schlenk flask equipped with a magnetic stir bar, was added **S7** (1.02 g, 1.34 mmol) and dry THF (20 mL) under argon. The reaction mixture was cooled to -78 °C, diethylaminosulfur trifluoride (DAST) (0.8 mL, 4.5 equiv) was added over 10 min, and was stirred at -78 °C for 6 h. After completion, the solution was allowed to warm to room temperature, then was quenched with $NaHCO_3$ aqueous solution and extracted with DCM. The combined organic layer was washed with brine

and dried with anhydrous Na_2SO_4. After removal of the solvent under reduced pressure, the residue was purified by silica gel column chromatography (DCM/MeOH, 200/1) to give target compound (*S, S, S*)-**L1** (0.55 g) as yellow solid in 60% yield.

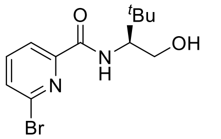

(*S*)-6-bromo-*N*-(1-hydroxy-3,3-dimethylbutan-2-yl)picolinamide (**S5**)

White solid, 2.30 g, 76% yield, **M.P.** 57-59 °C, $[\alpha]_D^{25}$ = -2.2 (c 0.50, $CHCl_3$).

^{1}H NMR (400 MHz, $CDCl_3$) δ 8.12 (d, *J* = 7.5 Hz, 1H), 7.99 (d, *J* = 9.0 Hz, 1H), 7.74 – 7.65 (m, 1H), 7.59 (d, *J* = 8.0 Hz, 1H), 4.03 – 3.92 (m, 2H), 3.68 (dd, *J* = 11.5, 8.6 Hz, 1H), 1.03 (d, *J* = 1.5 Hz, 9H).

^{13}C NMR (101 MHz, $CDCl_3$) δ 163.7, 150.7, 140.4, 139.6, 130.7, 121.4, 62.8, 60.2, 33.8, 26.9.

HRMS-ESI (m/z): $[M+Na]^+$ calcd for $C_{12}H_{17}N_2O_2NaBr^+$: 323.0366; found: 323.0365.

IR (neat) ν 3420, 3274, 3052, 2972, 1646, 1530, 1427 cm^{-1}.

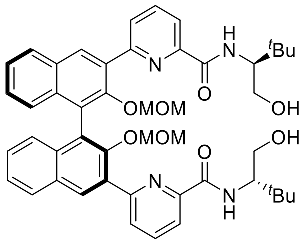

6,6'-((*S*)-2,2'-bis(methoxymethoxy)-[1,1'-binaphthalene]-3,3'-diyl)bis(*N*-((*S*)-1-hydroxy-3,3-dimethylbutan-2-yl)picolinamide) (**S6**)

White solid, 1.51 g, 93% yield, **M.P.** 129-131 °C, $[\alpha]_D^{25}$ = -17.0 (c 0.50, $CHCl_3$).

^{1}H NMR (400 MHz, $CDCl_3$) δ 8.56 (d, *J* = 9.0 Hz, 2H), 8.31 (s, 2H), 8.22 (d, *J* = 7.5 Hz, 2H), 8.09 (d, *J* = 7.9 Hz, 2H), 7.97 (t, *J* = 7.3 Hz, 4H), 7.49 (dt, *J* = 8.2, 3.9 Hz, 2H), 7.37 (d, *J* = 4.0 Hz, 4H), 4.58 – 4.36 (m, 4H), 4.07 – 3.97 (m, 4H), 3.75 (t, *J* = 9.5 Hz, 2H), 3.01 (s, 2H), 2.43 (s, 6H), 1.92 (s, 2H), 1.05 (s, 18H).

^{13}C NMR (101 MHz, $CDCl_3$) δ 165.4, 155.4, 151.1, 149.5, 137.8, 134.1, 133.2, 131.6,

130.7, 128.5, 127.3, 127.3, 126.5, 126.2, 125.6, 120.9, 99.0, 63.2, 60.3, 56.1, 33.9, 27.0.

HRMS-ESI (m/z): $[M+H]^+$ calcd for $C_{48}H_{55}N_4O_8^+$: 815.4014; found: 815.4013.

IR (neat) ν 3363, 2955, 2872, 1664, 1522, 1459, 1350 cm^{-1}.

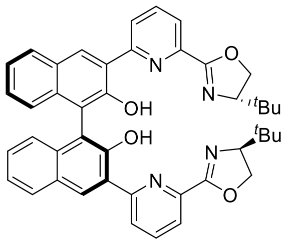

(*S*)-3,3'-bis(6-((*S*)-4-(*tert*-butyl)-4,5-dihydrooxazol-2-yl)pyridin-2-yl)-[1,1'-binaphthalene]-2,2'-diol ((*S, S, S*)-**L1**)

Yellow solid, 0.55 g, 60% yield, **M.P.** 200-202 °C, $[\alpha]_D^{25}$ = 4.0 (c 0.50, $CHCl_3$).

^{1}H NMR (400 MHz, $CDCl_3$) δ 13.61 (s, 2H), 8.52 (s, 2H), 8.32 (d, *J* = 8.2 Hz, 2H), 8.10 (d, *J* = 7.7 Hz, 2H), 7.99 – 7.88 (m, 4H), 7.40 – 7.16 (m, 6H), 4.27 (t, *J* = 9.5 Hz, 2H), 4.14 (t, *J* = 8.5 Hz, 2H), 4.09 – 3.92 (m, 2H), 0.89 (s, 18H).

^{13}C NMR (101 MHz, $CDCl_3$) δ 161.6, 157.7, 154.5, 143.7, 138.2, 135.5, 128.7, 127.6, 127.5, 127.5, 124.9, 123.1, 122.1, 122.0, 121.1, 118.7, 76.4, 69.3, 33.9, 25.8.

HRMS-ESI (m/z): $[M+H]^+$ calcd for $C_{44}H_{43}N_4O_4^+$: 691.3284; found: 691.3270.

IR (neat) ν 3053, 2952, 2867, 1650, 1568, 1645, 1361 cm^{-1}.

A series of BINOL-di-pyox ligands **L1-L8** were also successfully synthesized by following the similar procedure.

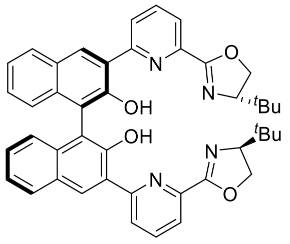

3,3'-bis(6-((S)-4-(*tert*-butyl)-4,5-dihydrooxazol-2-yl)pyridin-2-yl)-[1,1'-binaphthalene]-2,2'-diol ((*R, S, S*)-**L1**)

Yellow solid, 0.63 g, 70% yield, **M.P.** 201-203 °C, $[\alpha]_D^{25}$ = -275.4 (c 0.50, $CHCl_3$).

^{1}H NMR (400 MHz, $CDCl_3$) δ 13.60 (s, 2H), 8.52 (s, 2H), 8.32 (d, *J* = 8.2 Hz, 2H),

8.09 (d, J = 7.7 Hz, 2H), 7.98 (t, J = 8.0 Hz, 2H), 7.89 (d, J = 7.9 Hz, 2H), 7.40 – 7.16 (m, 6H), 4.27 (t, J = 9.5 Hz, 2H), 4.15 (t, J = 8.5 Hz, 2H), 4.09 – 3.92 (m, 2H), 0.89 (s, 18H).

^{13}C NMR (126 MHz, CDCl$_3$) δ 161.5, 157.5, 154.5, 143.5, 138.3, 135.5, 128.8, 127.5, 127.4, 124.8, 123.0, 122.1, 122.0, 121.0, 118.7, 76.3, 69.2, 33.9, 25.8.

HRMS-ESI (m/z): [M+H]$^+$ calcd for $C_{44}H_{43}N_4O_4^+$: 691.3272; found: 691.3279.

IR (neat) ν 2953, 2902, 1650, 1570, 1466, 1361 cm^{-1}.

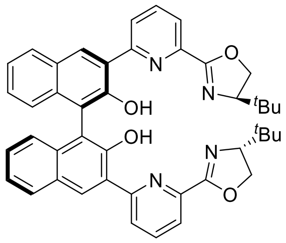

3,3'-bis(6-((*R*)-4-(*tert*-butyl)-4,5-dihydrooxazol-2-yl)pyridin-2-yl)-[1,1'-binaphthalene]-2,2'-diol ((*R, R, R*)-**L1**)

Yellow solid, 0.50 g, 68% yield, **M.P.** 202-204 °C, $[\alpha]_D^{25}$ = -6.2 (c 0.50, CHCl$_3$).

^{1}H NMR (400 MHz, CDCl$_3$) δ 13.63 (s, 2H), 8.51 (s, 2H), 8.31 (d, J = 8.2 Hz, 2H), 8.09 (d, J = 7.7 Hz, 2H), 7.96 (t, J = 8.0 Hz, 2H), 7.92 – 7.82 (m, 2H), 7.30 – 7.16 (m, 6H), 4.27 (t, J = 9.5 Hz, 2H), 4.14 (t, J = 8.5 Hz, 2H), 4.02 (dd, J = 10.2, 8.2 Hz, 2H), 0.90 (s, 18H).

^{13}C NMR (126 MHz, CDCl$_3$) δ 161.6, 157.6, 154.4, 143.6, 138.2, 135.5, 128.8, 127.6, 127. 5, 127.4, 124.8, 123.0, 122.1, 121.9, 121.2, 118.7, 76.3, 69.2, 33.9, 25.8.

HRMS-ESI (m/z): [M+H]$^+$ calcd for $C_{44}H_{43}N_4O_4^+$: 691.3274; found: 691.3279.

IR (neat) ν 2954, 2901, 1651, 1569, 1467, 1362 cm^{-1}.

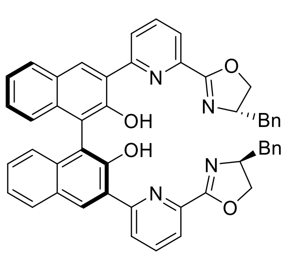

(*S*)-3,3'-bis(6-((*S*)-4-benzyl-4,5-dihydrooxazol-2-yl)pyridin-2-yl)-[1,1'-

binaphthalene]-2,2'-diol ((*S, S, S*)-**L2**)

Yellow solid, 0.48 g, 58% yield, **M.P.** 193-195 °C, $[\alpha]_D^{25}$ = 113.0 (c 0.50, $CHCl_3$).

^{1}H NMR (400 MHz, $CDCl_3$) δ 13.60 (s, 2H), 8.53 (s, 2H), 8.34 (d, *J* = 8.0 Hz, 2H), 8.07 – 7.97 (m, 4H), 7.91 (d, *J* = 7.9 Hz, 2H), 7.32 – 7.23 (m, 8H), 7.20 (t, *J* = 8.0 Hz, 8H), 4.64 – 4.46 (m, 2H), 4.29 (t, *J* = 9.1 Hz, 2H), 4.10 (t, *J* = 8.1 Hz, 2H), 3.19 (dd, *J* = 13.7, 5.2 Hz, 2H), 2.66 (dd, *J* = 13.7, 9.0 Hz, 2H).

^{13}C NMR (101 MHz, $CDCl_3$) δ 162.1, 157.7, 154.5, 143.4, 138.3, 137.6, 135.5, 129.1, 128.8, 128.5, 127.5, 127.4, 126.5, 124.8, 123.1, 122.2, 121.9, 121.0, 118.7, 72.4, 68.0, 41.6.

HRMS-ESI (m/z): $[M+H]^+$ calcd for $C_{50}H_{39}N_4O_4^+$: 759.2966; found: 759.2962.

IR (neat) ν 1646, 1569, 1464, 1210, 732, 699 cm^{-1}.

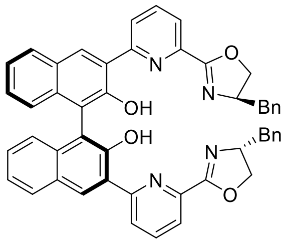

(*S*)-3,3'-bis(6-((*R*)-4-benzyl-4,5-dihydrooxazol-2-yl)pyridin-2-yl)-[1,1'-binaphthalene]-2,2'-diol ((*S, R, R*)-**L2**)

Yellow solid, 0.64 g, 57% yield, **M.P.** 196-198 °C, $[\alpha]_D^{25}$ = 220.4 (c 0.50, $CHCl_3$).

^{1}H NMR (400 MHz, $CDCl_3$) δ 13.70 (s, 2H), 8.48 (s, 2H), 8.28 (dd, *J* = 7.6, 1.7 Hz, 2H), 8.06 – 7.92 (m, 4H), 7.91 – 7.81 (m, 2H), 7.33 – 7.20 (m, 10H), 7.20 – 7.09 (m, 6H), 4.64 – 4.41 (m, 2H), 4.24 (t, *J* = 9.1 Hz, 2H), 4.04 (t, *J* = 8.1 Hz, 2H), 3.13 (dd, *J* = 13.7, 5.3 Hz, 2H), 2.64 (dd, *J* = 13.7, 8.8 Hz, 2H).

^{13}C NMR (101 MHz, $CDCl_3$) δ 162.2, 157.7, 154.5, 143.4, 138.4, 137.6, 135.6, 129.2, 128.8, 128.5, 127.5, 127.5, 126.5, 124.9, 123.2, 122.2, 121.9, 121.0, 118.8, 72.4, 68.1, 41.6.

HRMS-ESI (m/z): $[M+H]^+$ calcd for $C_{50}H_{39}N_4O_4^+$: 759.2966; found: 759.2963.

IR (neat) ν 1645, 1568, 1464, 1361, 732, 699 cm^{-1}.

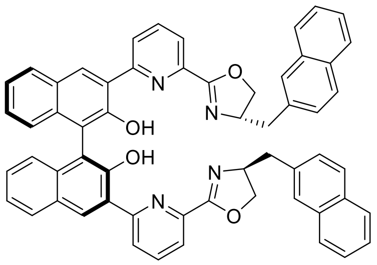

(*S*)-3,3'-bis(6-((*S*)-4-(naphthalen-2-ylmethyl)-4,5-dihydrooxazol-2-yl)pyridin-2-yl)-[1,1'-binaphthalene]-2,2'-diol ((*S, S, S*)-**L3**)

Yellow solid, 0.26 g, 39% yield, **M.P.** 188-190 °C, $[\alpha]_D^{25}$ = 144.4 (c 0.50, $CHCl_3$).

^{1}H NMR (400 MHz, $CDCl_3$) δ 13.60 (s, 2H), 8.54 (s, 2H), 8.34 (d, *J* = 7.9 Hz, 2H), 8.12 – 7.97 (m, 4H), 7.91 (d, *J* = 7.9 Hz, 2H), 7.76 (dd, *J* = 12.7, 8.6 Hz, 6H), 7.63 (s, 2H), 7.42 – 7.41 (m, 4H), 7.34 (dd, *J* = 8.4, 1.7 Hz, 2H), 7.29 (dt, *J* = 4.6, 2.3 Hz, 2H), 7.25 – 7.19 (m, 4H), 4.65 (td, *J* = 8.5, 5.5 Hz, 2H), 4.30 (t, *J* = 9.0 Hz, 2H), 4.16 (t, *J* = 8.1 Hz, 2H), 3.35 (dd, *J* = 13.8, 5.3 Hz, 2H), 2.83 (dd, *J* = 13.8, 8.9 Hz, 2H).

^{13}C NMR (101 MHz, $CDCl_3$) δ 162.2, 157.8, 154.6, 143.4, 138.3, 135.6, 135.2, 133.4, 132.2, 128.8, 128.1, 127.5, 127.5, 126.0, 125.4, 124.9, 123.1, 122.2, 121.9, 121.0, 118.8, 72.5, 68.0, 41.8.

HRMS-ESI (m/z): $[M+H]^+$ calcd for $C_{58}H_{43}N_4O_4^+$: 859.3279; found: 859.3276.

IR (neat) ν 1647, 1579, 1466, 1211, 817, 734 cm^{-1}.

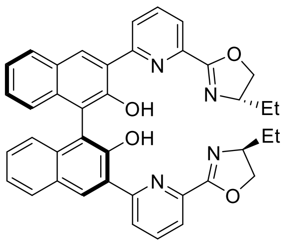

(*S*)-3,3'-bis(6-((*S*)-4-ethyl-4,5-dihydrooxazol-2-yl)pyridin-2-yl)-[1,1'-binaphthalene]-2,2'-diol ((*S, S, S*)-**L4**)

Yellow solid, 0.34 g, 51% yield, **M.P.** 181-183 °C, $[\alpha]_D^{25}$ = 53.6 (c 0.50, $CHCl_3$).

^{1}H NMR (400 MHz, $CDCl_3$) δ 13.67 (d, *J* = 5.1 Hz, 2H), 8.49 (d, *J* = 1.7 Hz, 2H), 8.28 (dd, *J* = 8.1, 1.7 Hz, 2H), 8.04 – 7.90 (m, 4H), 7.90 – 7.81 (m, 2H), 7.23 (m, 6H), 4.37 (m, 2H), 4.26 – 4.11 (m, 2H), 4.00 – 3.89 (m, 2H), 1.75 – 1.62 (m, 2H), 1.53 (dp, *J* =

14.3, 7.3 Hz, 2H), 0.98 – 0.86 (t, *J* = 7.4 Hz, 6H).

^{13}C NMR (101 MHz, CDCl$_3$) δ 161.7, 157.7, 154.5, 143.6, 138.3, 135.5, 128.8, 127.6, 127.5, 127.5, 124.9, 123.1, 122.1, 121.9, 121.1, 118.7, 72.6, 68.2, 28.4, 10.0.

HRMS-ESI (m/z): [M+H]$^+$ calcd for $C_{40}H_{35}N_4O_4^+$: 635.2653; found: 635.2661.

IR (neat) ν 2961, 1647, 1569, 1464, 819, 714 cm^{-1}.

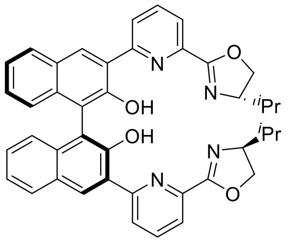

(*S*)-3,3'-bis(6-((*S*)-4-isopropyl-4,5-dihydrooxazol-2-yl)pyridin-2-yl)-[1,1'-binaphthalene]-2,2'-diol ((*S, S, S*)-**L5**)

Yellow solid, 0.46 g, 57% yield, **M.P.** 183-185 °C, $[\alpha]_D^{25}$ = 29.8(c 0.50, CHCl$_3$).

^{1}H NMR (400 MHz, CDCl$_3$) δ 13.66 (s, 2H), 8.55 (s, 2H), 8.44 – 8.26 (m, 2H), 8.12 – 8.07 (m, 2H), 8.06 – 7.96 (m, 2H), 7.95 – 7.90 (m, 2H), 7.36 – 7.23 (m, 6H), 4.55 – 4.24 (m, 2H), 4.23 – 3.94 (m, 4H), 1.84 (m, 2H), 1.01 (d, *J* = 6.7 Hz, 6H), 0.91 (d, *J* = 6.7 Hz, 6H).

^{13}C NMR (101 MHz, CDCl$_3$) δ 161.7, 157.7, 154.5, 143.8, 138.2, 135.6, 128.7, 127.6, 127.51, 127.46, 124.9, 123.1, 122.1, 122.0, 121.1, 118.7, 72.8, 70.7, 32.7, 18.9, 18.1.

HRMS-ESI (m/z): [M+H]$^+$ calcd for $C_{42}H_{39}N_4O_4^+$: 663.2967; found: 663.2958.

IR (neat) ν 2956, 1648, 1569, 1464, 820, 734 cm^{-1}.

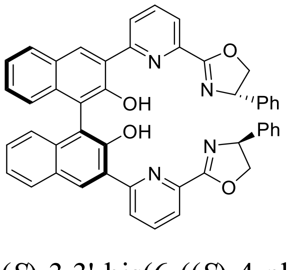

(*S*)-3,3'-bis(6-((*S*)-4-phenyl-4,5-dihydrooxazol-2-yl)pyridin-2-yl)-[1,1'-binaphthalene]-2,2'-diol ((*S, S, S*)-**L6**)

Yellow solid, 0.28 g, 67% yield, **M.P.** 184-186 °C, $[\alpha]_D^{25}$ = -97.4 (c 0.50, CHCl$_3$).

^{1}H NMR (400 MHz, CDCl$_3$) δ 13.61 (s, 2H), 8.55 (s, 2H), 8.37 (d, *J* = 8.3 Hz, 2H), 8.15 (d, *J* = 7.7 Hz, 2H), 8.01 (t, *J* = 8.0 Hz, 2H), 7.92 (d, *J* = 8.0 Hz, 2H), 7.39 – 7.16 (m, 18H), 5.36 (t, *J* = 9.4 Hz, 2H), 4.74 (t, *J* = 9.5 Hz, 2H), 4.24 (t, *J* = 8.6 Hz, 2H).

^{13}C NMR (101 MHz, CDCl$_3$) δ 162.9, 157.8, 154.5, 143.4, 141.6, 138.4, 135.6, 128.8, 127.7, 127.6, 127.5, 126.7, 124.9, 123.2, 122.4, 122.2, 121.0, 118.8, 75.3, 70.3.

HRMS-ESI (m/z): $[M+H]^+$ calcd for $C_{48}H_{35}N_4O_4^+$: 731.2653; found: 731.2648.

IR (neat) ν 1645, 1569, 1464, 819, 733, 697 cm^{-1}.

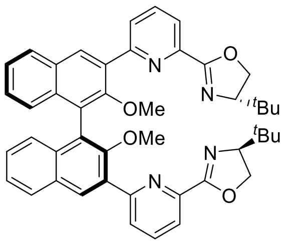

(*4S,4'S*)-2,2'-(((*S*)-2,2'-dimethoxy-[1,1'-binaphthalene]-3,3'-diyl)bis(pyridine-6,2-diyl))bis(4-(*tert*-butyl)-4,5-dihydrooxazole) (**L7**)

White solid, 0.22g, 51% yield, **M.P.** 114-116 °C, $[\alpha]_D^{25}$ = -64.4 (c 0.50, CHCl$_3$).

^{1}H NMR (400 MHz, CDCl$_3$) δ 8.48 (s, 2H), 8.15 (d, *J* = 7.7 Hz, 2H), 8.08 (d, *J* = 7.9 Hz, 2H), 8.00 (d, *J* = 8.3 Hz, 2H), 7.82 (t, *J* = 7.9 Hz, 2H), 7.43 – 7.37 (m, 2H), 7.31 – 7.23 (m, 2H), 7.14 (d, *J* = 8.5 Hz, 2H), 4.54 (t, *J* = 9.5 Hz, 2H), 4.39 (t, *J* = 8.6 Hz, 2H), 4.18 (dd, *J* = 10.3, 8.3 Hz, 2H), 3.22 (s, 6H), 1.02 (s, 18H).

^{13}C NMR (126 MHz, CDCl$_3$) δ 162.8, 156.3, 153.8, 147.1, 136.5, 134.3, 133.0, 132.1, 130.8, 128.8, 126.9, 126.8, 125.8, 125.6, 125.0, 122.6, 76.4, 69.4, 61.1, 34.0, 26.0.

HRMS-ESI (m/z): $[M+H]^+$ calcd for $C_{46}H_{47}N_4O_4^+$: 719.3592; found: 719.3593.

IR (neat) ν 2952, 1645, 1497, 1460, 1361, 745 cm^{-1}.

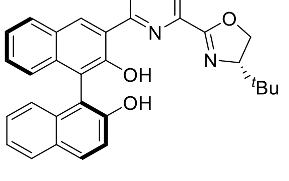

(*S*)-3-(6-((*S*)-4-(*tert*-butyl)-4,5-dihydrooxazol-2-yl)pyridin-2-yl)-[1,1'-binaphthalene]-2,2'-diol (**L8**)

White solid, 0.56 g, 67% yield, **M.P.** 169-171 °C, $[\alpha]_D^{25}$ = -207.2 (c 0.50, $CHCl_3$).

^{1}H NMR (400 MHz, $CDCl_3$) δ 13.83 (d, J = 8.7 Hz, 1H), 8.40 (d, J = 5.8 Hz, 2H), 8.16 – 7.80 (m, 5H), 7.75 (t, J = 8.0 Hz, 1H), 7.45 (d, J = 8.9 Hz, 1H), 7.42 – 7.10 (m, 6H), 6.41 (s, 1H), 4.14 (dq, J = 12.8, 8.7 Hz, 2H), 3.72 – 3.19 (m, 1H), 0.90 (s, 9H).

^{13}C NMR (126 MHz, $CDCl_3$) δ 161.2, 156.3, 155.3, 152.0, 142. 9, 138.4, 135.7, 133.8, 129.8, 129.2, 128.8, 128.20, 128.19, 127.6, 126.3, 124.9, 124.8, 123.8, 123.1, 122.6, 121.4, 120.9, 118.3, 115.4, 115.2, 75.8, 69.3, 33.8, 25.8.

HRMS-ESI (m/z): $[M+H]^+$ calcd for $C_{32}H_{29}N_2O_3^+$: 489.2173; found: 489.2166.

IR (neat) ν 2951, 1646, 1573, 1467, 847, 748 cm^{-1}.

2.2 Optimization of the reaction conditions (Table S1-S4)

Table S1. Effect of the ligands[a].

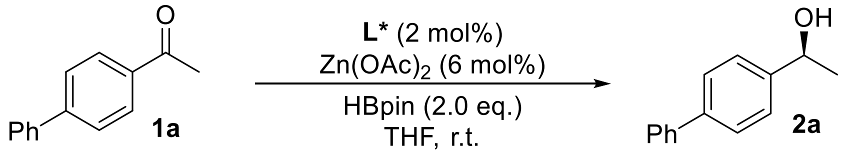

Entry	L*	Conv. (%)[b]	Yield (%)[c]	*ee* (%)[d]
1	(*S, S, S*)-**L1**	>99	99	90 (*S*)
2	(*R, S, S*)-**L1**	>99	99	80 (*R*)
3	(*R, R, R*)-**L1**	>99	99	90 (*R*)
4	(*S, S, S*)-**L2**	93	92	89 (*S*)
5	(*S, R, R*)-**L2**	90	89	88 (*S*)
6	(*S, S, S*)-**L3**	>99	99	90 (*S*)
7	(*S, S, S*)-**L4**	>99	99	90 (*S*)
8	(*S, S, S*)-**L5**	>99	99	90 (*S*)
9	(*S, S, S*)-**L6**	89	88	88 (*S*)
10[e]	(*S, S, S*)-**L1**	99	99	92 (*S*)
11[f]	(*S, S, S*)-**L7**	45	34	*rac.*
12[f]	**L8**	24	16	16 (*S*)
13[f]	**L9**	93	90	*rac.*

14[f]	**L10**	17	15	6 (*S*)
15[f]	**L9 + L10**	22	17	4 (*S*)

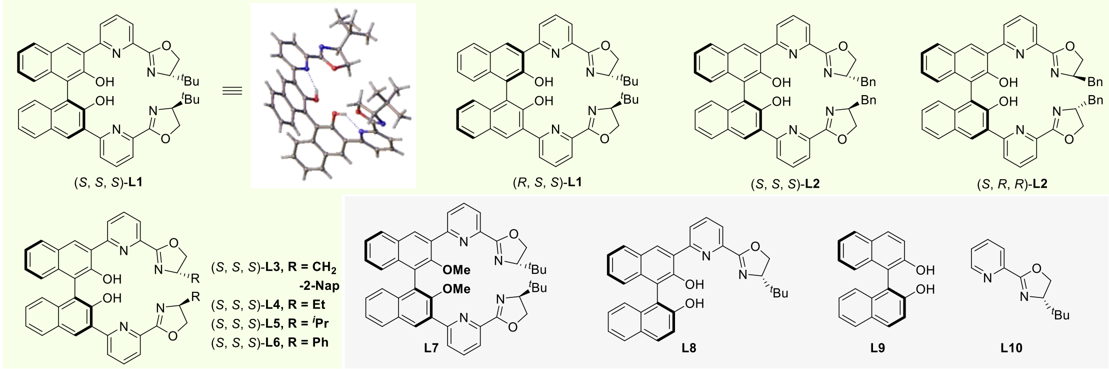

[a]Reaction conditions: **1a** (0.1 mmol), HBpin (2.0 equiv), **L*** (2 mol%), $Zn(OAc)_2$ (6 mol%), THF (1.0 mL), r.t., 4 h. [b]The conversion of **1a** was determined by GC using *n*-decane as the internal standard. [c]Isolated yield. [d]The *ee* values of **2a** were determined by chiral HPLC.[e]10 °C, 24 h. [f]**L*** (6 mol%).

Table S2. Zn_3**L1** complex as the catalyst[a]

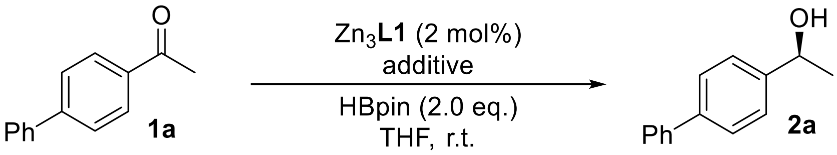

Entry	additive	Conv. (%)[b]	*ee* (%)[c]
1	-	99	84
2	KOAc (1.0 mol%)	99	90
3	KOAc (10 mol%)	99	90

[a]Reaction conditions: **1a** (0.1 mmol), HBpin (2.0 equiv), Zn_3**L1** (2 mol%), KOAc, THF (1.0 mL), r.t., 4 h. [b]The conversion of **1a** was determined by GC using *n*-decane as the internal standard. [c]The *ee* values of **2a** were determined by chiral HPLC.

Table S3. Effect of **[Zn]**[a].

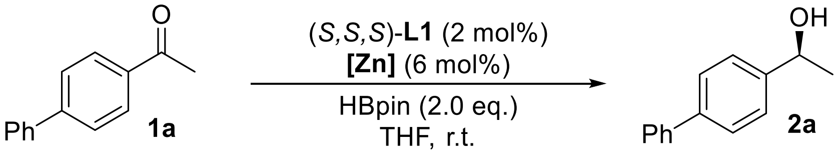

Entry	**[Zn]**	Conv. (%)[b]	*ee* (%)[c]
1	$Zn(OAc)_2$	99	90
2	$Zn(PhCO_2)_2$	82	86
3	$Zn(HCO_2)_2$	29	86
4	$Zn(NTf)_2$	48	*rac.*
5	$ZnClO_4•6H_2O$	13	*rac.*
6	$ZnSO_4$	-	-
7	$Zn(OTf)_2$	35	9
8	$ZnCl_2$	35	26
9	$ZnBr_2$	13	26
10	ZnI_2	18	16
11	Et_2Zn	98	*rac.*

[a]Reaction conditions: **1a** (0.1 mmol), HBpin (2.0 equiv), (*S, S, S*)-**L1** (2 mol%), **[Zn]** (6 mol%), THF (1.0 mL), r.t., 4 h. [b]The conversion of **1a** was determined by GC using *n*-decane as the internal standard. [c]The *ee* values of **2a** were determined by chiral HPLC.

Table S4. The loading of the catalyst[a].

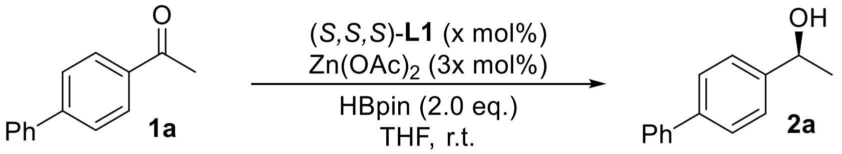

Entry	**1a** (mmol)	**L1** (mol%)	THF (mL)	Time (h)	Conv. (%)[b]	*ee* (%)[c]	TON
1	0.1	2	1.0	4	>99	90	-
2	0.1	1	1.0	4	>99	90	-
3	2.0	0.1	5.0	6	>99	90	-
4[d]	2.0	0.1	5.0	24	99 (98)[e]	92	-
5	20	0.01	40	12	>99	90	-
6	40	0.005	70	24	99 (97)[e]	90	19400
7	100	0.002	130	48	99 (93)[e]	80	46500

8	200	0.001	270	48	50	79	-
9[f]	0.1	-	1.0	4	99	*rac.*	-
10[g]	40	-	70	24	22	*rac.*	-

[a]Reaction conditions: **1a** (0.1-200 mmol), HBpin (2.0 equiv), (*S, S, S*)-**L1** (0.002 mmol), $Zn(OAc)_2$ (0.006 mmol), r.t. [b]The conversion of **1a** was determined by GC using *n*-decane as the internal standard. [c]The *ee* values of **2a** were determined by chiral HPLC. [d]0 °C. [e]Isolated yield in parenthesis. [f]$Zn(OAc)_2$ (6.0 mol%), without a chiral ligand. [g]$Zn(OAc)_2$ (0.015 mol%), without a chiral ligand.

2.3 General Procedure for asymmetric hydroboration of ketones.

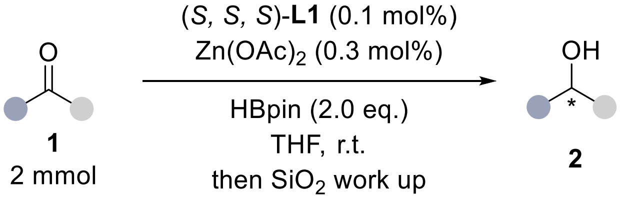

A typical experimental procedure for the tri-Zn catalyzed enantioselective hydroboration of ketone is described below. In an oven-dried 10 mL tube equipped with a stir bar was added $Zn(OAc)_2$ (1.1 mg, 0.006 mmol, 0.3 mol%), (*S, S, S*)-**L1** (1.4 mg, 0.002 mmol, 0.1mol%), **1** (2.0 mmol), HBpin (0.58 mL, 4.0 mmol, 2.0 equiv), THF (5.0 mL). The resulting mixture was stirred at r.t. for 12 h. Then the mixture was concentrated under reduced pressure. The residue was purified by silica gel chromatography to give the desired product **2**.

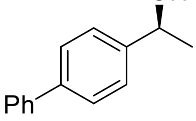

(*S*)-1-([1,1'-biphenyl]-4-yl)ethan-1-ol (**2a**)[2]

White solid, 98% isolated yield, 92% *ee*, $[\alpha]_D^{25}$ = -26.8 (c 0.5, $CHCl_3$).

^{1}H NMR (400 MHz, $CDCl_3$) δ 7.67 – 7.55 (m, 4H), 7.46 (m, 4H), 7.40 – 7.32 (m, 1H), 4.96 (q, J = 6.4 Hz, 1H), 1.96 (s, 1H), 1.55 (d, J = 6.4 Hz, 3H).

^{13}C NMR (101 MHz, $CDCl_3$) δ 144.8, 140.8, 140.4, 128.7, 127.2, 127.0, 125.8, 70.1, 25.1.

The enantiomeric excess was determined by HPLC on Chiralcel AD-H column, *n*-hexane : isopropanol = 95 : 5, flow rate = 1.0 mL/min, UV detection at λ = 210 nm, t_R = 13.0 min (major), t_R = 14.5 min (minor).

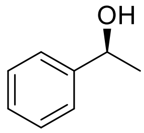

(*S*)-1-phenylethan-1-ol (**2b**)[2]

Colorless oil, 91% isolated yield, 92% *ee*, $[\alpha]_D^{25}$ = -46.8 (c 0.5, $CHCl_3$).

^{1}H NMR (400 MHz, $CDCl_3$) δ 7.38 – 7.24 (m, 5H), 4.85 (q, *J* = 6.5 Hz, 1H), 2.35 (s, 1H), 1.47 (d, *J* = 6.5 Hz, 3H).

^{13}C NMR (101 MHz, $CDCl_3$) δ 145.9, 128.5, 127.4, 125.4, 70.3, 25.2.

The enantiomeric excess was determined by HPLC on Chiralcel OD-H column, *n*-hexane : isopropanol = 95 : 5, flow rate = 1.0 mL/min, UV detection at λ = 210 nm, t_R = 9.0 min (minor), t_R = 10.7 min (major).

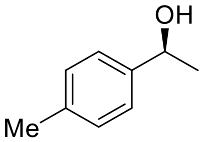

(*S*)-1-(*p*-tolyl)ethan-1-ol (**2c**)[2]

Colorless oil, 99% isolated yield, 91% *ee*, $[\alpha]_D^{25}$ = -45.9 (c 0.5, $CHCl_3$).

^{1}H NMR (400 MHz, $CDCl_3$) δ 7.25 (d, *J* = 7.7 Hz, 2H), 7.15 (d, *J* = 7.7 Hz, 2H), 4.83 (q, *J* = 6.5 Hz, 1H), 2.35 (s, 3H), 1.46 (d, *J* = 6.5 Hz, 3H).

^{13}C NMR (101 MHz, $CDCl_3$) δ 142.9, 137.1, 129.1, 125.4, 70.1, 25.1, 21.1.

The enantiomeric excess was determined by HPLC on Chiralcel IA-3 column, *n*-hexane : isopropanol = 97 : 3, flow rate = 1.0 mL/min, UV detection at λ = 254 nm, t_R = 11.0 min (minor), t_R = 11.6 min (major).

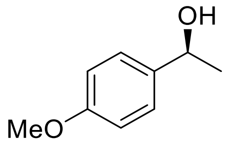

(*S*)-1-(4-methoxyphenyl)ethan-1-ol (**2d**)[2]

Colorless oil, 99% isolated yield, 89% *ee*, $[\alpha]_D^{25}$ = -21.8 (c 0.5, $CHCl_3$).

^{1}H NMR (400 MHz, $CDCl_3$) δ 7.30 (d, *J* = 8.7 Hz, 2H), 6.88 (d, *J* = 8.7 Hz, 2H), 4.85 (q, *J* = 6.4 Hz, 1H), 3.80 (s, 3H), 1.91 (s, 1H), 1.47 (d, *J* = 6.4 Hz, 3H).

^{13}C NMR (101 MHz, $CDCl_3$) δ 158.9, 137.9, 126.6, 113.8, 69.9, 55.3, 25.0.

The enantiomeric excess was determined by HPLC on Chiralcel OD-H column, *n*-hexane : isopropanol = 95 : 5, flow rate = 1.0 mL/min, UV detection at λ = 210 nm, t_R = 11.9 min (minor), t_R = 13.5 min (major).

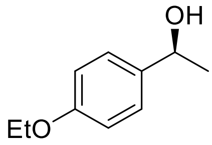

(*S*)-1-(4-ethoxyphenyl)ethan-1-ol (**2e**)[3]

Colorless oil, 99% isolated yield, 84% *ee*, $[\alpha]_D^{25}$ = -45.4 (c 0.5, $CHCl_3$).

^{1}H NMR (400 MHz, $CDCl_3$) δ 7.23 (d, *J* = 8.7 Hz, 2H), 6.83 (d, *J* = 8.7 Hz, 2H), 4.77 (q, *J* = 6.4 Hz, 1H), 3.99 (q, *J* = 7.0 Hz, 2H), 2.44 (s, 1H), 1.45 – 1.40 (m, 3H), 1.38 (m, 2H).

^{13}C NMR (101 MHz, $CDCl_3$) δ 158.1, 137.8, 126.5, 114.2, 69.7, 63.3, 24.9, 14.7.

The enantiomeric excess was determined by HPLC on Chiralcel OJ-H column, *n*-hexane : isopropanol = 95 : 5, flow rate = 1.0 mL/min, UV detection at λ = 210 nm, t_R = 19.8 min (minor), t_R = 22.0 min (major).

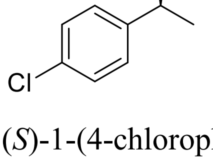

(*S*)-1-(4-chlorophenyl)ethan-1-ol (**2f**)[4]

Colorless oil, 82% isolated yield, 83% *ee*, $[\alpha]_D^{25}$ = -43.2 (c 0.5, $CHCl_3$).

^{1}H NMR (400 MHz, CDCl$_3$) δ 7.28 – 7.22 (m, 4H), 4.78 (q, J = 6.5 Hz, 1H), 2.59 (s, 1H), 1.40 (d, J = 6.4 Hz, 1H).

^{13}C NMR (101 MHz, CDCl$_3$) δ 144.2, 132.9, 128.5, 126.7, 69.5, 25.1.

The enantiomeric excess was determined by HPLC on Chiralcel OD-H column, *n*-hexane : isopropanol = 95 : 5, flow rate = 1.0 mL/min, UV detection at λ = 210 nm, t_R = 8.4 min (major), t_R = 9.3 min (minor).

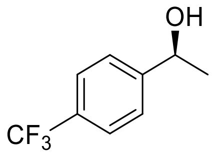

(*S*)-1-(4-(trifluoromethyl)phenyl)ethan-1-ol (**2g**)[5]

Colorless oil, 94% isolated yield, 80% *ee*, $[\alpha]_D^{25}$ = -24.4 (c 0.5, CHCl$_3$).

^{1}H NMR (400 MHz, CDCl$_3$) δ 7.56 (d, J = 8.0 Hz, 2H), 7.40 (d, J = 7.9 Hz, 2H), 4.85 (q, J = 6.5 Hz, 1H), 3.34 (s, 1H), 1.43 (d, J = 6.5 Hz, 3H).

^{13}C NMR (101 MHz, CDCl$_3$) δ 149.6, 129.5 (q, J = 32.2 Hz), 125.6, 125.3 (q, J = 3.8 Hz), 124.8 (q, J = 261.6 Hz), 69.6, 25.1.

^{19}F NMR (376 MHz, CDCl$_3$) δ -62.52.

The enantiomeric excess was determined by HPLC on Chiralcel OJ-3 column, *n*-hexane : isopropanol = 95 : 5, flow rate = 1.0 mL/min, UV detection at λ = 220 nm, t_R = 7.9 min (major), t_R = 8.6 min (minor).

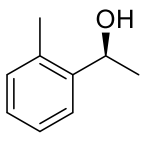

(*S*)-1-(*o*-tolyl)ethan-1-ol (**2h**)[2]

Colorless oil, 94% isolated yield, 86% *ee*, $[\alpha]_D^{25}$ = -67.6 (c 0.5, CHCl$_3$).

^{1}H NMR (400 MHz, CDCl$_3$) δ 7.53 – 7.45 (m, 1H), 7.28 – 7.22 (m, 1H), 7.20 – 7.16 (m, 1H), 7.14 – 7.10 (m, 1H), 5.05 (q, J = 6.3 Hz, 1H), 2.64 (s, 1H), 2.32 (s, 3H), 1.44 (d, J = 6.4 Hz, 3H).

^{13}C NMR (101 MHz, CDCl$_3$) δ 143.8, 134.0, 130.1, 126.9, 126.2, 124.4, 66.4, 23.8,

18.7.

The enantiomeric excess was determined by HPLC on Chiralcel OD-H column, *n*-hexane : isopropanol = 95 : 5, flow rate = 1.0 mL/min, UV detection at λ = 210 nm, t_R = 8.0 min (minor), t_R = 8.9 min (major).

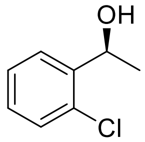

(*S*)-1-(2-chlorophenyl)ethan-1-ol (**2i**)[6]

Colorless oil, 99% isolated yield, 94% *ee*, $[\alpha]_D^{25}$ = -62.0 (c 0.5, $CHCl_3$).

^{1}H NMR (400 MHz, $CDCl_3$) δ 7.53 (d, *J* = 7.6 Hz, 1H), 7.29 – 7.21 (m, 2H), 7.17 – 7.13 (m, 1H), 5.22 (q, *J* = 6.4 Hz, 1H), 3.10 (s, 1H), 1.42 (d, *J* = 6.5 Hz, 3H).

^{13}C NMR (101 MHz, $CDCl_3$) δ 143.0, 131.4, 129.2, 128.2, 127.0, 126.3, 66.6, 23.4.

The enantiomeric excess was determined by HPLC on Chiralcel OD-H column, *n*-hexane : isopropanol = 98 : 2, flow rate = 1.0 mL/min, UV detection at λ = 210 nm, t_R = 14.0 min (minor), t_R = 14.9 min (major).

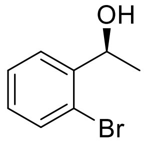

(*S*)-1-(2-bromophenyl)ethan-1-ol (**2j**)[6]

Colorless oil, 99% isolated yield, 92% *ee*, $[\alpha]_D^{25}$ = -52.6 (c 0.5, $CHCl_3$).

^{1}H NMR (400 MHz, $CDCl_3$) δ 7.56 – 7.44 (m, 2H), 7.32 – 7.29 (m, 1H), 7.11 – 7.07 (m, 1H), 5.18 (q, *J* = 6.4 Hz, 1H), 3.08 (s, 1H), 1.43 (d, *J* = 6.4 Hz, 3H).

^{13}C NMR (101 MHz, $CDCl_3$) δ 144.6, 132.4, 128.5, 127.7, 126.6, 121.5, 68.9, 23.5.

The enantiomeric excess was determined by HPLC on Chiralcel OD-H column, *n*-hexane : isopropanol = 98 : 2, flow rate = 1.0 mL/min, UV detection at λ = 210 nm, t_R = 14.3 min (major), t_R = 15.9 min (minor).

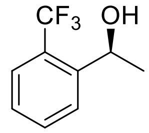

(S)-1-(2-(trifluoromethyl)phenyl)ethan-1-ol (**2k**)[6]

Colorless oil, 94% isolated yield, 97% *ee*, $[\alpha]_D^{25}$ = -30.2 (c 0.5, $CHCl_3$).

^{1}H NMR (400 MHz, $CDCl_3$) δ 7.76 (d, J = 7.9 Hz, 1H), 7.67 – 7.49 (m, 2H), 7.33 (t, J = 7.7 Hz, 1H), 5.27 (q, J = 6.5 Hz, 1H), 2.97 (s, 1H), 1.41 (d, J = 6.3 Hz, 3H).

^{13}C NMR (101 MHz, $CDCl_3$) δ 145.1, 132.3, 127.2, 127.1, 126.3 (q, J = 30.3 Hz). 125.2 (q, J = 5.9 Hz), 124.3 (q, J = 273.9 Hz), 65.5 (q, J = 2.5 Hz), 25.3.

^{19}F NMR (376 MHz, $CDCl_3$) δ -58.42.

The enantiomeric excess was determined by HPLC on Chiralcel AD-H column, *n*-hexane : isopropanol = 97 : 3, flow rate = 1.0 mL/min, UV detection at λ = 210 nm, t_R = 9.4 min (major), t_R = 10.7 min (minor).

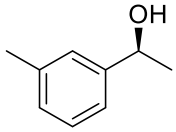

(S)-1-(*m*-tolyl)ethan-1-ol (**2l**)[2]

Colorless oil, 99% isolated yield, 91% *ee*, $[\alpha]_D^{25}$ = -47.8 (c 0.5, $CHCl_3$).

^{1}H NMR (400 MHz, $CDCl_3$) δ 7.24 (dd, J = 8.9, 6.1 Hz, 1H), 7.20 – 7.12 (m, 1H), 7.09 (d, J = 7.5 Hz, 1H), 4.82 (q, J = 6.5 Hz, 1H), 2.36 (s, 3H), 2.30 (s, 1H), 1.47 (d, J = 6.5 Hz, 3H).

^{13}C NMR (101 MHz, $CDCl_3$) δ 145.7, 138.0, 128.3, 128.1, 126.0, 122.4, 70.2, 25.0, 21.4.

The enantiomeric excess was determined by HPLC on Chiralcel OD-H column, *n*-hexane : isopropanol = 99 : 1, flow rate = 0.8 mL/min, UV detection at λ = 210 nm, t_R = 14.1 min (minor), t_R = 17.9 min (major).

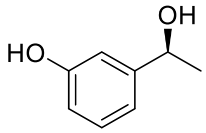

(*S*)-3-(1-hydroxyethyl)phenol (**2m**)[2]

White solid, 95% isolated yield, 90% *ee*, $[\alpha]_D^{25}$ = -9.0 (c 0.5, $CHCl_3$).

^{1}H NMR (400 MHz, CD_3OD) δ 7.17 – 7.02 (m, 1H), 6.78 (d, *J* = 7.4 Hz, 2H), 6.68 – 6.59 (m, 1H), 4.86 (s, 1H), 4.70 (q, *J* = 6.4 Hz, 1H), 1.37 (d, *J* = 6.4 Hz, 3H).

^{13}C NMR (101 MHz, CD_3OD) δ 158.2, 149.0, 130.1, 117.5, 114.7, 113.1, 70.6, 25.3.

The enantiomeric excess was determined by HPLC on Chiralcel OD-H column, *n*-hexane : isopropanol = 93 : 7, flow rate = 1.0 mL/min, UV detection at λ = 210 nm, t_R = 18.6 min (minor), t_R = 20.6 min (major).

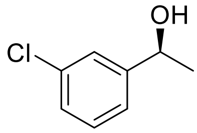

(*S*)-1-(3-chlorophenyl)ethan-1-ol (**2n**)[2]

Colorless oil, 94% isolated yield, 89% *ee*, $[\alpha]_D^{25}$ = -37.6 (c 0.5, $CHCl_3$).

^{1}H NMR (400 MHz, $CDCl_3$) δ 7.31 (d, *J* = 1.9 Hz, 1H), 7.28 – 7.15 (m, 3H), 4.78 (q, *J* = 6.1 Hz, 1H), 2.65 (s, 1H), 1.41 (d, *J* = 6.4 Hz, 3H).

^{13}C NMR (101 MHz, $CDCl_3$) δ 147.8, 134.2, 129.7, 127.4, 125.5, 123.5, 69.6, 25.1.

The enantiomeric excess was determined by HPLC on Chiralcel OJ-H column, *n*-hexane : isopropanol = 95 : 5, flow rate = 1.0 mL/min, UV detection at λ = 210 nm, t_R = 9.2 min (major), t_R = 10.4 min (minor).

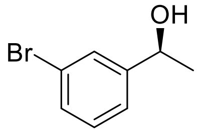

(*S*)-1-(3-bromophenyl)ethan-1-ol (**2o**)[2]

Colorless oil, 96% isolated yield, 90% *ee*, $[\alpha]_D^{25}$ = -31.8 (c 0.5, $CHCl_3$).

^{1}H NMR (400 MHz, $CDCl_3$) δ 7.48 (s, 1H), 7.37 (d, *J* = 7.3 Hz, 1H), 7.18 (dd, *J* =

14.4, 6.8 Hz, 2H), 4.76 (q, *J* = 6.4 Hz, 1H), 3.00 (s, 1H), 1.41 (d, *J* = 6.5 Hz, 3H).

^{13}C NMR (101 MHz, $CDCl_3$) δ 148.0, 130.2, 130.0, 128.4, 123.9, 122.4, 69.4, 25.1.

The enantiomeric excess was determined by HPLC on Chiralcel IB column, *n*-hexane : isopropanol = 95 : 5, flow rate = 1.0 mL/min, UV detection at λ = 210 nm, t_R = 7.7 min (major), t_R = 8.2 min (minor).

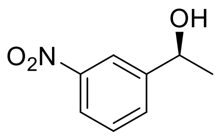

(*S*)-1-(3-nitrophenyl)ethan-1-ol (**2p**)[3]

Colorless oil, 99% isolated yield, 85% *ee*, $[\alpha]_D^{25}$ = -27.6 (c 0.5, $CHCl_3$).

^{1}H NMR (400 MHz, $CDCl_3$) δ 8.22 (s, 1H), 8.09 (d, *J* = 6.9 Hz, 1H), 7.69 (d, *J* = 7.6 Hz, 1H), 7.50 (t, *J* = 7.9 Hz, 1H), 5.00 (q, *J* = 6.5 Hz, 1H), 2.41 (s, 1H), 1.51 (d, *J* = 6.5 Hz, 3H).

^{13}C NMR (101 MHz, $CDCl_3$) δ 148.2, 147.8, 131.6, 129.4, 122.3, 120.3, 69.3, 25.4.

The enantiomeric excess was determined by HPLC on Chiralcel OD-H column, *n*-hexane : isopropanol = 99 : 1, flow rate = 1.0 mL/min, UV detection at λ = 210 nm, t_R = 23.8 min (major), t_R = 25.5 min (minor).

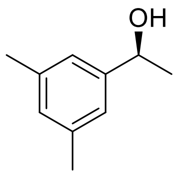

(*S*)-1-(3,5-dimethylphenyl)ethan-1-ol (**2q**)[7]

Colorless oil, 99% isolated yield, 92% *ee*, $[\alpha]_D^{25}$ = -40.0 (c 0.5, $CHCl_3$).

^{1}H NMR (400 MHz, $CDCl_3$) δ 7.00 (s, 1H), 6.94 (s, 2H), 4.81 (q, *J* = 6.5 Hz, 1H), 2.35 (s, 6H), 1.49 (d, *J* = 6.5 Hz, 3H).

^{13}C NMR (101 MHz, $CDCl_3$) δ 145.8, 137.9, 128.9, 123.1, 70.2, 25.0, 21.2.

The enantiomeric excess was determined by HPLC on Chiralcel OD-H column, *n*-hexane : isopropanol = 98 : 2, flow rate = 1.0 mL/min, UV detection at λ = 210 nm, t_R

= 11.4 min (minor), t_R = 12.3 min (major).

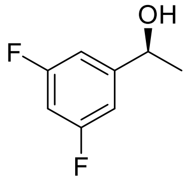

(*S*)-1-(3,5-difluorophenyl)ethan-1-ol (**2r**)[8]

Colorless oil, 80% isolated yield, 88% *ee*, $[\alpha]_D^{25}$ = -27.2 (c 0.5, $CHCl_3$).

^{1}H NMR (400 MHz, $CDCl_3$) δ 6.86 (d, *J* = 6.4 Hz, 1H), 6.70 – 6.65 (m, 1H), 4.83 (q, *J* = 6.5 Hz, 1H), 2.34 (s, 1H), 1.44 (d, *J* = 6.5 Hz, 3H).

^{13}C NMR (101 MHz, $CDCl_3$) δ 163.0 (dd, *J* = 248.5, 12.5 Hz), 149.9 (t, *J* = 8.1 Hz), 108.10 (d, *J* = 11.7 Hz), 108.10 (d, *J* = 25.4 Hz)., 102.5 (t, *J* = 25.4 Hz), 69.4, 25.1.

^{19}F NMR (376 MHz, $CDCl_3$) δ -109.66 (t, *J* = 7.8 Hz).

The enantiomeric excess was determined by HPLC on Chiralcel AD-H column, *n*-hexane : isopropanol = 98 : 2, flow rate = 1.0 mL/min, UV detection at λ = 210 nm, t_R = 9.1 min (minor), t_R = 10.7 min (major).

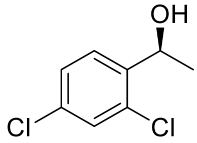

(*S*)-1-(2,4-dichlorophenyl)ethan-1-ol (**2s**)[7]

Colorless oil, 91% isolated yield, 91% *ee*, $[\alpha]_D^{25}$ = -54.0 (c 0.5, $CHCl_3$).

^{1}H NMR (400 MHz, $CDCl_3$) δ 7.46 (d, *J* = 8.5 Hz, 1H), 7.29 (d, *J* = 2.2 Hz, 1H), 7.22 (dd, *J* = 8.4, 2.1 Hz, 1H), 5.16 (q, *J* = 6.4 Hz, 1H), 2.55 (s, 1H), 1.40 (d, *J* = 6.4 Hz, 3H).

^{13}C NMR (101 MHz, $CDCl_3$) δ 141.6, 133.3, 132.0, 129.0, 127.4, 127.3, 66.4, 23.5.

The enantiomeric excess was determined by HPLC on Chiralcel OD-H column, *n*-hexane : isopropanol = 99 : 1, flow rate = 0.8 mL/min, UV detection at λ = 210 nm, t_R = 11.4 min (minor), t_R = 12.3 min (major).

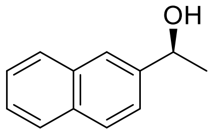

(*S*)-1-(naphthalen-2-yl)ethan-1-ol (**2t**)[2]

White solid, 96% isolated yield, 85% *ee*, $[\alpha]_D^{25}$ = -38.6 (c 0.5, $CHCl_3$).

^{1}H NMR (400 MHz, $CDCl_3$) δ 8.04 – 7.81 (m, 3H), 7.79 (s, 1H), 7.50 – 7.48 (m, 3H), 5.03 (q, *J* = 6.4 Hz, 1H), 2.30 (s, 1H), 1.57 (d, *J* = 6.5 Hz, 3H).

^{13}C NMR (101 MHz, $CDCl_3$) δ 143.1, 133.2, 132.8, 128.2, 127.9, 127.6, 126.1, 125.7, 123.8, 123.7, 70.4, 25.0.

The enantiomeric excess was determined by HPLC on Chiralcel IA-3 column, *n*-hexane : isopropanol = 97 : 3, flow rate = 1.0 mL/min, UV detection at λ = 254 nm, t_R = 18.9 min (major), t_R = 19.6 min (minor).

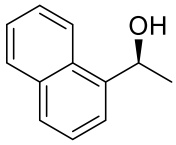

(*S*)-1-(naphthalen-1-yl)ethan-1-ol (**2u**)[2]

Colorless oil, 99% isolated yield, 80% *ee*, $[\alpha]_D^{25}$ = -54.6 (c 0.5, $CHCl_3$).

^{1}H NMR (400 MHz, $CDCl_3$) δ 8.09 (d, *J* = 7.1 Hz, 1H), 7.93 – 7.86 (m, 1H), 7.78 (d, *J* = 8.2 Hz, 1H), 7.67 (d, *J* = 7.2 Hz, 1H), 7.56 – 7.45 (m, 3H), 5.63 (q, *J* = 6.5 Hz, 1H), 2.28 (s, 1H), 1.65 (d, *J* = 6.5 Hz, 3H).

^{13}C NMR (101 MHz, $CDCl_3$) δ 141.3, 133.7, 130.2, 128.8, 127.8, 125.9, 125.4, 121.9, 66.9, 24.3.

The enantiomeric excess was determined by HPLC on Chiralcel OD-H column, *n*-hexane : isopropanol = 90 : 10, flow rate = 1.0 mL/min, UV detection at λ = 210 nm, t_R = 9.9 min (major), t_R = 17.1 min (minor).

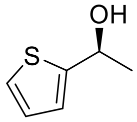

(S)-1-(thiophen-2-yl)ethan-1-ol (**2v**)[2]

Colorless oil, 92% isolated yield, 92% *ee*, $[\alpha]_D^{25}$ = -22.8 (c 0.5, $CHCl_3$).

^{1}H NMR (400 MHz, $CDCl_3$) δ 7.26 – 7.18 (m, 1H), 6.96 (d, J = 3.8 Hz, 2H), 5.09 (q, J = 6.4 Hz, 1H), 2.61 (s, 1H), 1.58 (d, J = 6.5 Hz, 3H).

^{13}C NMR (101 MHz, $CDCl_3$) δ 149.8, 126.5, 124.3, 123.1, 66.0, 25.1.

The enantiomeric excess was determined by HPLC on Chiralcel IC column, *n*-hexane : isopropanol = 95 : 5, flow rate = 1.0 mL/min, UV detection at λ = 210 nm, t_R = 7.5 min (major), t_R = 8.2 min (minor).

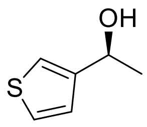

(S)-1-(thiophen-3-yl)ethan-1-ol (**2w**)[2]

Colorless oil, 93% isolated yield, 85% *ee*, $[\alpha]_D^{25}$ = -27.0 (c 0.5, $CHCl_3$).

^{1}H NMR (400 MHz, $CDCl_3$) δ 7.30 – 7.26 (m, 1H), 7.18 (s, 1H), 7.09 (d, J = 5.1 Hz, 1H), 4.95 (q, J = 6.4 Hz, 1H), 2.11 (s, 1H), 1.51 (d, J = 6.5 Hz, 1H).

^{13}C NMR (101 MHz, $CDCl_3$) δ 147.2, 126.1, 125.6, 120.1, 66.4, 24.4.

The enantiomeric excess was determined by HPLC on Chiralcel OJ-H column, *n*-hexane : isopropanol = 98 : 2, flow rate = 1.0 mL/min, UV detection at λ = 210 nm, t_R = 11.6 min (major), t_R = 13.7 min (minor).

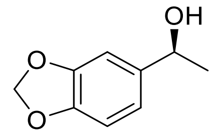

(S)-1-(benzo[*d*][1,3]dioxol-5-yl)ethan-1-ol (**2x**)[2]

Colorless oil, 99% isolated yield, 91% *ee*, $[\alpha]_D^{25}$ = -49.8 (c 0.5, $CHCl_3$).

^{1}H NMR (400 MHz, $CDCl_3$) δ 6.83 – 6.82 (m, 1H), 6.79 – 6.65 (m, 2H), 5.89 (s, 2H), 4.73 (q, J = 6.3 Hz, 1H), 2.76 (s, 1H), 1.39 (d, J = 6.4 Hz, 3H).

^{13}C NMR (101 MHz, $CDCl_3$) δ 147.5, 146.5, 139.9, 118.5, 107.9, 105.9, 100.8, 69.9, 25.0.

The enantiomeric excess was determined by HPLC on Chiralcel OD-H column, *n*-hexane : isopropanol = 95 : 5, flow rate = 1.0 mL/min, UV detection at λ = 210 nm, t_R = 17.1 min (minor), t_R = 19.3 min (major).

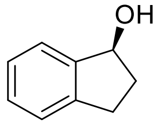

(*S*)-2,3-dihydro-1*H*-inden-1-ol (**2y**)[2]

White solid, 90% isolated yield, 86% *ee*, $[\alpha]_D^{25}$ = 10.0 (c 0.5, $CHCl_3$).

^{1}H NMR (400 MHz, $CDCl_3$) δ 7.42 – 7.41 (m, 1H), 7.25 – 7.23 (m, 3H), 5.24 (t, *J* = 6.09, 1H), 3.05 (m, 1H), 2.81 (m, 1H), 2.48 (m, 1H), 1.94 (m, 2H).

^{13}C NMR (101 MHz, $CDCl_3$) δ 144.9, 143.3, 128.3, 126.7, 124.9, 124.2, 76.4, 35.9, 29.8.

The enantiomeric excess was determined by HPLC on Chiralcel OD-H column, *n*-hexane : isopropanol = 95 : 5, flow rate = 1.0 mL/min, UV detection at λ = 210 nm, t_R = 9.3 min (major), t_R = 10.3 min (minor).

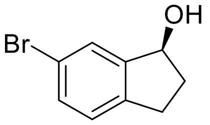

(*S*)-6-bromo-2,3-dihydro-1*H*-inden-1-ol (**2z**)[9]

White solid, 99% isolated yield, 92% *ee*, $[\alpha]_D^{25}$ = 48.8 (c 0.5, $CHCl_3$).

^{1}H NMR (400 MHz, $CDCl_3$) δ 7.51 (s, 1H), 7.36 – 7.34 (m, 1H), 7.11 – 7.09 (m, 1H), 5.18 (s, 1H), 2.98 – 2.94 (m, 1H), 2.77 – 2.70 (m, 1H), 2.53 – 2.44 (m, 1H), 2.31 (s, 1H), 1.92 (dt, *J* = 13.9, 6.9 Hz, 1H).

^{13}C NMR (101 MHz, $CDCl_3$) δ 147.2, 142.1, 131.2, 127.4, 126.4, 120.2, 75.9, 36.1, 29.3.

The enantiomeric excess was determined by HPLC on Chiralcel OD-H column, *n*-hexane : isopropanol = 99 : 1, flow rate = 1.0 mL/min, UV detection at λ = 210 nm, t_R = 13.4 min (major), t_R = 14.6 min (minor).

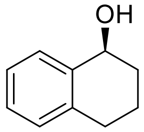

(S)-1,2,3,4-tetrahydronaphthalen-1-ol (**2aa**)[2]

White solid, 99% isolated yield, 97% *ee*, $[\alpha]_D^{25}$ = 31.2 (c 0.5, $CHCl_3$).

^{1}H NMR (400 MHz, $CDCl_3$) δ 7.49 – 7.38 (m, 1H), 7.24 – 7.18 (m, 2H), 7.14 – 7.08 (m, 1H), 4.79 (t, J = 4.7 Hz, 1H), 2.87 – 2.80 (m, 1H), 2.78 – 2.64 (m, 1H), 2.05 – 1.75 (m, 5H).

^{13}C NMR (101 MHz, $CDCl_3$) δ 138.7, 137.0, 128.9, 128.6, 127.5, 126.1, 68.0, 32.2, 29.2, 18.7.

The enantiomeric excess was determined by HPLC on Chiralcel IB column, *n*-hexane : isopropanol = 98 : 2, flow rate = 0.8 mL/min, UV detection at λ = 210 nm, t_R = 15.3 min (major), t_R = 16.2 min (minor).

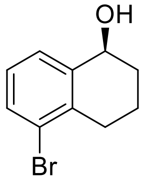

(S)-5-bromo-1,2,3,4-tetrahydronaphthalen-1-ol (**2ab**)[10]

White solid, 95% isolated yield, 90% *ee*, $[\alpha]_D^{25}$ = -3.2 (c 0.5, $CHCl_3$).

^{1}H NMR (400 MHz, $CDCl_3$) δ 7.47 (d, J = 7.9 Hz, 1H), 7.39 (d, J = 7.7 Hz, 1H), 7.07 (t, J = 7.8 Hz, 1H), 4.73 (t, J = 4.8 Hz, 1H), 2.82 (m, 1H), 2.65 (m, 1H), 2.10 (s, 1H), 1.96 (m, 2H), 1.82 (m, 2H).

^{13}C NMR (101 MHz, $CDCl_3$) δ 141.2, 136.5, 131.7, 127.8, 127.3, 125.3, 68.2, 31.5, 30.0, 18.4.

The enantiomeric excess was determined by HPLC on Chiralcel OD-H column, *n*-hexane : isopropanol = 90 : 10, flow rate = 1.0 mL/min, UV detection at λ = 210 nm, t_R = 9.5 min (major), t_R = 11.2 min (minor).

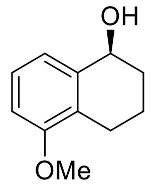

(*S*)-5-methoxy-1,2,3,4-tetrahydronaphthalen-1-ol (**2ac**)[10]

White solid, 94% isolated yield, 96% *ee*, $[\alpha]_D^{25}$ = 15.4 (c 0.5, $CHCl_3$).

^{1}H NMR (400 MHz, $CDCl_3$) δ 7.20 (t, J = 7.9 Hz, 1H), 7.06 (d, J = 7.7 Hz, 1H), 6.76 (d, J = 8.1 Hz, 1H), 4.77 (t, J = 4.4 Hz, 1H), 3.83 (s, 3H), 2.79 – 2.72 (m, 1H), 2.61 – 2.46 (m, 1H), 2.09 – 1.83 (m, 3H), 1.82 – 1.67 (m, 2H).

^{13}C NMR (101 MHz, $CDCl_3$) δ 157.0, 140.0, 126.5, 126.1, 120.5, 108.6, 68.1, 55.3, 31.7, 23.0, 18.0.

The enantiomeric excess was determined by HPLC on Chiralcel AD-H column, *n*-hexane : isopropanol = 95 : 5, flow rate = 1.0 mL/min, UV detection at λ = 210 nm, t_R = 12.9 min (minor), t_R = 17.0 min (major).

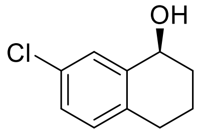

(*S*)-7-chloro-1,2,3,4-tetrahydronaphthalen-1-ol (**2ad**)[10]

White solid, 99% isolated yield, 96% *ee*, $[\alpha]_D^{25}$ = 68.8 (c 0.5, $CHCl_3$).

^{1}H NMR (400 MHz, $CDCl_3$) δ 7.43 (d, J = 2.3 Hz, 1H), 7.16 – 7.13 (m, 1H), 7.02 (d, J = 8.1 Hz, 1H), 4.73 – 4.70 (m, 1H), 2.84 – 2.51 (m, 2H), 2.07 – 1.71 (m, 5H).

^{13}C NMR (101 MHz, $CDCl_3$) δ 140.5, 135.4, 131.6, 130.3, 128.3, 127.6, 67.9, 32.1, 28.6, 18.9.

The enantiomeric excess was determined by HPLC on Chiralcel OD-H column, *n*-hexane : isopropanol = 99 : 1, flow rate = 0.8 mL/min, UV detection at λ = 210 nm, t_R = 14.5 min (major), t_R = 15.7 min (minor).

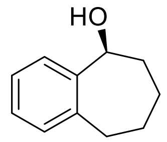

(S)-6,7,8,9-tetrahydro-5H-benzo[7]annulen-5-ol (**2ae**)[2]

White solid, 99% isolated yield, 94% *ee*, $[\alpha]_D^{25}$ = -29.4 (c 0.5, $CHCl_3$).

^{1}H NMR (400 MHz, $CDCl_3$) δ 7.44 (d, J = 7.4 Hz, 1H), 7.24 – 7.14 (m, 2H), 7.10 (d, J = 7.2 Hz, 1H), 4.93 (d, J = 8.0 Hz, 1H), 2.96 – 2.90 (m, 1H), 2.83 – 2.63 (m, 1H), 2.08 – 2.03 (m, 1H), 2.01 – 1.87 (m, 2H), 1.86 – 1.70 (m, 3H), 1.53 – 1.44 (m, 1H).

^{13}C NMR (101 MHz, $CDCl_3$) δ 144.2, 140.8, 129.5, 126.9, 126.1, 124.5, 74.0, 36.6, 35.7, 27.8, 27.6.

The enantiomeric excess was determined by HPLC on Chiralcel OD-H column, *n*-hexane : isopropanol = 99 : 1, flow rate = 0.8 mL/min, UV detection at λ = 210 nm, t_R = 15.4 min (minor), t_R = 16.8 min (major).

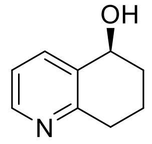

(S)-5,6,7,8-tetrahydroquinolin-5-ol (**2af**)[2]

White solid, 89% isolated yield, 82% *ee*, $[\alpha]_D^{25}$ = 54.2 (c 0.5, $CHCl_3$).

^{1}H NMR (400 MHz, $CDCl_3$) δ 8.28 – 8.27 (m, 1H), 7.76 (d, J = 7.6, 1H), 7.08 (dd, J = 7.8, 4.8 Hz, 1H), 4.76 (t, J = 4.6 Hz, 1H), 3.92 (s, 1H), 2.91 – 2.78 (m, 2H), 2.09 – 1.99 (m, 2H), 1.89 – 1.73 (m, 2H).

^{13}C NMR (101 MHz, $CDCl_3$) δ 156.9, 147.9, 136.5, 134.9, 121.4, 67.5, 32.1, 31.9, 18.7.

The enantiomeric excess was determined by HPLC on Chiralcel AD-H column, *n*-hexane : isopropanol = 93 : 7, flow rate = 1.0 mL/min, UV detection at λ = 210 nm, t_R = 11.6 min (minor), t_R = 13.2 min (major).

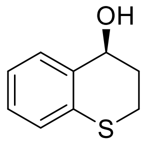

(S)-thiochroman-4-ol (**2ag**)[2]

White solid, 83% isolated yield, 95% *ee*, $[\alpha]_D^{25}$ = -2.4 (c 0.5, $CHCl_3$).

^{1}H NMR (400 MHz, $CDCl_3$) δ 7.30 (d, J = 7.6 Hz, 1H), 7.17 – 7.11 (m, 2H), 7.08 – 7.04 m, 1H), 4.76 (s, 1H), 3.33 – 3.26 (m, 1H), 2.87 – 2.81 (m, 1H), 2.39 – 2.24 (m, 1H), 2.17 – 1.93 (m, 2H).

^{13}C NMR (101 MHz, $CDCl_3$) δ 134.5, 133.1, 130.3, 128.4, 66.4, 29.9, 21.4.

The enantiomeric excess was determined by HPLC on Chiralcel OD-H column, *n*-hexane : isopropanol = 95 : 5, flow rate = 1.0 mL/min, UV detection at λ = 210 nm, t_R = 12.2 min (major), t_R = 15.5 min (minor).

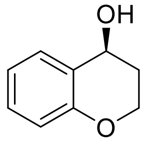

(S)-chroman-4-ol (**2ah**)[2]

White solid, 97% isolated yield, 96% *ee*, $[\alpha]_D^{25}$ = -67.2 (c 0.5, $CHCl_3$).

^{1}H NMR (400 MHz, $CDCl_3$) δ 7.31 (dd, J = 7.7, 1.7 Hz, 1H), 7.24 – 7.15 (m, 1H), 6.92 (t, J = 7.4 Hz, 1H), 6.84 (d, J = 8.3 Hz, 1H), 4.78 (t, J = 4.1 Hz, 1H), 4.33 – 4.11 (m, 2H), 2.22 – 2.07 (m, 1H), 2.05 – 1.97 (m, 1H), 1.97 (s, 1H).

^{13}C NMR (101 MHz, $CDCl_3$) δ 154.5, 129.7, 129.6, 124.3, 120.6, 117.0, 63.2, 61.9, 30.8.

The enantiomeric excess was determined by HPLC on Chiralcel OD-H column, *n*-hexane : isopropanol = 93 : 7, flow rate = 1.0 mL/min, UV detection at λ = 210 nm, t_R = 19.9 min (major), t_R = 24.4 min (minor).

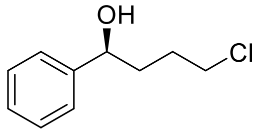

(*S*)-4-chloro-1-phenylbutan-1-ol (**2ai**)[11]

Colorless oil, 96% isolated yield, 96% *ee*, $[\alpha]_D^{25}$ = -34.2 (c 0.5, $CHCl_3$).

^{1}H NMR (400 MHz, $CDCl_3$) δ 7.39 – 7.24 (m, 5H), 4.72 –4.68 (m, 1H), 3.59 – 3.52 (m, 2H), 2.03 – 1.85 (m, 4H), 1.85 – 1.73 (m, 1H).

^{13}C NMR (101 MHz, $CDCl_3$) δ 144.3, 128.5, 127.7, 125.8, 73.9, 45.0, 36.1, 28.9.

The enantiomeric excess was determined by HPLC on Chiralcel OD-H column, *n*-hexane : isopropanol = 95 : 5, flow rate = 1.0 mL/min, UV detection at λ = 210 nm, t_R = 13.7 min (major), t_R = 15.4 min (minor).

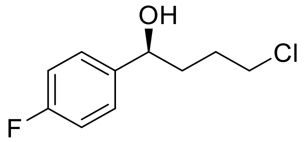

(*S*)-4-chloro-1-(4-fluorophenyl)butan-1-ol (**2aj**)[11]

Colorless oil, 96% isolated yield, 88% *ee*, $[\alpha]_D^{25}$ = -35.8 (c 0.5, $CHCl_3$).

^{1}H NMR (400 MHz, $CDCl_3$) δ 7.23 – 7.21 (m, 2H), 6.99 – 6.95 (m, 2H), 4.56 (t, *J* = 6.1 Hz, 1H), 3.47 (t, *J* = 6.1 Hz, 2H), 3.00 (s, 1H), 1.87 – 1.59 (m, 4H).

^{13}C NMR (101 MHz, $CDCl_3$) δ 162.0 (d, *J* = 245.4 Hz), 139.9 (d, *J* = 3.1 Hz), 127.3 (d, *J* = 8.1 Hz), 115.1 (d, *J* = 21.4 Hz), 72.9, 44.7, 36.0, 28.6.

^{19}F NMR (376 MHz, $CDCl_3$) δ -114.79 – -114.89 (m).

The enantiomeric excess was determined by HPLC on Chiralcel OD-H column, *n*-hexane : isopropanol = 95 : 5, flow rate = 1.0 mL/min, UV detection at λ = 210 nm, t_R = 10.4 min (major), t_R = 11.5 min (minor).

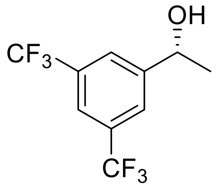

(*R*)-1-(3,5-bis(trifluoromethyl)phenyl)ethan-1-ol (**2ak**)[2]

White solid, 94% isolated yield, 92 % *ee*, $[\alpha]_D^{25}$ = 22.8 (c 0.5, $CHCl_3$).

^{1}H NMR (400 MHz, $CDCl_3$) δ 7.84 (s, 2H), 7.78 (s, 1H), 5.04 (q, *J* = 6.5 Hz, 1H), 2.12

(s, 1H), 1.54 (dd, J = 6.5, 0.9 Hz, 3H).

^{13}C NMR (101 MHz, CDCl$_3$) δ 148.2, 131.7 (q, J = 33.3 Hz), 125.6 (q, J = 5.24 Hz), 123.3 (q, J = 272.6 Hz), 121.4 – 121.2 (m), 69.3, 25.4.

^{19}F NMR (376 MHz, CDCl$_3$) δ -62.90.

The enantiomeric excess was determined by HPLC on Chiralcel OD-3 column, *n*-hexane : isopropanol = 98 : 2, flow rate = 1.0 mL/min, UV detection at λ = 220 nm, t_R = 8.9 min (minor), t_R = 10.4 min (major).

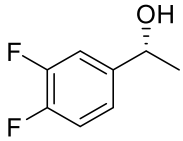

(R)-1-(3,4-difluorophenyl)ethan-1-ol (**2al**)[12]

Colorless oil, 93% isolated yield, 85% *ee*, $[\alpha]_D^{25}$ = 36.0 (c 0.5, CHCl$_3$).

^{1}H NMR (400 MHz, CDCl$_3$) δ 7.22 – 6.97 (m, 3H), 4.83 (q, J = 6.03 Hz, 1H), 2.28 – 2.04 (m, 1H), 1.52 – 1.37 (m, 3H).

^{13}C NMR (101 MHz, CDCl$_3$) δ 150.3 (dd, J = 247.97, 13.0 Hz), 149.5 (dd, J = 246.37, 13.2 Hz), 142.8 (t, J = 4.25 Hz), 121.2 (dd, J = 6.3, 3.6 Hz), 117.1 (d, J = 17.3 Hz), 114.3 (d, J = 17.6 Hz), 69.3, 25.3.

^{19}F NMR (376 MHz, CDCl$_3$) δ -136.06 – -139.05 (m), -139.96 – -140.29 (m).

The enantiomeric excess was determined by HPLC on Chiralcel ID column, *n*-hexane : isopropanol = 95 : 5, flow rate = 1.0 mL/min, UV detection at λ = 210 nm, t_R = 6.4 min (major), t_R = 7.0 min (minor).

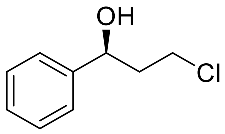

(S)-3-chloro-1-phenylpropan-1-ol (**2am**)[13]

White solid, 99% isolated yield, 82% *ee*, $[\alpha]_D^{25}$ = -21.6 (c 0.5, CHCl$_3$).

^{1}H NMR (400 MHz, CDCl$_3$) δ 7.45 – 7.20 (m, 5H), 5.00 – 4.84 (m, 1H), 3.77 – 3.71 (m, 1H), 3.59 – 3.53 (m, 1H), 2.28 – 2.19 (m, 1H), 2.15 – 2.02 (m, 2H).

^{13}C NMR (101 MHz, CDCl$_3$) *δ* 143.7, 128.6, 127.9, 125.7, 71.3, 41.7, 41.4.

The enantiomeric excess was determined by HPLC on Chiralcel OD-H column, *n*-hexane : isopropanol = 95 : 5, flow rate = 1.0 mL/min, UV detection at λ = 210 nm, t_R = 11.4 min (major), t_R = 14.1 min (minor).

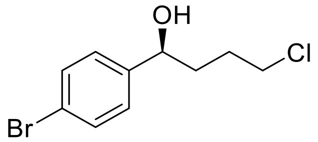

(*S*)-1-(4-bromophenyl)-4-chlorobutan-1-ol (**2an**)[11]

Colorless oil, 97% isolated yield, 84% *ee*, $[\alpha]_D^{25}$ = -27.4 (c 0.5, CHCl$_3$).

^{1}H NMR (400 MHz, CDCl$_3$) *δ* 7.46 (d, *J* = 8.4 Hz, 2H), 7.20 (d, *J* = 8.2 Hz, 2H), 4.65 (t, *J* = 6.1 Hz, 1H), 3.56 – 3.52 (m, 2H), 2.14 (s, 1H), 1.96 – 1.68 (m, 4H).

^{13}C NMR (101 MHz, CDCl$_3$) *δ* 143.2, 131.6, 127.5, 121.4, 73.1, 44.8, 36.0, 28.6.

The enantiomeric excess was determined by HPLC on Chiralcel OD-H column, *n*-hexane : isopropanol = 95 : 5, flow rate = 1.0 mL/min, UV detection at λ = 210 nm, t_R = 13.2 min (major), t_R = 15.0 min (minor).

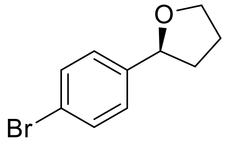

(*S*)-2-(4-bromophenyl)tetrahydrofuran (**3an**)[11]

Colorless oil, 80% isolated yield, 82% *ee*, $[\alpha]_D^{25}$ = -32.0 (c 0.5, CHCl$_3$).

^{1}H NMR (400 MHz, CDCl$_3$) *δδ* 7.47 – 7.38 (m, 2H), 7.21 (d, *J* = 8.3 Hz, 2H), 4.84 (t, *J* = 7.2 Hz, 1H), 4.07 (m, 1H), 3.92 (m, 1H), 2.31 (m, 1H), 1.99 (m, 2H), 1.73 (m, 1H).

^{13}C NMR (101 MHz, CDCl$_3$) *δ* 142.5, 131.2, 127.3, 120.7, 79.9, 68.6, 34.6, 25.9.

The enantiomeric excess was determined by HPLC on Chiralcel OD-H column, *n*-hexane : isopropanol = 95 : 5, flow rate = 1.0 mL/min, UV detection at λ = 210 nm, t_R = 6.5 min (minor), t_R = 7.0 min (major).

2.4 Kinetic studies

Geneal ReactIR Experimental Details for kinetic reaction profile measurements:

For the ReactIR kinetic experiments, the reaction spectra were recorded using a Mettler-Toledo ReactIR™ 45m. Data manipulation was carried out using the iC IR software, version 4.1. An overlayed IR spectrum of the reactant (**1b**) and an inspection of the spectra indicated that the IR absorbance at wavenumber of 1688 cm^{-1} can be used to track the variation of concentration of the substrate **1b** as a function of reaction courses.

Typical procedure for the reaction profile measurement using ReactIR:

The IR probe of the ReactIR was inserted through an adapter into the middle neck of a three necked reaction vessel which was fitted with a magnetic stirring bar. The left neck was capped by an argon line, and the right neck was capped with a rubber plug. Following evacuation under vacuum and flushing with argon for three times, the $Zn(OAc)_2$/(*S, S, S*)-**L1** catalyst solution was added [$Zn(OAc)_2$ (1.1 mg, 0.006 mmol, 0.3 mol%), (*S, S, S*)-**L1** (1.4 mg, 0.002 mmol, 0.1 mol%) and dry THF (5 mL) were added in a Schlenk tube and were pre-stirred until completely dissolved] and the data collection was started immediately, followed by sequential addition of the HBpin (0.58 mL, 4.0 mmol, 2.0 equiv) and **1b** (0.24 mL, 2.0 mmol).

2.4.1 Reaction order studies.

1) The order in Zn_3**L1** was determined by obtaining the initial rate of the **1b** consumption at differing amount of Zn_3**L1** following the typical procedure for the reaction profile measurement using ReactIR.

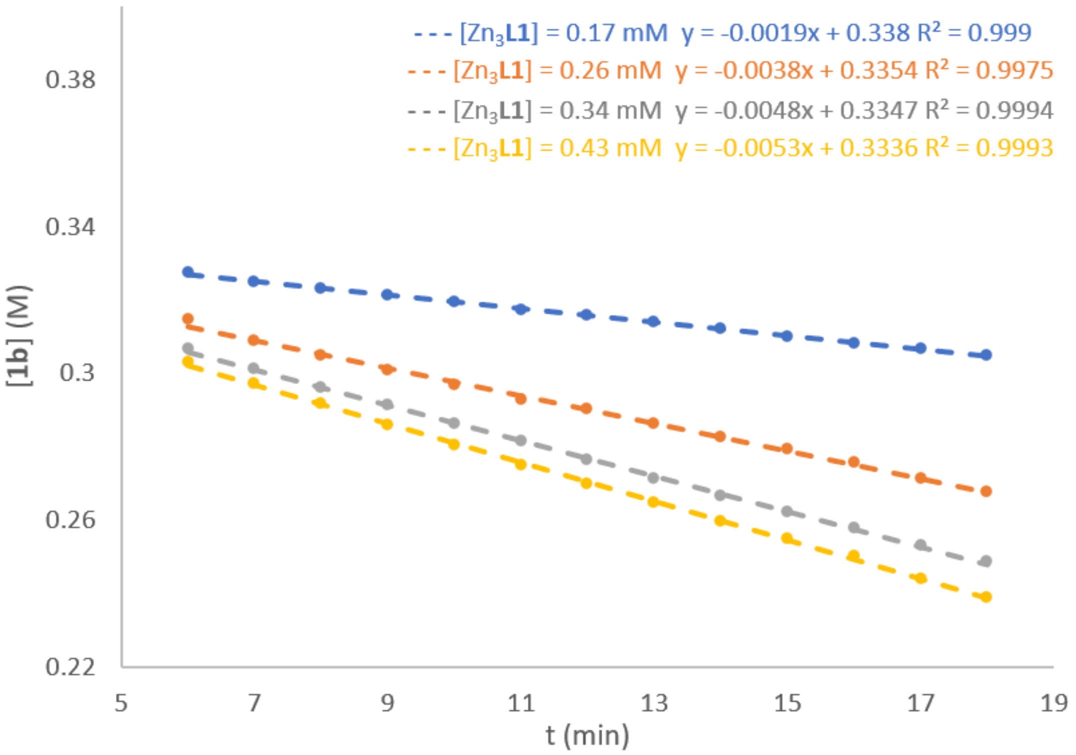

Figure S1. Initial rates of **1b** consumption with different concentration of Zn_3**L1**.

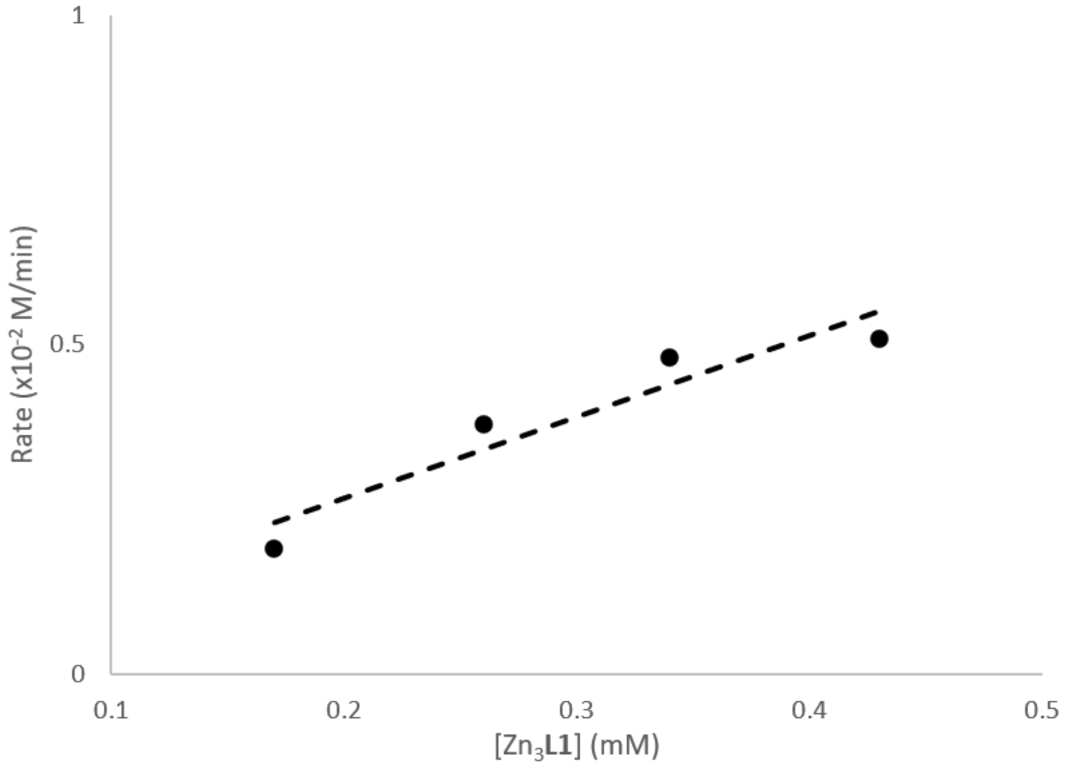

Figure S2. Initial rates of **1b** consumption with different concentration of Zn_3**L1**.

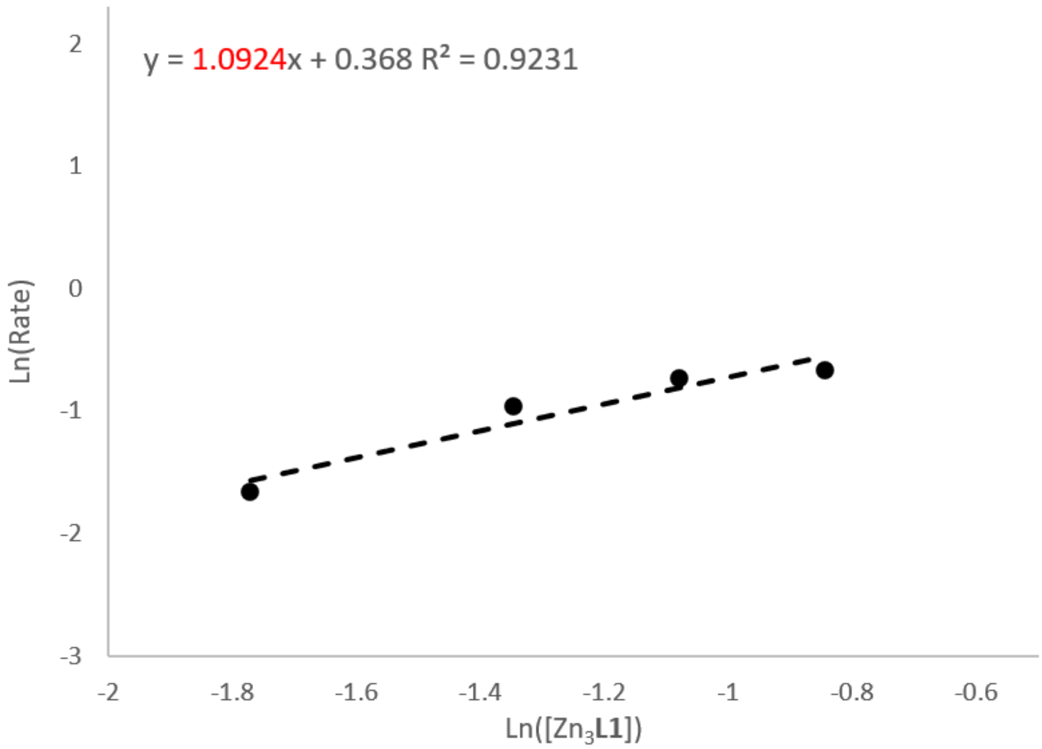

Figure S3. Plot of initial rates with varying Zn_3**L1**.

2) The order in **1b** was determined by obtaining the initial rate of the **1b** consumption at differing amount of **1b** following the typical procedure for the reaction profile measurement using ReactIR.

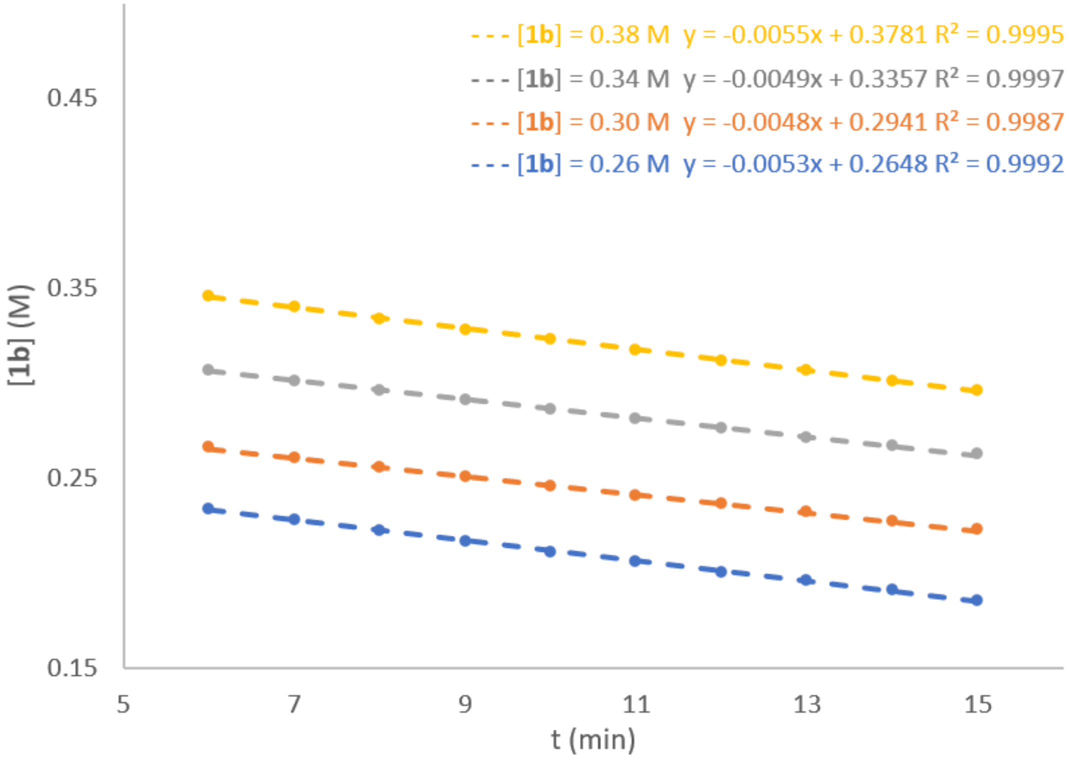

Figure S4. Initial rates of **1b** consumption with different concentration of **1b**.

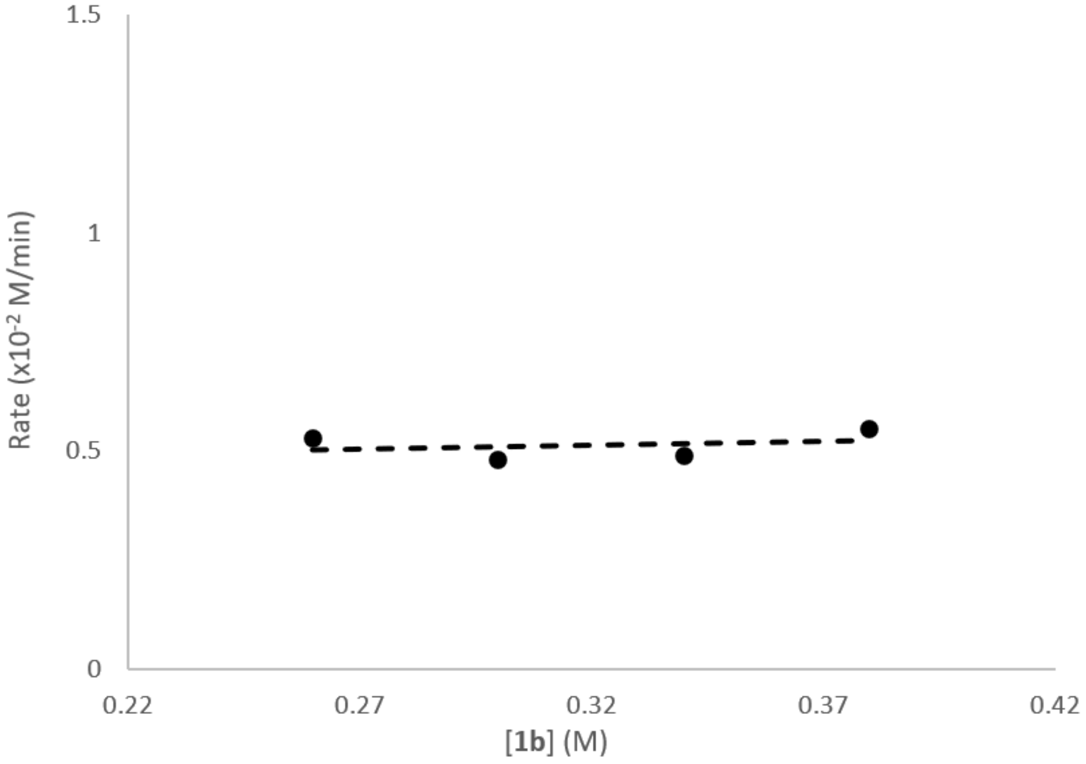

Figure S5. Initial rates of **1b** consumption with different concentration of **1b**.

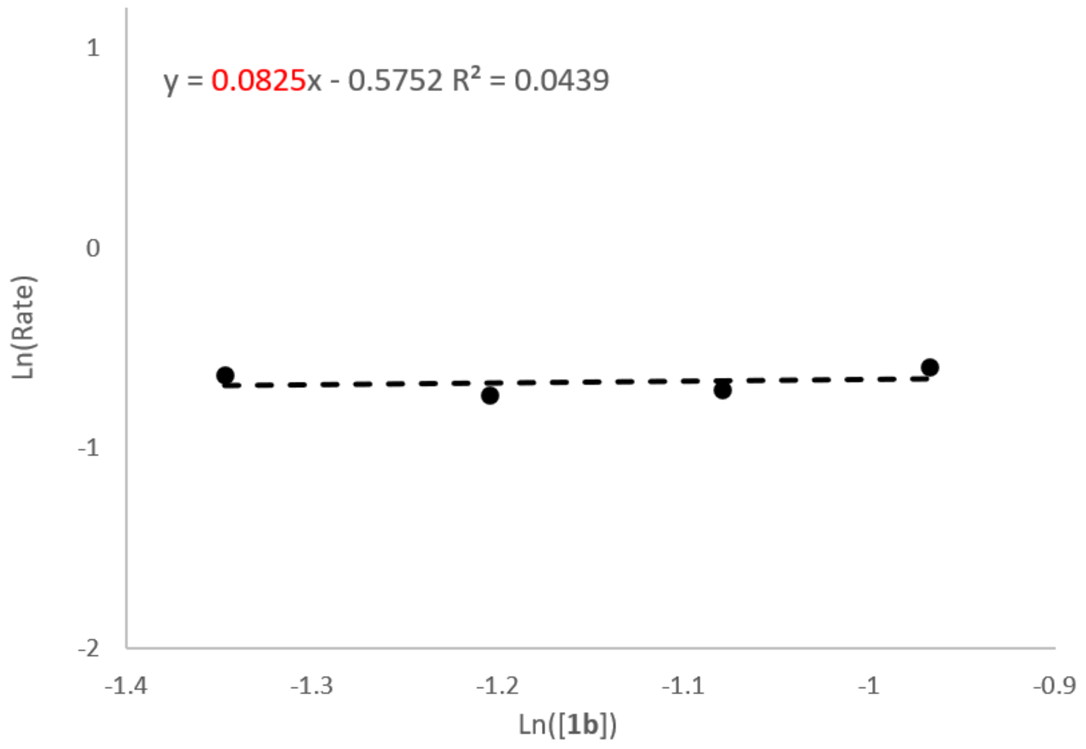

Figure S6. Plot of initial rates with varying **1b**.

3) The order in HBpin was determined by obtaining the initial rate of the **1b**

consumption at differing amount of HBpin following the typical procedure for the reaction profile measurement using ReactIR.

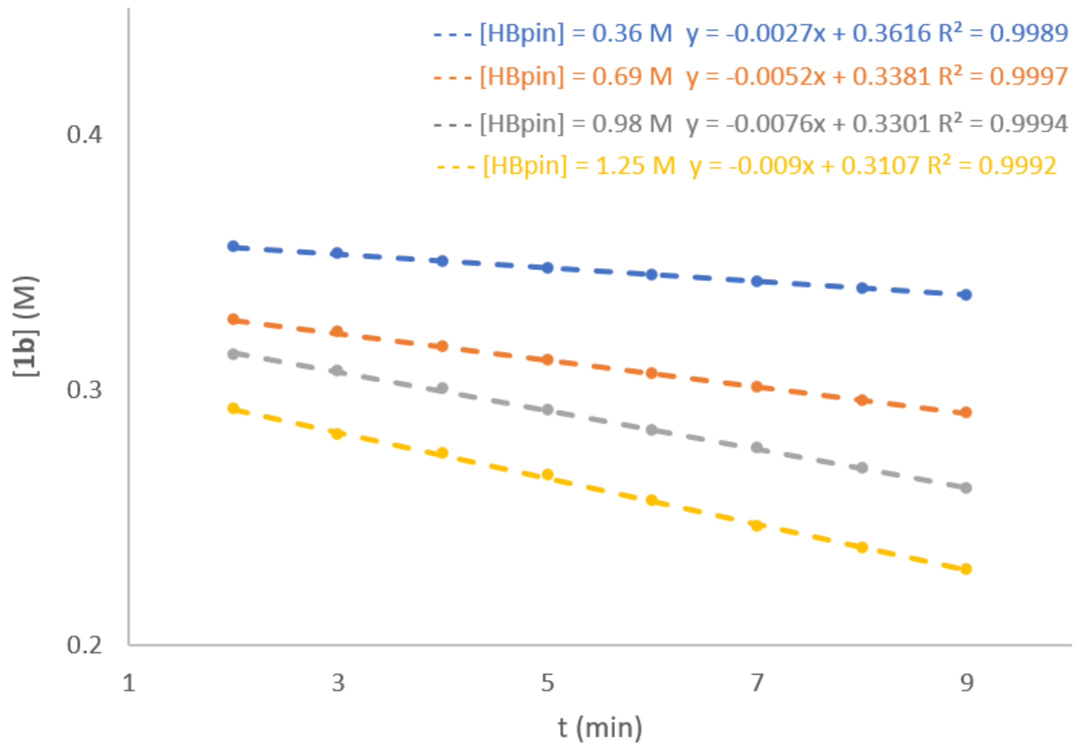

Figure S7. Initial rates of **1b** consumption with different concentration of HBpin.

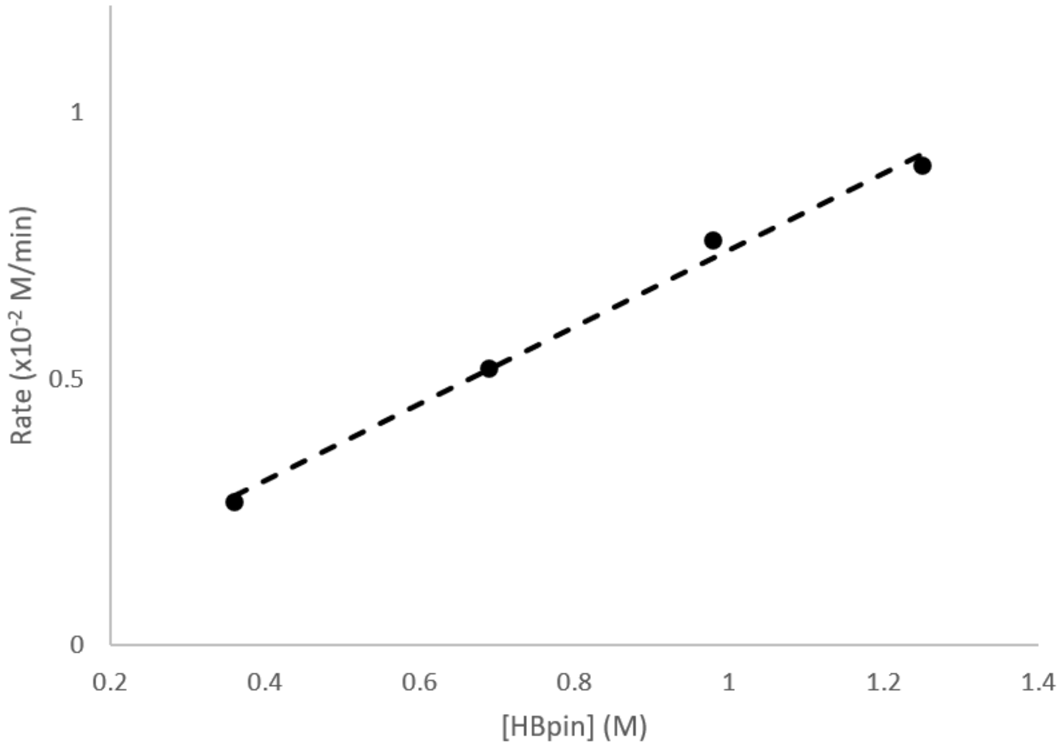

Figure S8. Initial rates of **1b** consumption with different concentration of HBpin.

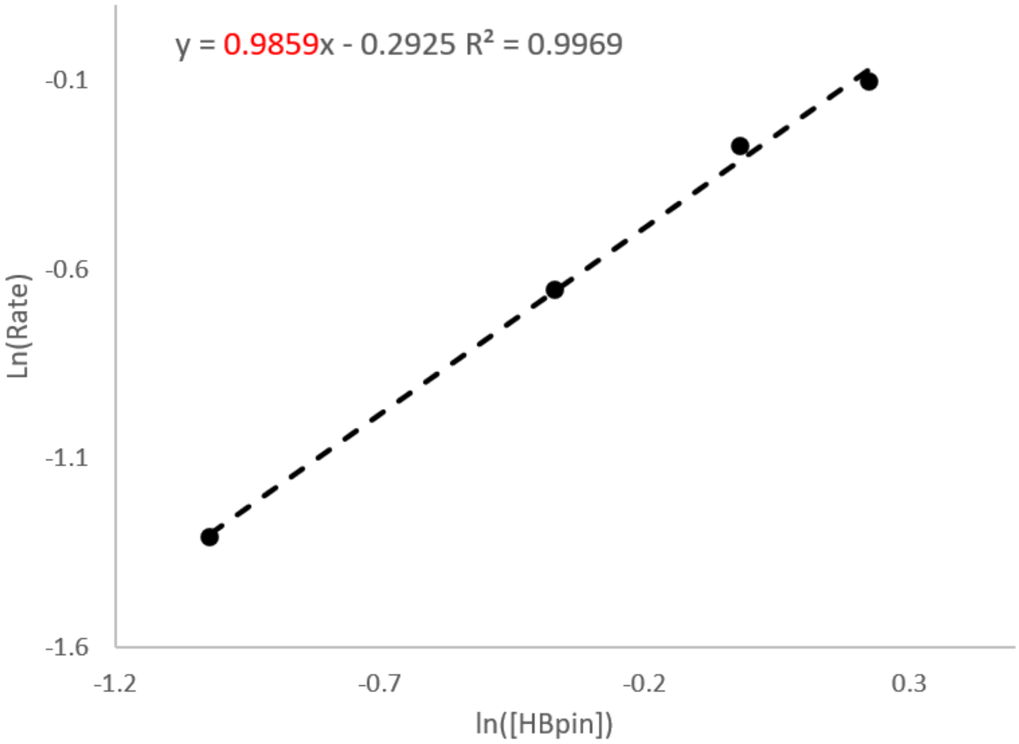

Figure S9. Plot of initial rates with varying HBpin.

2.4.2 Hammett plot

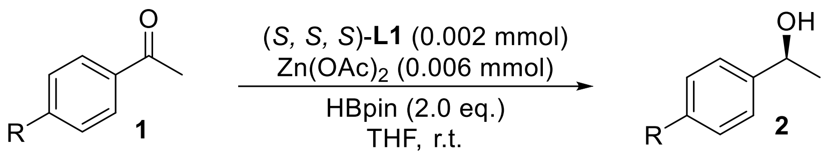

The Hammett plot was determined by obtaining the initial rate of the consumption of **1** with different ketones **1** following the typical procedure for the reaction profile measurement using ReactIR.

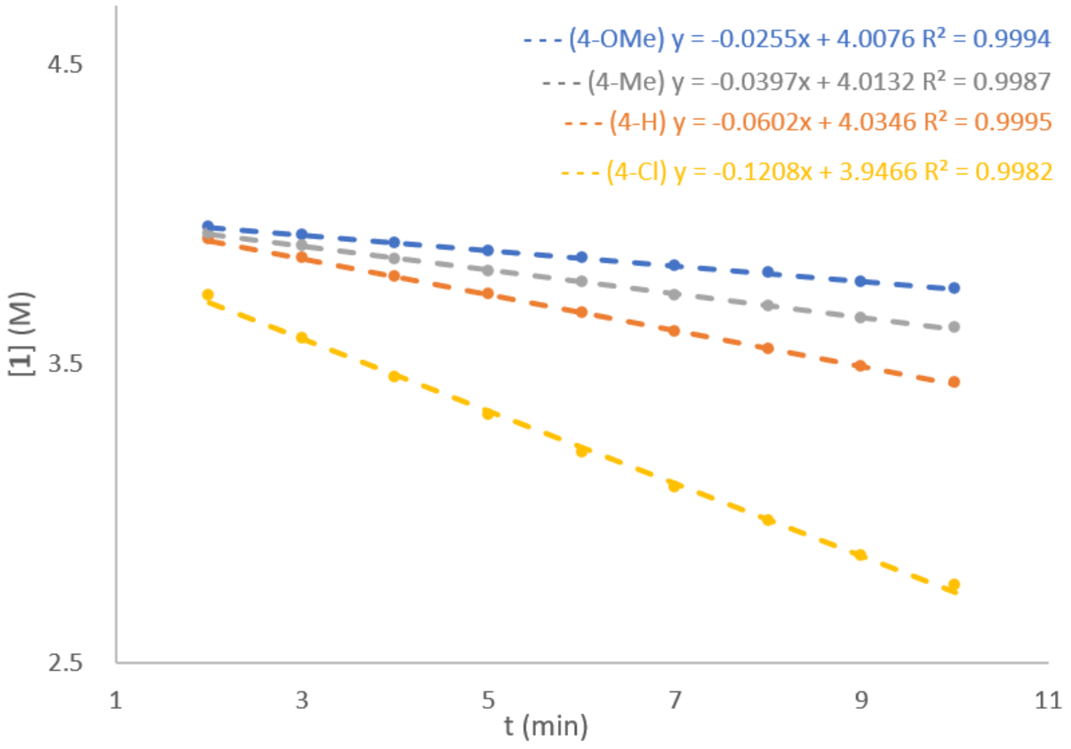

Figure S10. Plot of initial rates with varying **1**.

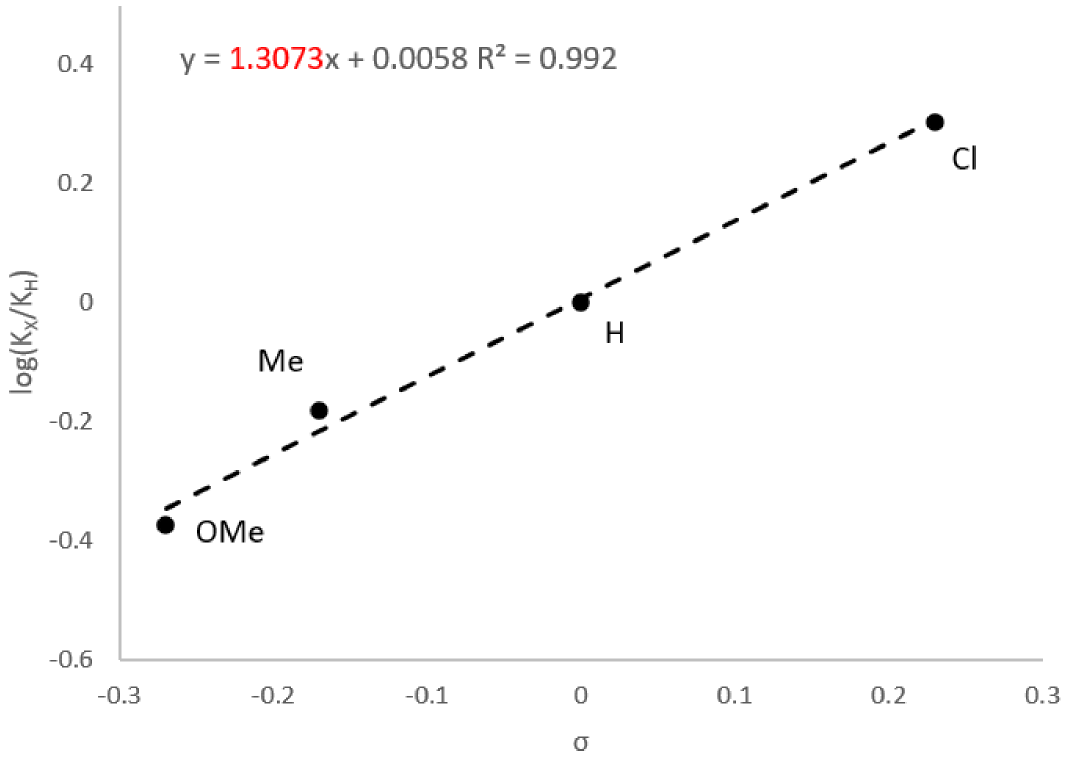

Figure S11. Hammett plot of **1**.

2.4.3 Nonlinear effect

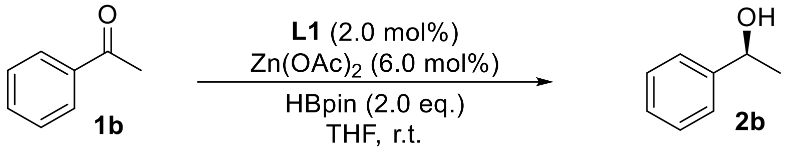

The specified *ee* values of **L1** (preferentially in *S, S, S* configuration) were achieved by premixing a certain amount of enantiopure (*S, S, S*)-**L1** with the (*R, R, R*)-**L1**. To a Schlenk tube equipped with magnetic stirring bar were added $Zn(OAc)_2$ (11 mg, 0.06 mmol), **L1** with different *ee* (13.8 mg, 0.02 mmol) and THF in glove box filled with N_2. Then the substrate **1b** (0.12 mL, 1.0 mmol) and HBpin (0.29 mL, 2.0 equiv) was added to the solution and the reaction was stirred at r.t. for 12 h. The *ee* value of the product was determined by HPLC analysis using a Chiralcel column. Experimental details are summarized in Table S5.

Table S5. Nonlinear relationship between the *ee* values of **L1** and the *ee* values of **2b** in THF[a].

Entry	*ee* of **L1** (%)	*ee* of **2b** (%)[b]
1	0	0
2	20	58
3	40	76
4	60	79
5	80	82
6	100	90

[a]Reaction conditions: **1b** (1.0 mmol), HBpin (2.0 equiv), **L1** (0.02 mmol), $Zn(OAc)_2$ (0.06 mmol), THF (5.0 mL), r.t., 12 h. [b]The *ee* values of **2b** were determined by chiral HPLC.

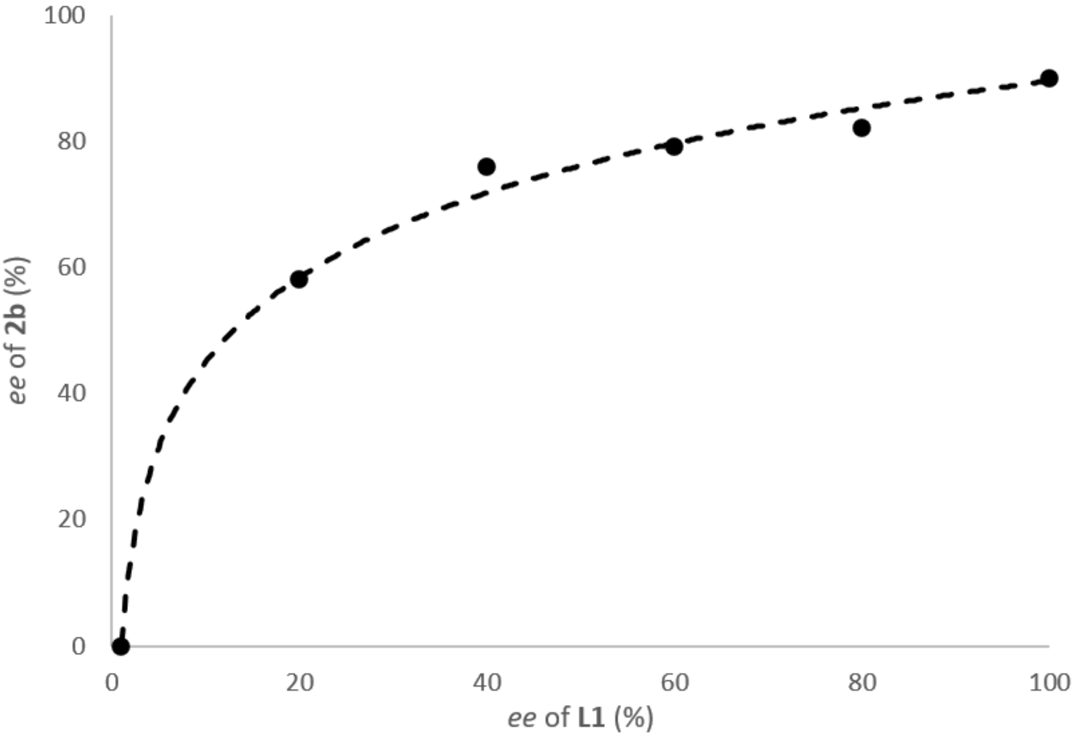

Figure S12. Positive NLE plot between **L1** and **2b** in THF.

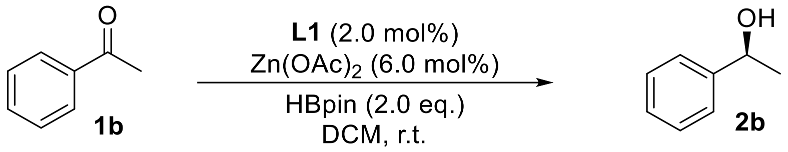

The specified *ee* values of **L1** (preferentially in *S, S, S* configuration) were achieved by premixing a certain amount of enantiopure (*S, S, S*)-**L1** with the (*R, R, R*)-**L1**. To a Schlenk tube equipped with magnetic stirring bar were added $Zn(OAc)_2$ (1.1 mg, 0.006 mmol), **L1** with different *ee* (1.4 mg, 0.002 mmol) and DCM in glove box filled with N_2. Then the substrate **1b** (12 μL, 0.1 mmol) and HBpin (29 μL, 2.0 equiv) was added to the solution and the reaction was stirred at r.t. for 4 h. The *ee* value of the product was determined by HPLC analysis using a Chiralcel column. Experimental details are summarized in Table S6.

Table S6. Nonlinear relationship between the *ee* values of **L1** and the *ee* values of **2b** in DCM[a].

Entry	*ee* of **L1** (%)	*ee* of **2b** (%)[b]
1	0	0
2	20	16
3	40	36
4	60	54
5	80	69
6	100	89

[a]Reaction conditions: **1b** (0.1 mmol), HBpin (2.0 equiv), **L1** (0.002 mmol), $Zn(OAc)_2$ (0.006 mmol), DCM (1.0 mL), r.t., 4 h. [b]The *ee* values of **2b** were determined by chiral HPLC.

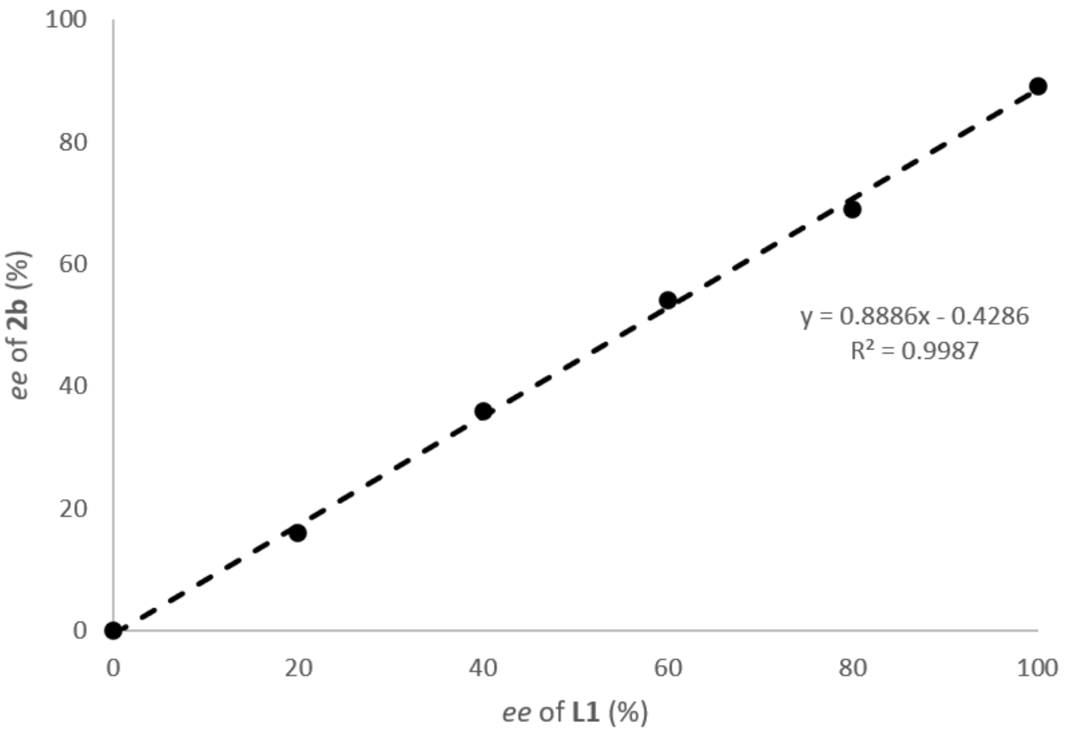

Figure S13. NLE plot between **L1** and **2b** in DCM.

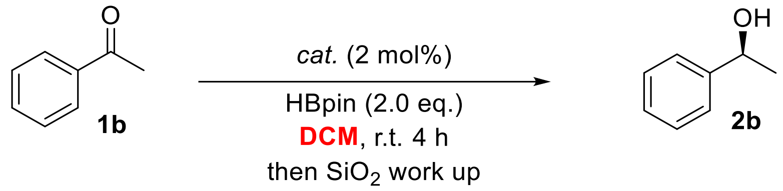

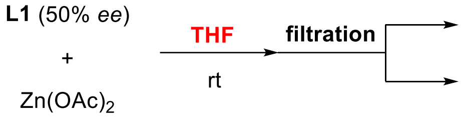

cat.	Conv. (%)	*ee* (%)
cat. in filtrate	99	72
cat. in precipitate	99	8

To a Schlenk tube equipped with magnetic stirring bar were added $Zn(OAc)_2$ (27.5 mg, 0.15 mmol), **L1** with 50% *ee* [(*S, S, S*)-**L1** (25.9 mg, 0.0375 mmol), (*R, R, R*)-**L1** (8.6 mg, 0.0125 mmol)] and THF (5.0 mL) in glove box filled with N_2. The mixture was stirred at r.t. overnight and was then filtered to give 21.1 mg precipitate as a yellow solid. The filtrate was concentrated in vacuum to give 23.3 mg yellow solid. In an oven-dried 5.0 mL tube equipped with a stir bar was added *cat.* (2.2 mg, 0.002 mmol, 2.0 mol%), **1b** (12 μL, 0.1 mmol) and HBpin (29 μL, 2.0 equiv), DCM (1.0 mL). The resulting mixture was stirred at r.t. for 4 h. The conversion of **1b** was determined by GC using *n*-hexane as the internal standard. Then the mixture was concentrated under reduced pressure. The residue was purified by silica gel chromatography to give the desired product **2b**. The *ee* value of the product was determined by HPLC analysis using a Chiralcel column.

2.4.4 Kinetic experiments with different ratio of $Zn(OAc)_2$ to (*S, S, S*)-L1.

The IR probe of the ReactIR was inserted through an adapter into the middle neck of a three necked reaction vessel which was fitted with a magnetic stirring bar. The left neck was capped by an argon line, and the right neck was capped with a rubber plug. Following evacuation under vacuum and flushing with argon for three times, the $Zn(OAc)_2$/(*S, S, S*)-**L1** catalyst solution was added [$Zn(OAc)_2$ (0.37x mg, 0.002x mmol, 0.1x mol%, x = 1, 2, 3), (*S, S, S*)-**L1** (1.4 mg, 0.002 mmol, 0.1 mol%) and dry THF (5 mL) were added in a Schlenk tube and were pre-stirred until completely dissolved] and the data collection was started immediately, followed by sequential addition of the

HBpin (0.58 mL, 4.0 mmol, 2.0 equiv) and **1b** (0.24 mL, 2.0 mmol).

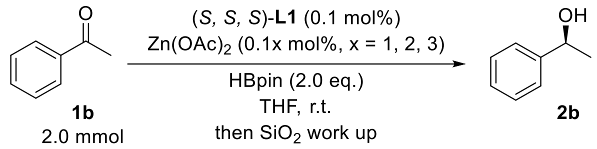

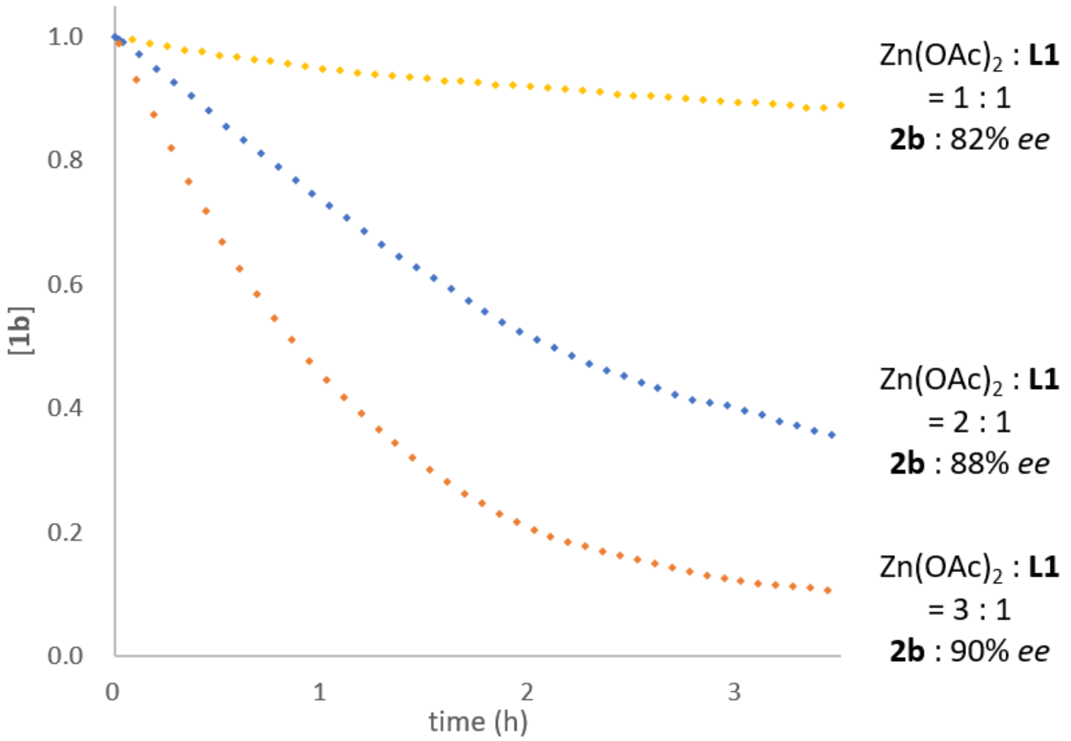

Figure S14. Different reaction rate of **1b** with different ratio of $Zn(OAc)_2$ to (*S, S, S*)-**L1**.

2.4.5 ESI-MS and gMS^2 analysis of the catalyst in different *ee* values.

Electrospray ionization-mass spectrometry (ESI-MS) and gradient tandem mass spectrometry (gMS^2) were collected on a Waters Synapt G2-Si mass spectrometer (Waters Corp., Milford, MA, USA). The samples were dissolved in MeOH with a concentration of 0.5 mg/mL. The ESI-MS experiments were performed under the following conditions: ESI capillary voltage, 0.5 kV; sample cone voltage, 5 V; source offset, 0 V; source temperature, 100 °C; desolvation temperature, 100 °C; cone gas flow, 80 L/h; desolvation gas flow, 800 L/h (N_2); source gas control, 0 mL/min; sample flow rate, 10 μL/min.

Gradient MS^2 spectra were recorded under the following conditions: the reaction

intermediates (m/z = 2182) were isolated by quadrupole for the following collision induced dissociation (CID), in which collision energy was gradually increased by changing the voltages of trap cell (Ar was pressurized into the trap cell with 1.5 mL/min flow rate). The product ions exiting the trap cell were accelerated orthogonally into the TOF analyzer for mass analysis.

1) ESI-MS of compound Zn_3**L1**

HRMS (ESI) m/z: calcd. for $C_{50}H_{49}N_4O_{10}Zn_3^{\bullet+}$: 1061.1323, Found: 1061.2119 [M-OAc]$^{\bullet+}$.

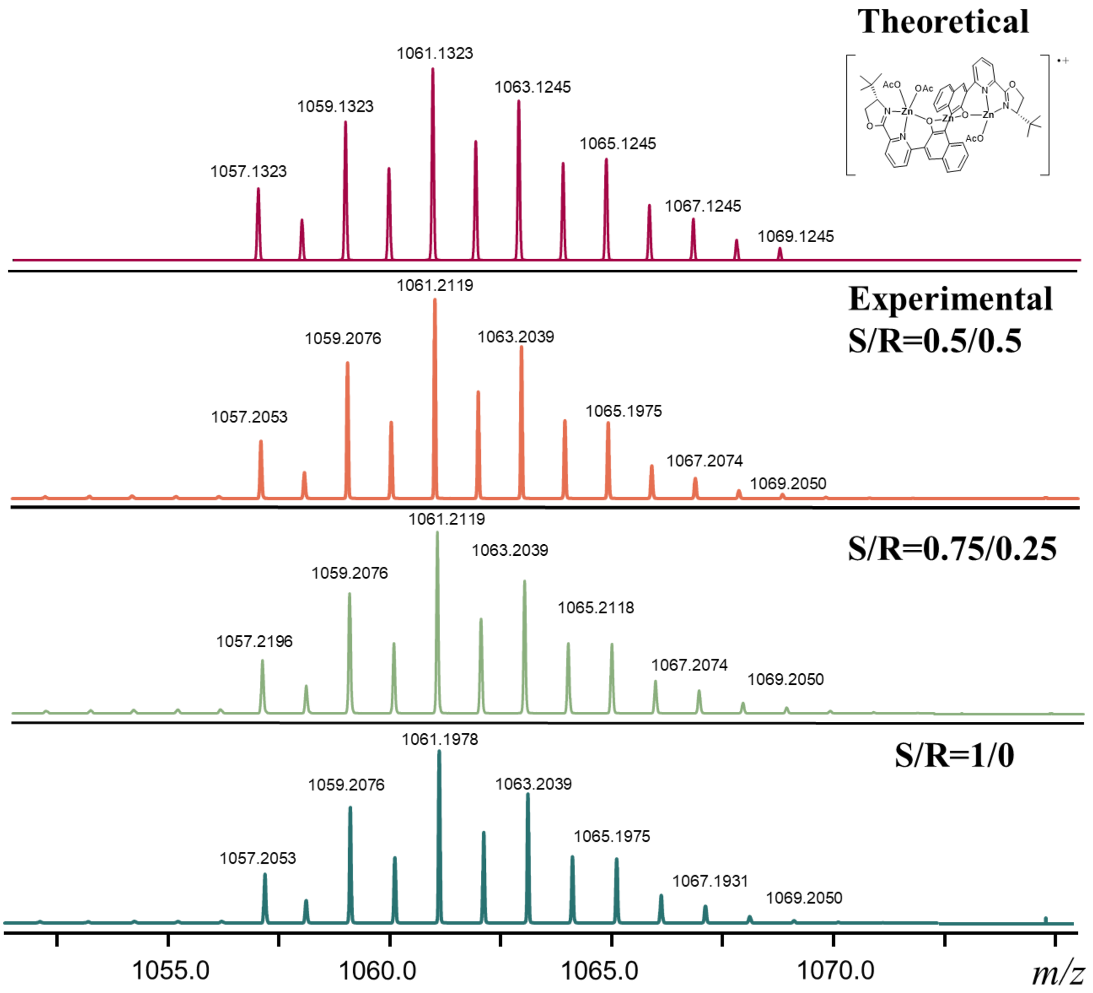

Figure S15. The experimental and simulated isotope patterns of the compound Zn_3**L1**.

2) ESI-MS of compound Zn_6**L1**$_2$

HRMS (ESI) m/z: calcd. for $C_{102}H_{101}N_8O_{22}Zn_6^{\bullet+}$: 2183.2686, Found: 2183.4688 [M-

OAc]$^{•+}$.

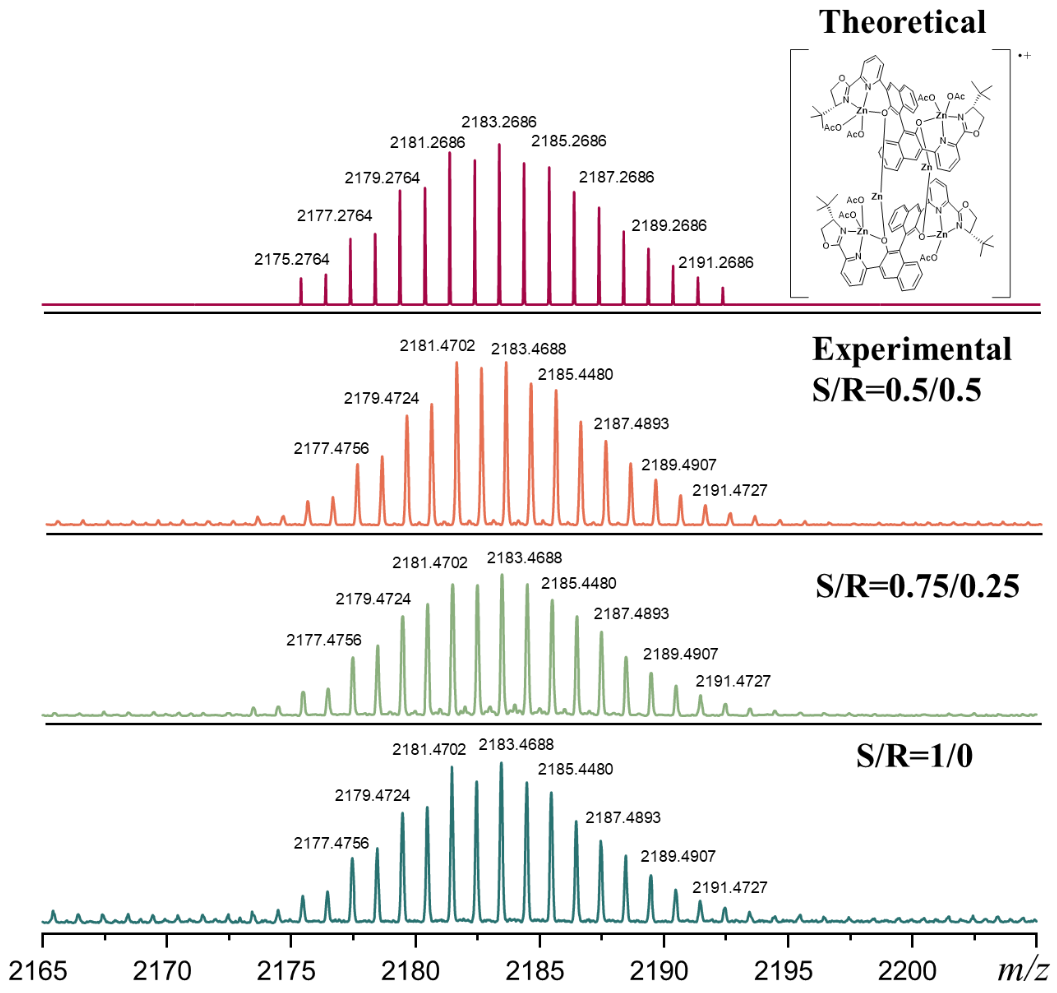

Figure S16. The experimental and simulated isotope patterns of the compound Zn_6**L1**$_2$.

3) The gMS^2 at *m/z* 2183 of compound Zn_6**L1**$_2$

The gMS^2 was applied to further unveil the structural information of the precursor ions at *m/z* = 2183 with different collision energy (Figure S17), where the precursor ions underwent collision-induced dissociation to lose [OAc]$^•$, generating [Zn_3**L1**]$^{•+}$ fragment ions. The experimental isotope patterns of product ions perfectly matched the simulated isotope pattern (Figure S18).

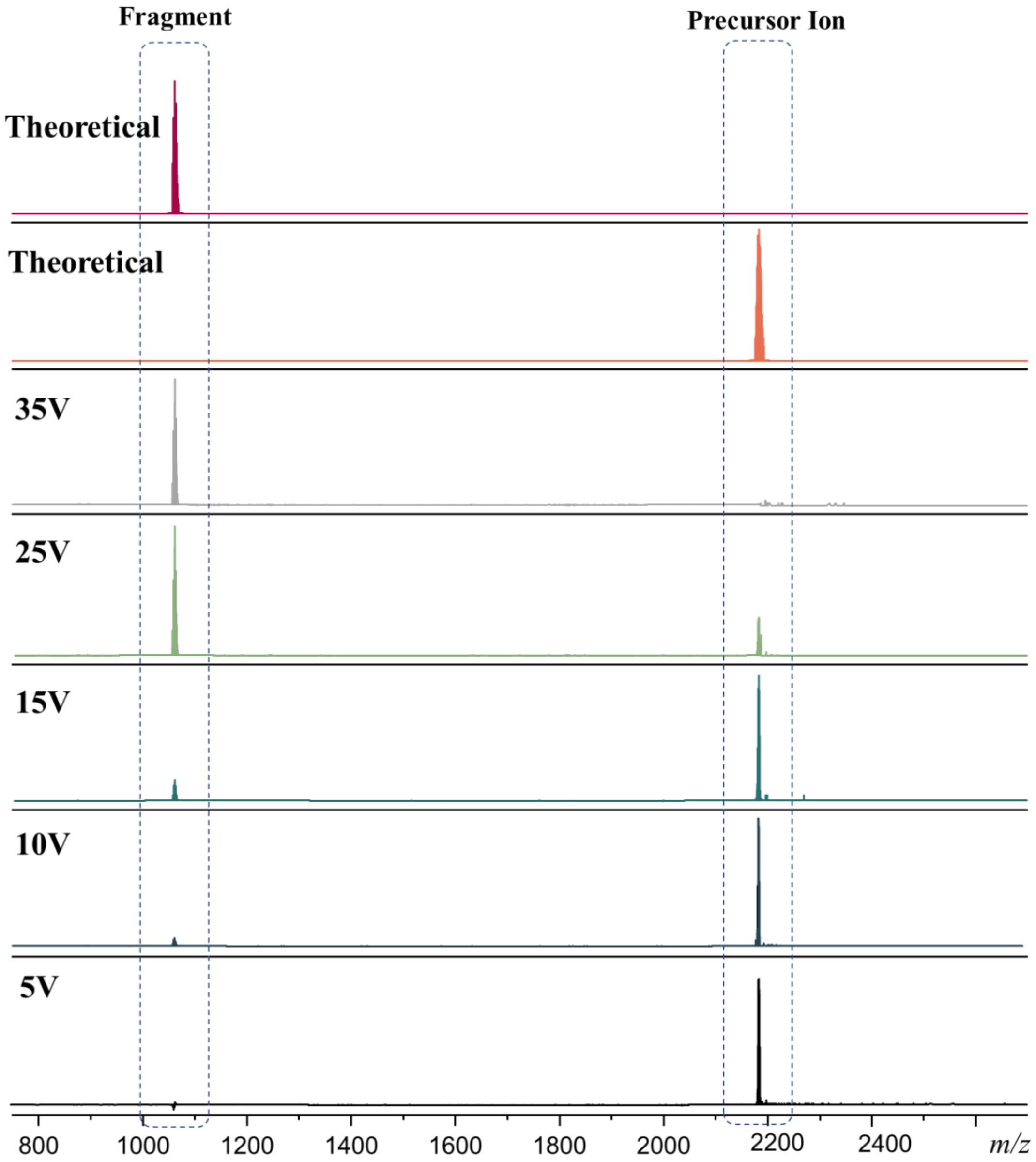

Figure S17. The gMS^2 of the selected compound Zn_6**L1**$_2$ of m/z = 2183 with different collision energy.

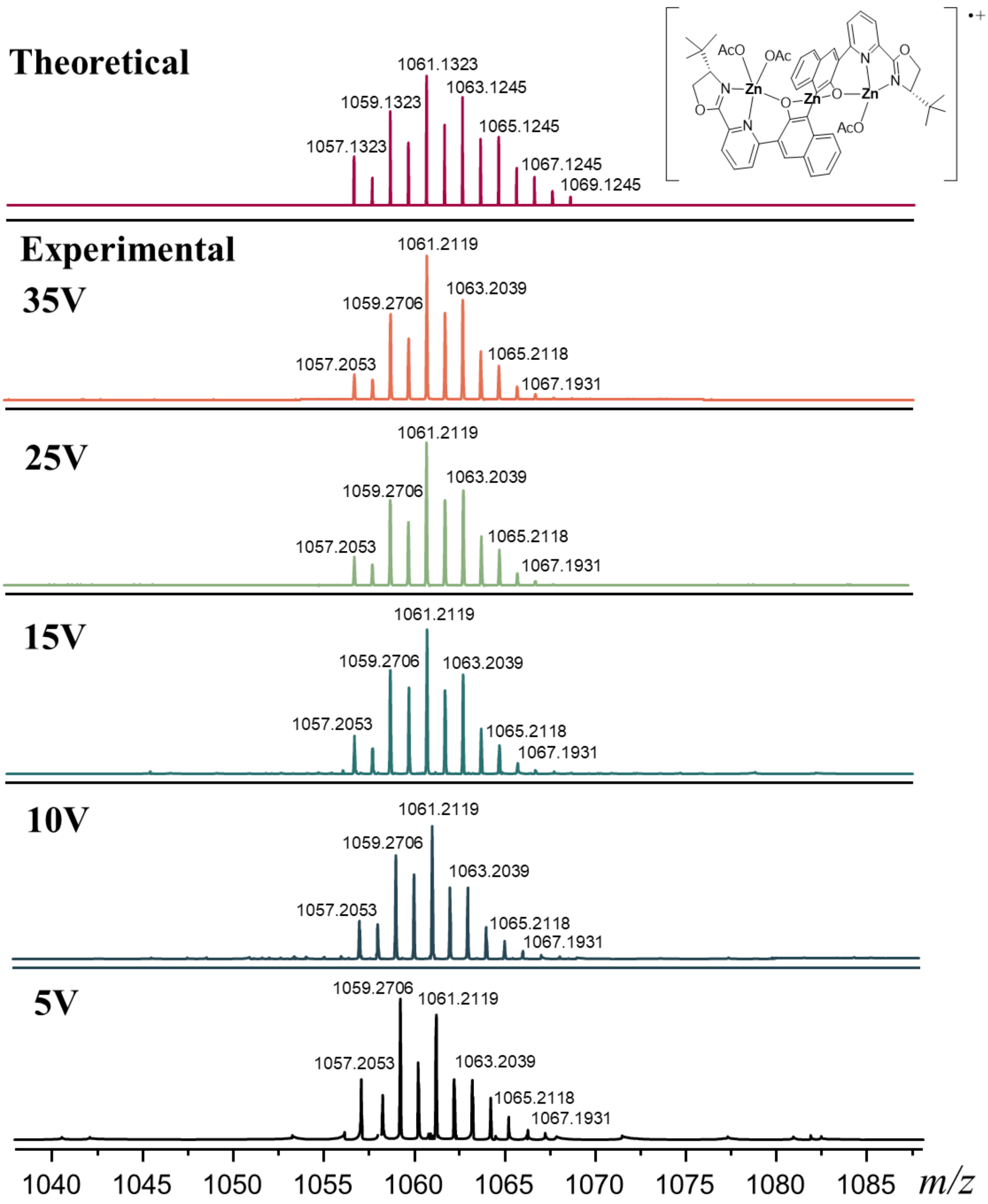

Figure S18. The experimental and simulated isotope patterns of product ion $Zn_3\mathbf{L1}_1$ with m/z = 1061 dissociated from $Zn_6\mathbf{L1}_2$.

2.4.6 NMR analysis of the possible Zn-H species

Procedure for the NMR analysis:

To a Schlenk tube equipped with a magnetic stirring bar in glove box filled with N_2 was added AcOH (57 μL, 1.0 mmol, 1.0 equiv), HBpin (145 μL, 1.0 mmol, 1.0 equiv) and

$CDCl_3$ (1.0 mL), the mixture was stirred at r.t. for 30 min. The mixture was taken for ^1H NMR analysis, and the result is shown in Figure S19 (i). HBpin was bought from Adamas and the ^1H NMR analysis is shown in Figure S19 (ii).

To a Schlenk tube equipped with a magnetic stirring bar in glove box filled with N_2 was added Zn_3**L1** complex (11.2 mg, 0.01 mmol) and $CDCl_3$ (1.0 mL), the mixture was stirred at r.t. for 3 min. After Zn_3**L1** was fully dissolved, using a micropipette, 0.5 mL of the solution was taken for ^1H NMR analysis, and the result is shown in Figure S19 (iii). At the same time, 0.5 mL of the solution was placed in an NMR tube, followed by the addition of HBpin (2.2 μL, 3.0 equiv) to the NMR tube. The result of the subsequent ^1H NMR analysis was shown in Figure S19 (iv).

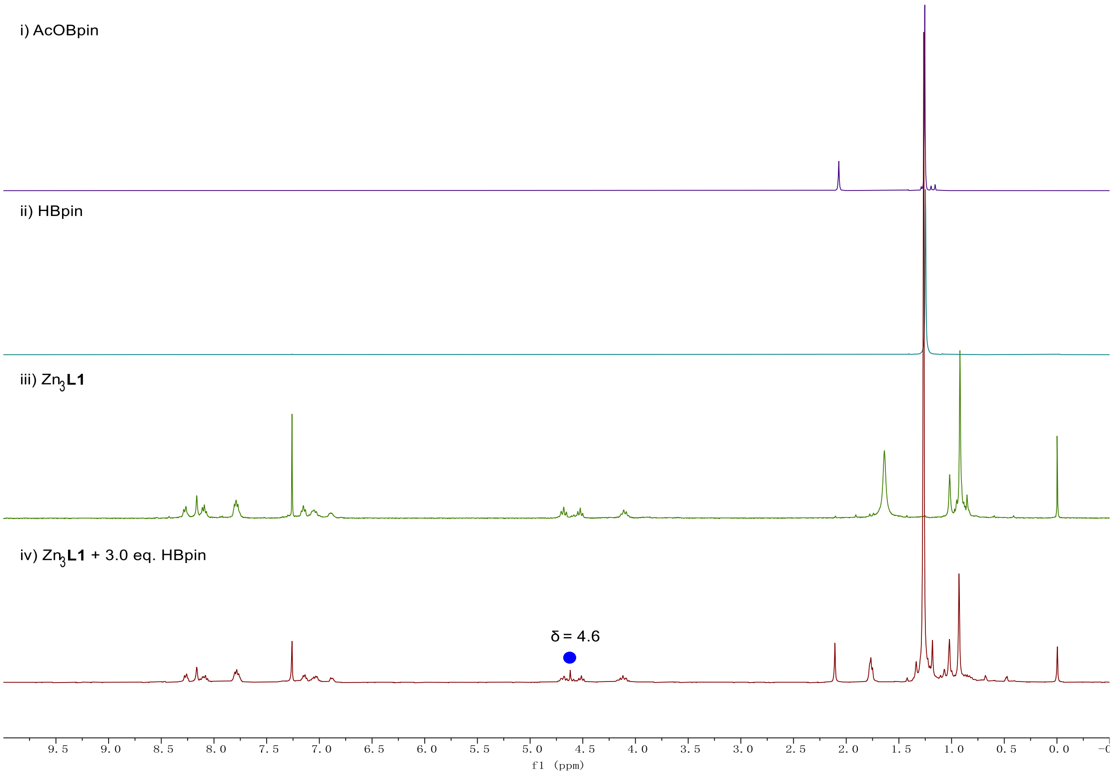

Figure S19. The ^1H NMR spectra [400 MHz, $CDCl_3$] of AcOBpin (i), HBpin (ii), Zn_3**L1** (iii) and Zn_3**L1**+3.0 eq. HBpin (iv).

3. X-ray Crystallographic

3.1 X-ray Crystallographic for (*S*, *S*, *S*)-L1

Crystals suitable for X-ray single-crystal diffraction analysis was obtained through

slowly evaporating the mixture solution of DCM and *n*-hexane at room temperature. CIF file for (*S, S, S*)-**L1** has been deposited at the Cambridge Crystallographic Data Centre with deposition number 2454252. Copies of these data can be obtained, free of charge, on application to the CCDC, 12 Union Road, Cambridge CB2 1EZ, UK [fax: +44(1223)336033; e-mail: deposit@ccdc.cam.ac.uk].

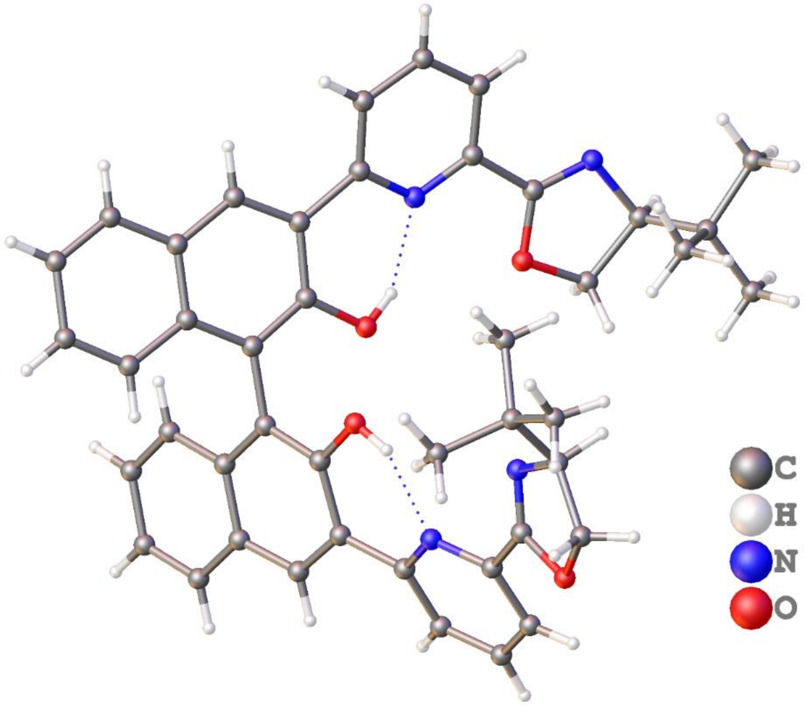

Figure S20. X-ray structure of (*S, S, S*)-**L1** with thermal ellipsoids shown at 50% probability.

Table S7. Crystal data and structure refinement for (*S, S, S*)-**L1**

Identification code	(*S, S, S*)-**L1**
Empirical formula	$C_{44}H_{42}N_4O_4$
Formula weight	690.81
Temperature	173.00 K
Wavelength	1.34139 Å
Crystal system	Orthorhombic
Space group	$P2_12_12_1$
a/Å	23.1920(6)

b/Å	23.5097(8)
c/Å	25.2306(7)
α/°	90
β/°	90
γ/°	90
Volume	13756.7(7) $Å^3$
Z	12
Density (calculated)	1.001 Mg/m^3
Absorption coefficient	0.327 mm^{-1}
F(000)	4392
Crystal size	0.17 x 0.17 x 0.05 mm^3
Theta range for data collection	2.783 to 55.030°.
Index ranges	-19<=h<=28, -28<=k<=28, -30<=l<=30
Reflections collected	186313
Independent reflections	26194 [R(int) = 0.1013]
Completeness to theta = 53.594°	99.9 %
Absorption correction	Semi-empirical from equivalents
Max. and min. transmission	0.7508 and 0.6161
Refinement method	Full-matrix least-squares on F^2
Data / restraints / parameters	26194 / 0 / 1429
Goodness-of-fit on F^2	1.019
Final R indices [I>2sigma(I)]	R1 = 0.0677, wR2 = 0.1908
R indices (all data)	R1 = 0.0809, wR2 = 0.2037
Absolute structure parameter	0.02(9)
Extinction coefficient	n/a

Largest diff. peak and hole	0.672 and -0.333 e.Å^{-3}

3.2 X-ray Crystallographic for Zn_3**L1**

Crystals suitable for X-ray single-crystal diffraction analysis was obtained through slowly evaporating the mixture solution of MeCN and ethyl acetate cooling by gradient. CIF file for Zn_3**L1** has been deposited at the Cambridge Crystallographic Data Centre with deposition number 2454347. Copies of these data can be obtained, free of charge, on application to the CCDC, 12 Union Road, Cambridge CB2 1EZ, UK [fax: +44(1223)336033; e-mail: deposit@ccdc.cam.ac.uk].

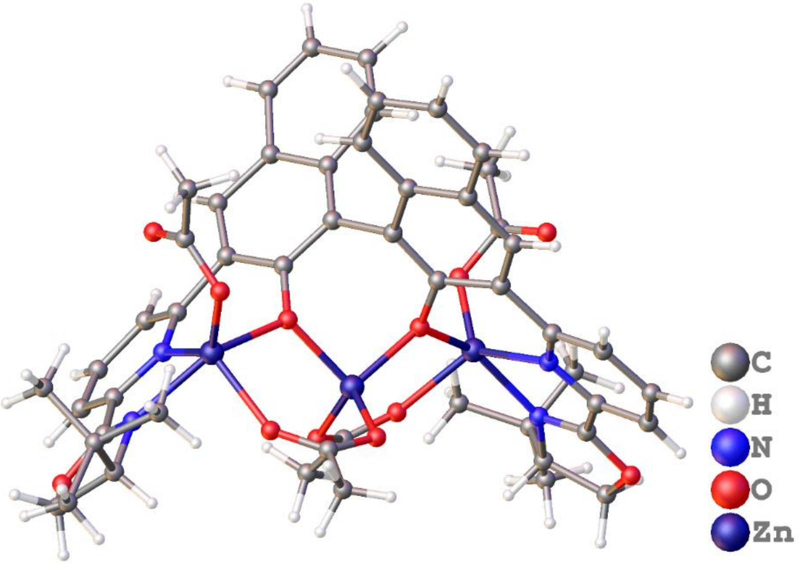

Figure S21. X-ray structure of Zn_3**L1** with thermal ellipsoids shown at 50% probability.

Table S8. Crystal data and structure refinement for Zn_3**L1**

Identification code	Zn_3**L1**
Empirical formula	$C_{110}H_{114}N_9O_{26}Zn_6$
Formula weight	2370.32
Temperature/K	172.99(10)
Crystal system	tetragonal
Space group	$P4_1$
a/Å	20.0152(5)

b/Å	20.0152(5)
c/Å	37.6884(14)
α/°	90
β/°	90
γ/°	90
Volume/Å^3	15098.3(9)
Z	4
ρ_{calc}g/cm^3	1.043
μ /mm^{-1}	1.514
F(000)	4900.0
Crystal size/mm^3	0.27 × 0.26 × 0.11
Radiation	Cu Kα (λ = 1.54184)
2Θ range for data collection/°	6.244 to 134.09
Index ranges	-23 ≤ h ≤ 16, -23 ≤ k ≤ 23, -44 ≤ l ≤ 45
Reflections collected	180584
Independent reflections	26916 [R_{int} = 0.1505, R_{sigma} = 0.0989]
Data/restraints/parameters	26916/49/1381
Goodness-of-fit on F^2	1.050
Final R indexes [I>=2σ (I)]	R_1 = 0.1109, wR_2 = 0.2865
Final R indexes [all data]	R_1 = 0.1520, wR_2 = 0.3206
Largest diff. peak/hole / e Å^{-3}	1.27/-0.44
Flack parameter	0.031(17)

4. References

1. He, Y.; Bian, Z.; Kang, C.; Gao, L. Stereoselective and Hierarchical Self-Assembly from Nanotubular Homochiral Helical Coordination Polymers to Supramolecular Gels. *Chem. Commun.* **2010**, *46 (31)*, 5695–5697.

2. Zhang, L.; Tang, Y.; Han, Z.; Ding, K. Lutidine-Based Chiral Pincer Manganese Catalysts for Enantioselective Hydrogenation of Ketones. *Angew. Chem. Int. Ed.* **2019**, *58 (15)*, 4973–4977.

3 Wang, B.; Jiang, L.; Wang, J.; Ma, J.; Liu, M.; Yu, H. A Tandem and Fully Enzymatic Procedure for the Green Resolution of Chiral Alcohols: Acylation and Deacylation in Non-Aqueous Media. *Tetrahedron: Asymmetry* **2011**, *22 (9)*, 980–985.

4 Vasilenko, V.; Blasius, C. K.; Wadepohl, H.; Gade, L. H. Mechanism-Based Enantiodivergence in Manganese Reduction Catalysis: A Chiral Pincer Complex for the Highly Enantioselective Hydroboration of Ketones. *Angew. Chem. Int. Ed.* **2017**, *56 (29)*, 8393–8397.

5 Falconnet, A.; Magre, M.; Maity, B.; Cavallo, L.; Rueping, M. Asymmetric Magnesium-Catalyzed Hydroboration by Metal-Ligand Cooperative Catalysis. *Angew. Chem. Int. Ed.* **2019**, *58 (49)*, 17567–17571.

6 Sun, Y.; Lu, C.; Zhao, B.; Xue, M. Enantioselective Hydroboration of Ketones Catalyzed by Rare-Earth Metal Complexes Containing Trost Ligands. *J. Org. Chem.* **2020**, *85 (16)*, 10504–10513.

7 Zhang, L.; Liu, C.; Sun, M.; Liang, C.; Cao, L.; Yao, X.; Ma, Y.; Cheng, R.; Ye, J. Iridium-Catalyzed Asymmetric Hydrogenation of Simple Ketones with Tridentate PNN Ligands Bearing Unsymmetrical Vicinal Diamines. *J. Org. Chem.* **2023**, *88 (5)*, 2942–2951.

8 Su, Y.; Ma, Z.; Wang, J.; Li, L.; Yan, X.; Ma, N.; Liu, Q.; Solan, G. A.; Wang, Z. Asymmetric Transfer Hydrogenation of Ketones Improved by PNN–Manganese Complexes. *J. Org. Chem.* **2024**, *89 (17)*, 12318–12325.

9 Garcia-Lopez, M.; Torrens-Jover, A. Indane-Amine Derivatives, Their Preparation and Use as Medicaments. WO2009003719A2, January 8, 2009.

10 Zhang, S.-X.; Long, L.; Li, Z.; He, Y.-M.; Li, S.; Chen, H.; Hao, W.; Fan, Q.-H. Rhodium-Catalyzed Homogeneous Asymmetric Hydrogenation of Naphthol Derivatives. *J. Am. Chem. Soc.* **2025**, *147 (6)*, 5197–5211.

11 Zong, Y.; Zou, X.; Song, J.; Chen, G.-Q.; Zhang, X. Chemoselective and Divergent Synthesis of Chlorohydrins and Oxaheterocycles via Ir-Catalyzed Asymmetric Hydrogenation. *Org. Lett.* **2023**, *25 (37)*, 6875–6880.

12 Liu, Y.; Yan, J.; Yuan, Q.; Ma, L.; Zhou, L.; He, Y.; Liu, G.; Yue, X.; Jiang, Y. Enzymatic Reduction of Halogenated Aryl Ketones in an Aqueous Micellar Solution with Enhanced Catalytic Performance. *Green Chem.* **2024**, *26 (11)*, 6666–6674.

13 Blasius, C. K.; Vasilenko, V.; Gade, L. H. Ultrafast Iron-Catalyzed Reduction of Functionalized Ketones: Highly Enantioselective Synthesis of Halohydrines, Oxaheterocycles, and Aminoalcohols. *Angew. Chem. Int. Ed.* **2018**, *57 (32)*, 10231–10235.

5. NMR Spectra

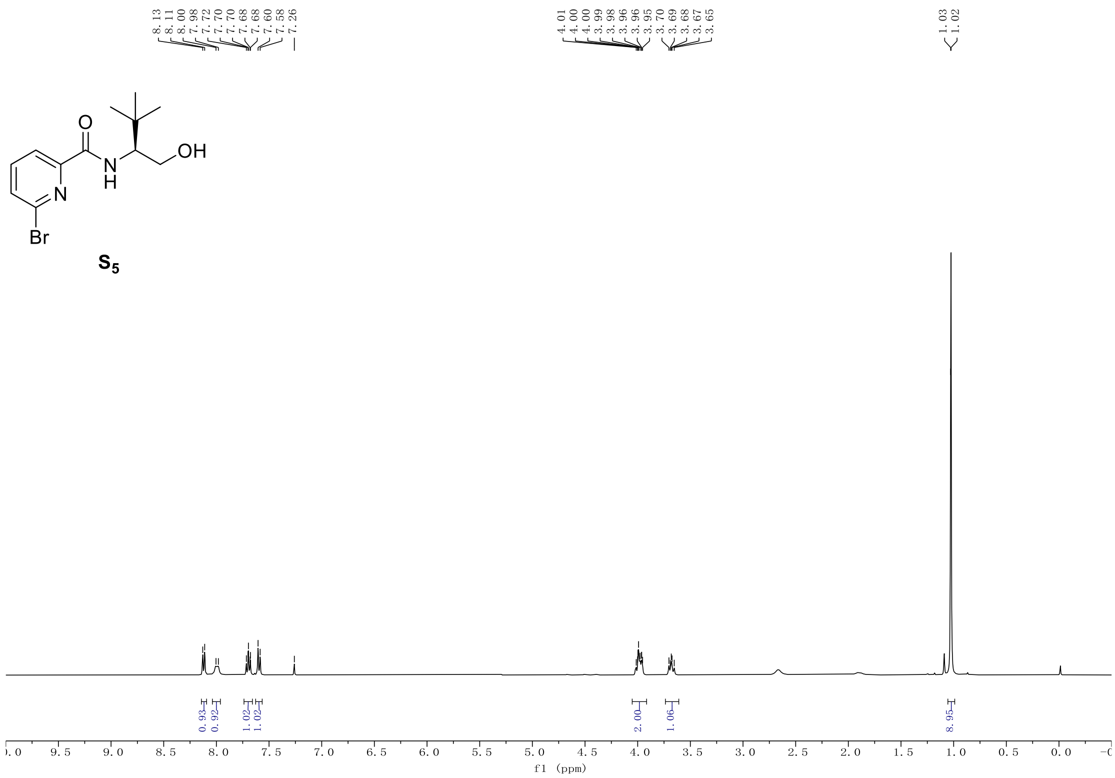

^{1}H NMR spectra (400 MHz, $CDCl_3$) of **S5**

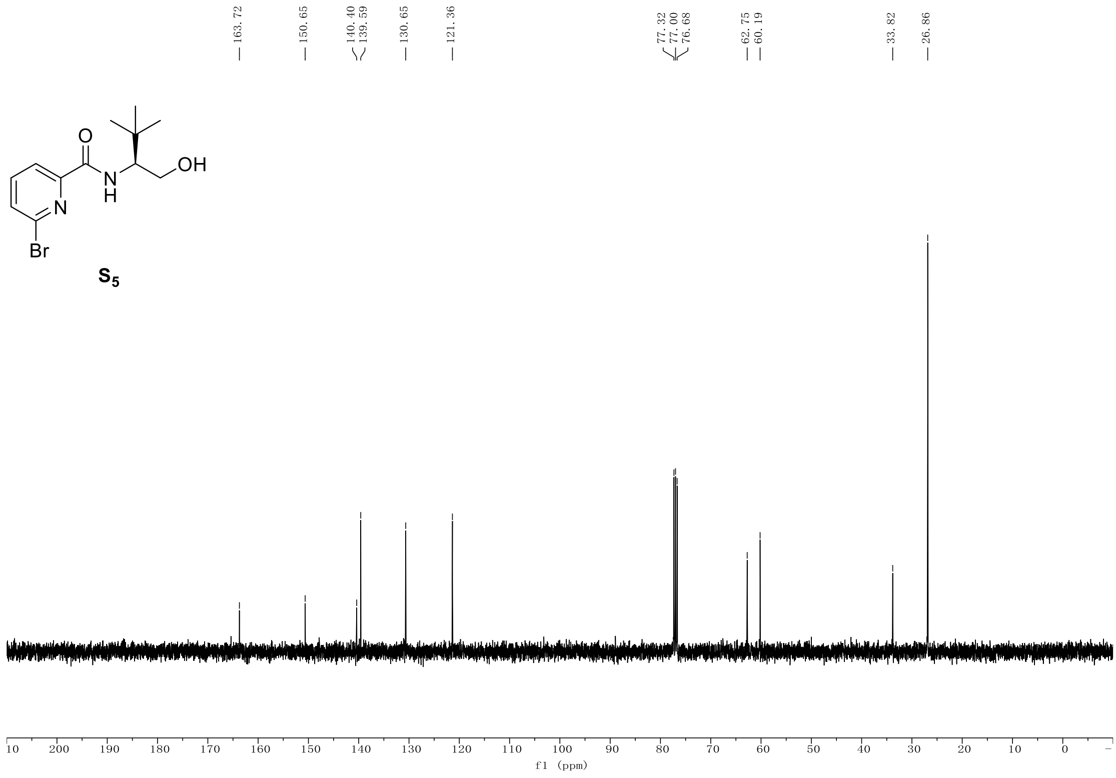

^{13}C NMR spectra (101 MHz, $CDCl_3$) of **S5**

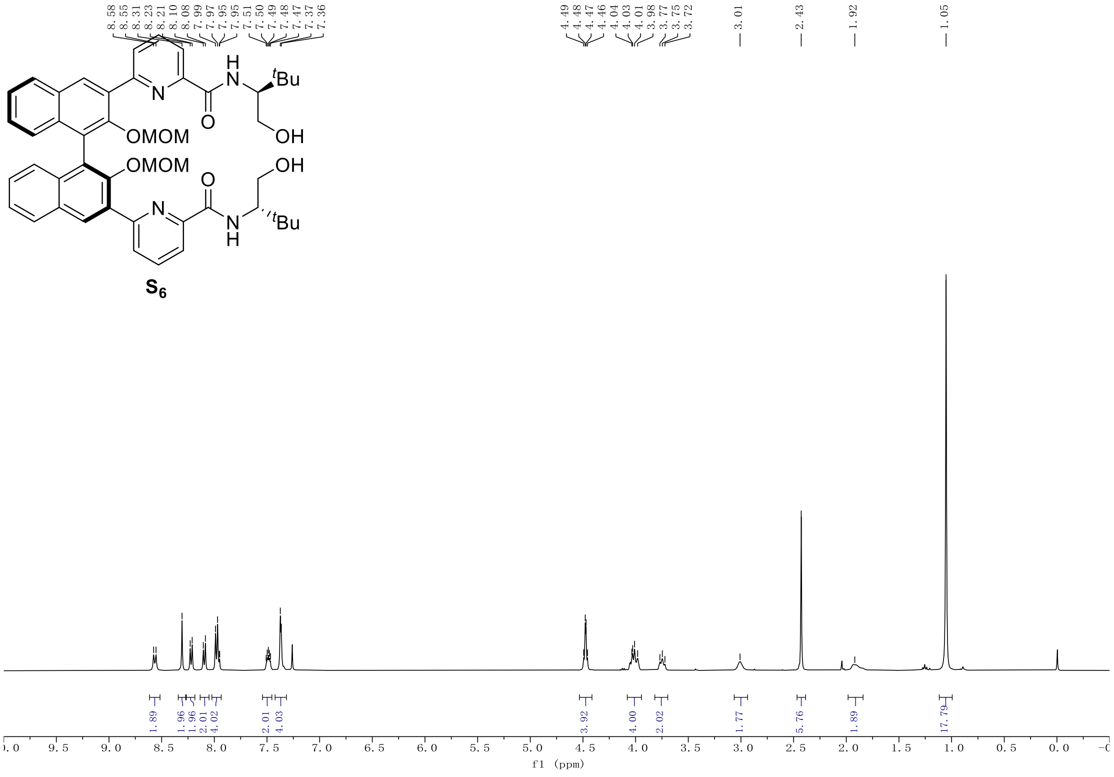

^{1}H NMR spectra (400 MHz, $CDCl_3$) of **S_6**

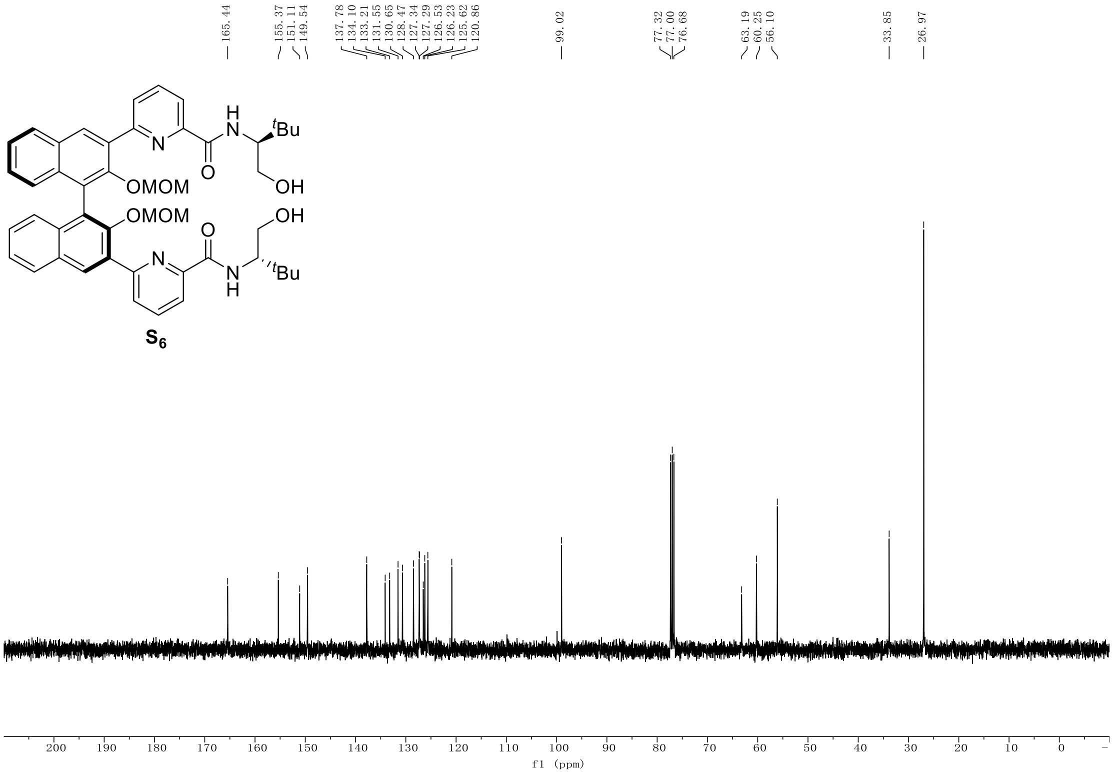

^{13}C NMR spectra (101 MHz, $CDCl_3$) of **S_6**

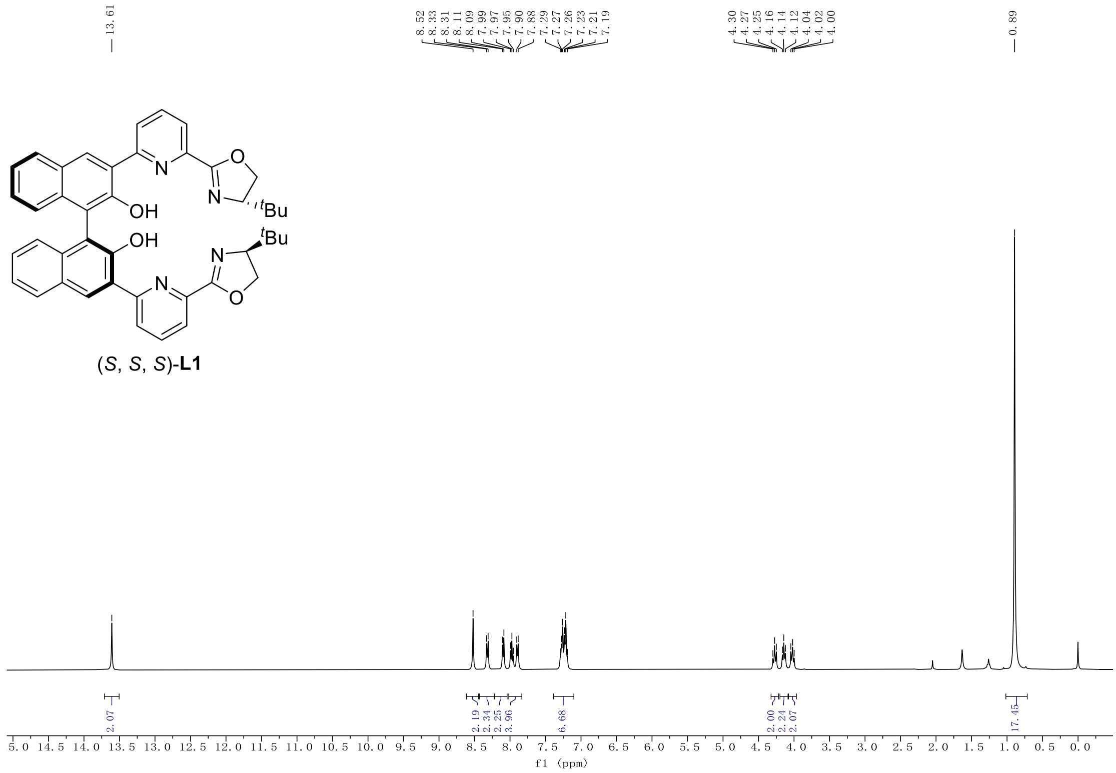

^{1}H NMR spectra (400 MHz, $CDCl_3$) of (*S, S, S*)-**L1**

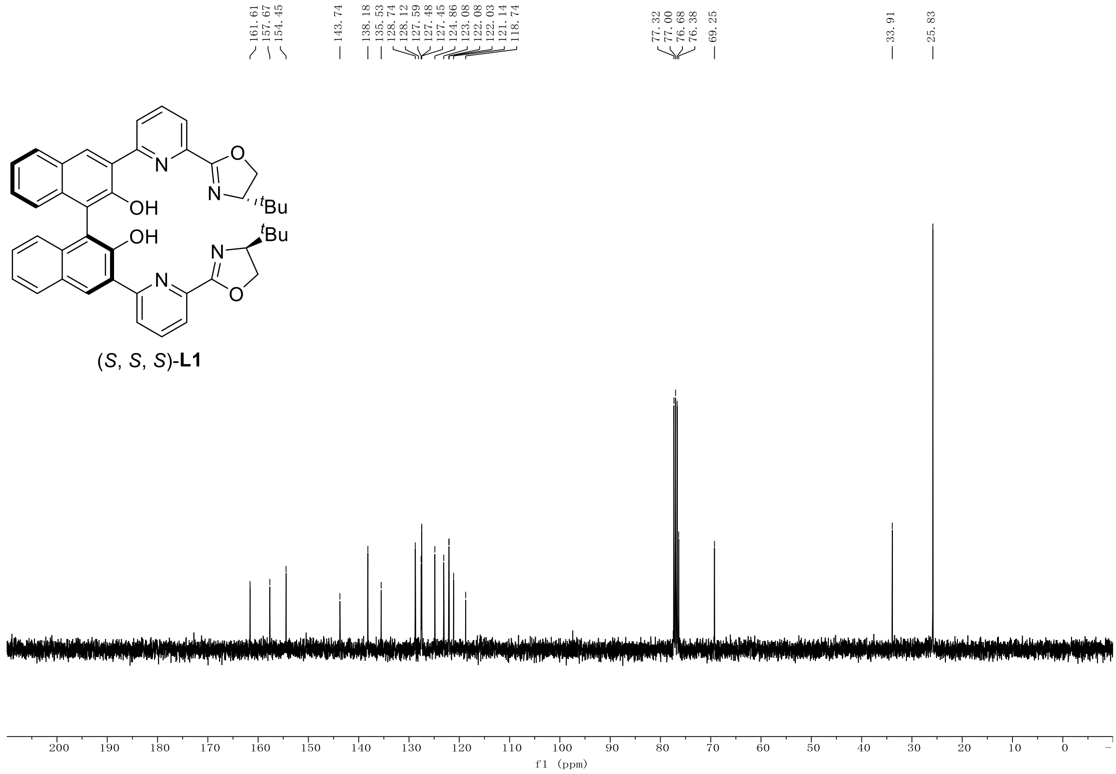

^{13}C NMR spectra (101 MHz, $CDCl_3$) of (*S, S, S*)-**L1**

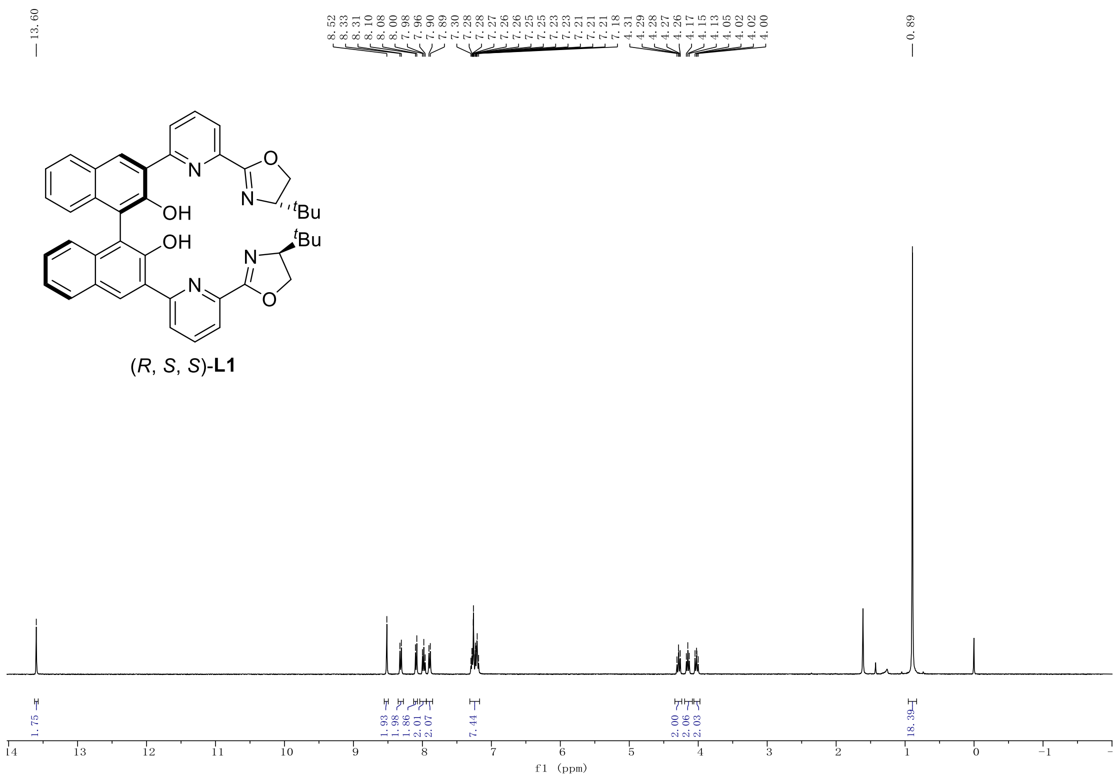

^{1}H NMR spectra (400 MHz, $CDCl_3$) of (*R, S, S*)-**L1**

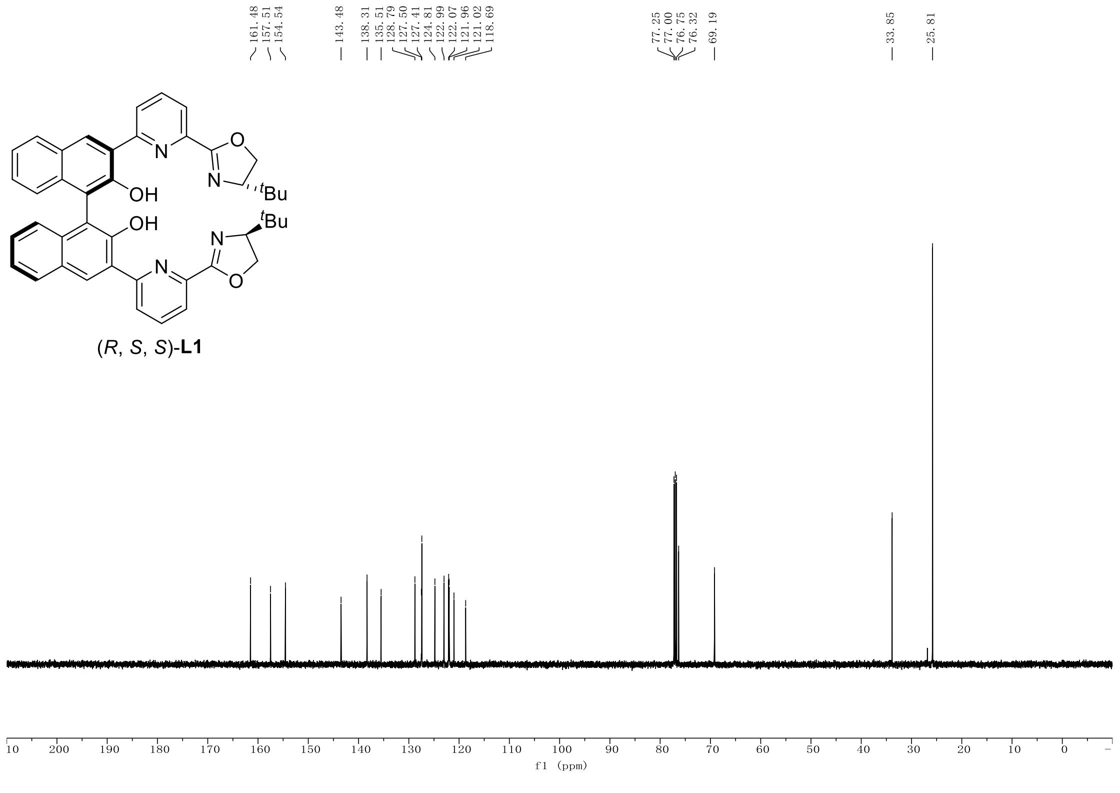

^{13}C NMR spectra (126 MHz, $CDCl_3$) of (*R, S, S*)-**L1**

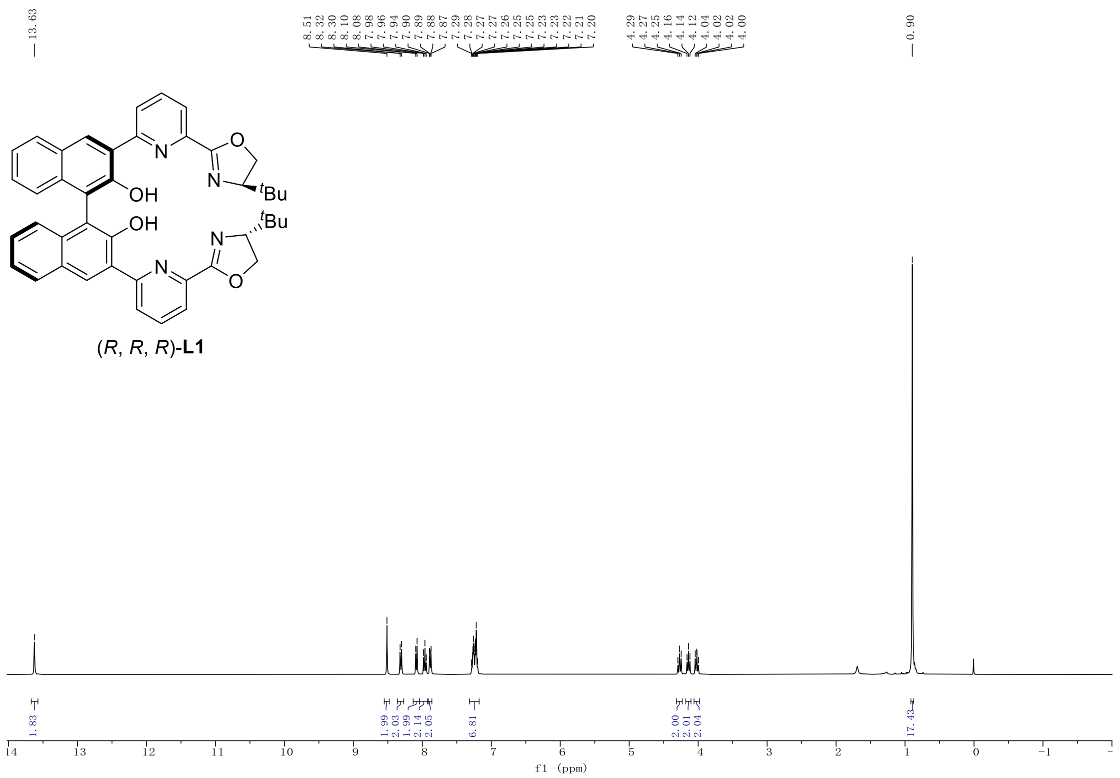

^{1}H NMR spectra (400 MHz, $CDCl_3$) of (*R, R, R*)-**L1**

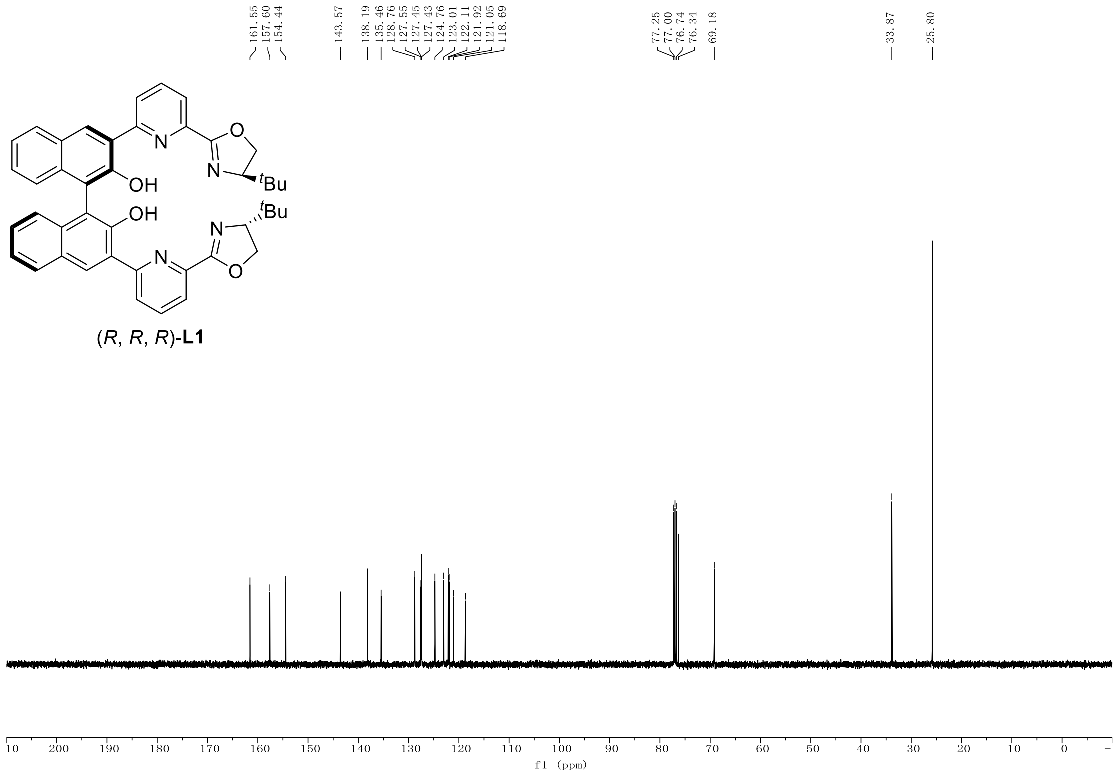

^{13}C NMR spectra (126 MHz, $CDCl_3$) of (*R, R, R*)-**L1**

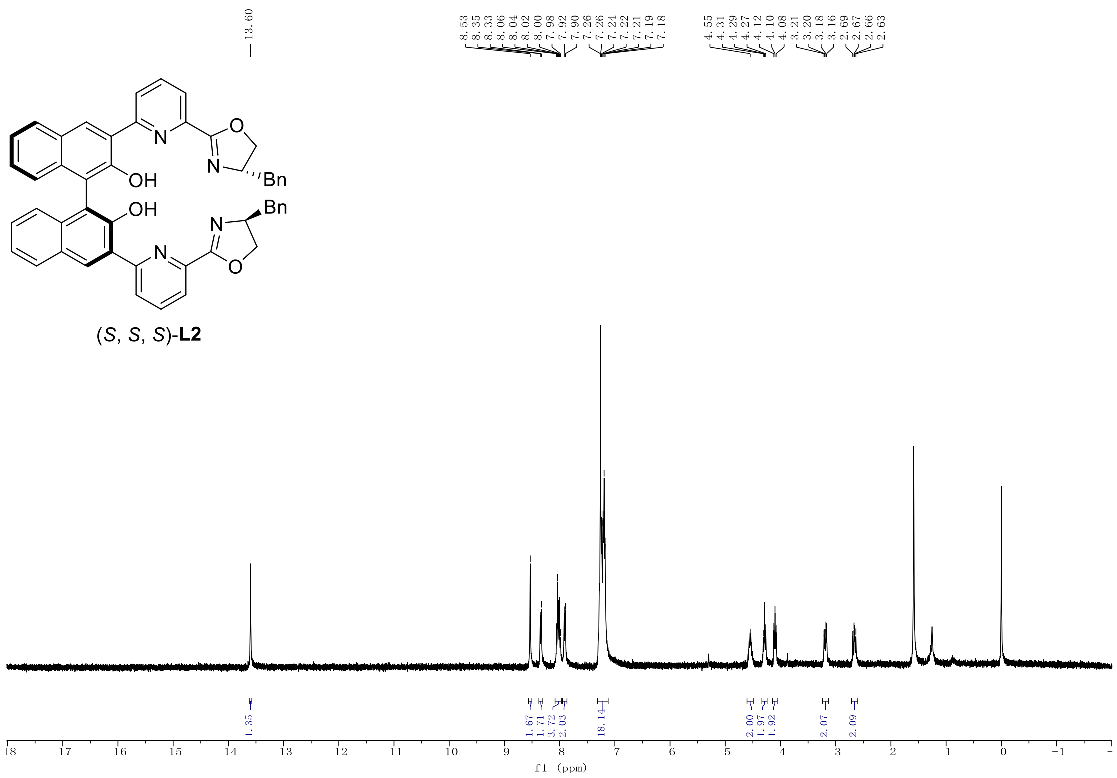

^{1}H NMR spectra (400 MHz, $CDCl_3$) of (*S, S, S*)-**L2**

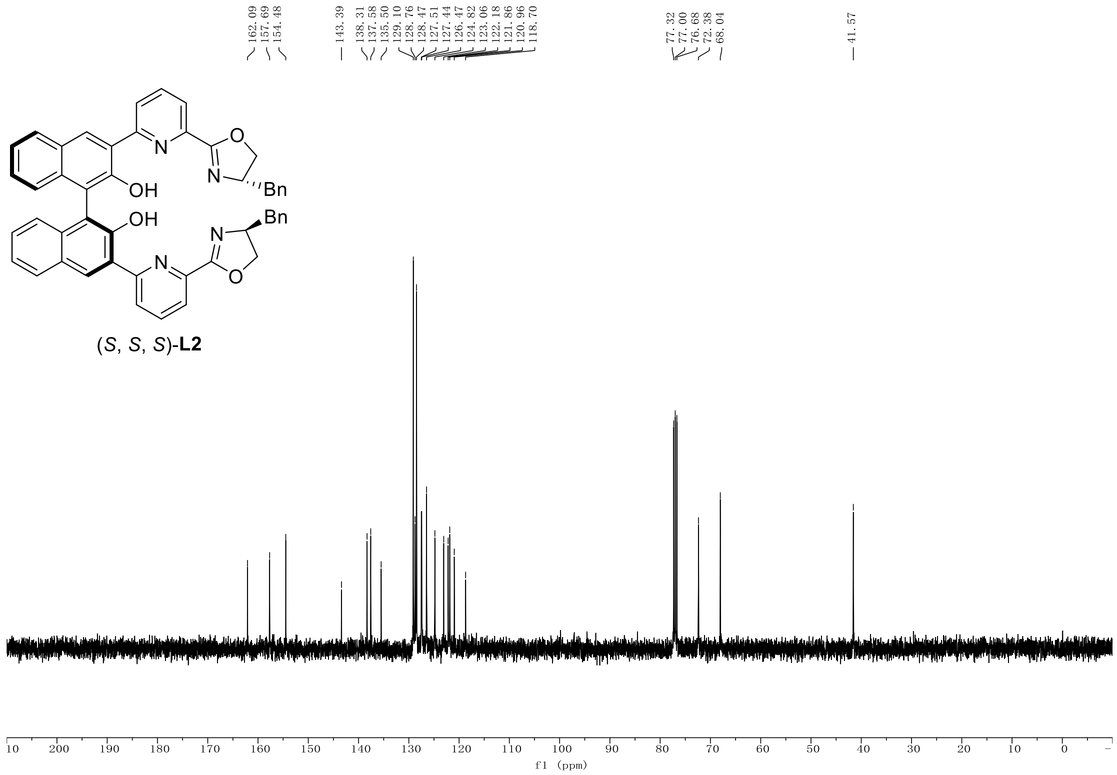

^{13}C NMR spectra (101 MHz, $CDCl_3$) of (*S, S, S*)-**L2**

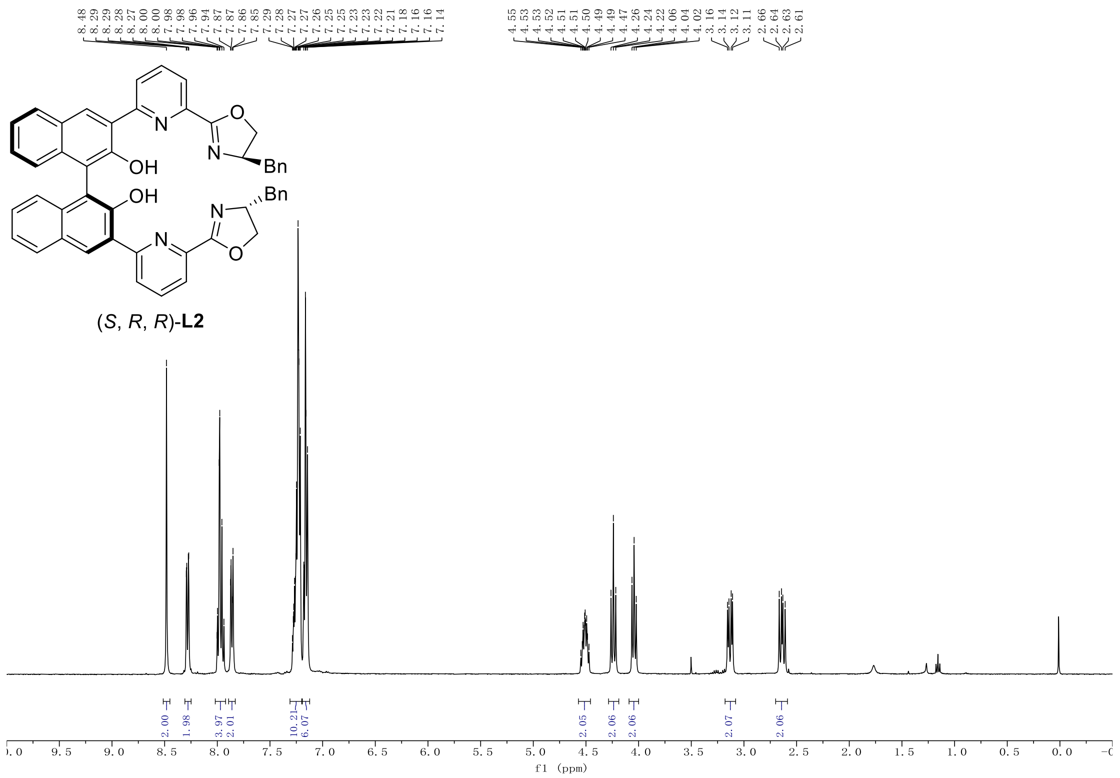

^{1}H NMR spectra (400 MHz, $CDCl_3$) of (*S, R, R*)-**L2**

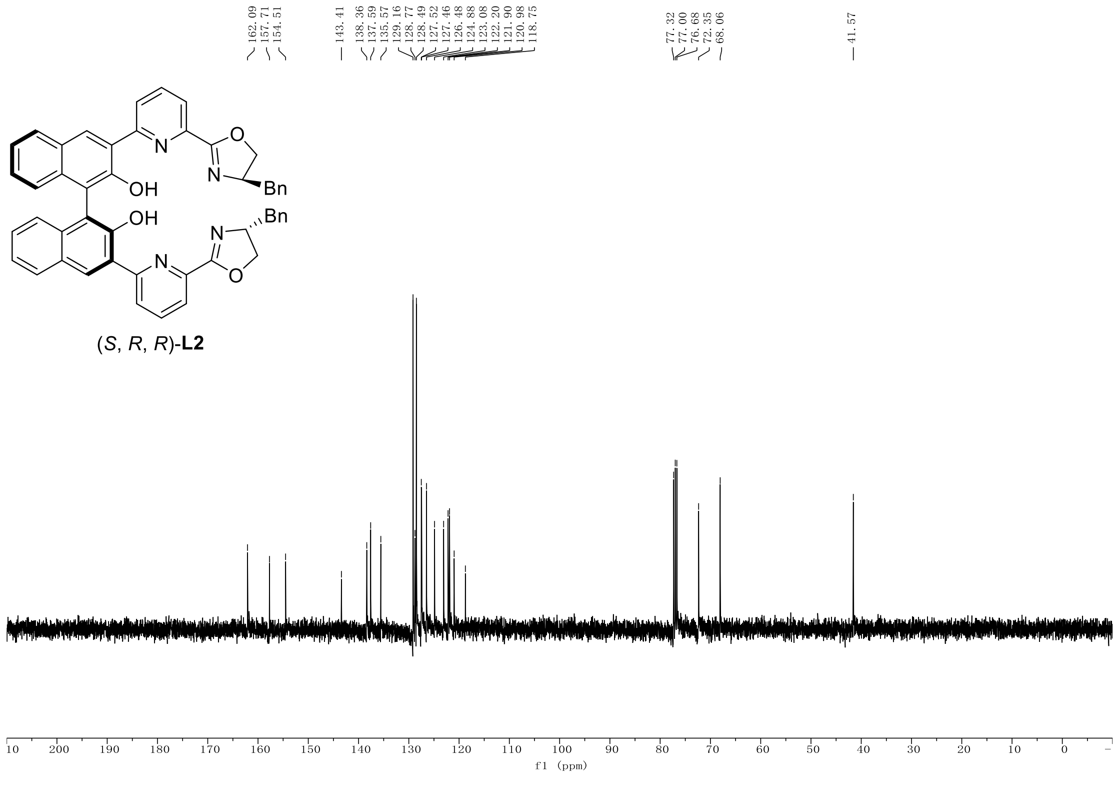

^{13}C NMR spectra (101 MHz, $CDCl_3$) of (*S, R, R*)-**L2**

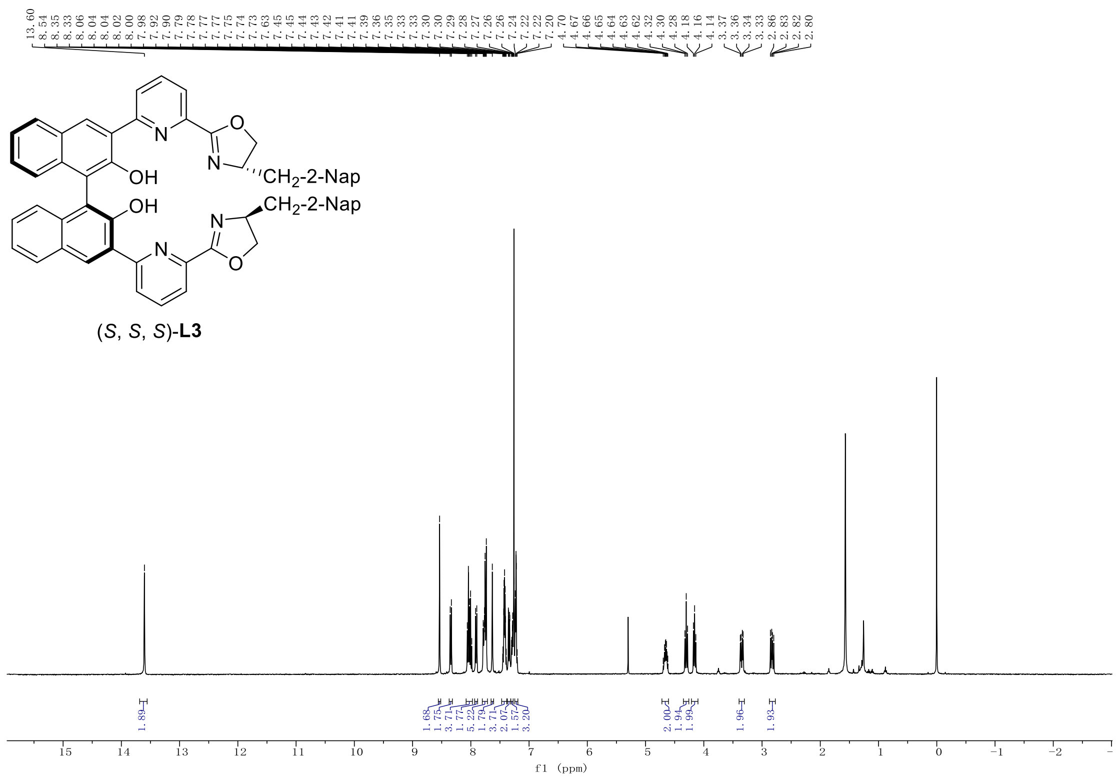

^{1}H NMR spectra (400 MHz, $CDCl_3$) of (*S, S, S*)-**L3**

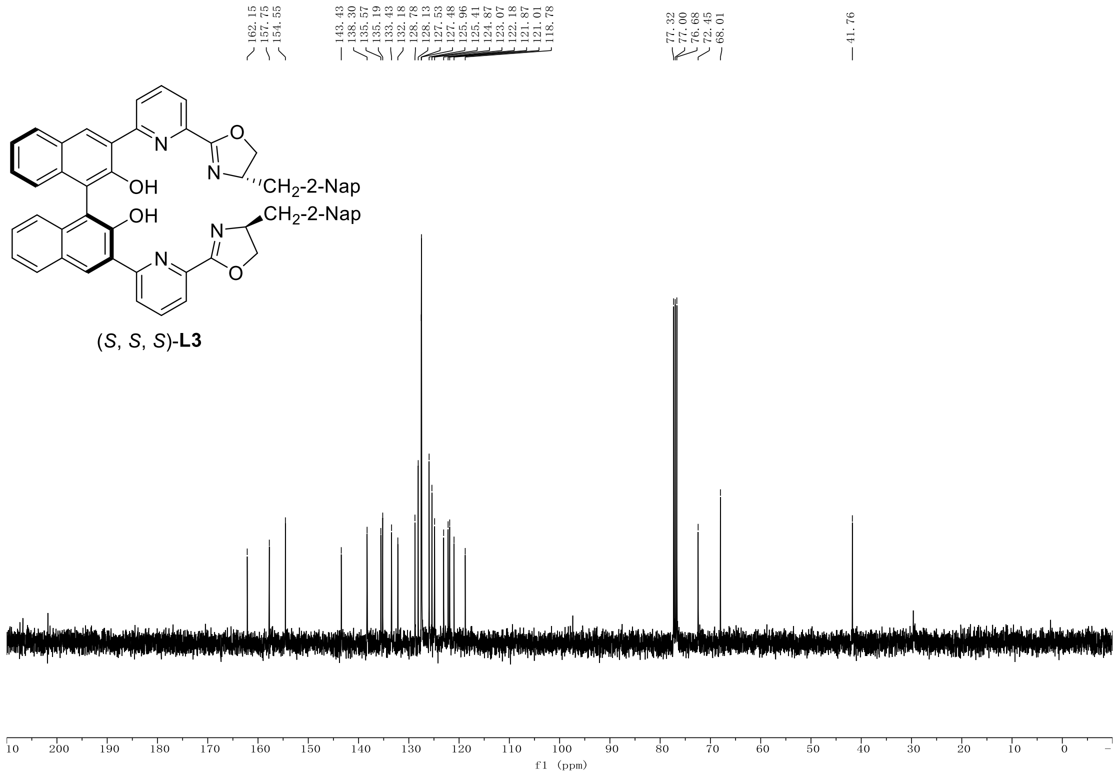

^{13}C NMR spectra (101 MHz, $CDCl_3$) of (*S, S, S*)-**L3**

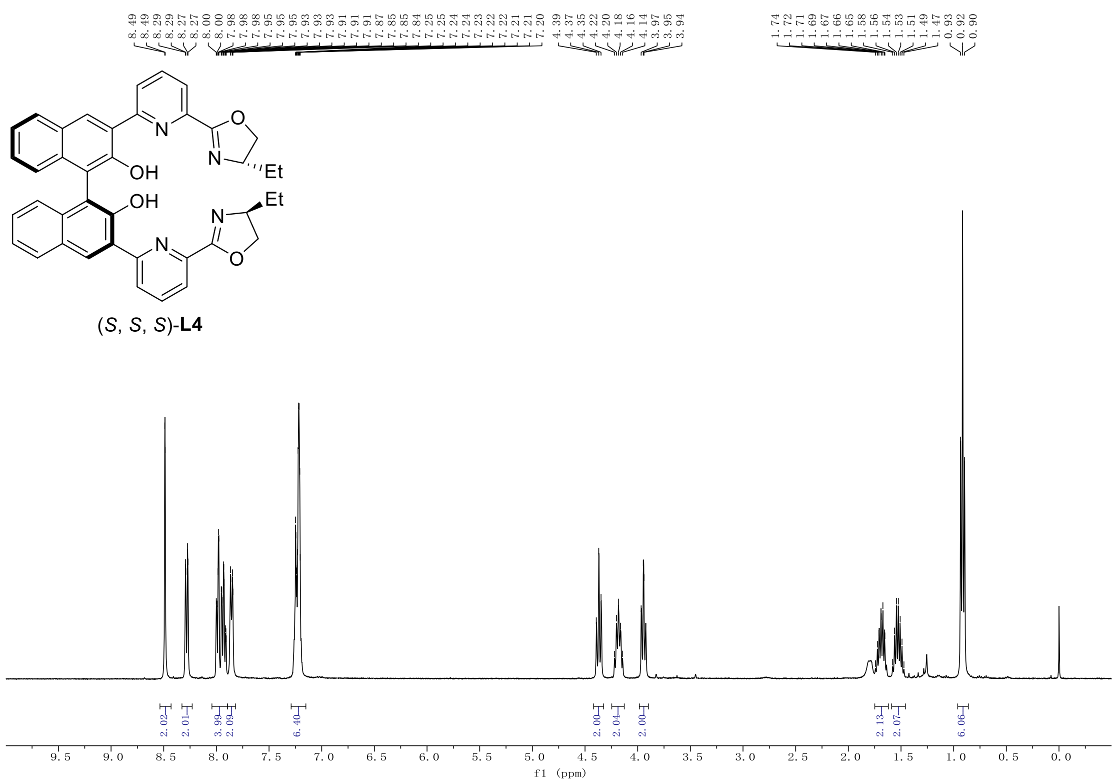

^{1}H NMR spectra (400 MHz, $CDCl_3$) of (*S, S, S*)-**L4**

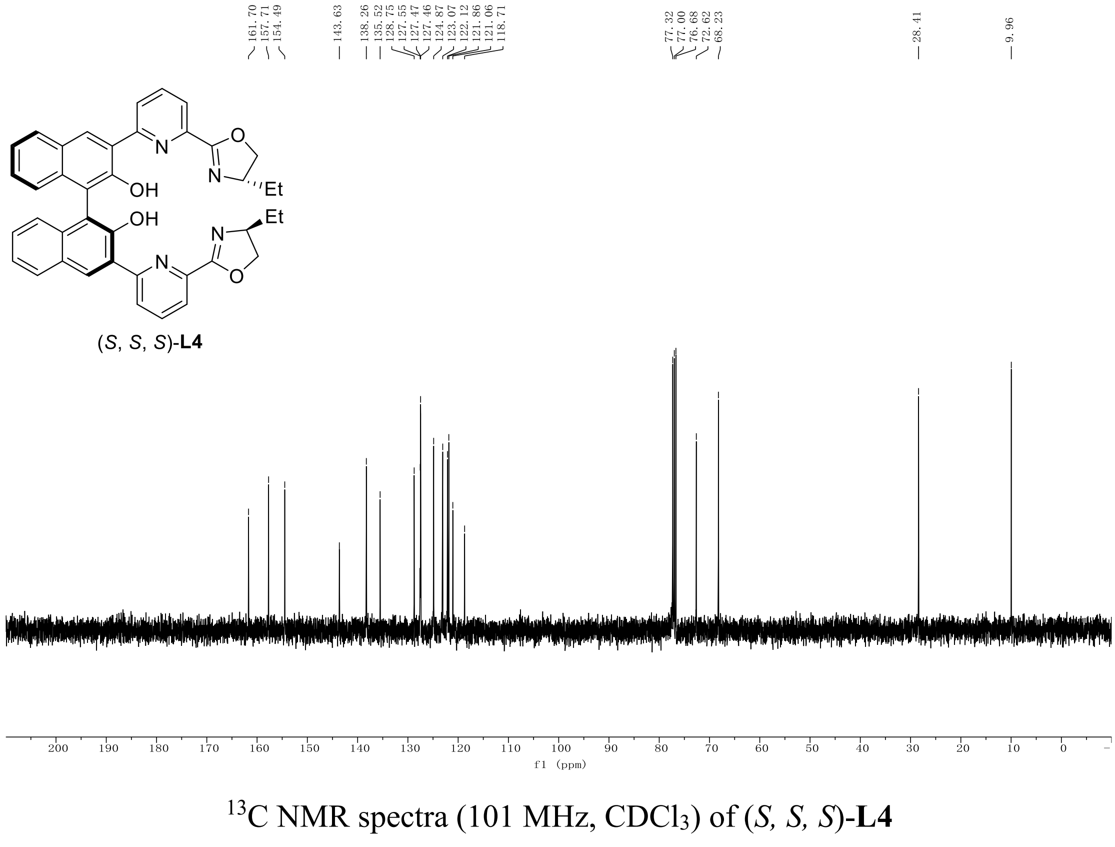

^{13}C NMR spectra (101 MHz, $CDCl_3$) of (*S, S, S*)-**L4**

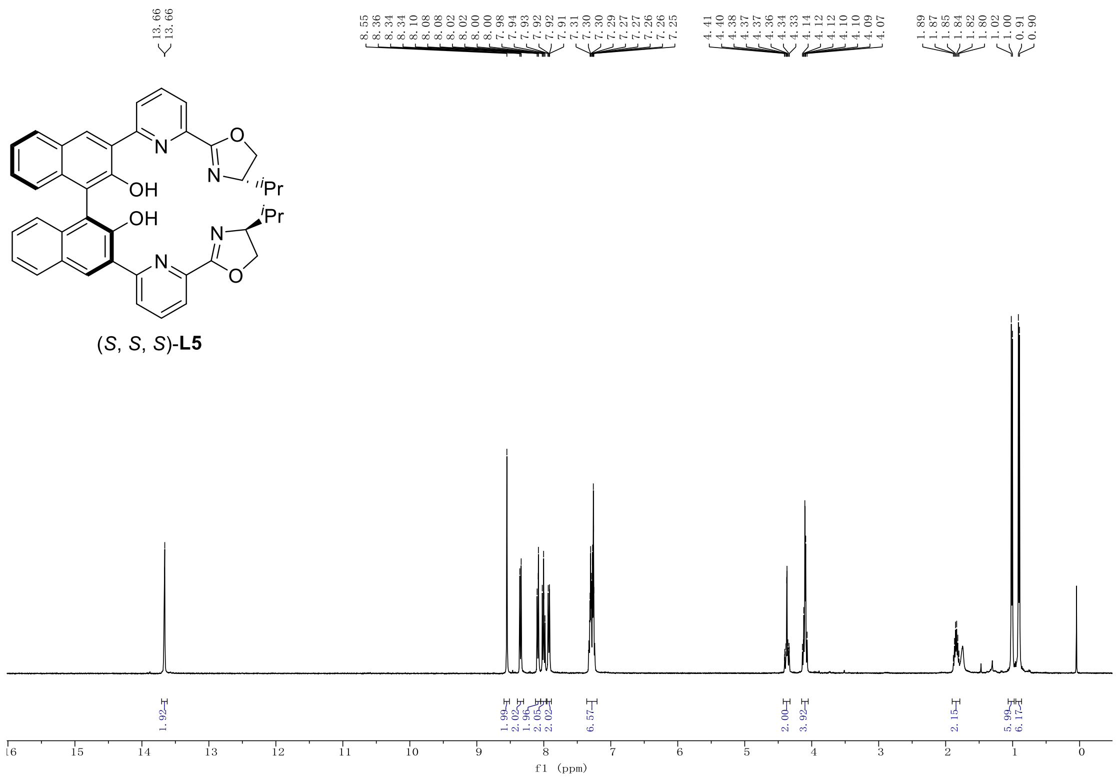

^{1}H NMR spectra (400 MHz, $CDCl_3$) of (*S, S, S*)-**L5**

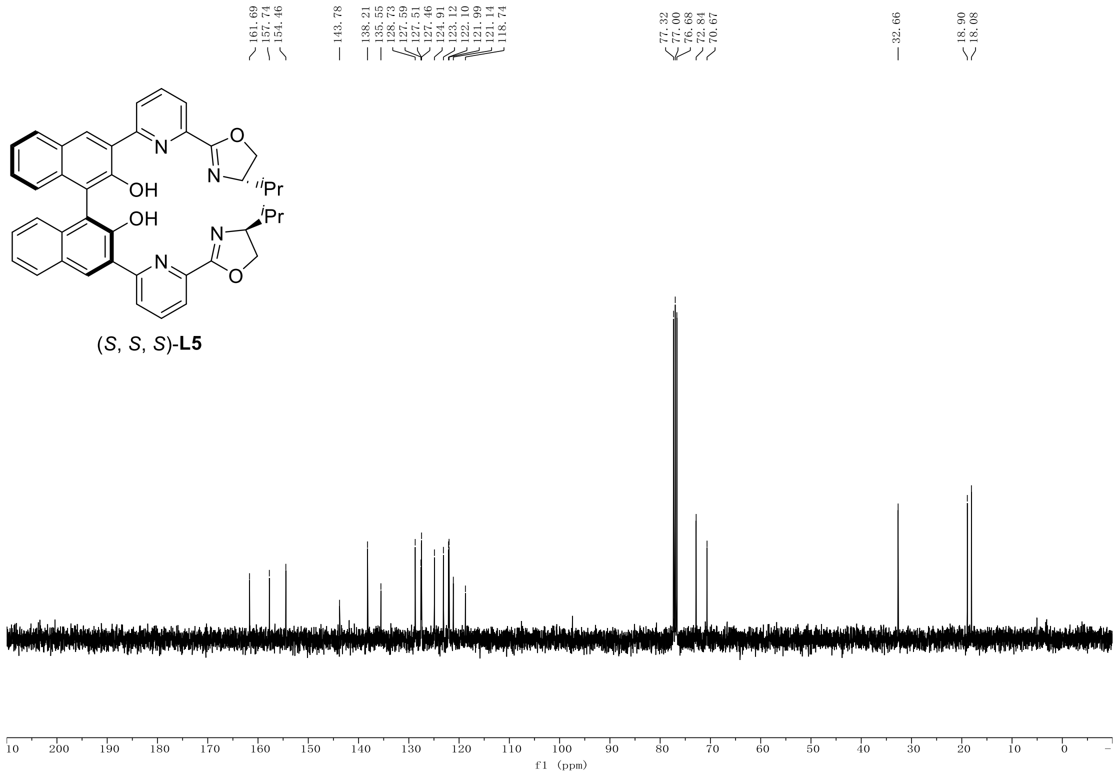

^{13}C NMR spectra (101 MHz, $CDCl_3$) of (*S, S, S*)-**L5**

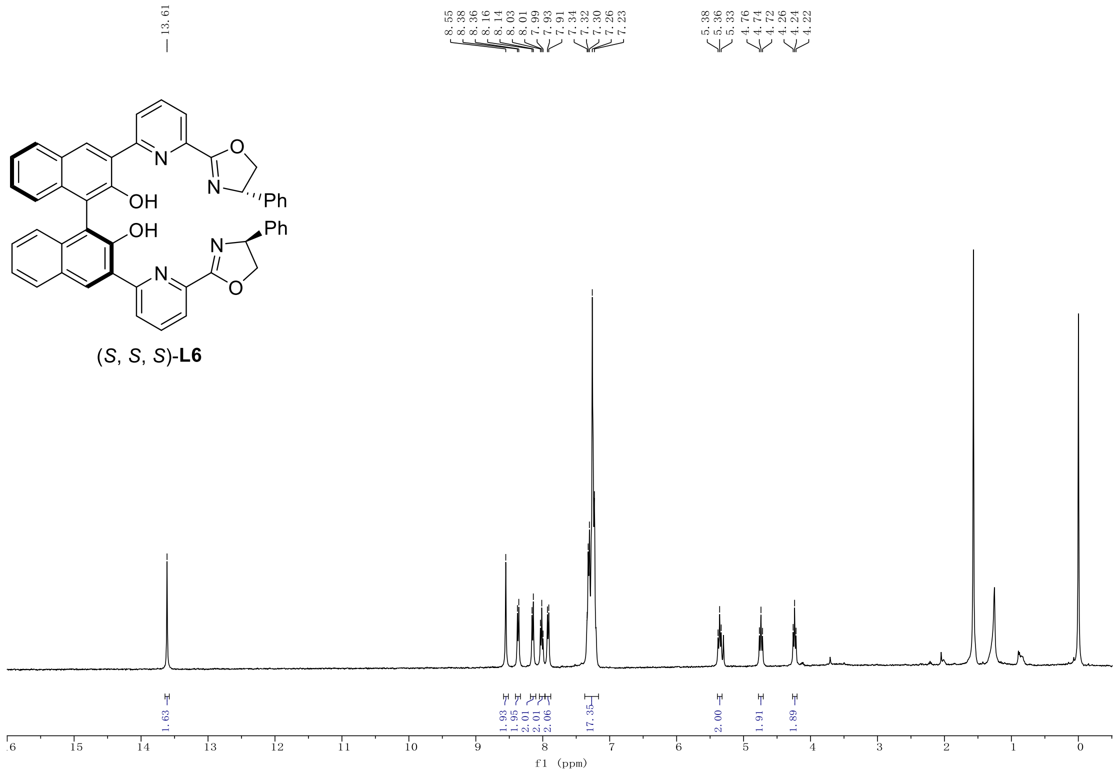

^{1}H NMR spectra (400 MHz, $CDCl_3$) of (*S, S, S*)-**L6**

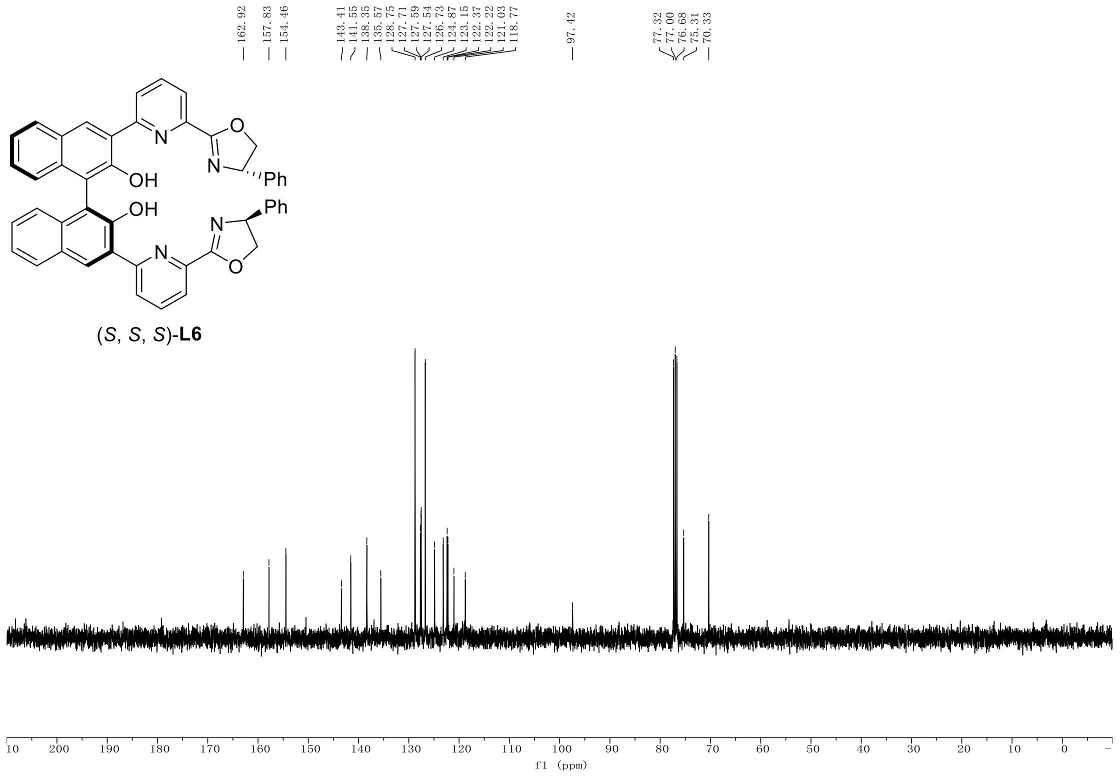

^{13}C NMR spectra (101 MHz, $CDCl_3$) of (*S, S, S*)-**L6**

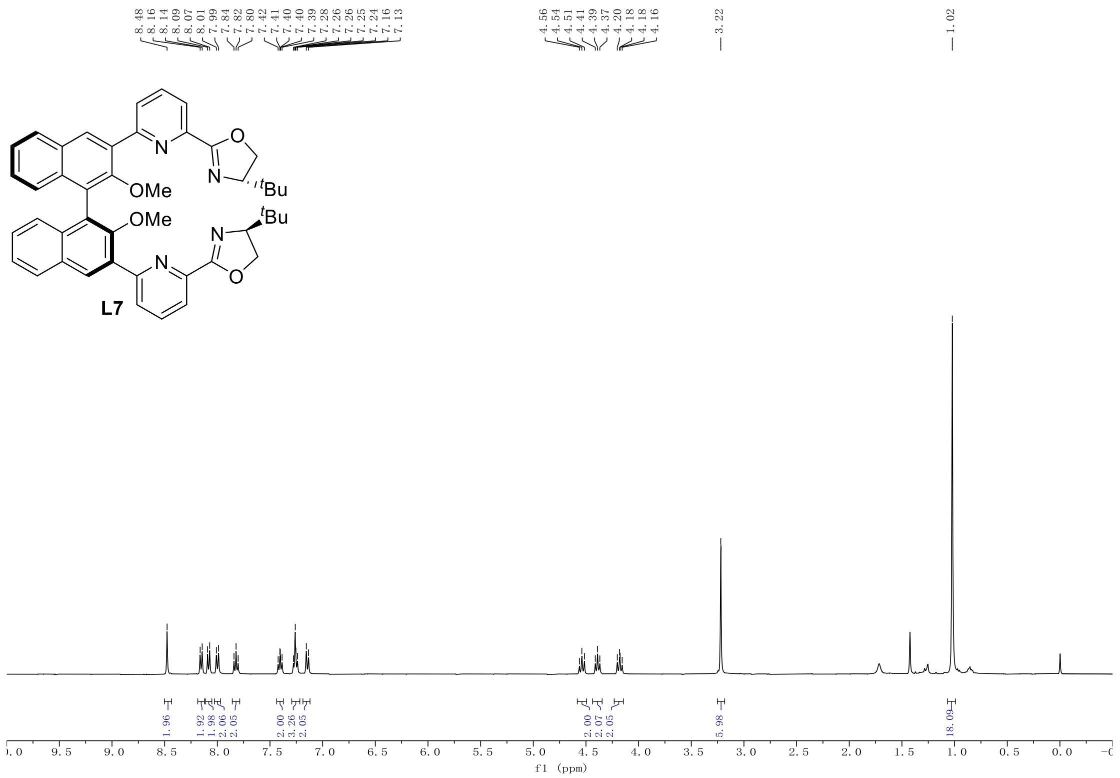

^{1}H NMR spectra (400 MHz, $CDCl_3$) of **L7**

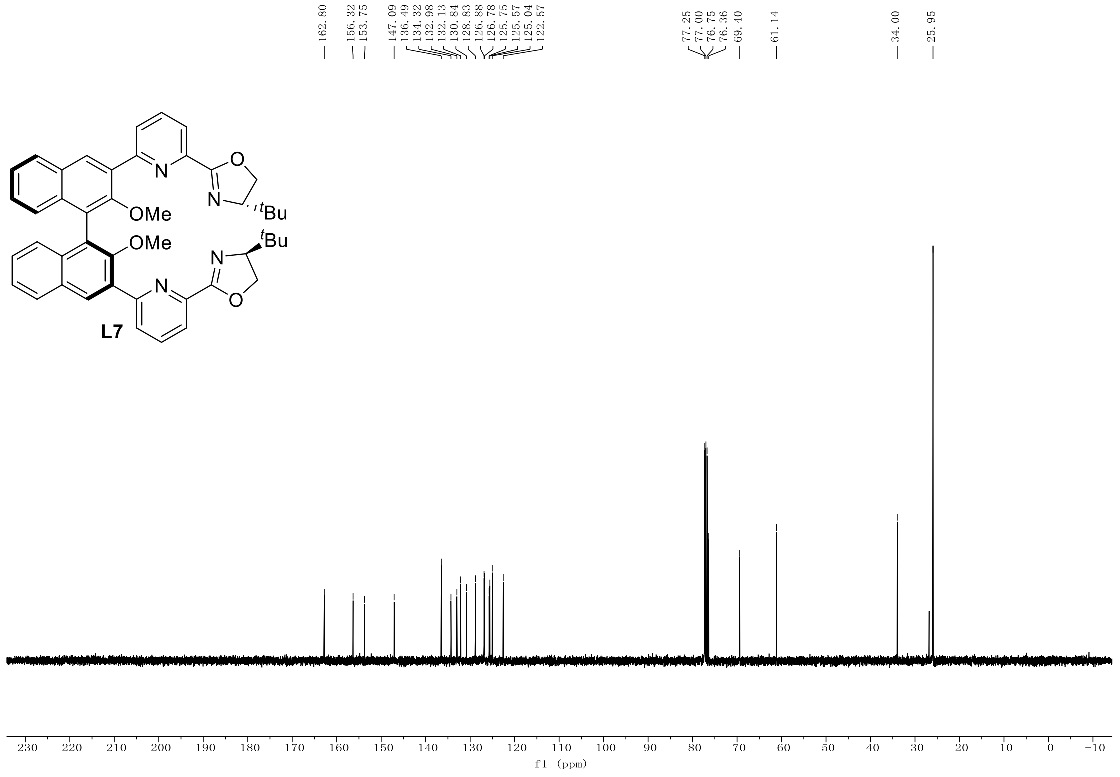

^{13}C NMR spectra (126 MHz, $CDCl_3$) of **L7**

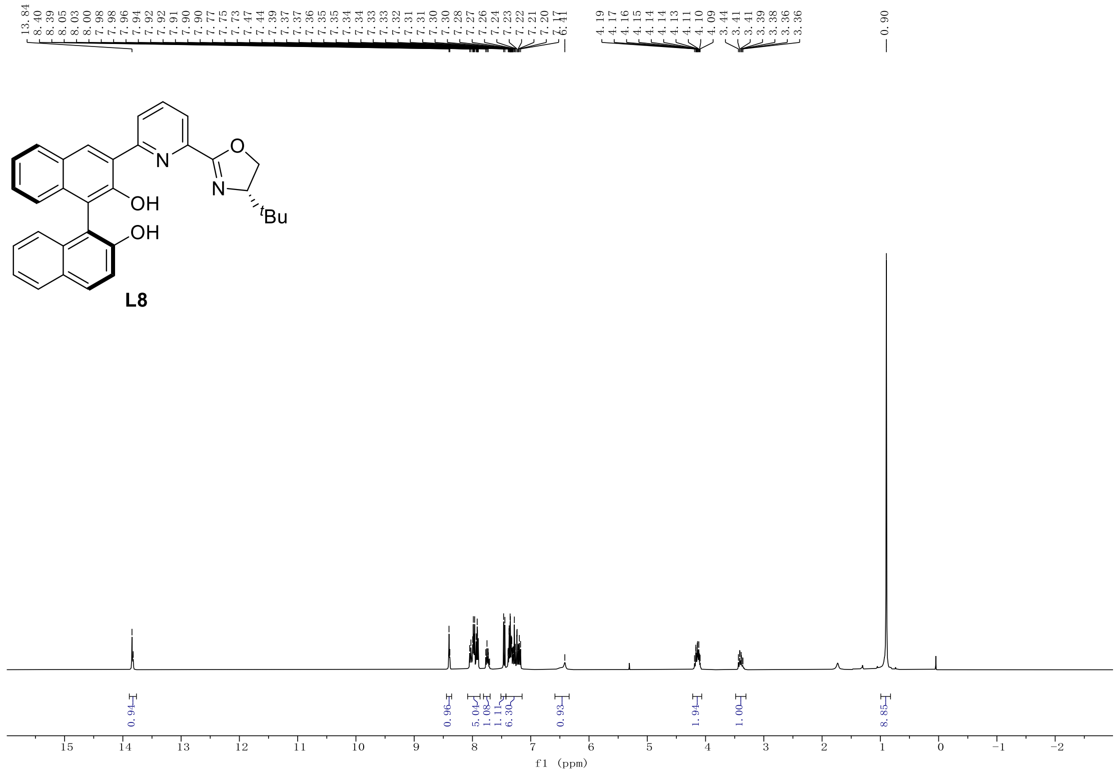

^{1}H NMR spectra (400 MHz, $CDCl_3$) of **L8**

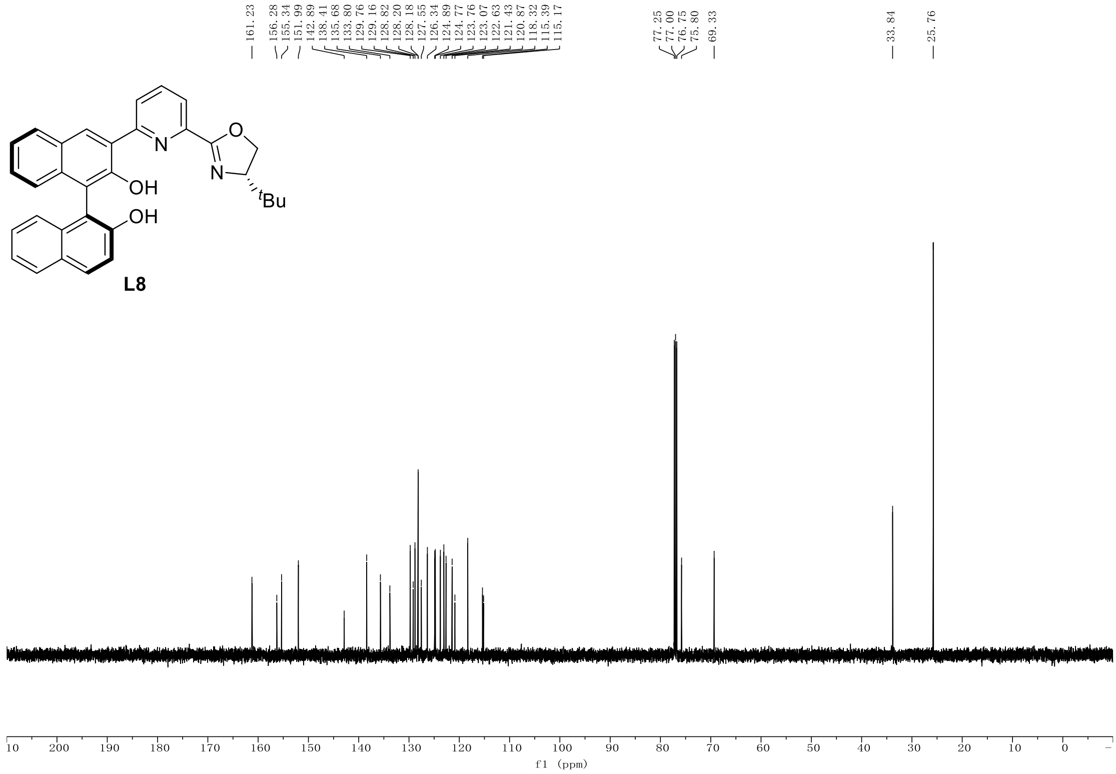

^{13}C NMR spectra (126 MHz, $CDCl_3$) of **L8**

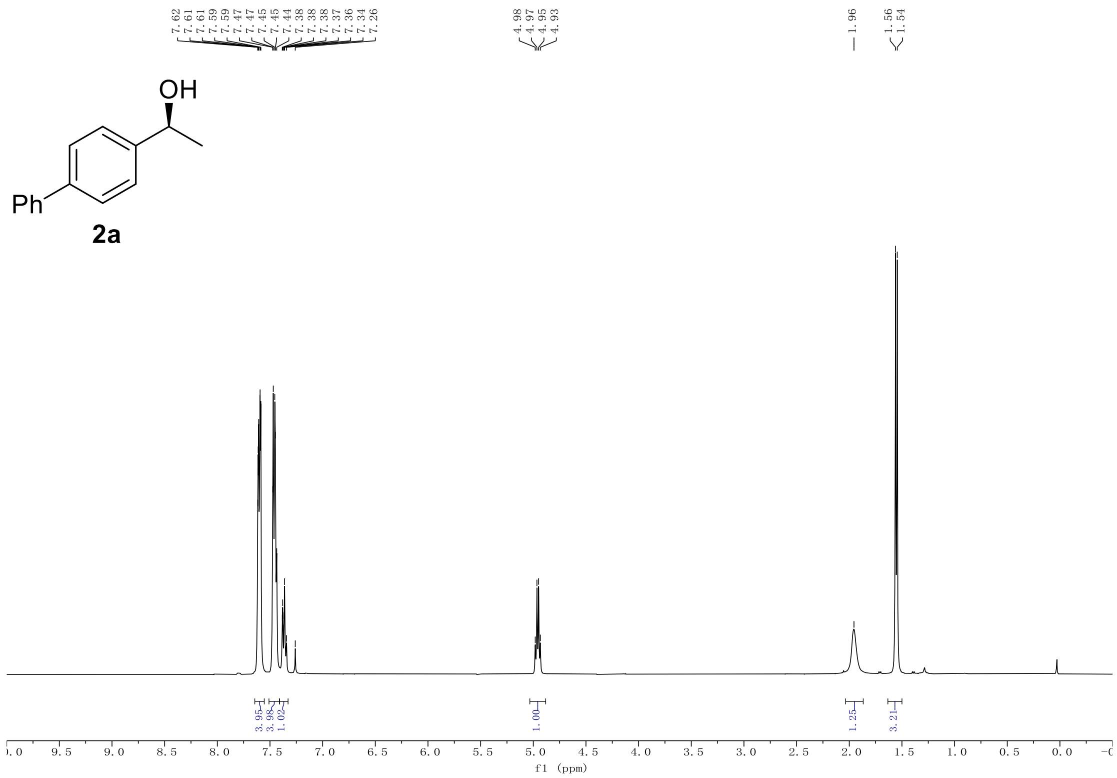

^{1}H NMR spectra (400 MHz, $CDCl_3$) of **2a**

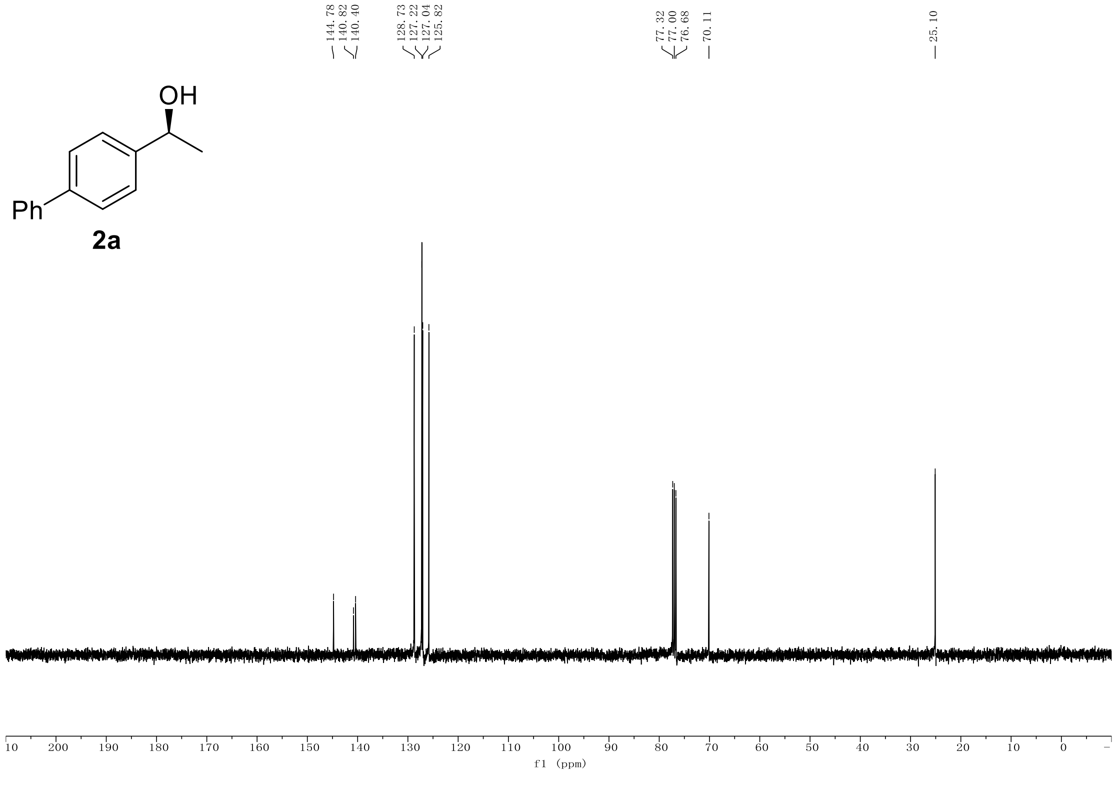

^{13}C NMR spectra (101 MHz, $CDCl_3$) of **2a**

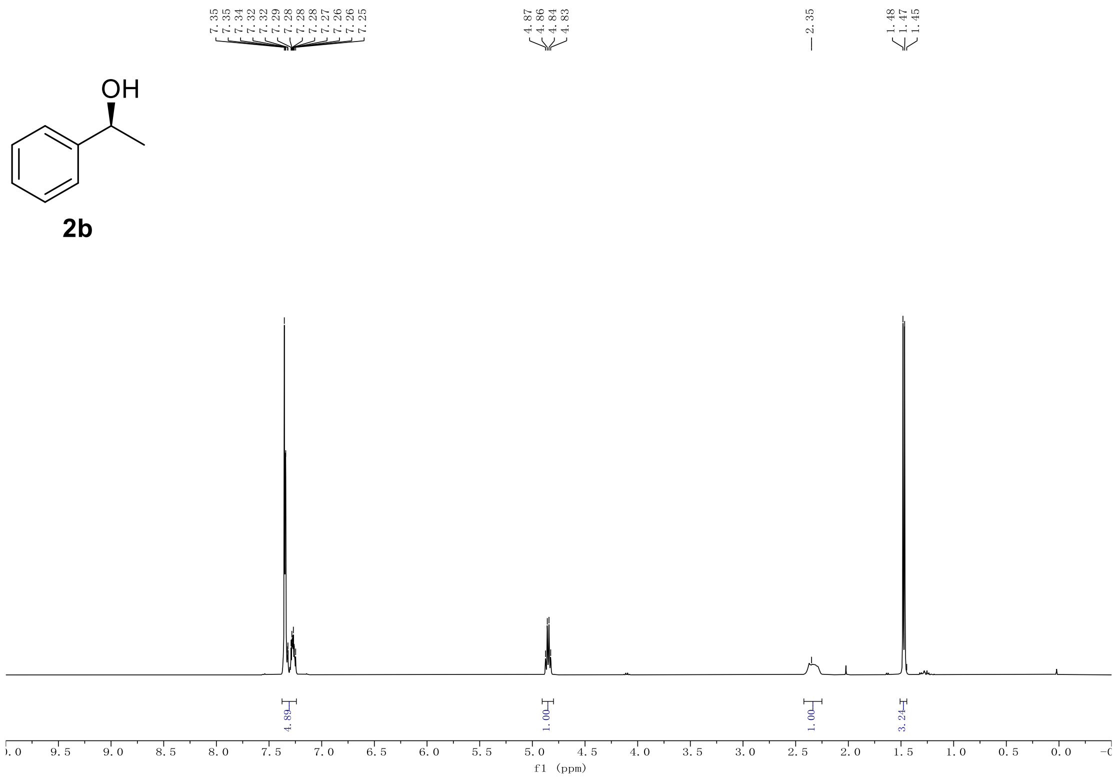

^{1}H NMR spectra (400 MHz, $CDCl_3$) of **2b**

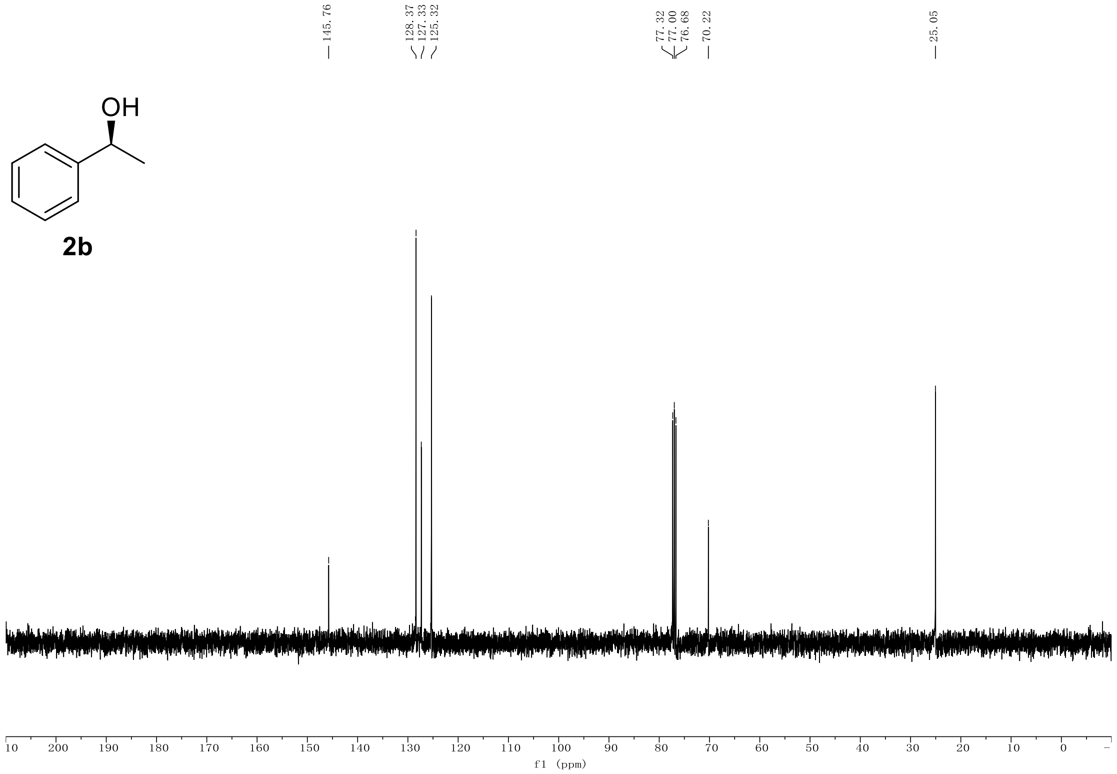

^{13}C NMR spectra (101 MHz, $CDCl_3$) of **2b**

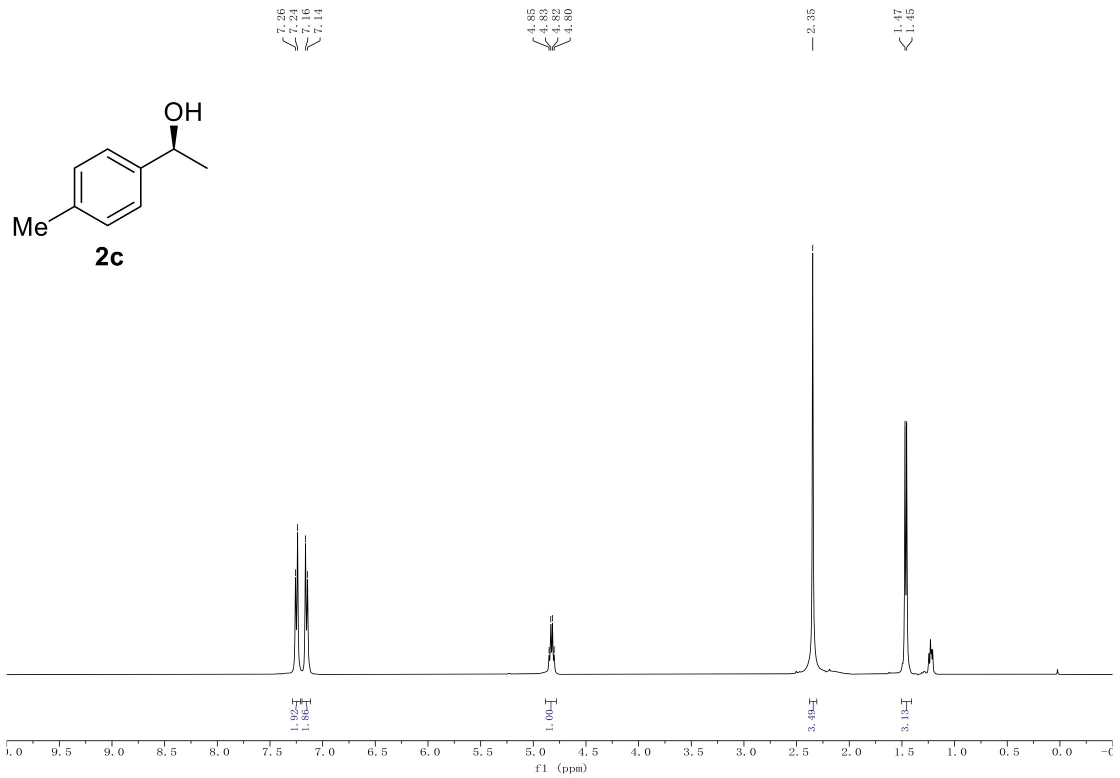

^{1}H NMR spectra (400 MHz, $CDCl_3$) of **2c**

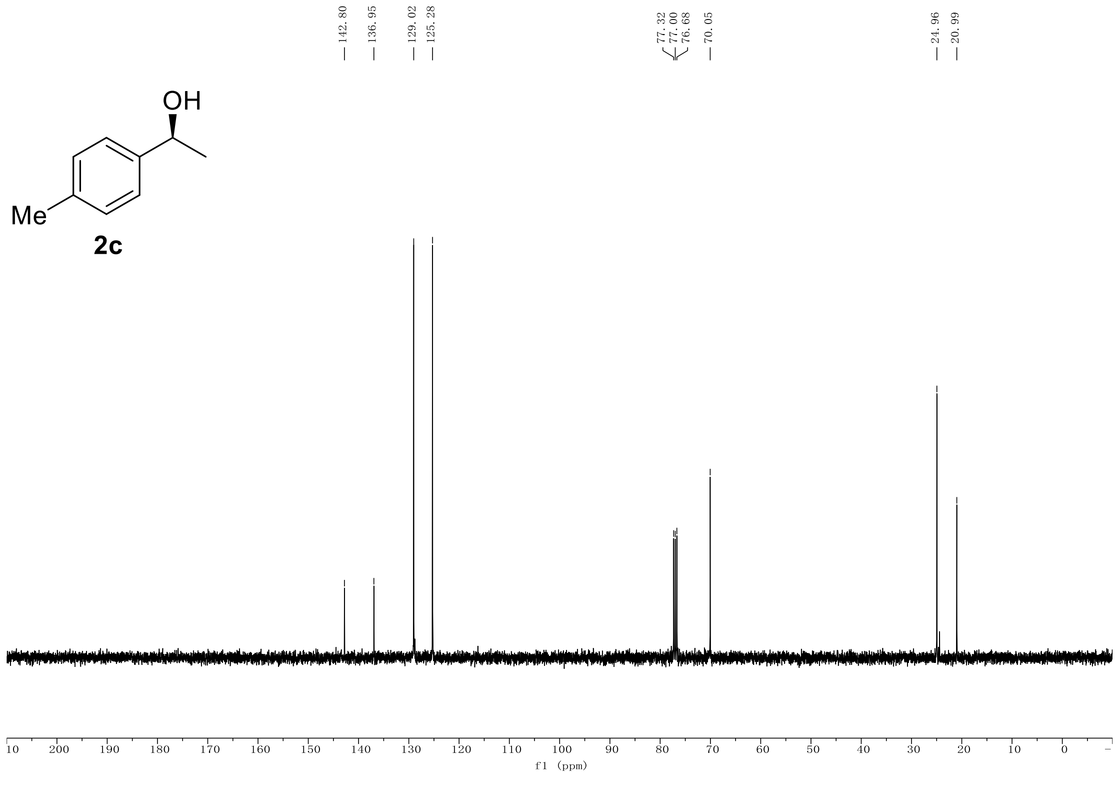

^{13}C NMR spectra (101 MHz, $CDCl_3$) of **2c**

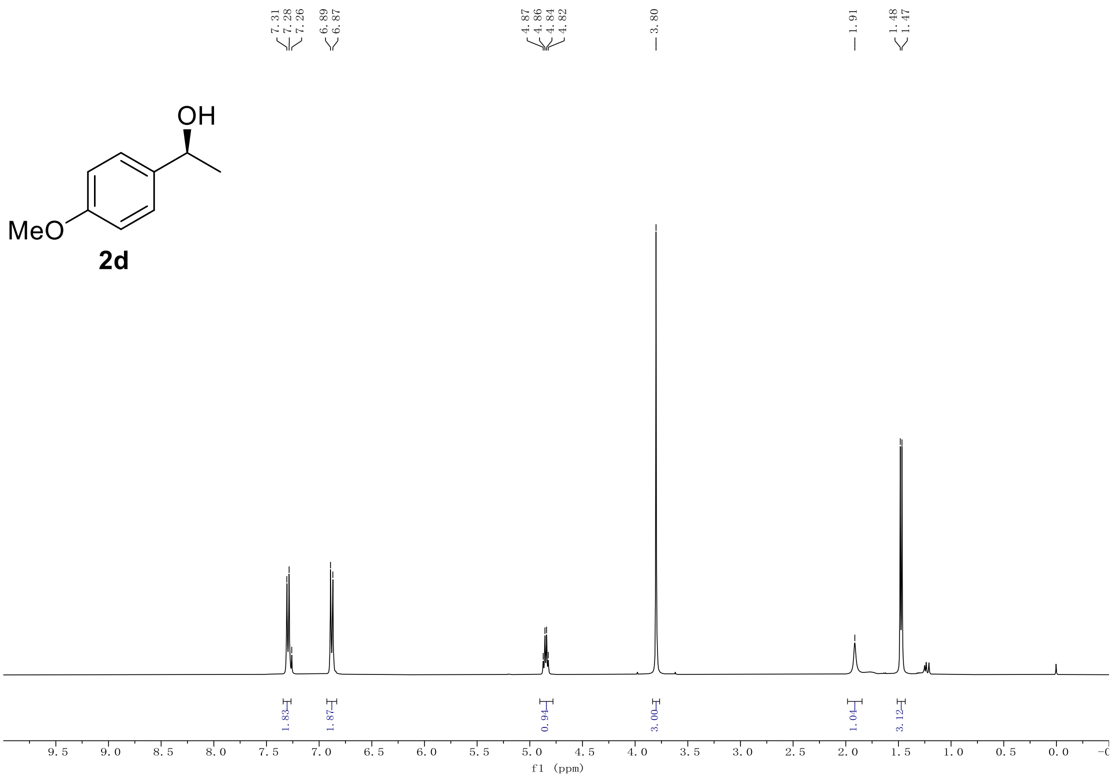

^{1}H NMR spectra (400 MHz, $CDCl_3$) of **2d**

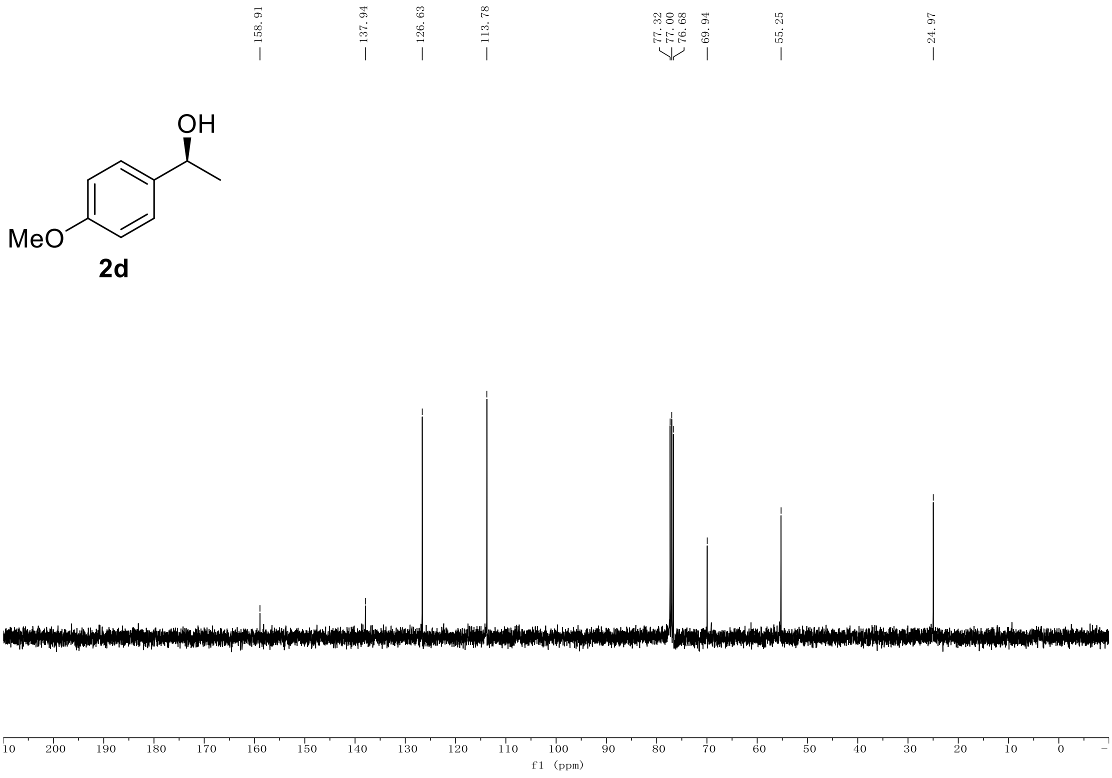

^{13}C NMR spectra (101 MHz, $CDCl_3$) of **2d**

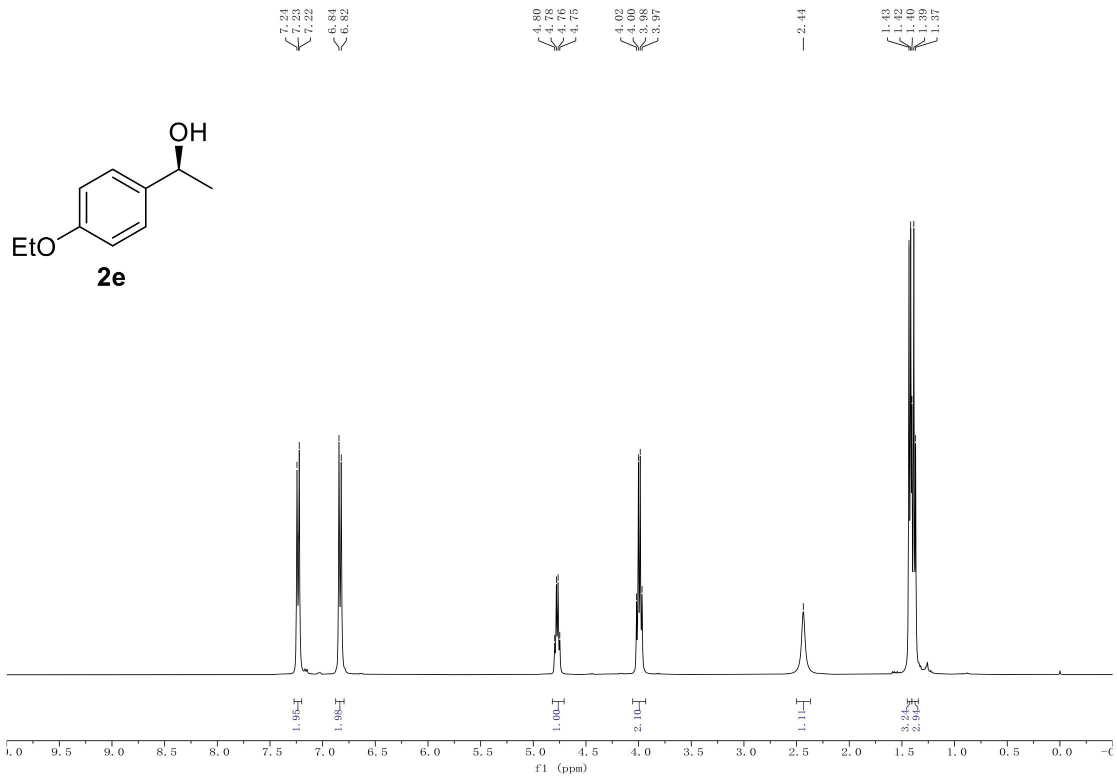

^{1}H NMR spectra (400 MHz, $CDCl_3$) of **2e**

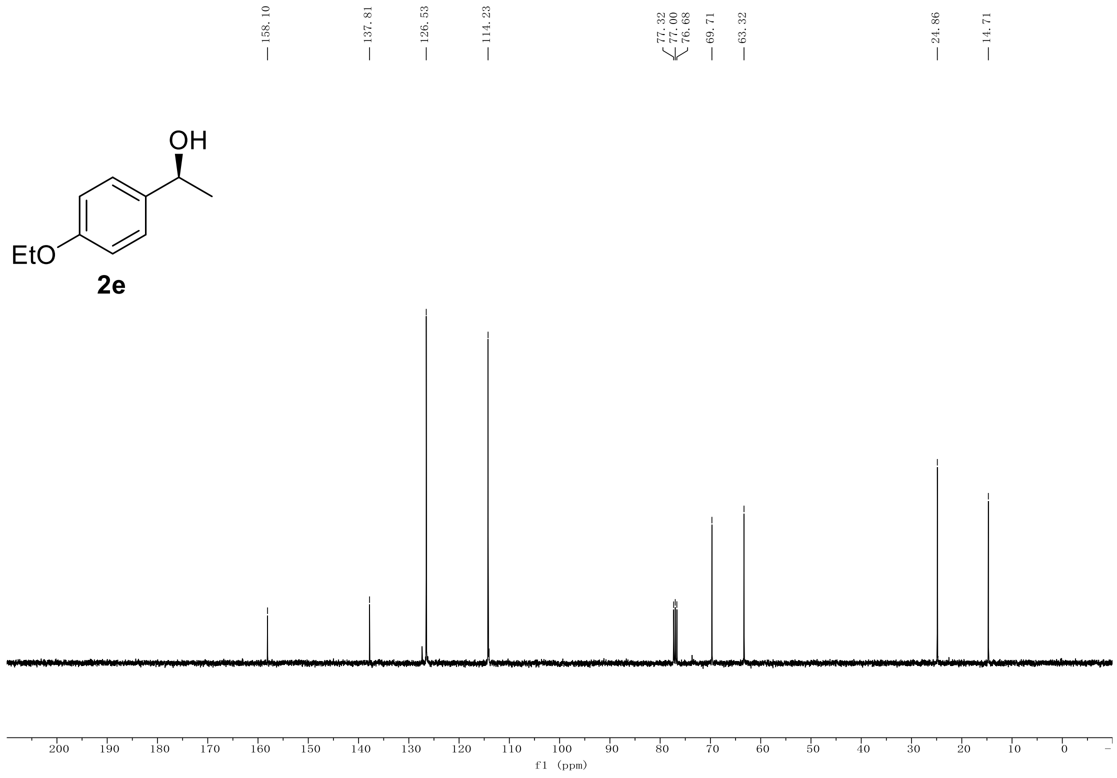

^{13}C NMR spectra (101 MHz, $CDCl_3$) of **2e**

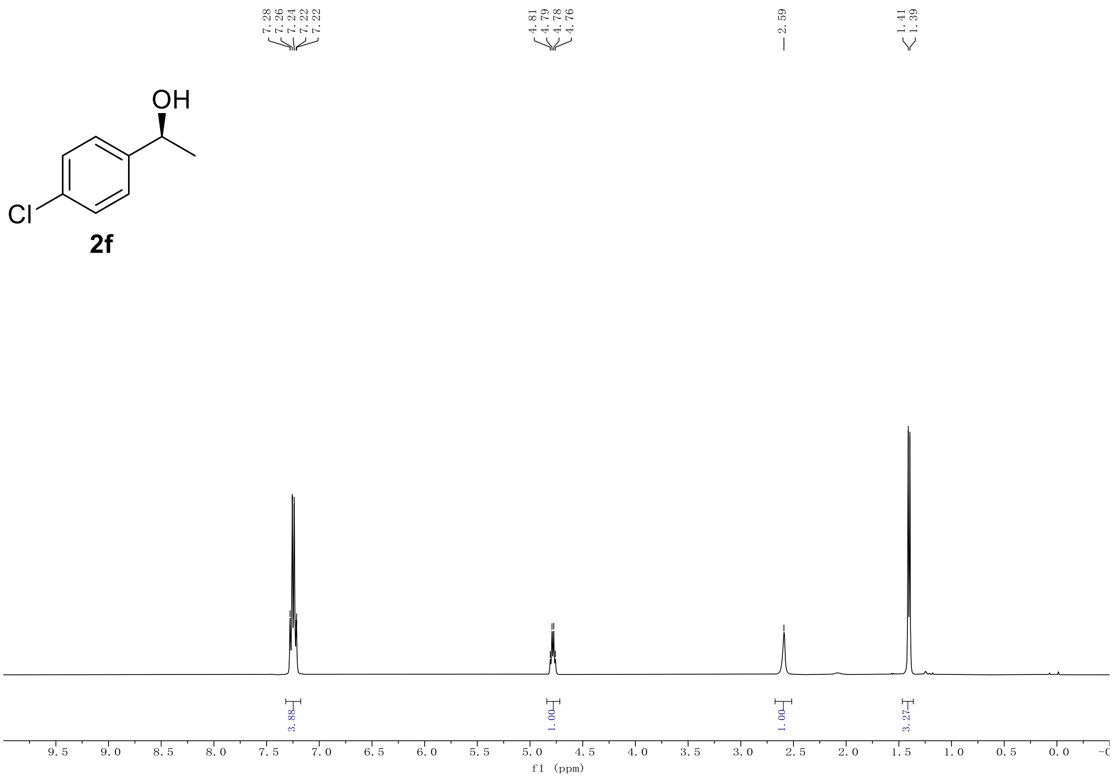

^{1}H NMR spectra (400 MHz, $CDCl_3$) of **2f**

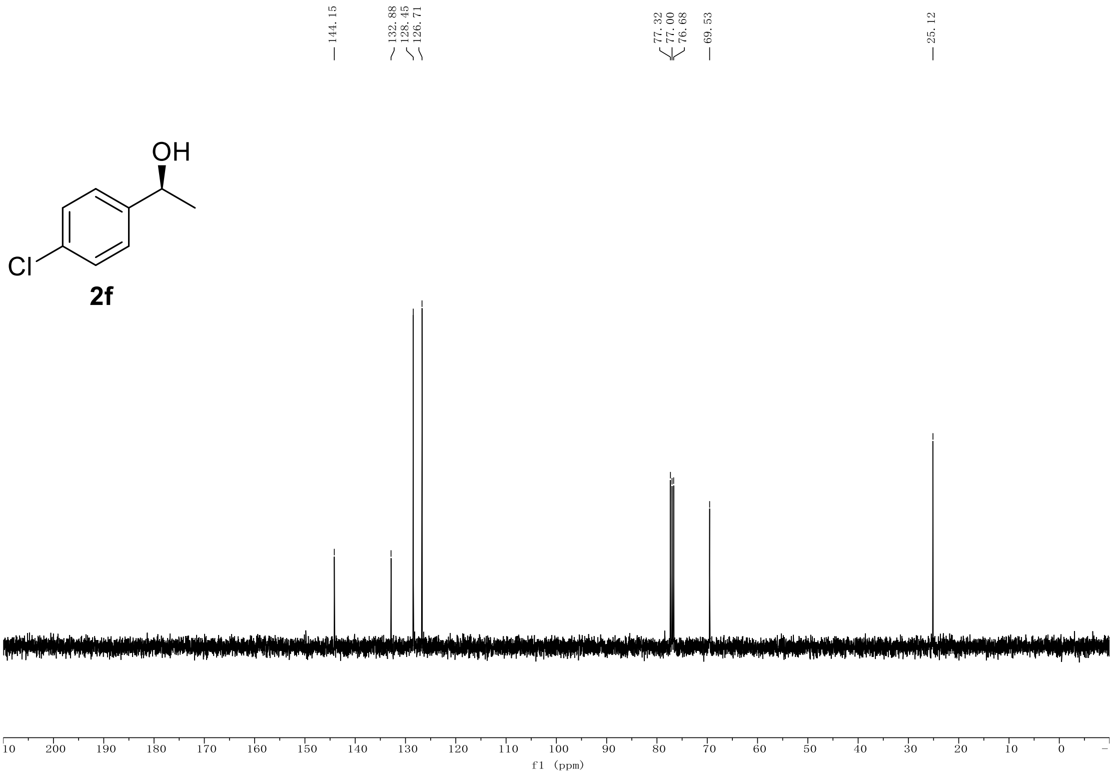

^{13}C NMR spectra (400 MHz, $CDCl_3$) of **2f**

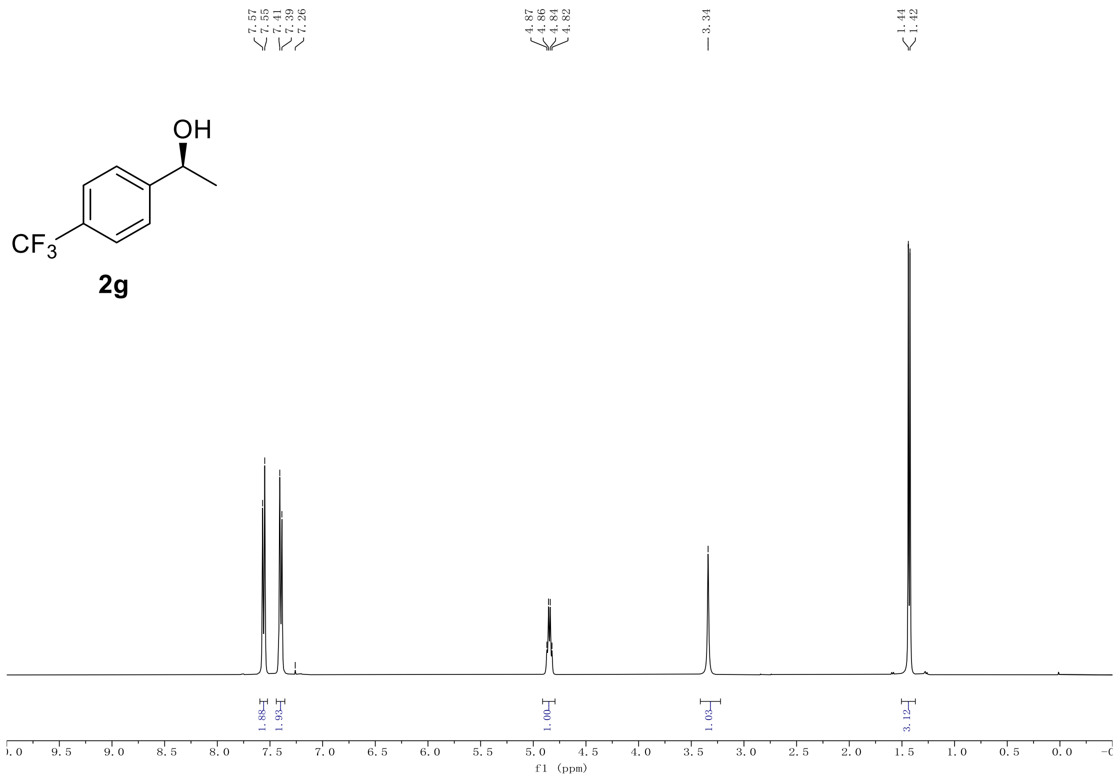

^{1}H NMR spectra (400 MHz, $CDCl_3$) of **2g**

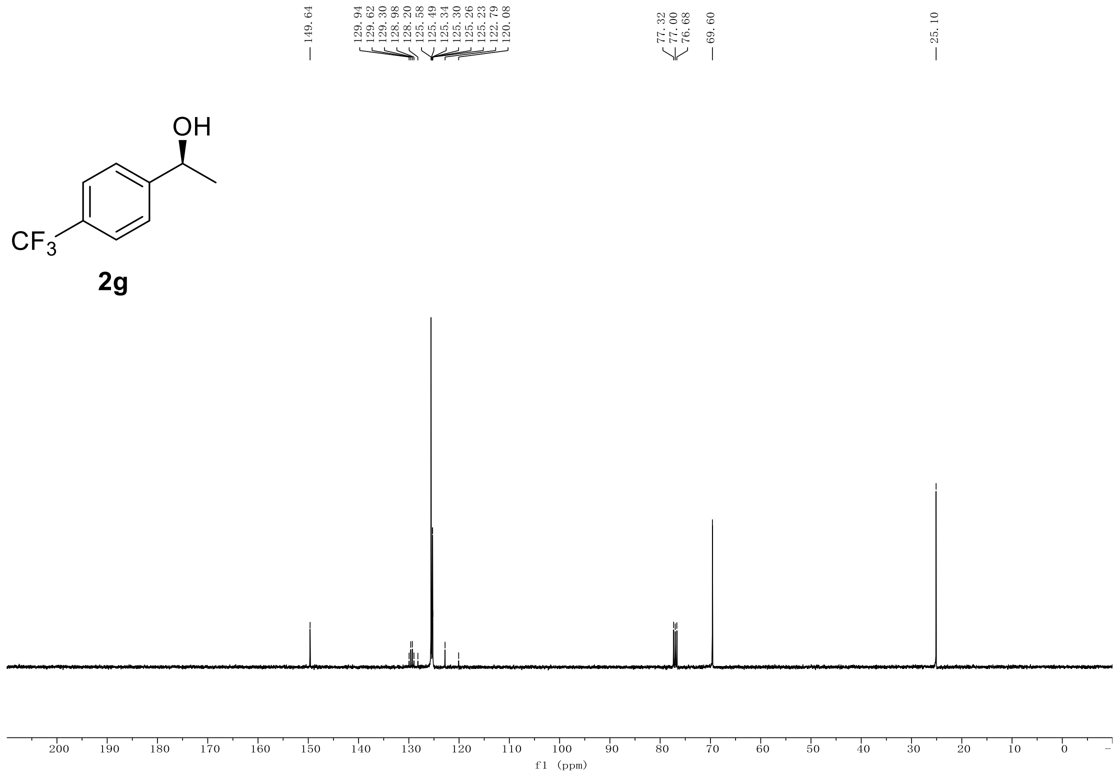

^{13}C NMR spectra (101 MHz, $CDCl_3$) of **2g**

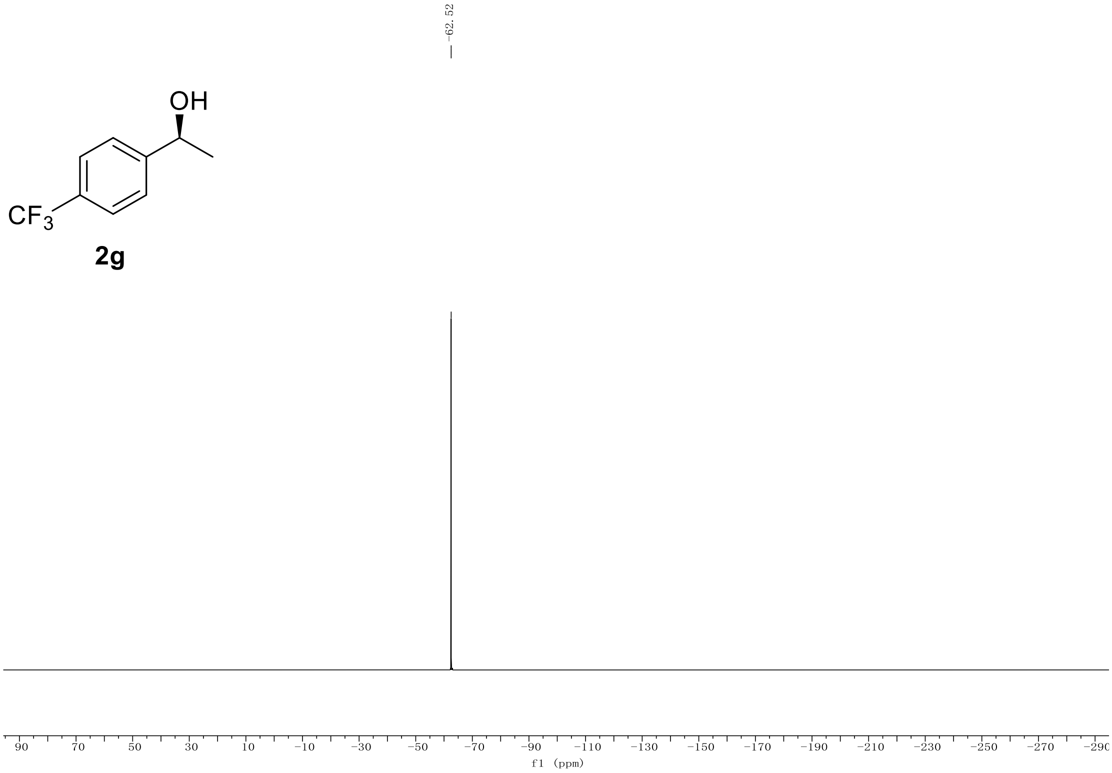

^{19}F NMR spectra (376 MHz, $CDCl_3$) of **2g**

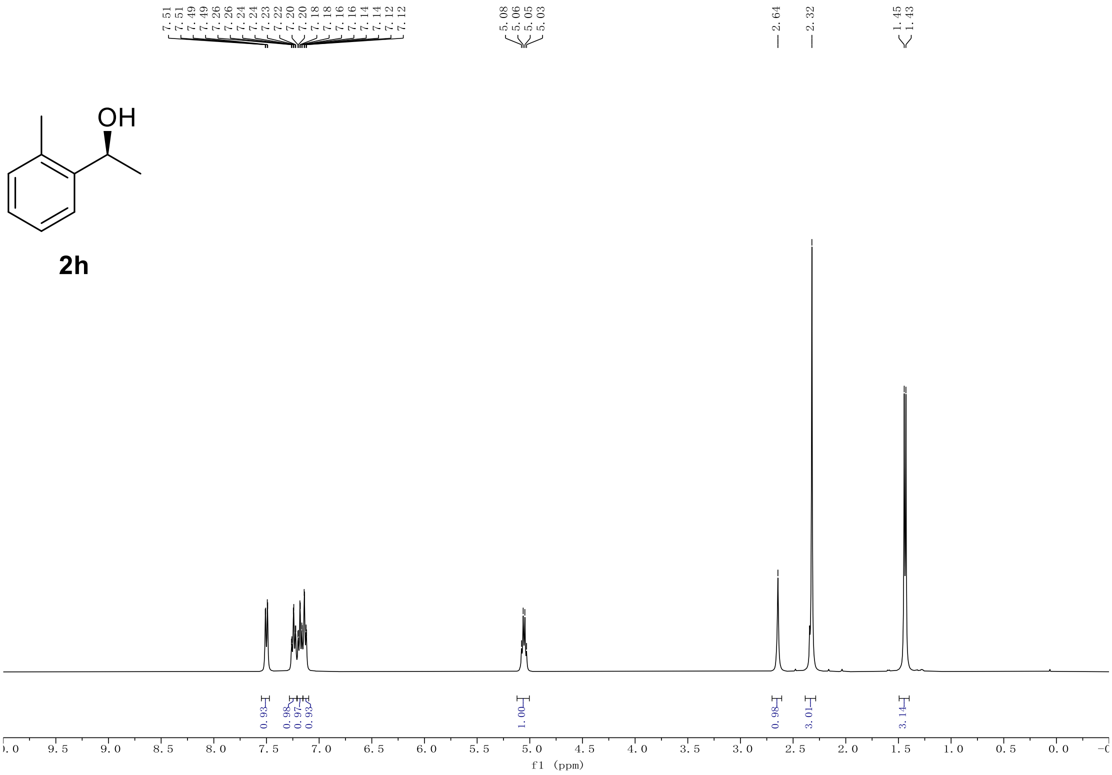

^{1}H NMR spectra (400 MHz, $CDCl_3$) of **2h**

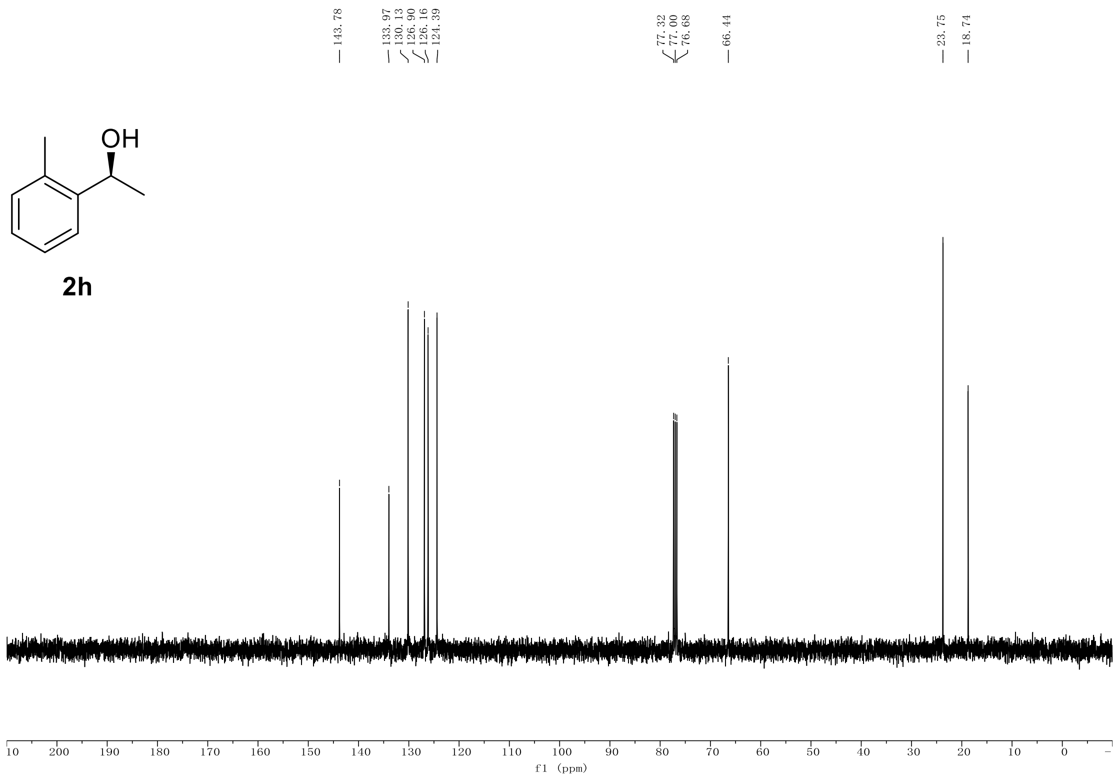

^{13}C NMR spectra (101 MHz, $CDCl_3$) of **2h**

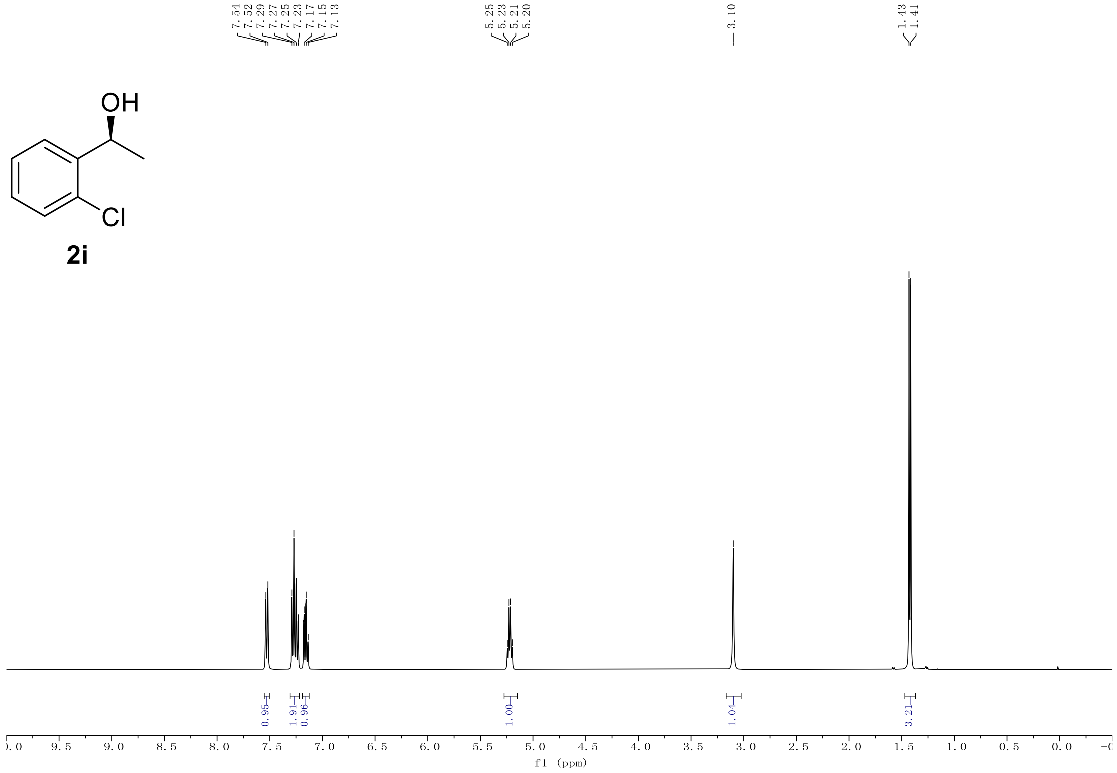

^{1}H NMR spectra (400 MHz, $CDCl_3$) of **2i**

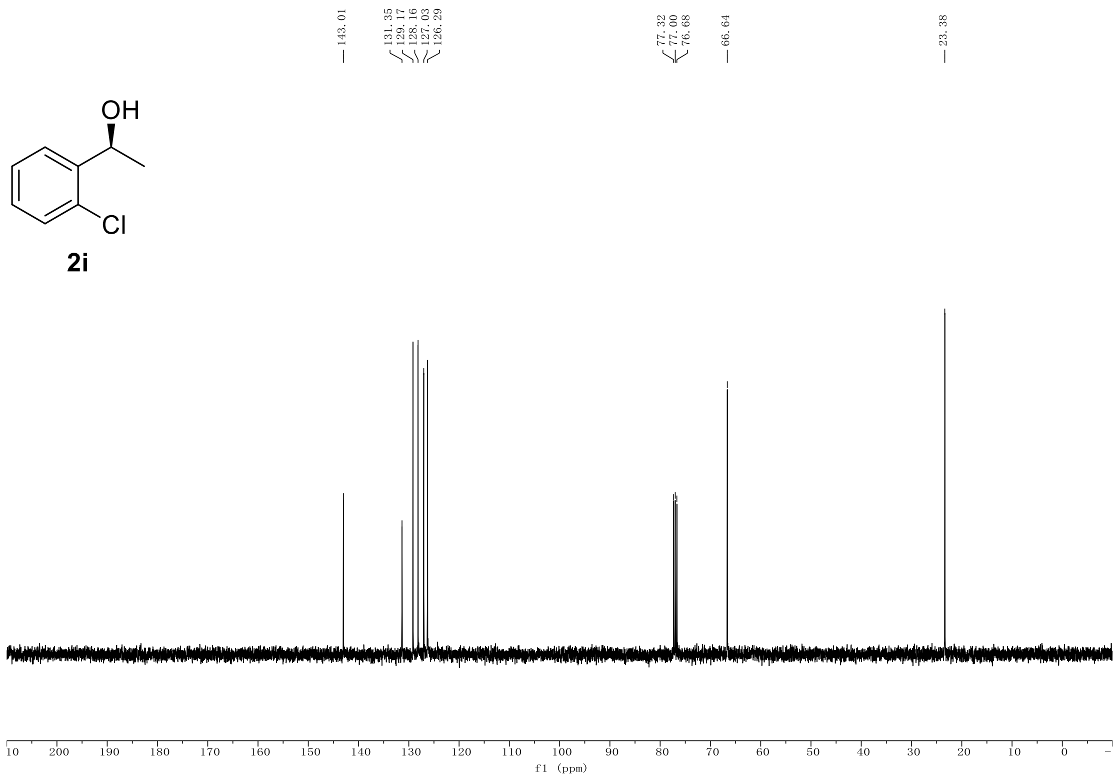

^{13}C NMR spectra (101 MHz, $CDCl_3$) of **2i**

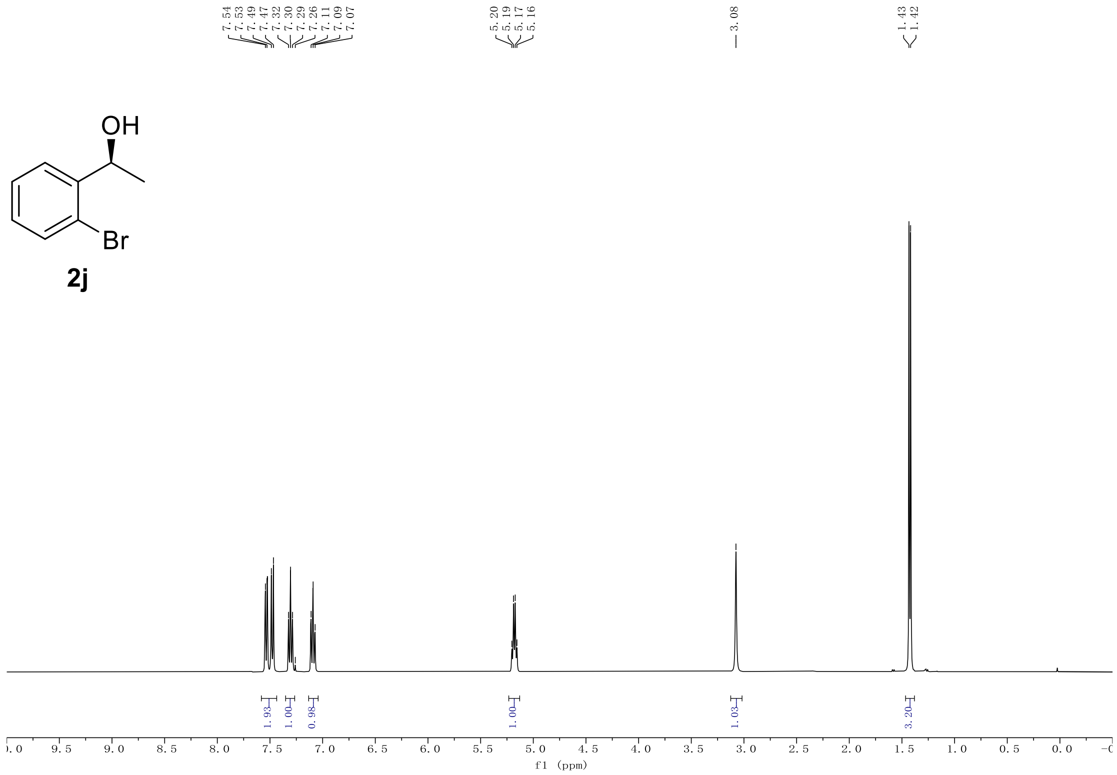

^{1}H NMR spectra (400 MHz, $CDCl_3$) of **2j**

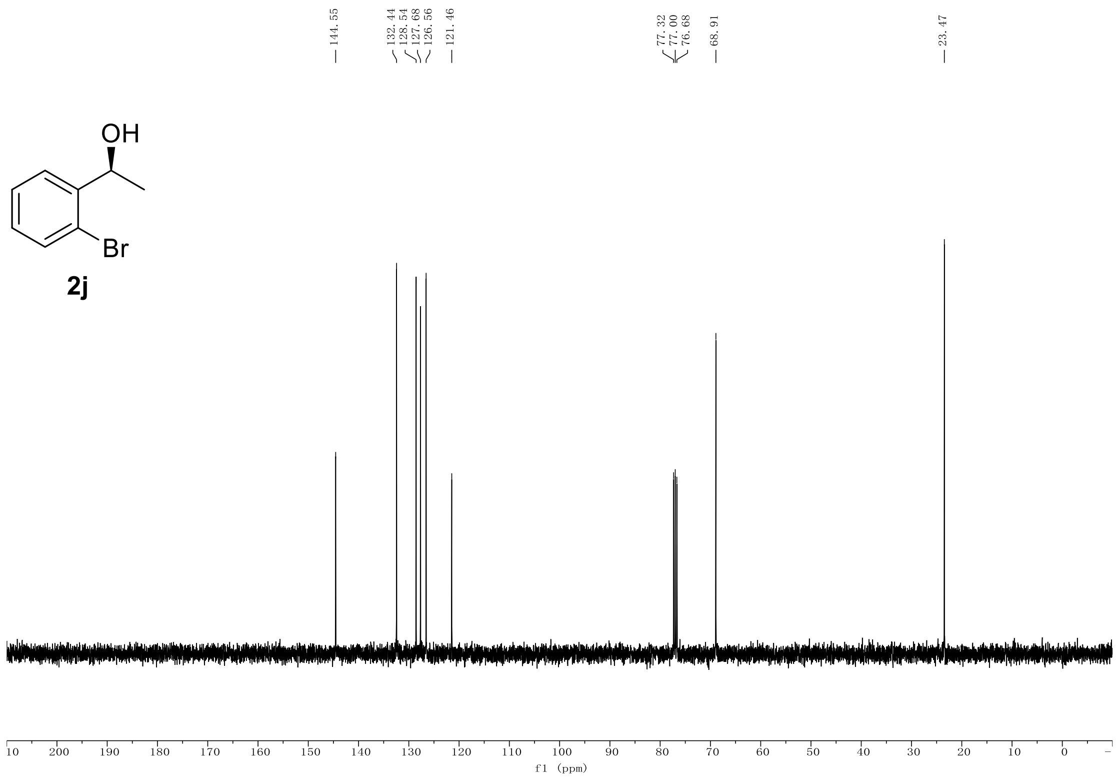

^{13}C NMR spectra (101 MHz, $CDCl_3$) of **2j**

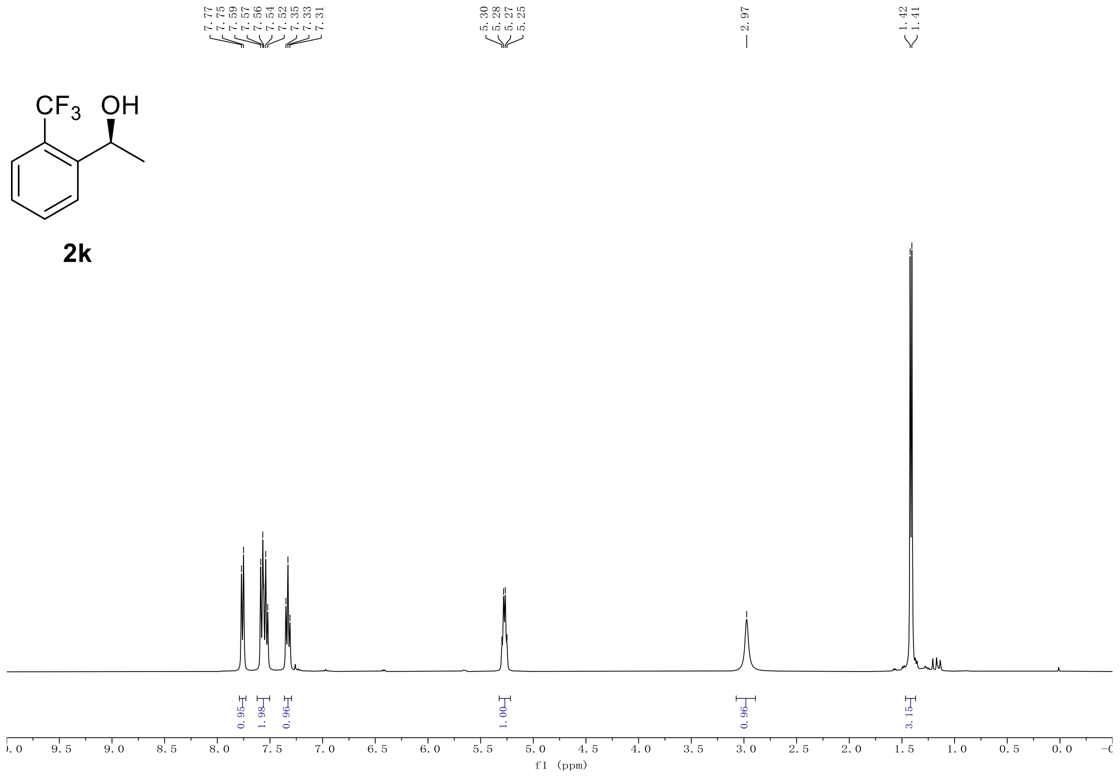

^{1}H NMR spectra (400 MHz, $CDCl_3$) of **2k**

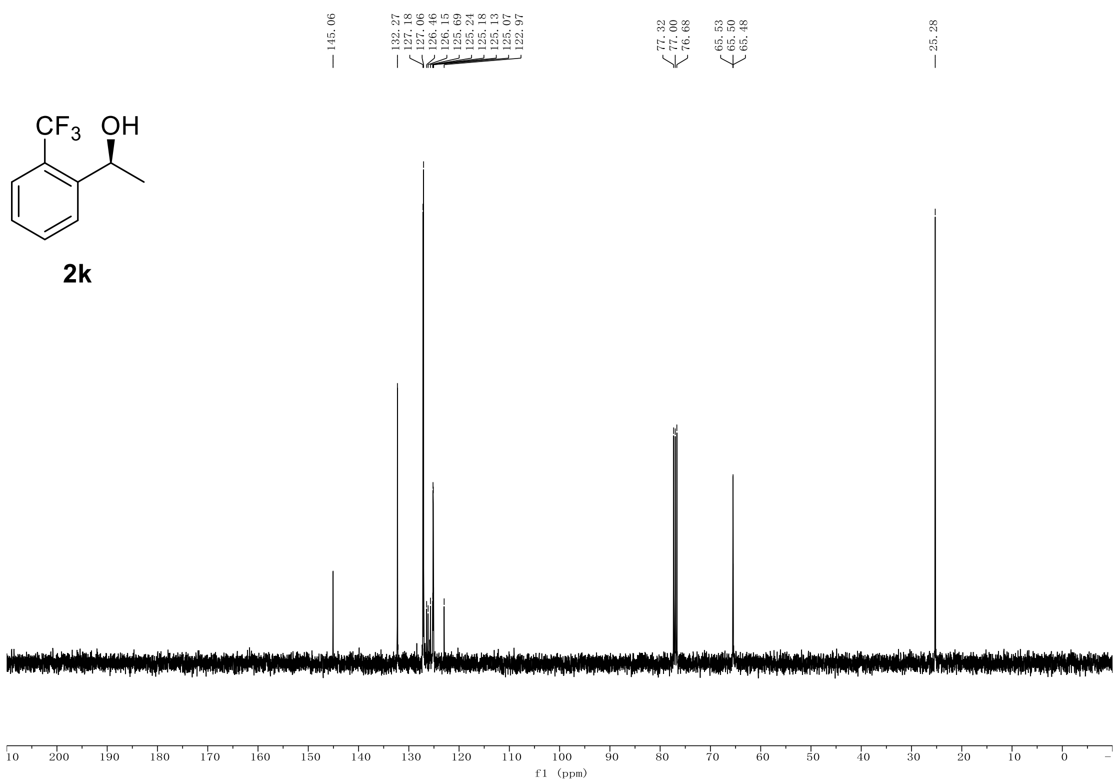

^{13}C NMR spectra (101 MHz, $CDCl_3$) of **2k**

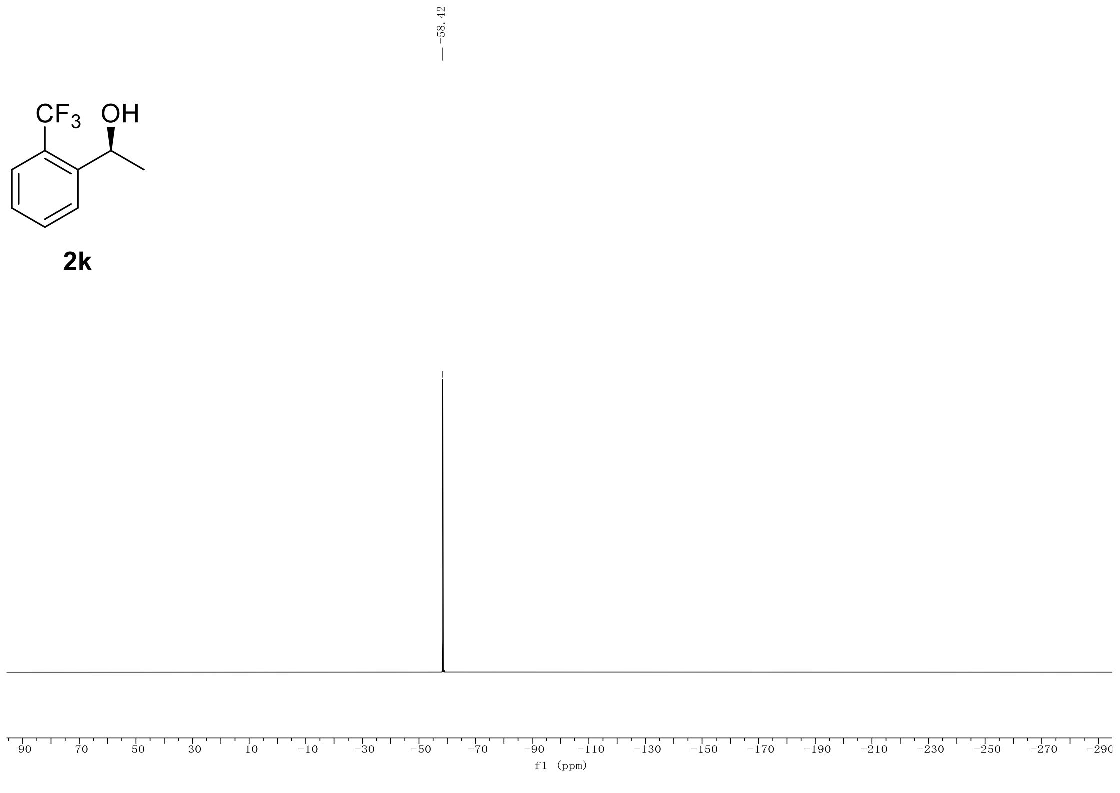

^{19}F NMR spectra (376 MHz, $CDCl_3$) of **2k**

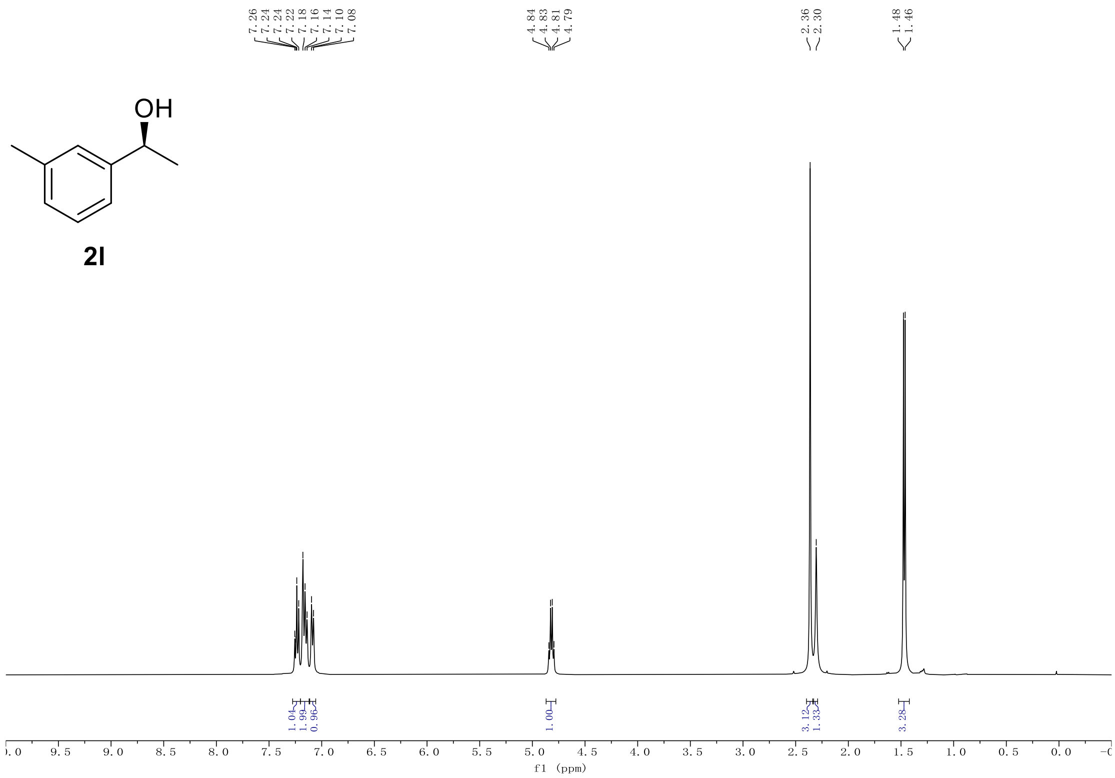

^{1}H NMR spectra (400 MHz, CDCl$_3$) of **2l**

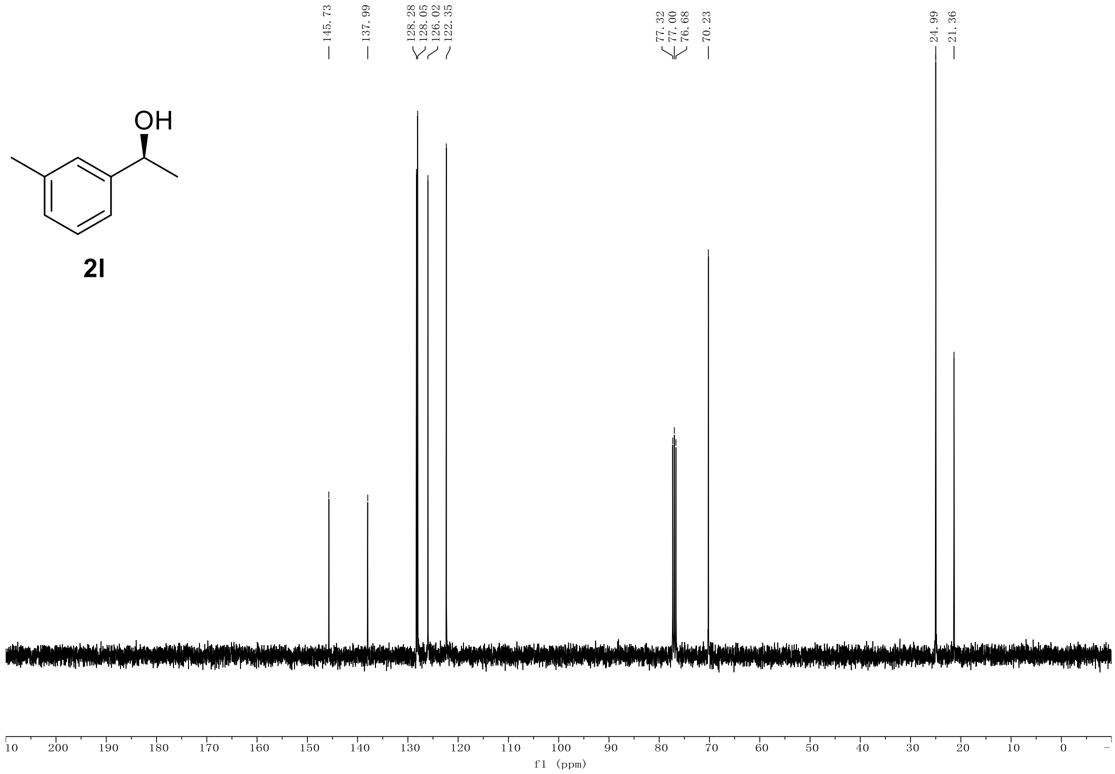

^{13}C NMR spectra (101 MHz, CDCl$_3$) of **2l**

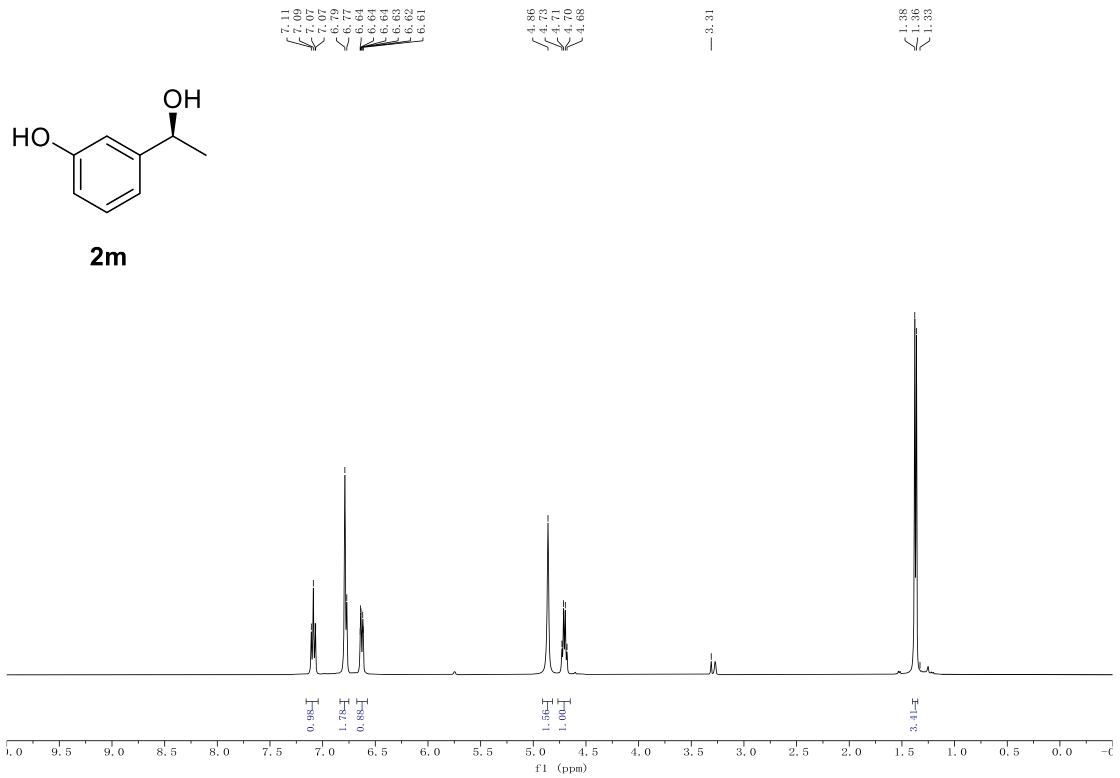

^{1}H NMR spectra (400 MHz, CD_3OD) of **2m**

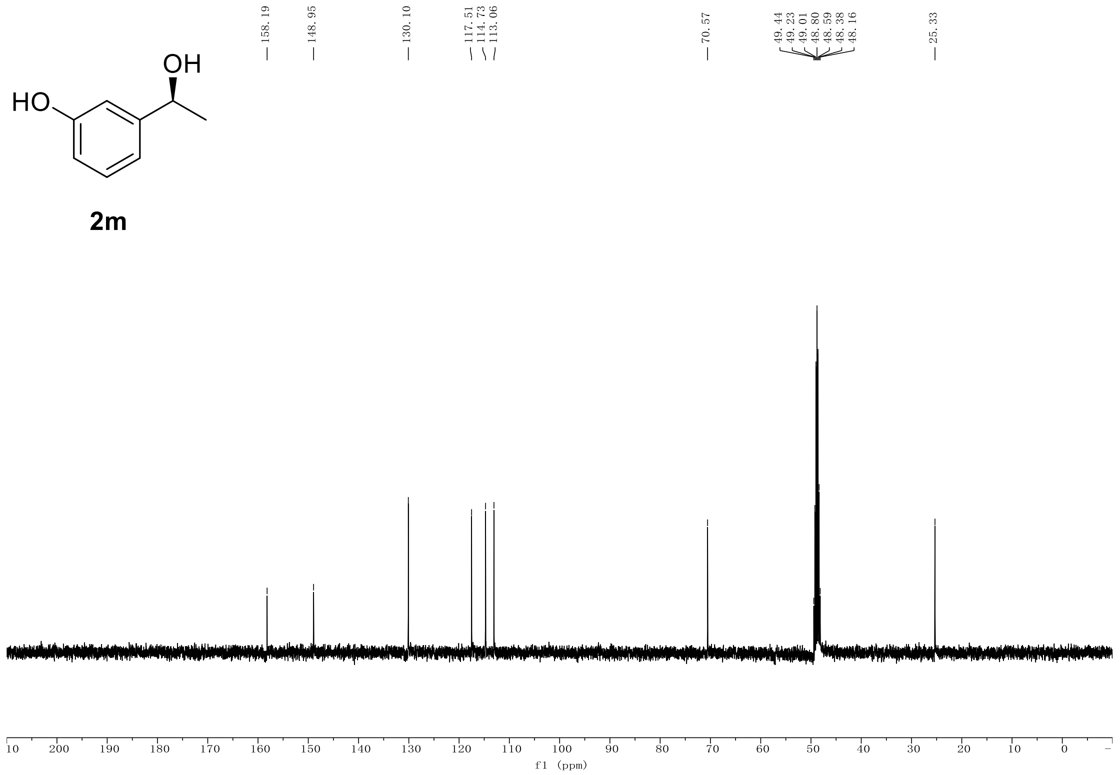

^{13}C NMR spectra (101 MHz, CD_3OD) of **2m**

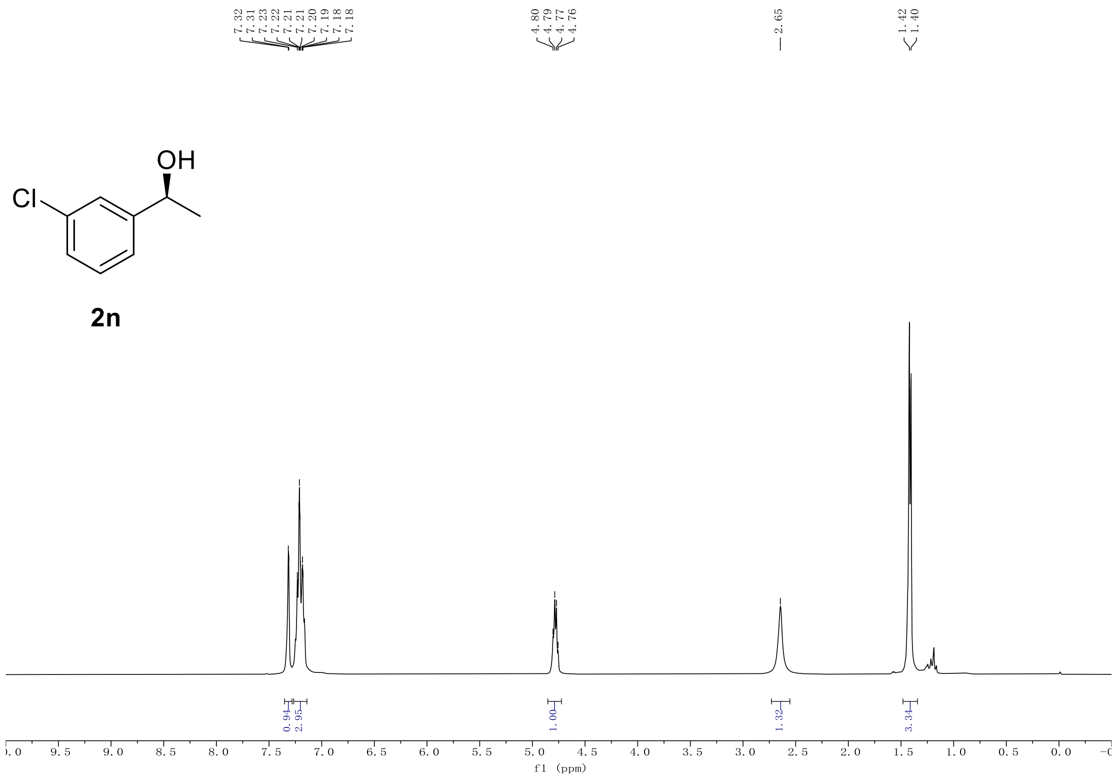

^{1}H NMR spectra (400 MHz, $CDCl_3$) of **2n**

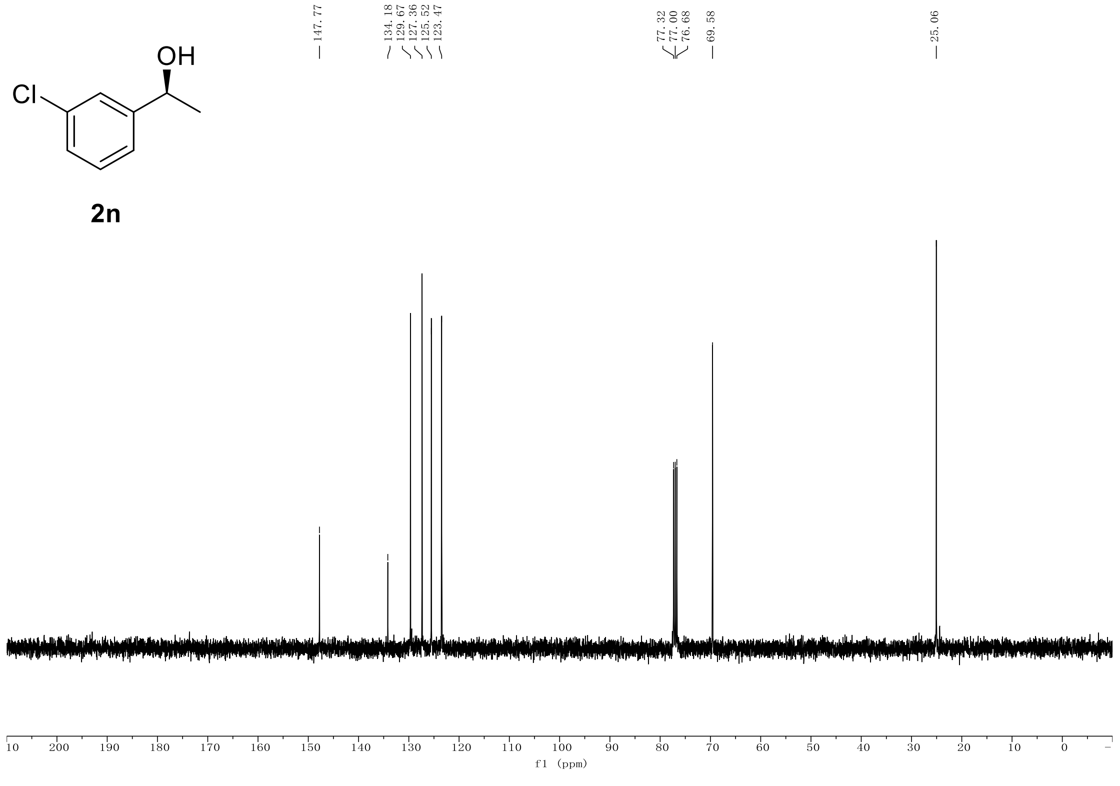

^{13}C NMR spectra (101 MHz, $CDCl_3$) of **2n**

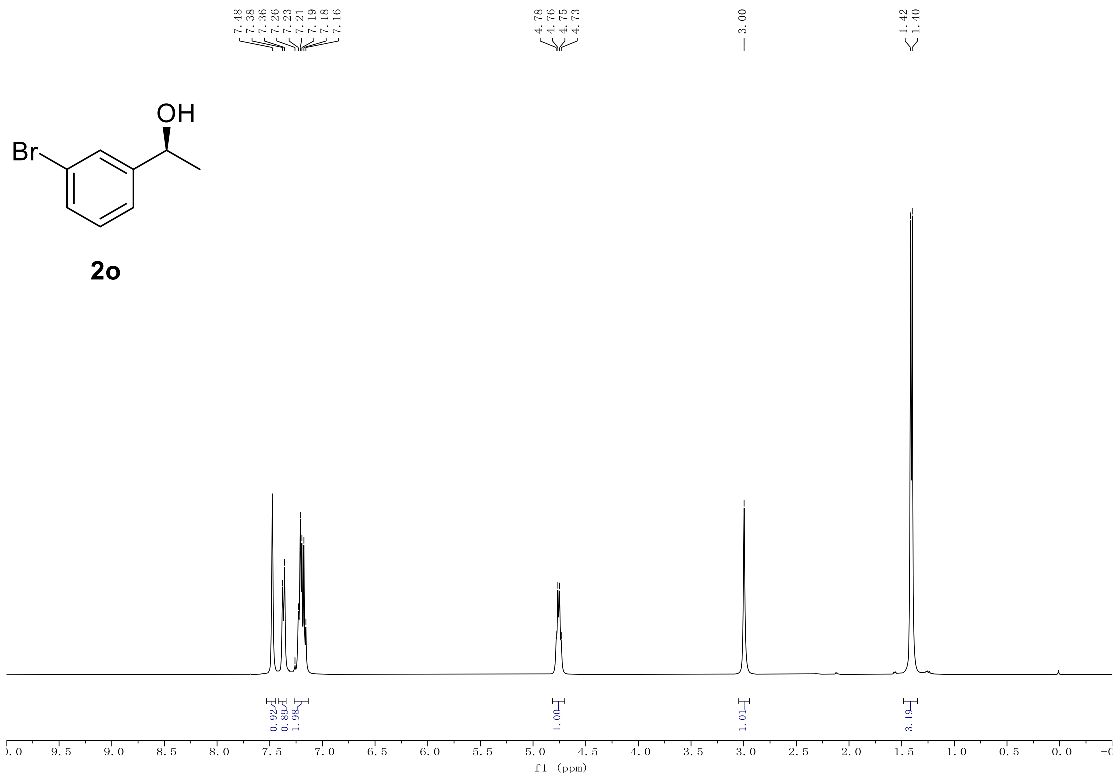

^{1}H NMR spectra (400 MHz, $CDCl_3$) of **2o**

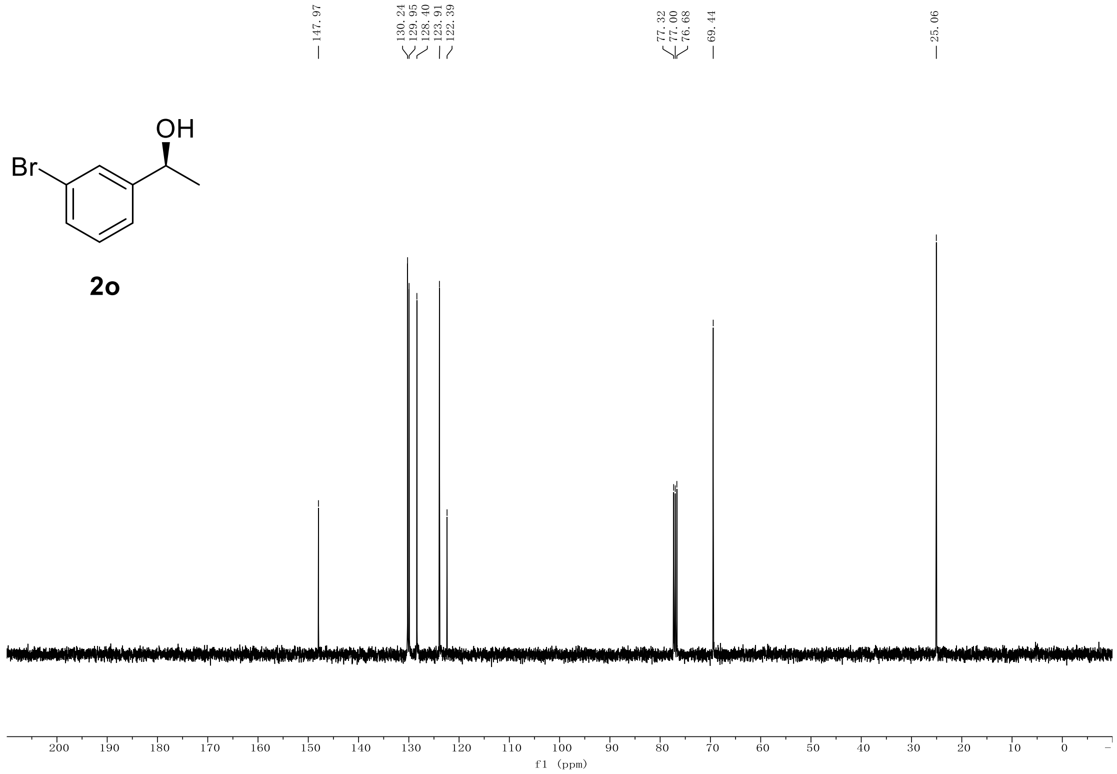

^{13}C NMR spectra (101 MHz, $CDCl_3$) of **2o**

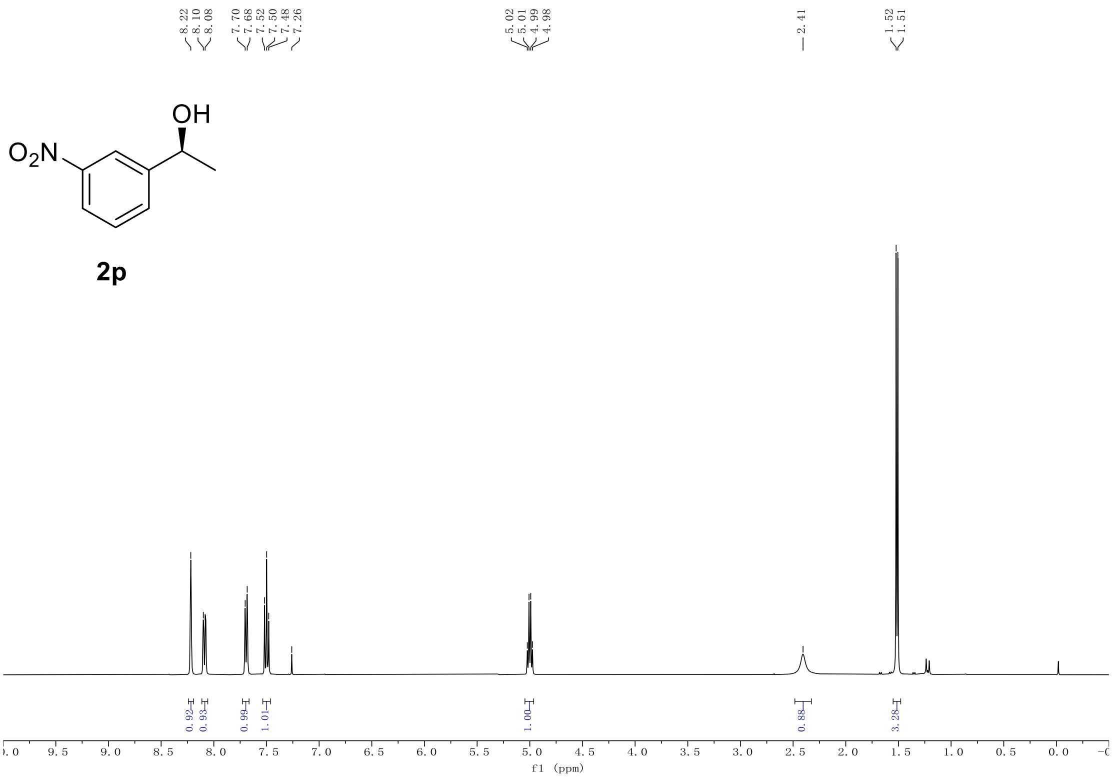

2p

^{1}H NMR spectra (400 MHz, $CDCl_3$) of **2p**

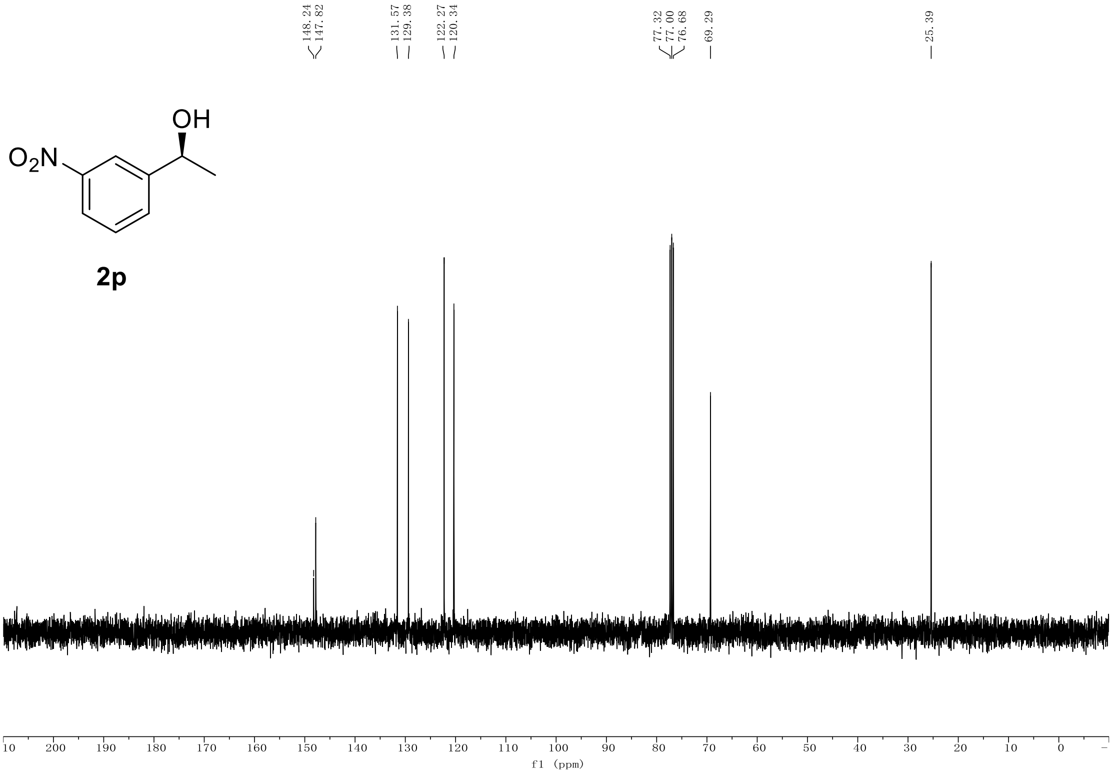

2p

^{13}C NMR spectra (101 MHz, $CDCl_3$) of **2p**

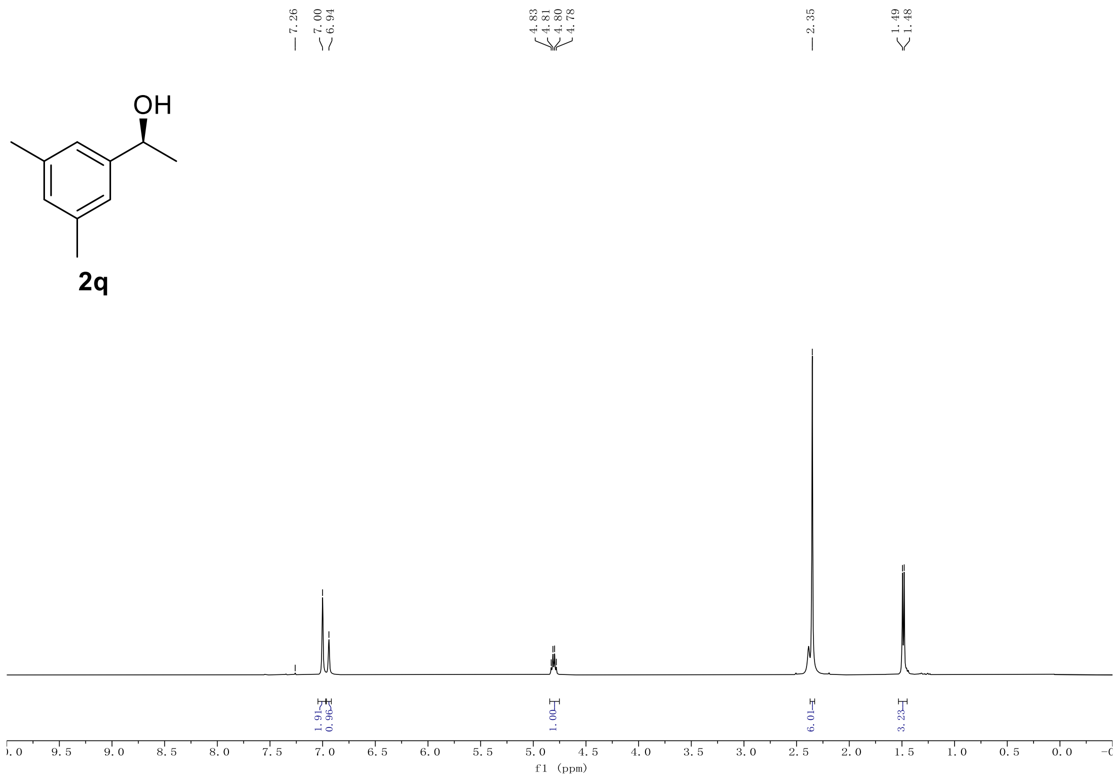

^{1}H NMR spectra (400 MHz, $CDCl_3$) of **2q**

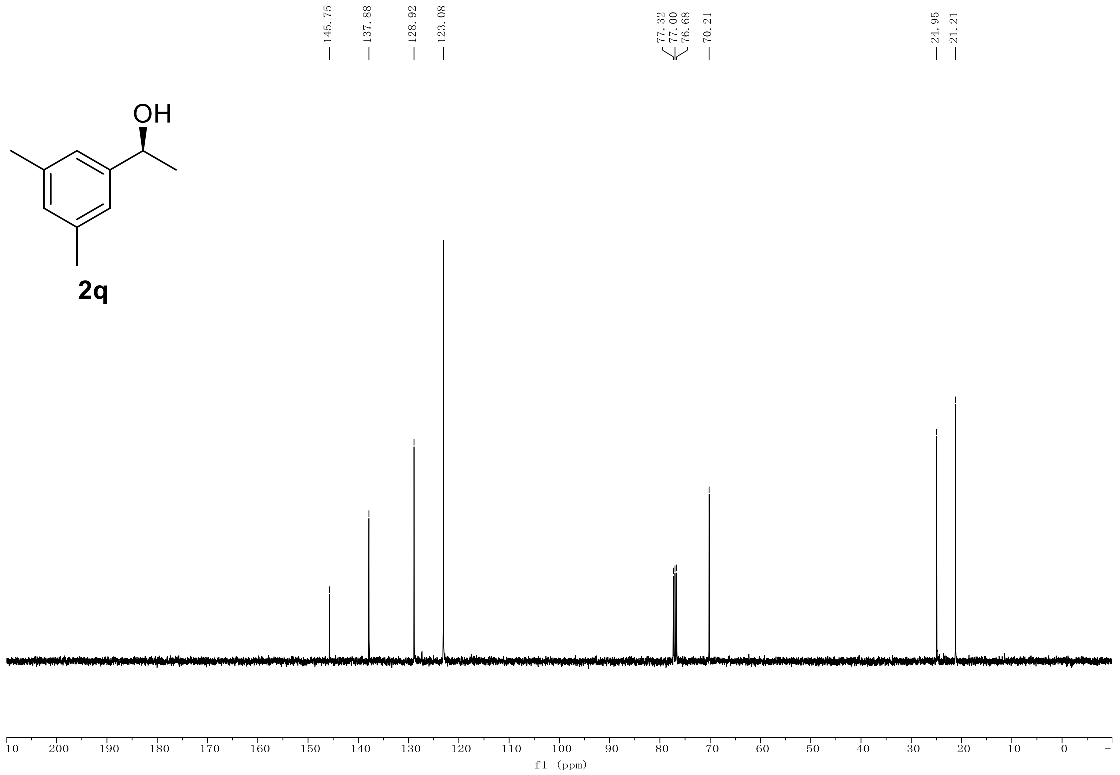

^{13}C NMR spectra (101 MHz, $CDCl_3$) of **2q**

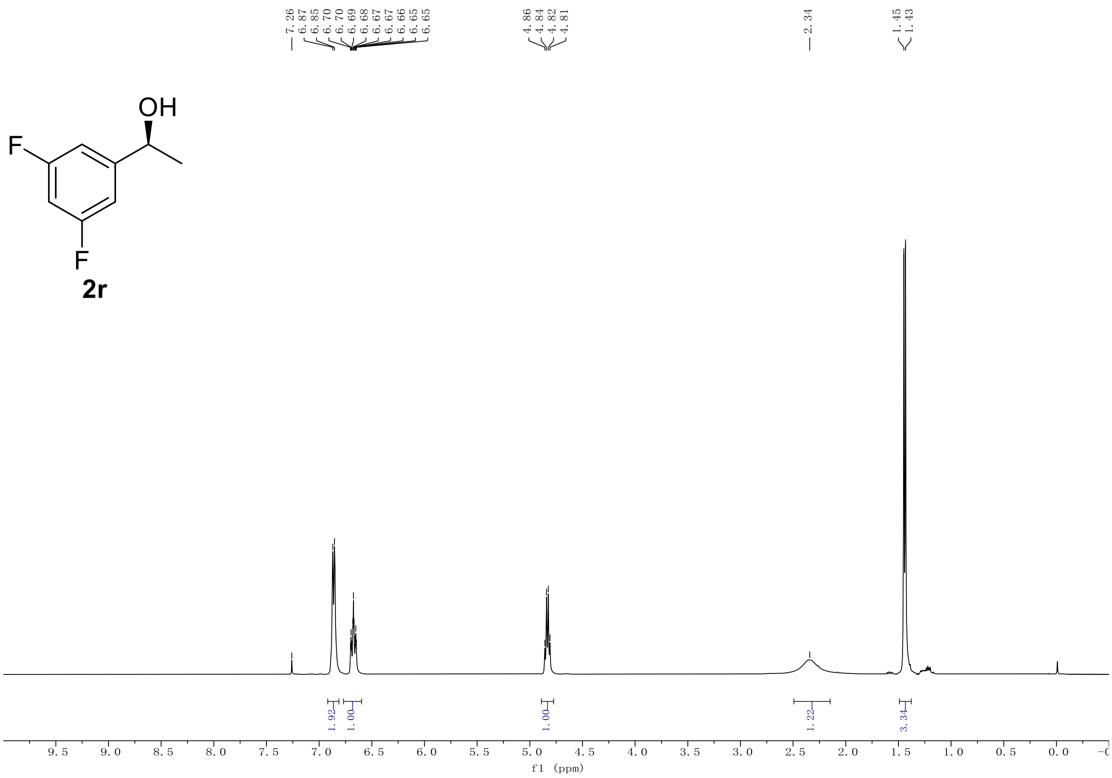

^{1}H NMR spectra (400 MHz, $CDCl_3$) of **2r**

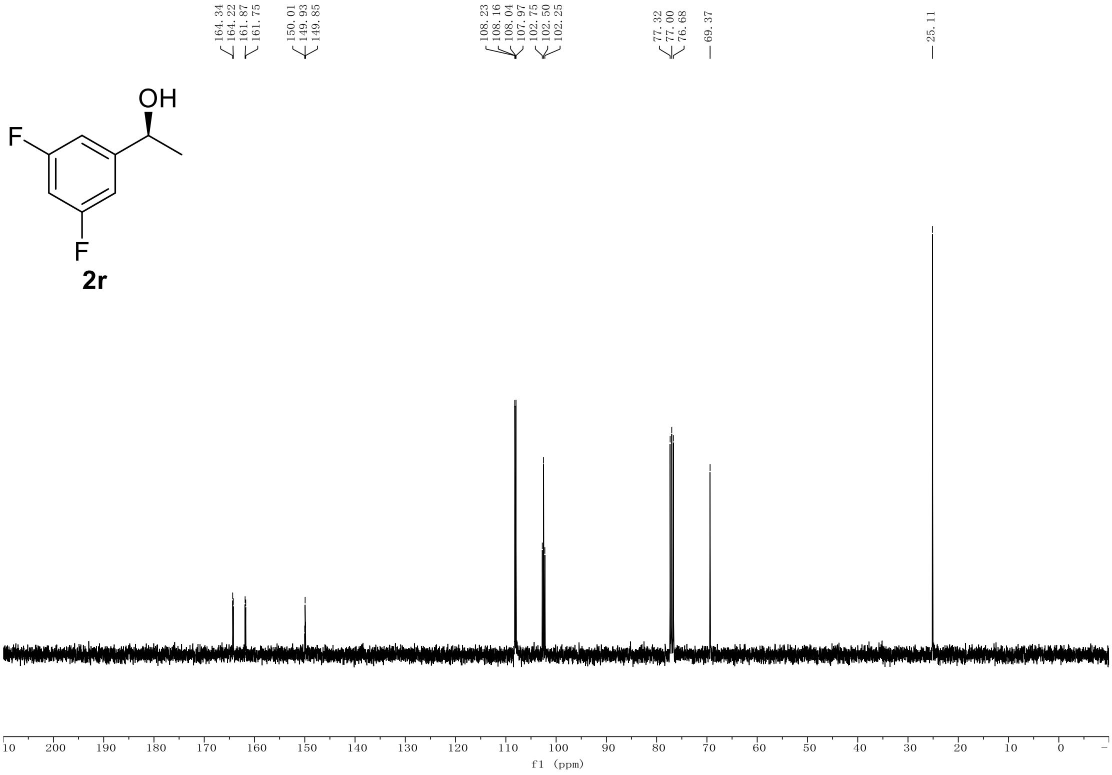

^{13}C NMR spectra (101 MHz, $CDCl_3$) of **2r**

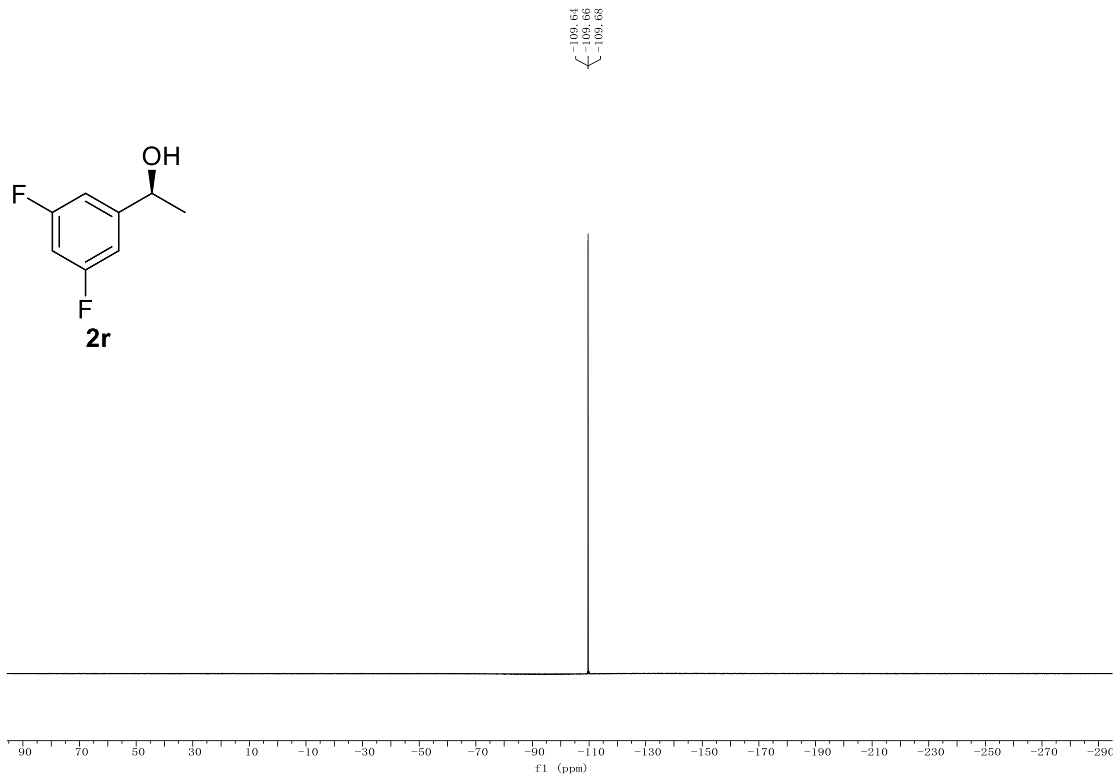

^{19}F NMR spectra (376 MHz, $CDCl_3$) of **2r**

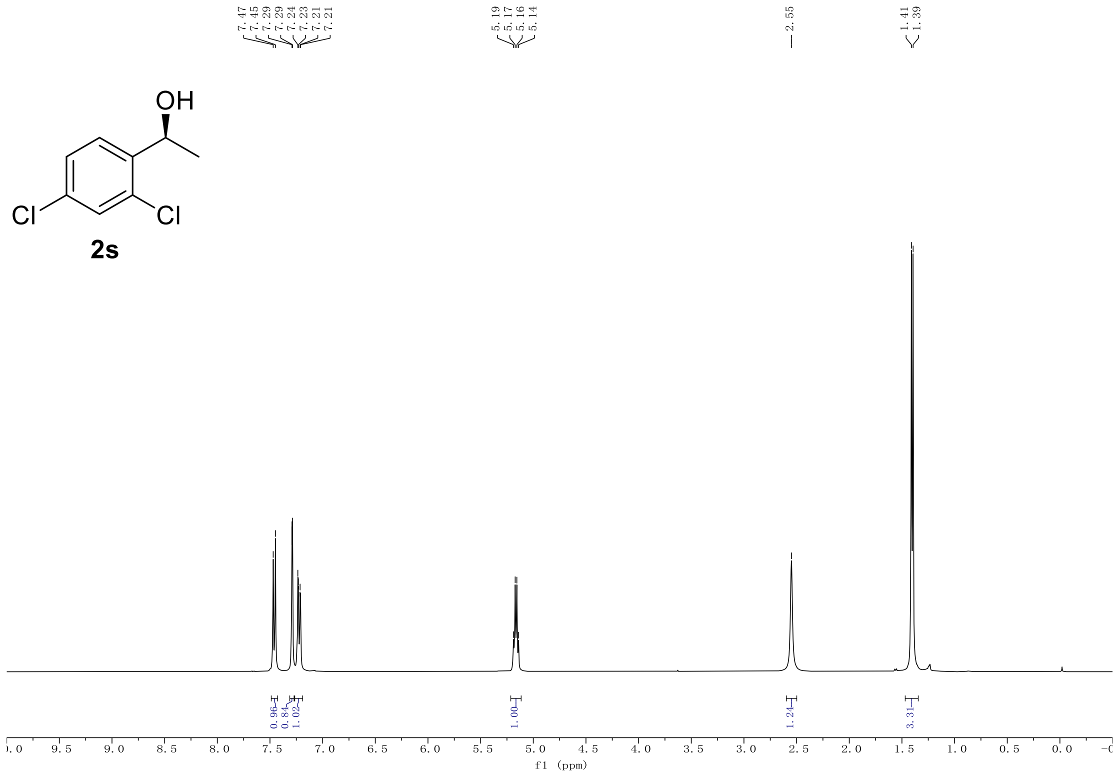

^{1}H NMR spectra (400 MHz, $CDCl_3$) of **2s**

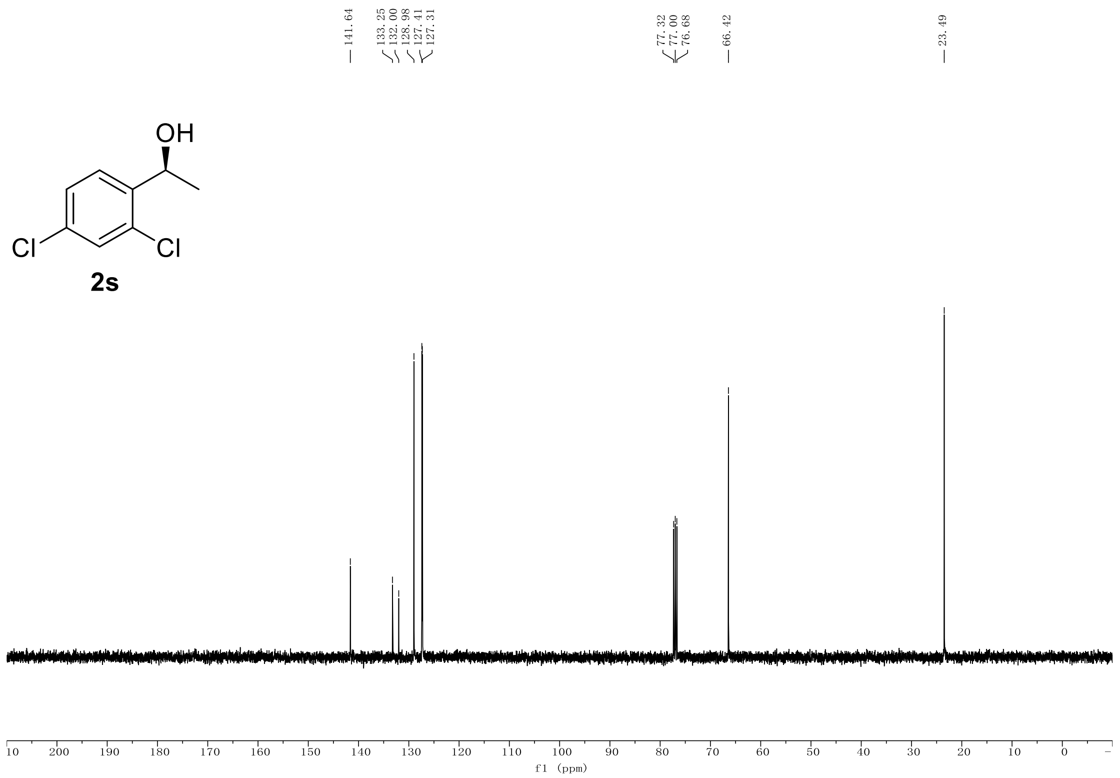

^{13}C NMR spectra (101 MHz, $CDCl_3$) of **2s**

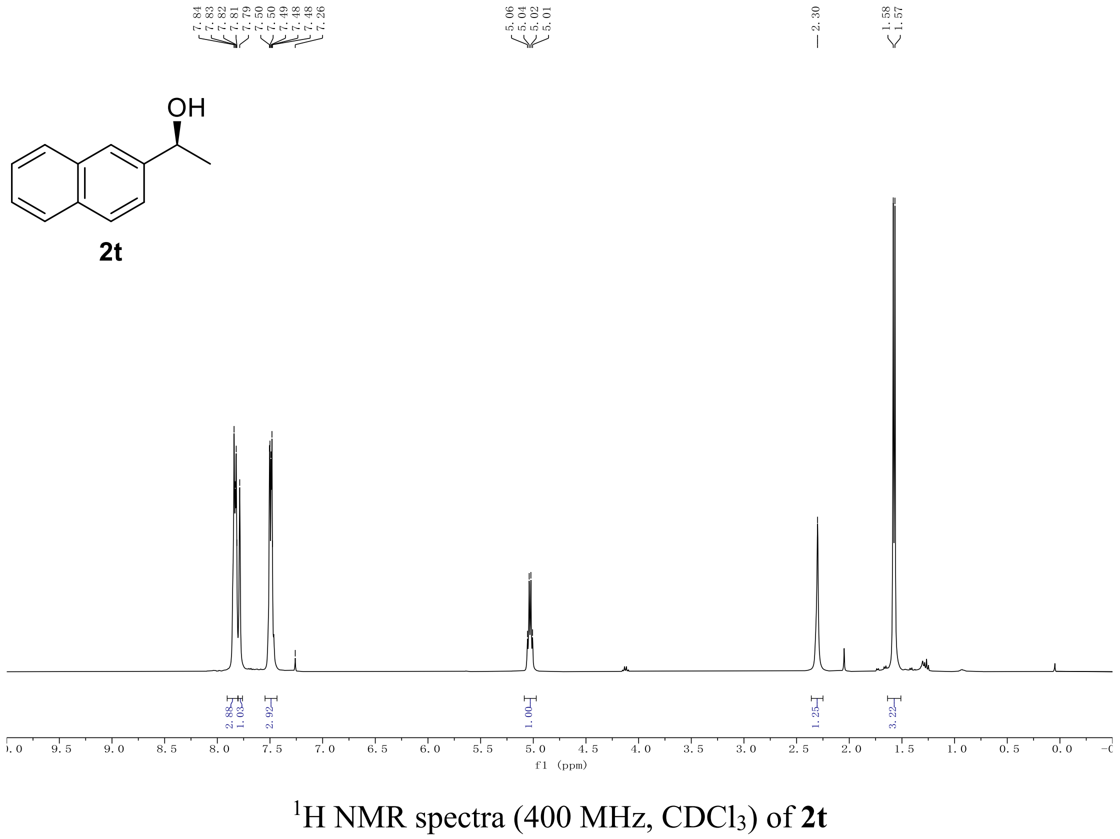

^{1}H NMR spectra (400 MHz, $CDCl_3$) of **2t**

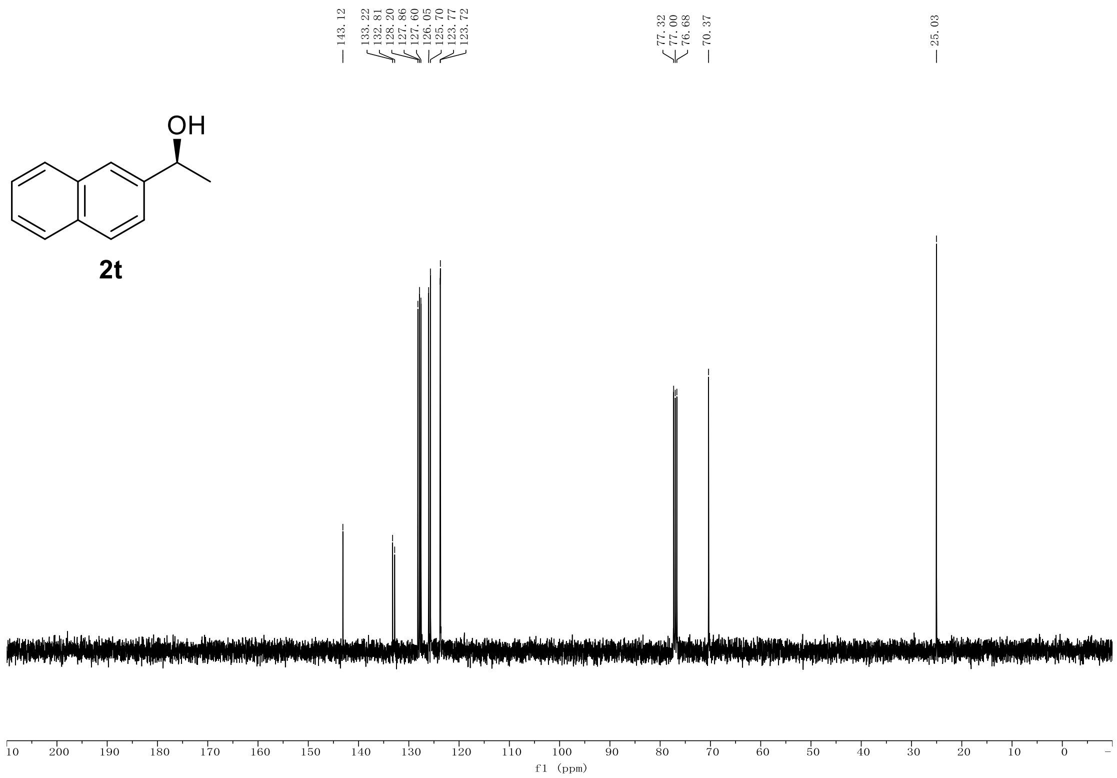

^{13}C NMR spectra (101 MHz, $CDCl_3$) of **2t**

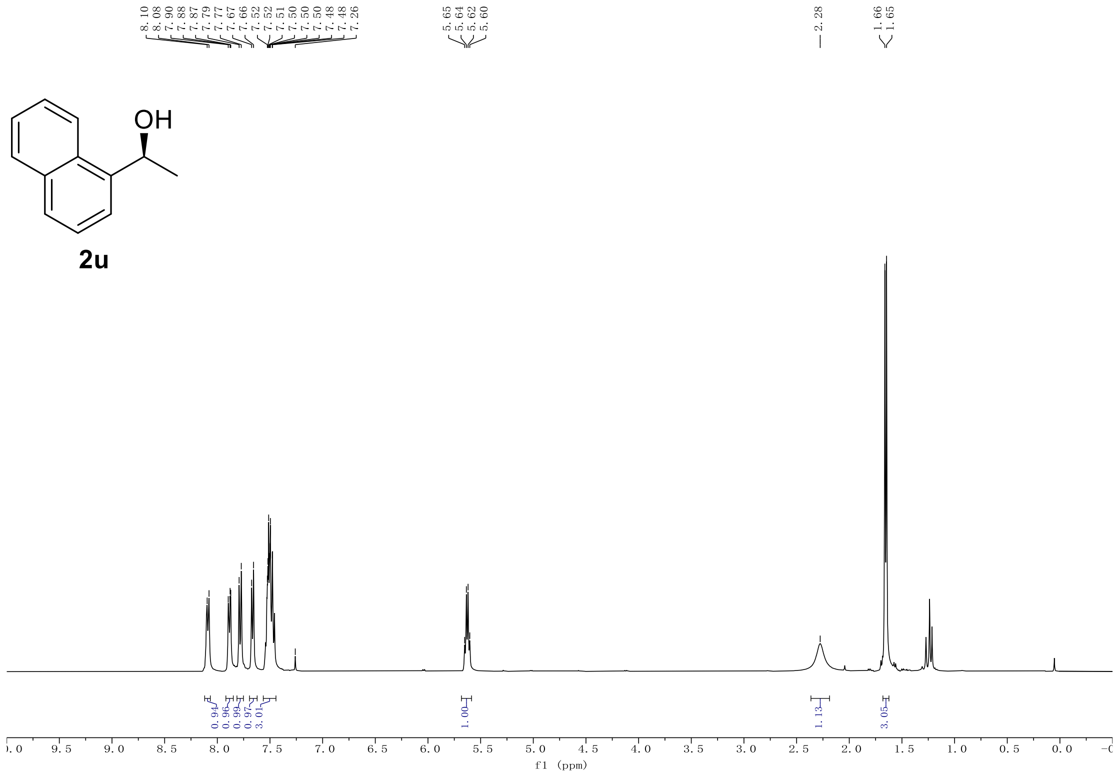

^{1}H NMR spectra (400 MHz, $CDCl_3$) of **2u**

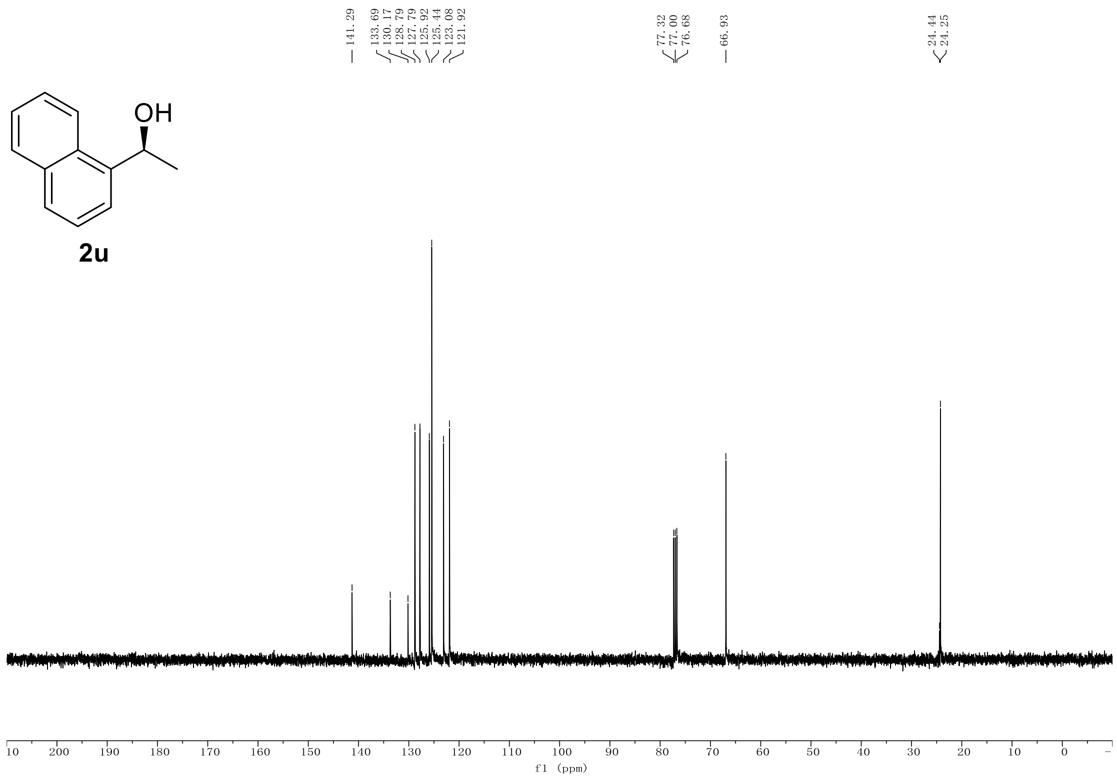

^{13}C NMR spectra (101 MHz, $CDCl_3$) of **2u**

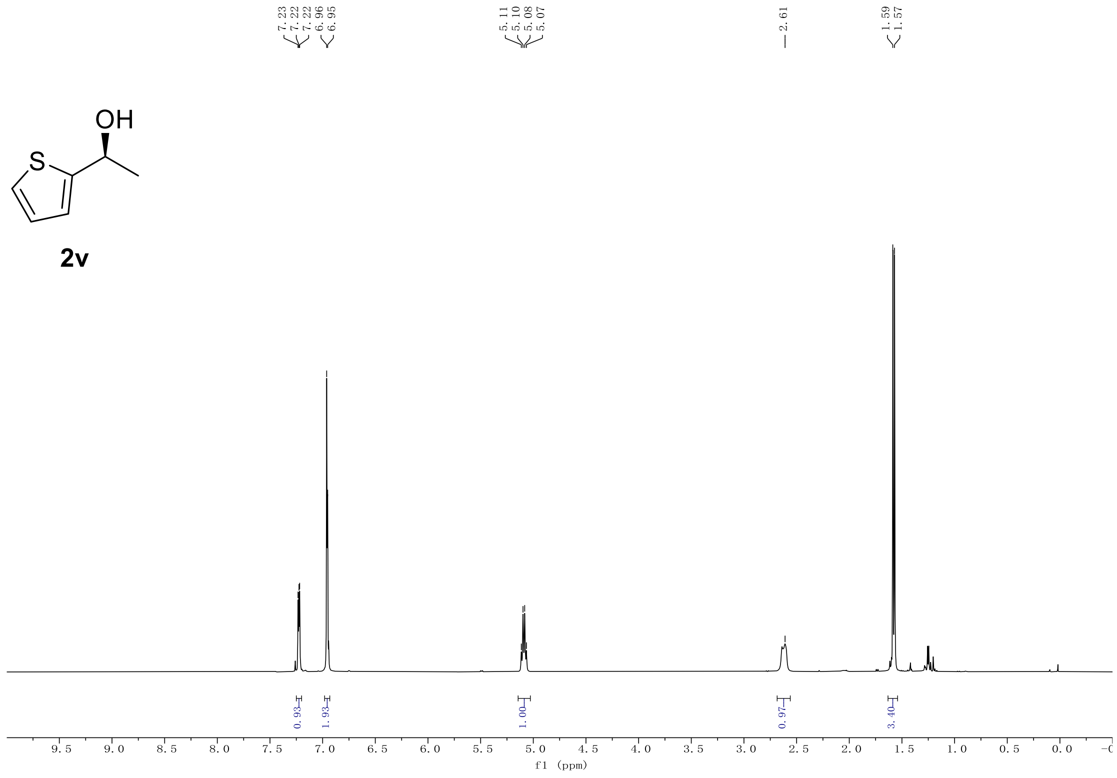

^{1}H NMR spectra (400 MHz, $CDCl_3$) of **2v**

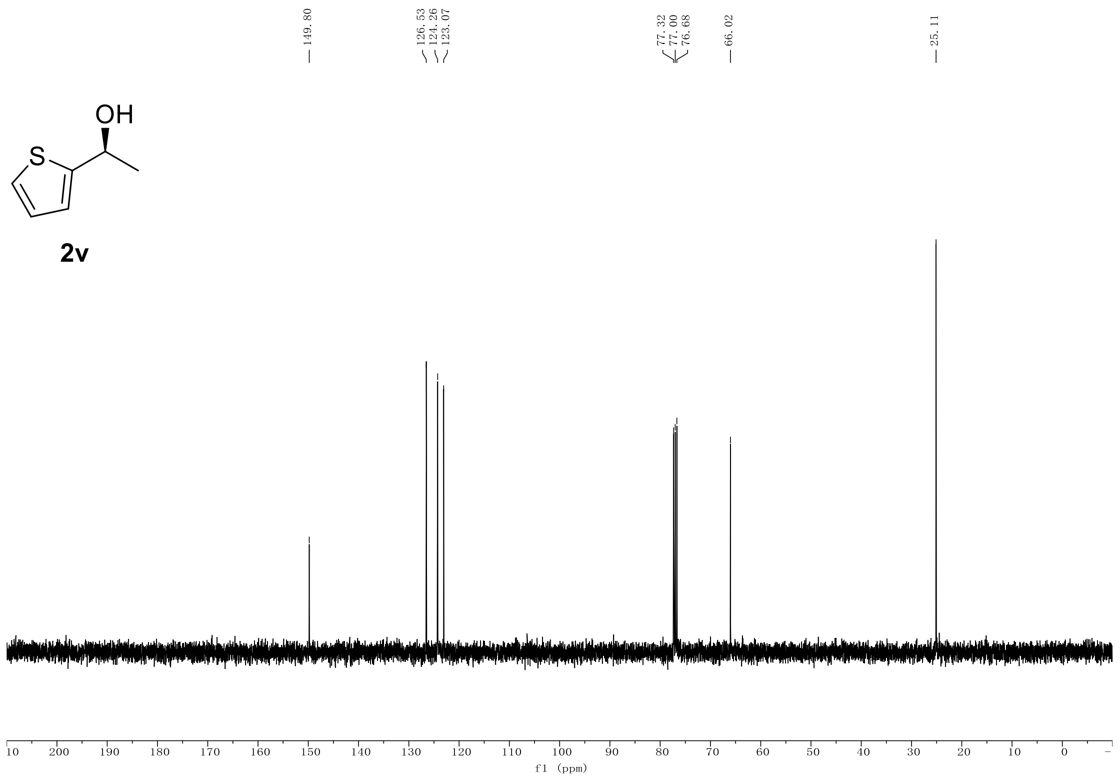

^{13}C NMR spectra (101 MHz, $CDCl_3$) of **2v**

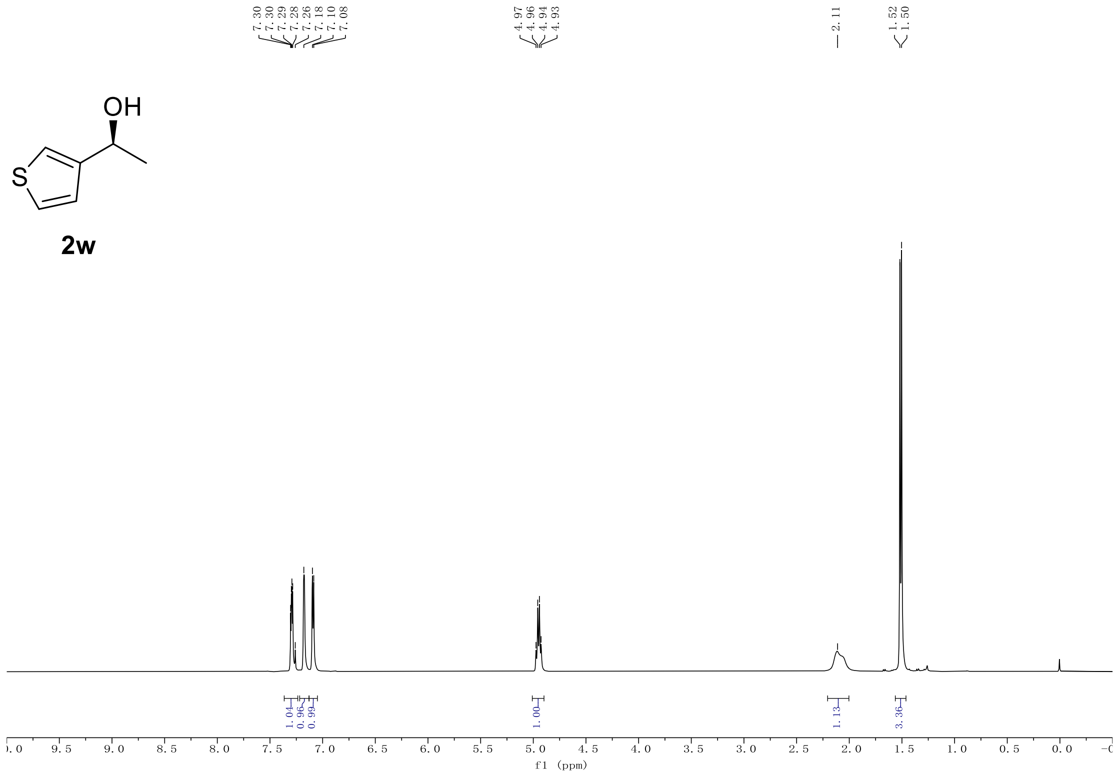

^{1}H NMR spectra (400 MHz, $CDCl_3$) of **2w**

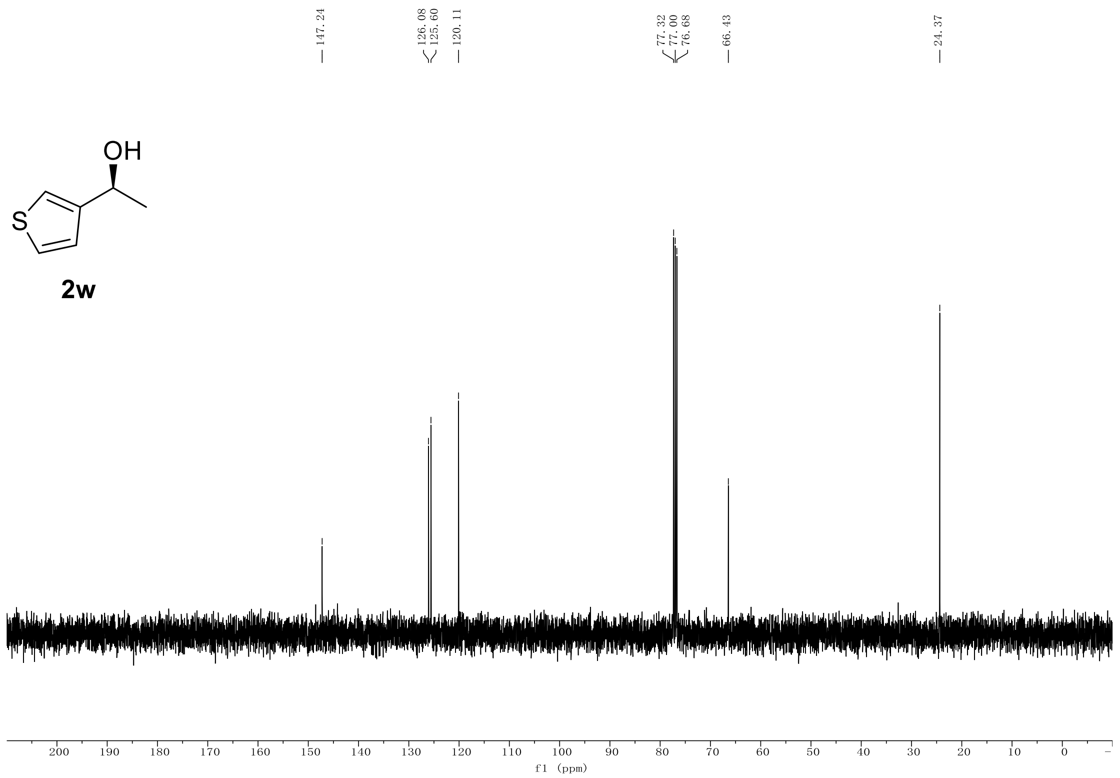

^{13}C NMR spectra (101 MHz, $CDCl_3$) of **2w**

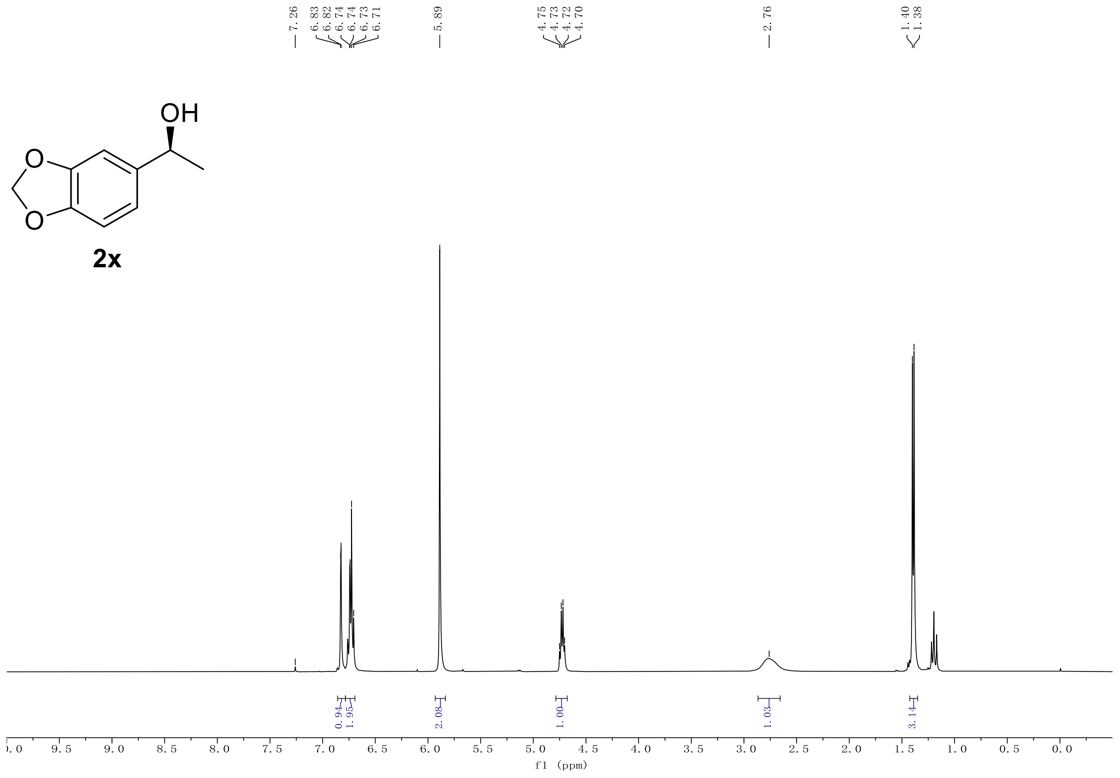

^{1}H NMR spectra (400 MHz, $CDCl_3$) of **2x**

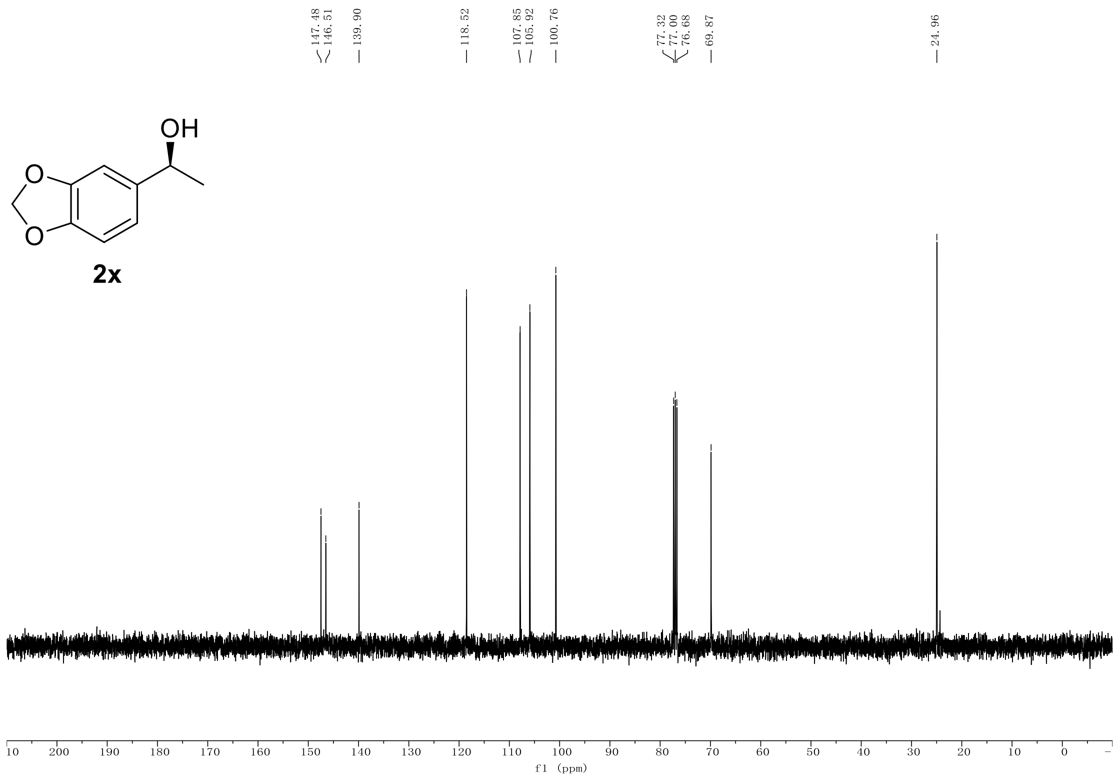

^{13}C NMR spectra (101 MHz, $CDCl_3$) of **2x**

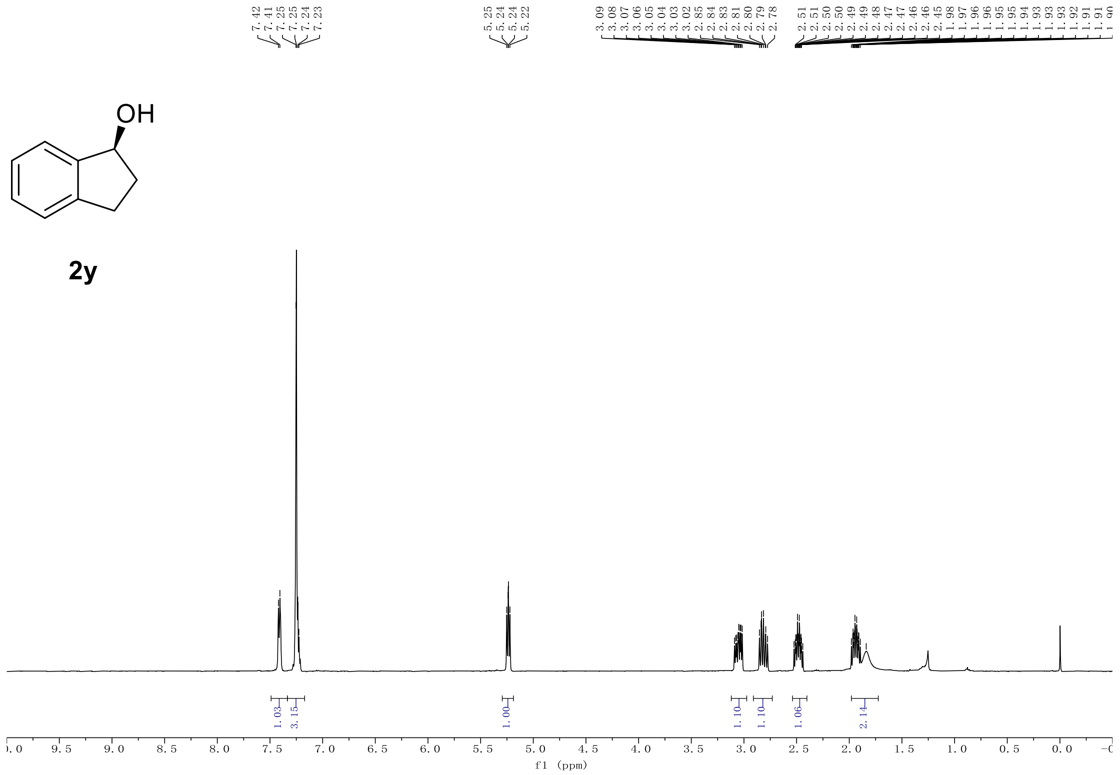

^{1}H NMR spectra (400 MHz, $CDCl_3$) of **2y**

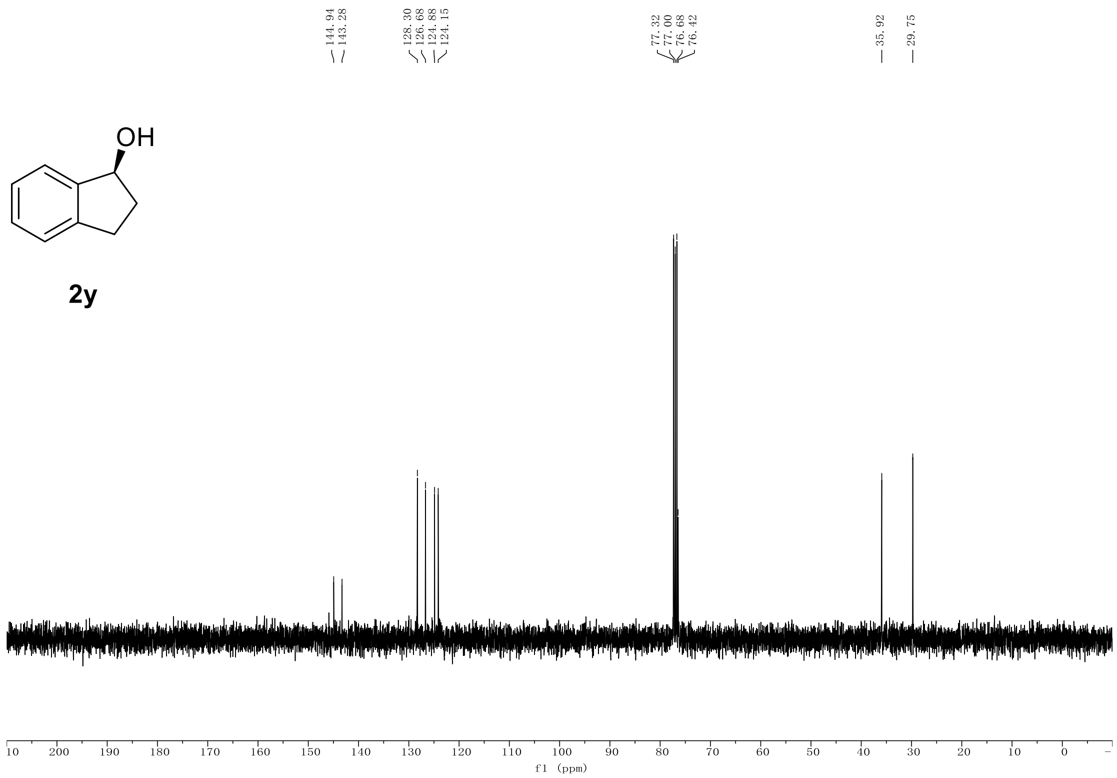

^{13}C NMR spectra (101 MHz, CDCl$_3$) of **2y**

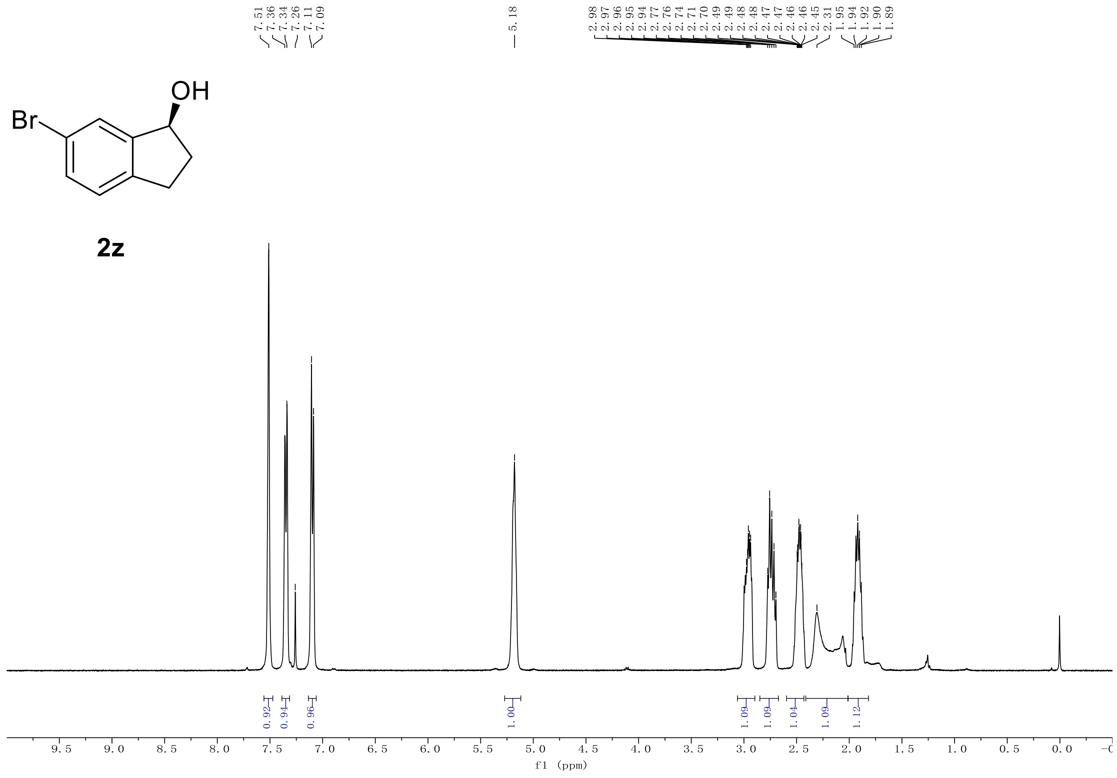

^{1}H NMR spectra (400 MHz, CDCl$_3$) of **2z**

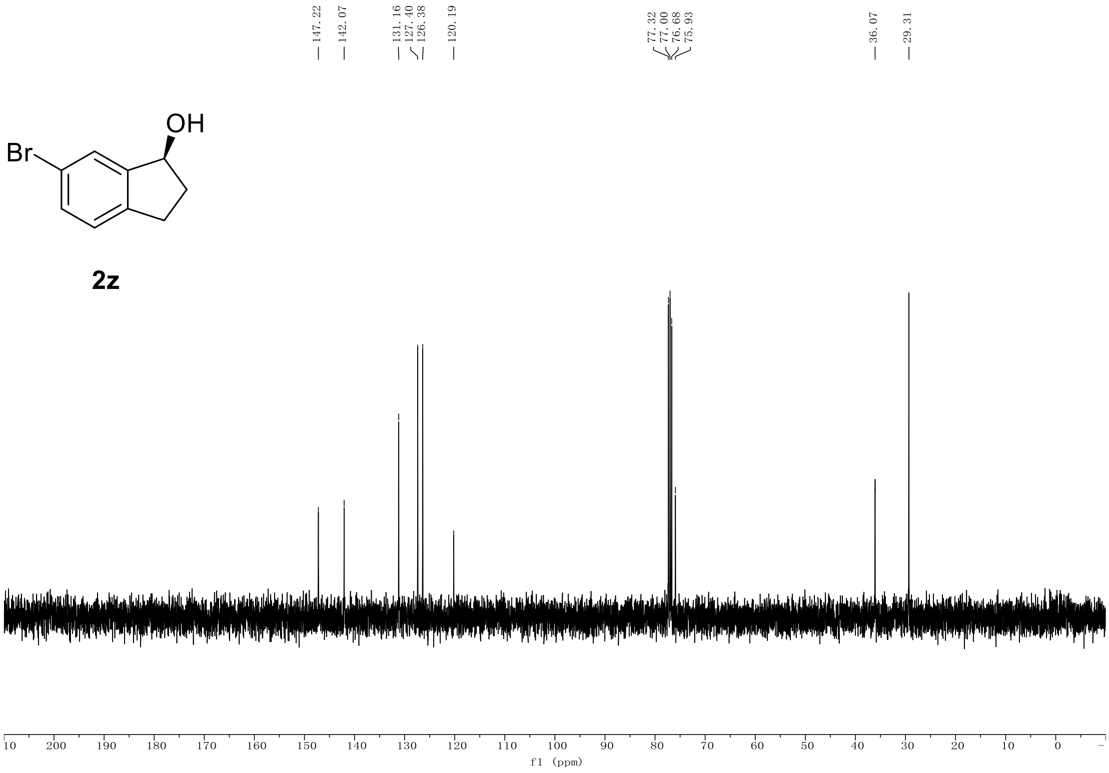

^{13}C NMR spectra (101 MHz, $CDCl_3$) of **2z**

2z

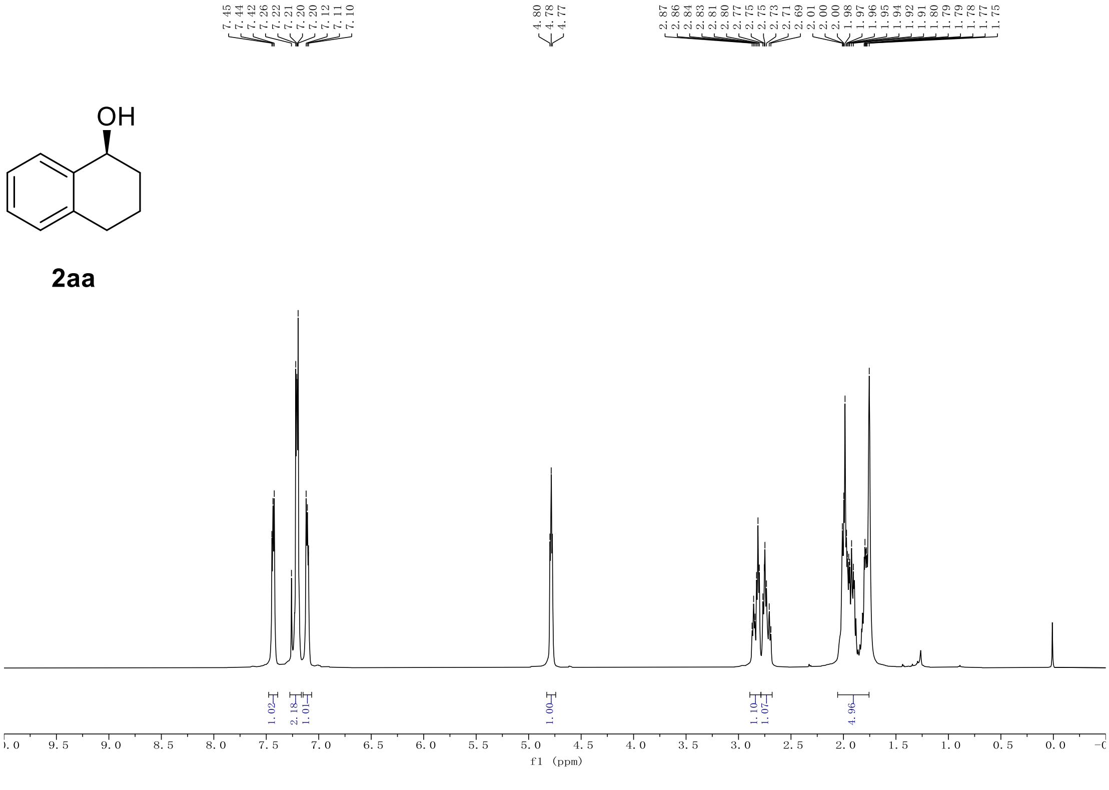

^{1}H NMR spectra (400 MHz, $CDCl_3$) of **2aa**

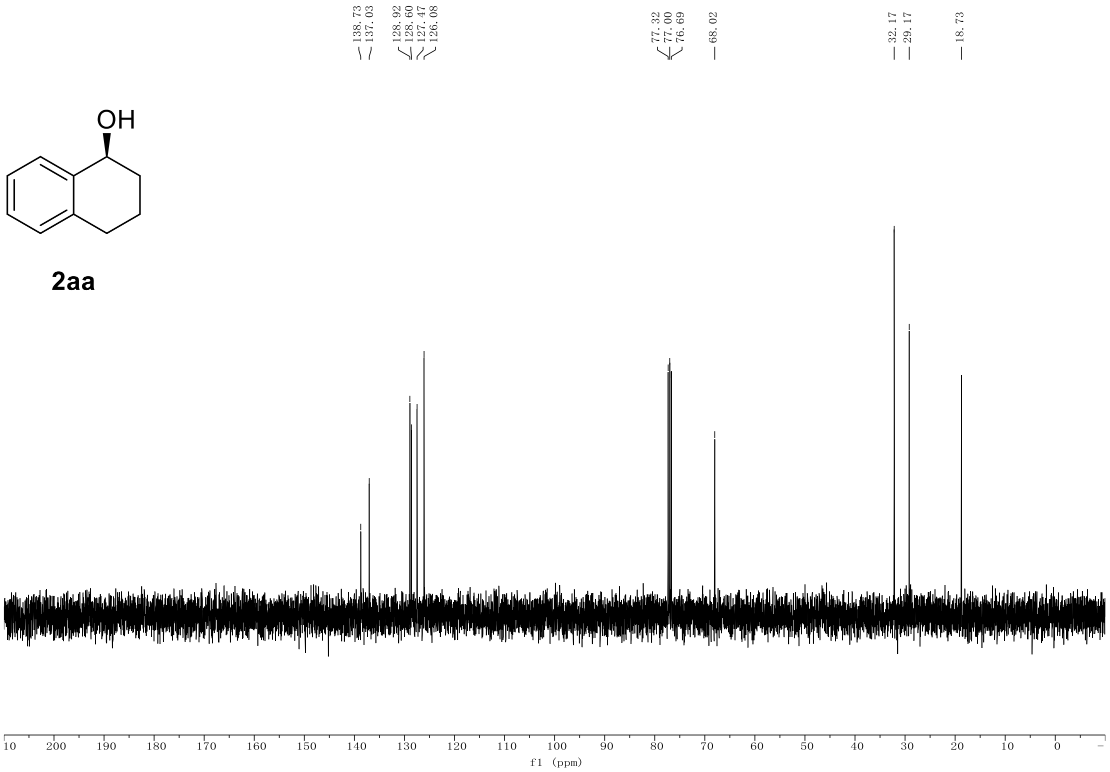

^{13}C NMR spectra (101 MHz, $CDCl_3$) of **2aa**

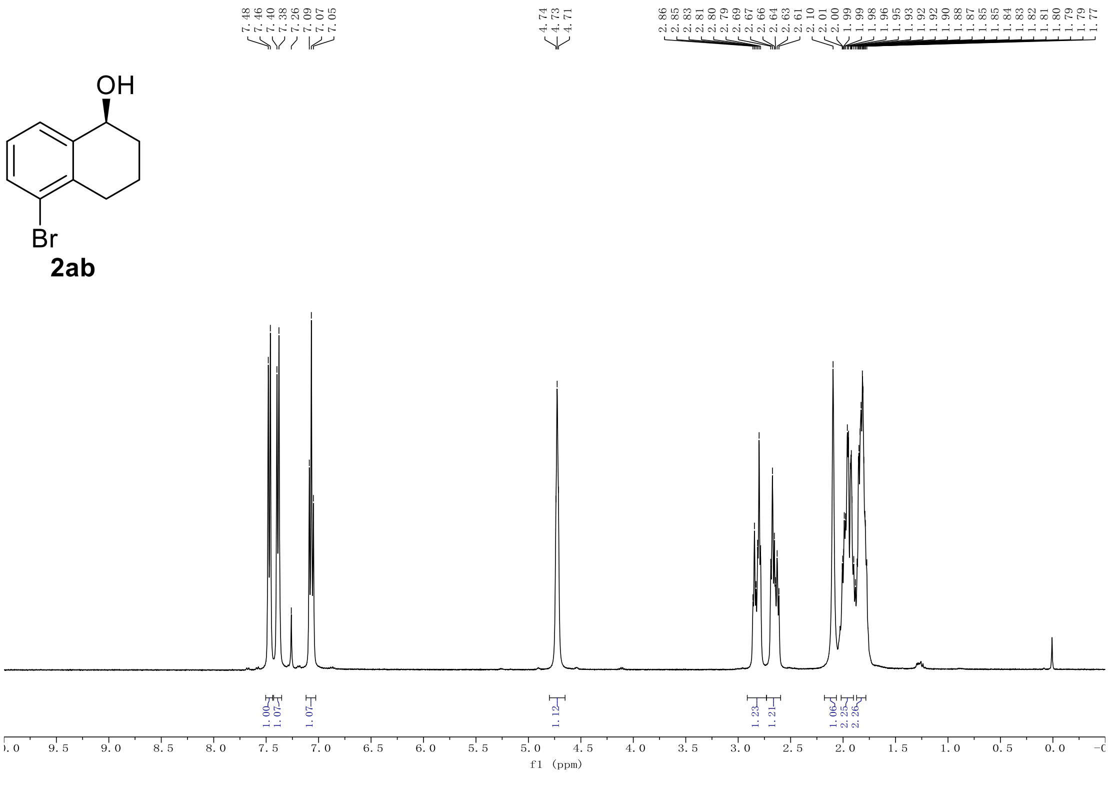

^{1}H NMR spectra (400 MHz, $CDCl_3$) of **2ab**

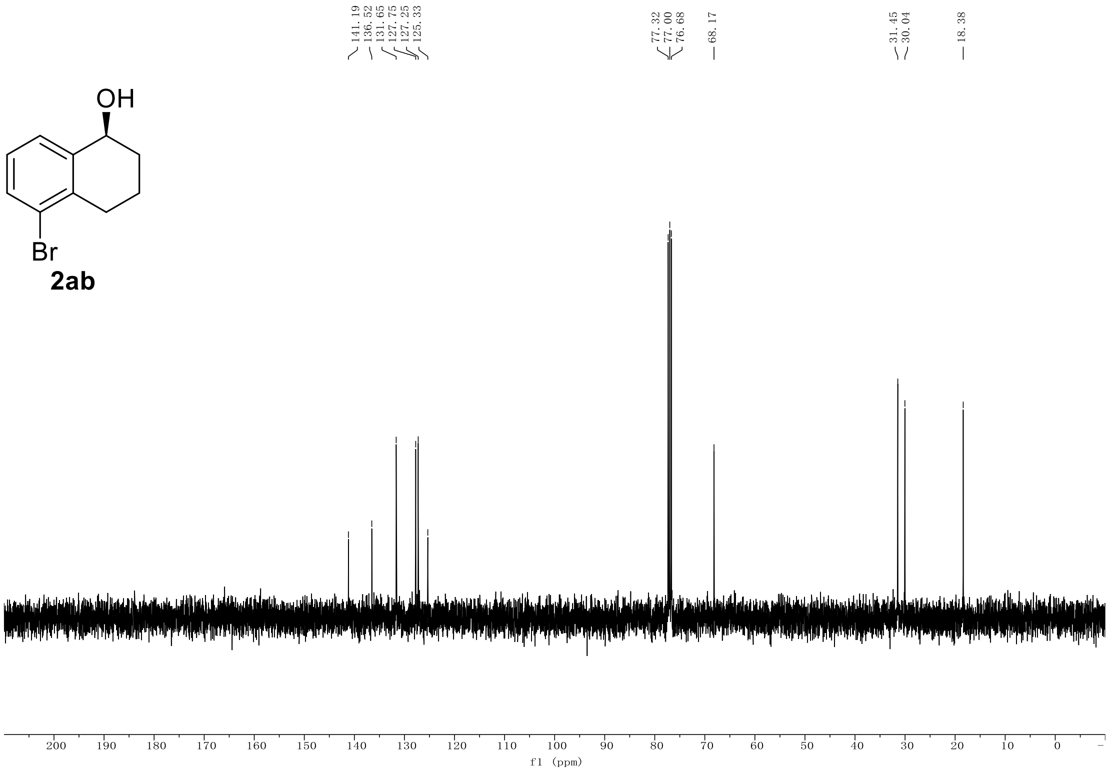

^{13}C NMR spectra (101 MHz, $CDCl_3$) of **2ab**

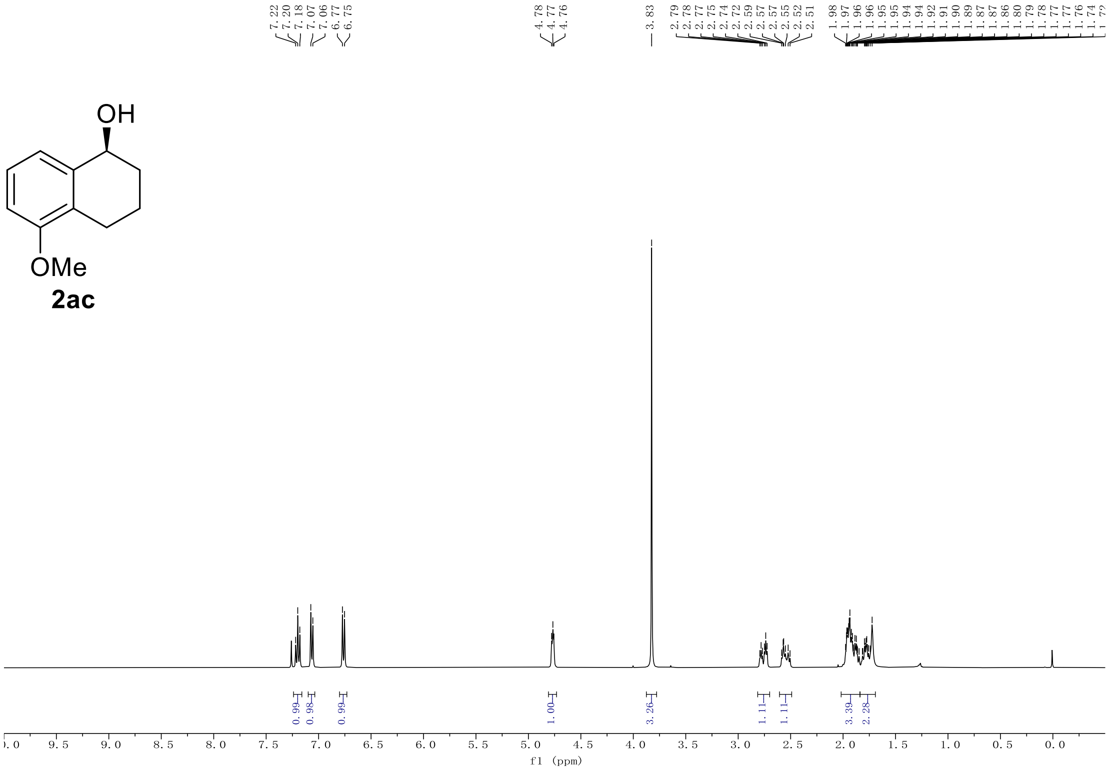

^{1}H NMR spectra (400 MHz, $CDCl_3$) of **2ac**

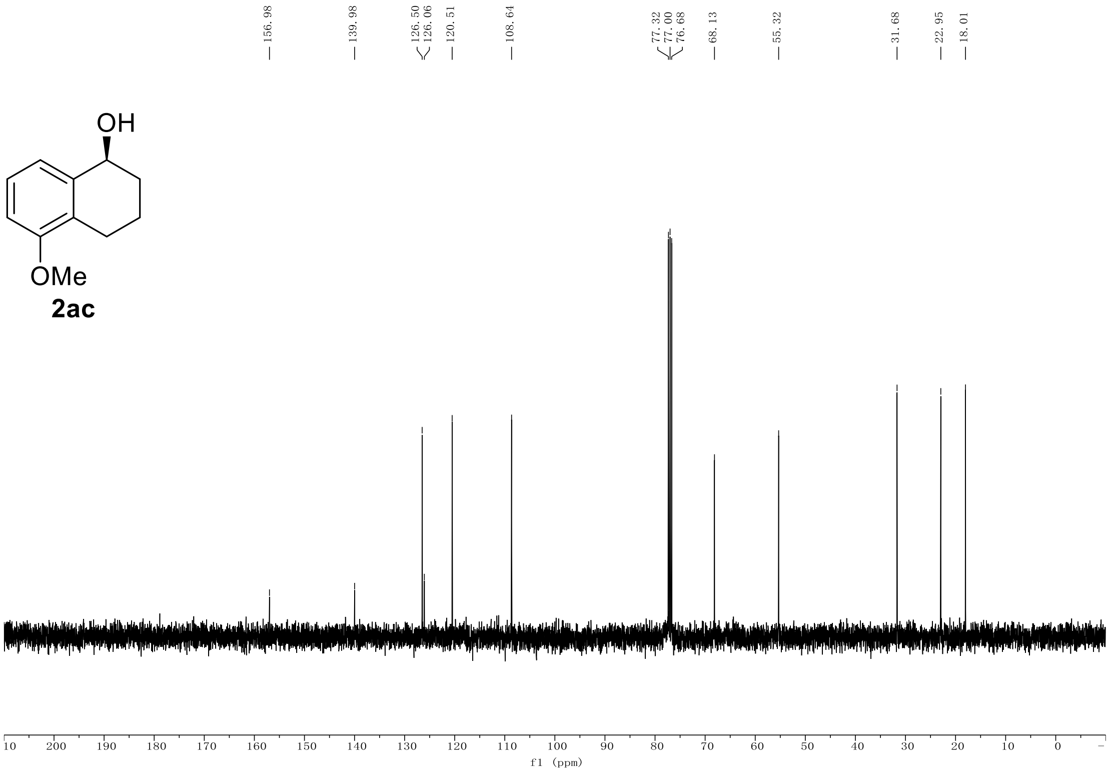

^{13}C NMR spectra (101 MHz, $CDCl_3$) of **2ac**

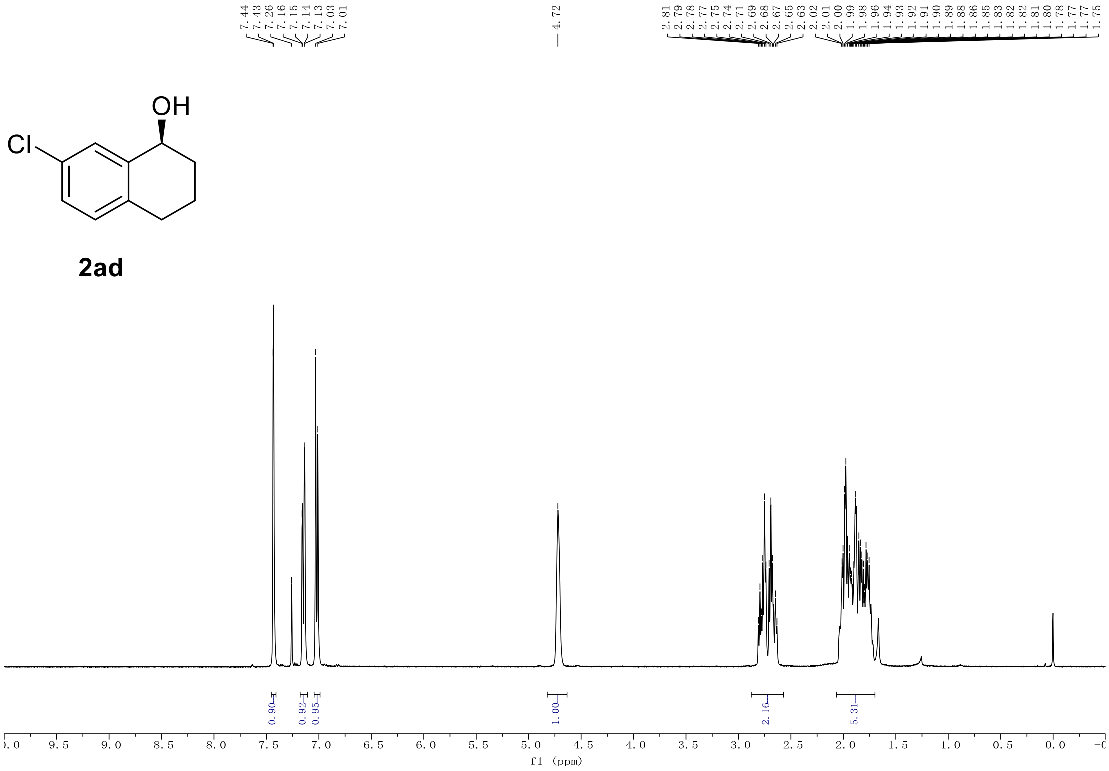

^{1}H NMR spectra (400 MHz, $CDCl_3$) of **2ad**

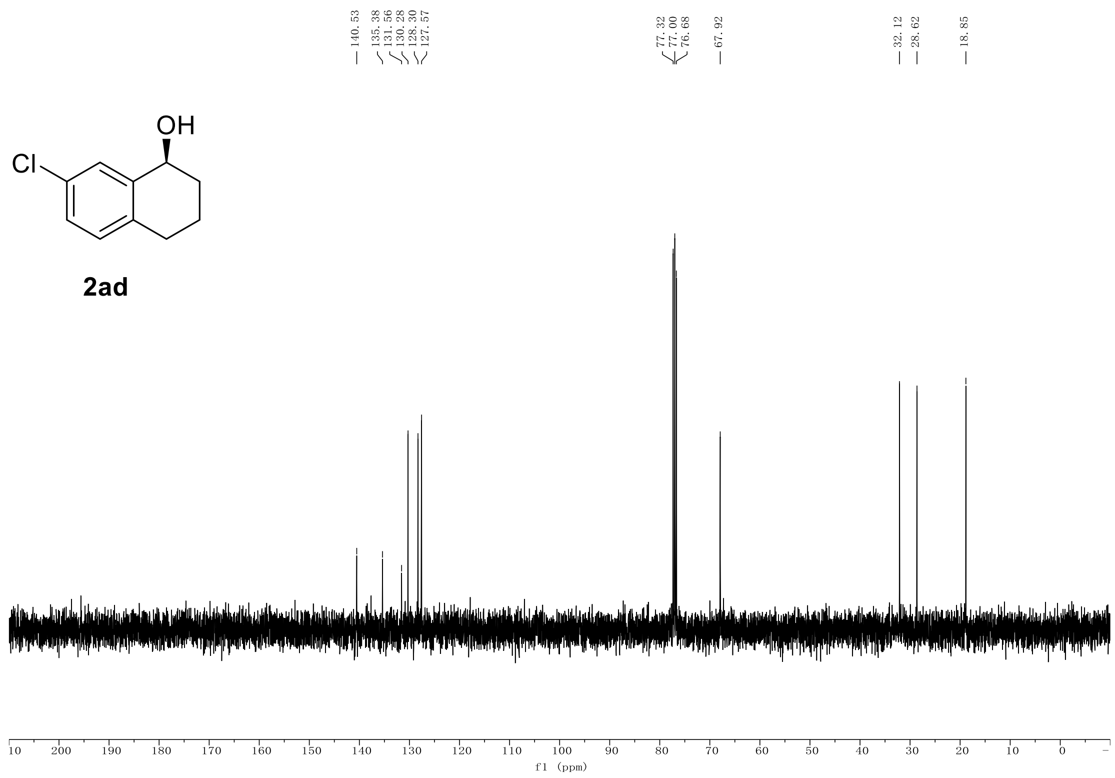

^{13}C NMR spectra (101 MHz, $CDCl_3$) of **2ad**

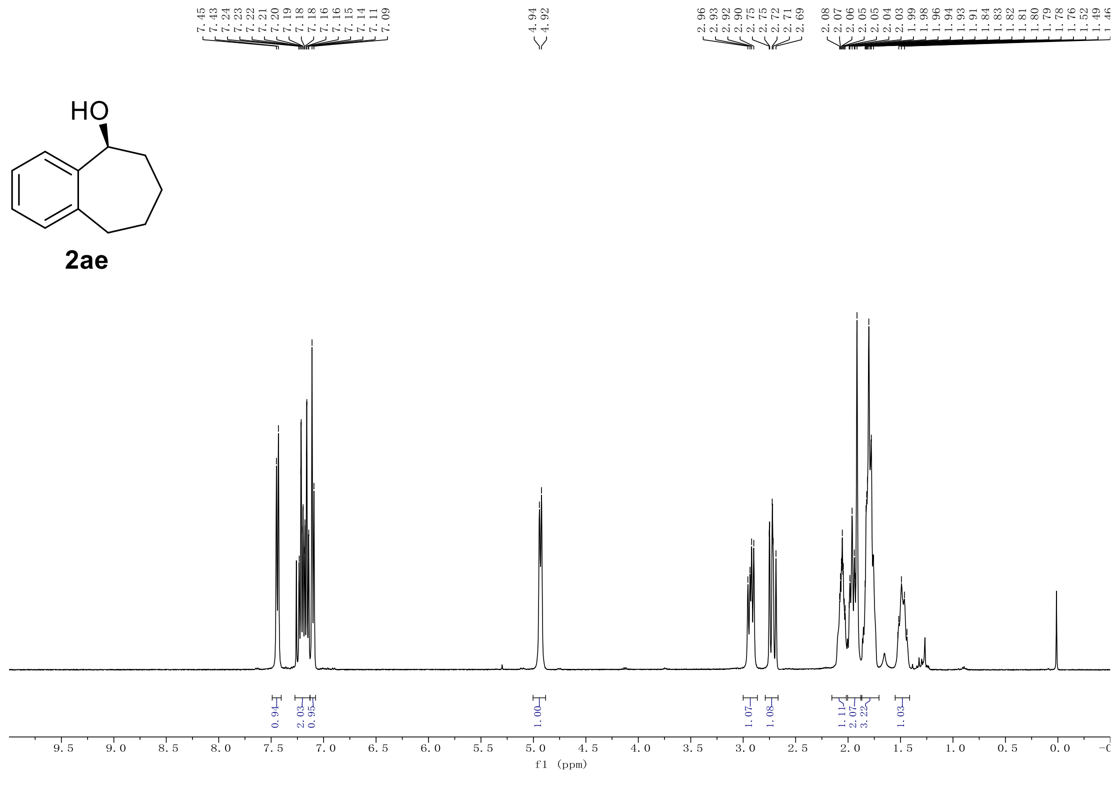

^{1}H NMR spectra (400 MHz, $CDCl_3$) of **2ae**

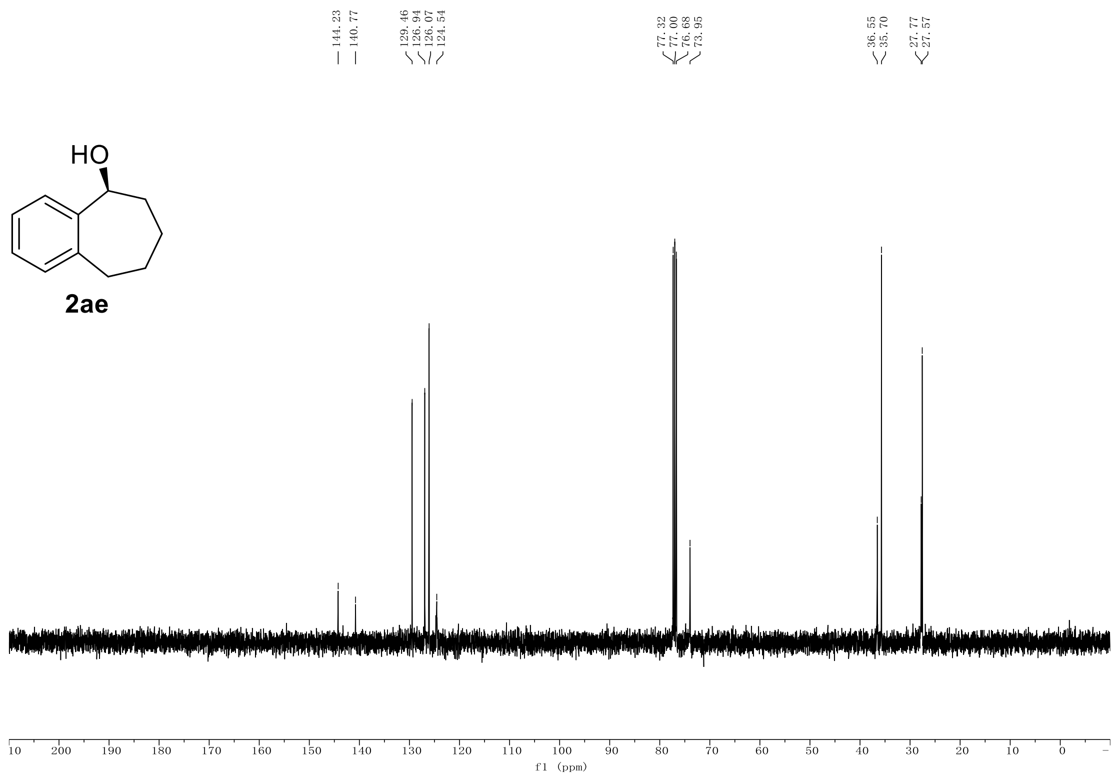

^{13}C NMR spectra (101 MHz, $CDCl_3$) of **2ae**

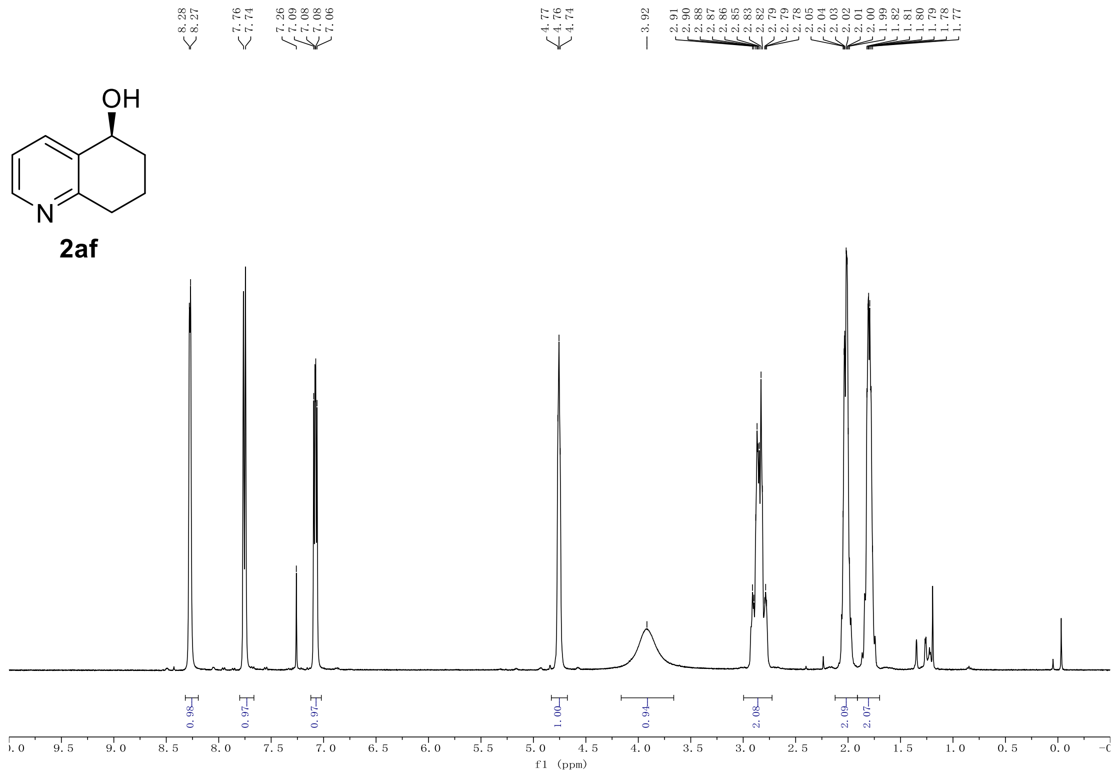

^{1}H NMR spectra (400 MHz, $CDCl_3$) of **2af**

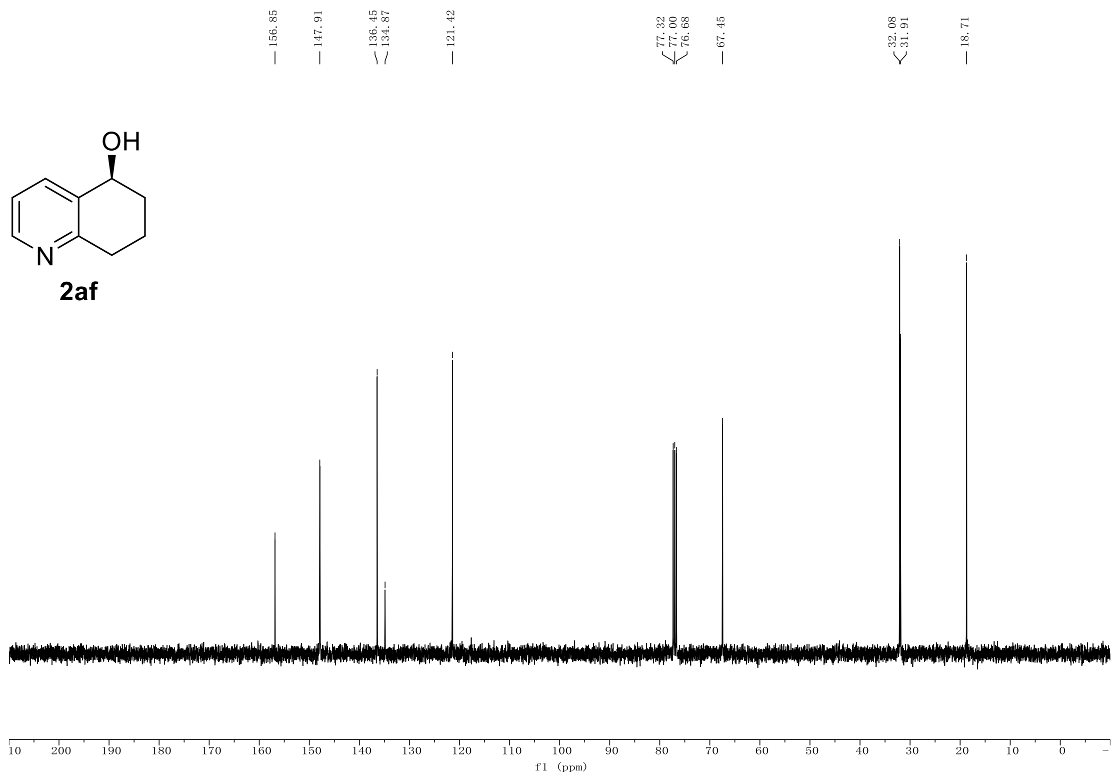

^{13}C NMR spectra (101 MHz, $CDCl_3$) of **2af**

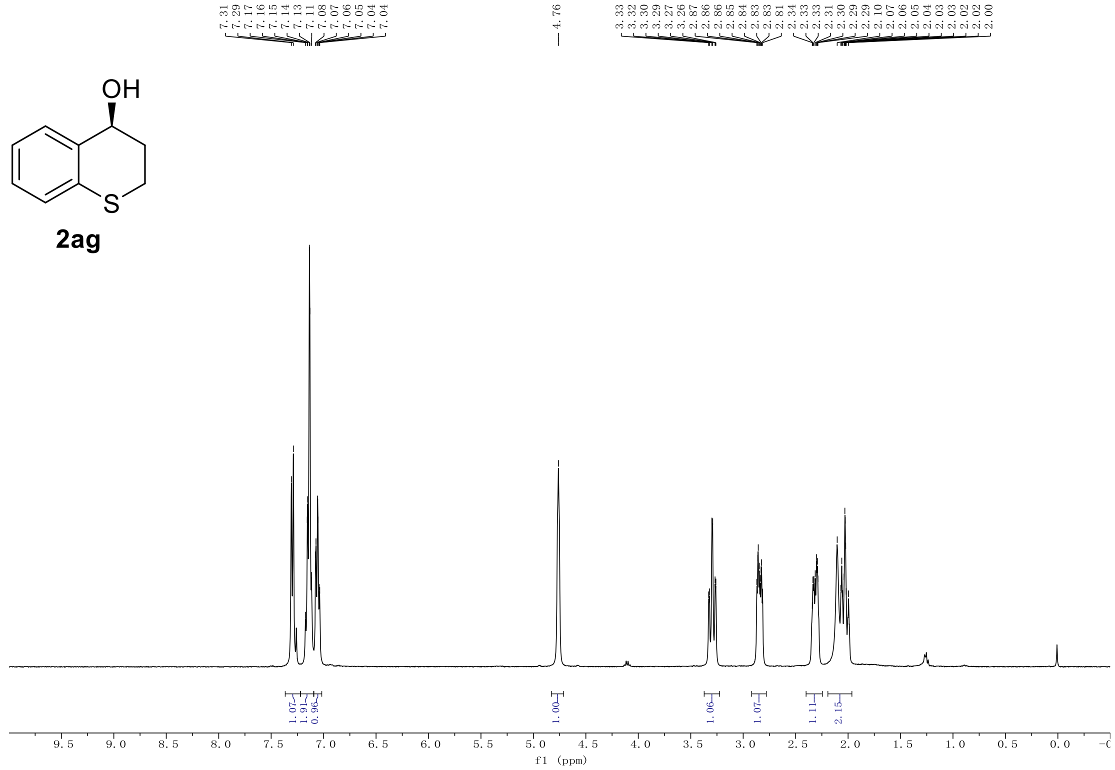

^{1}H NMR spectra (400 MHz, $CDCl_3$) of **2ag**

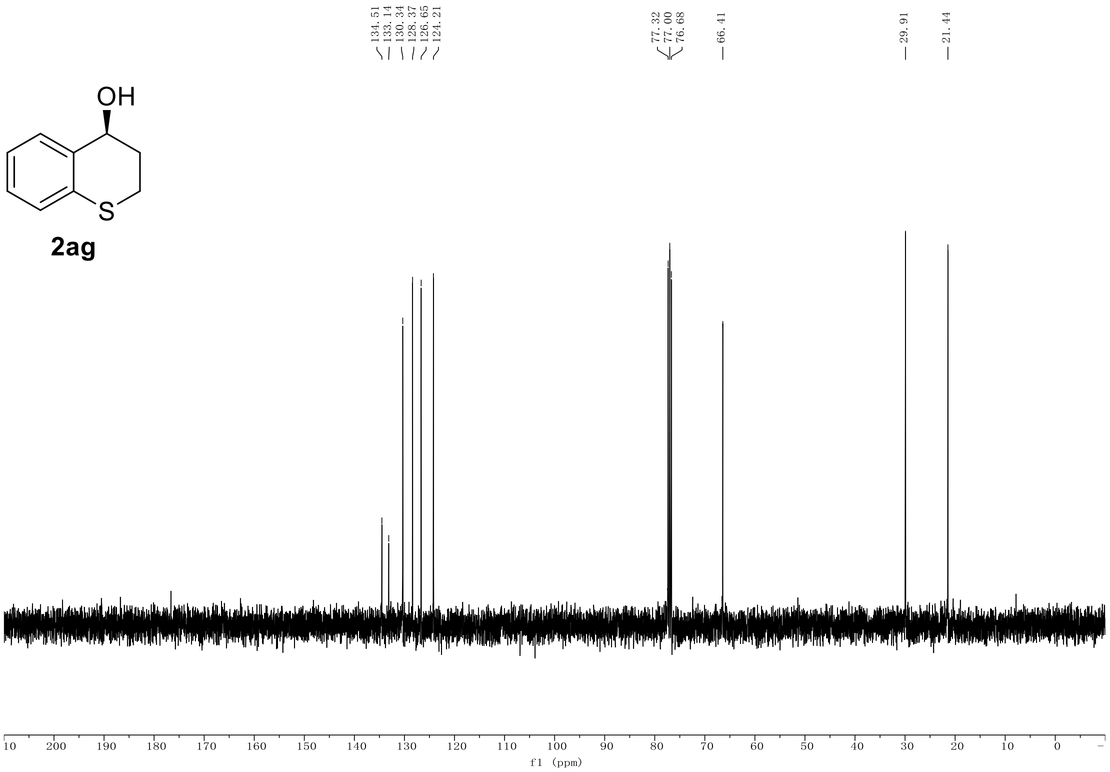

^{13}C NMR spectra (101 MHz, $CDCl_3$) of **2ag**

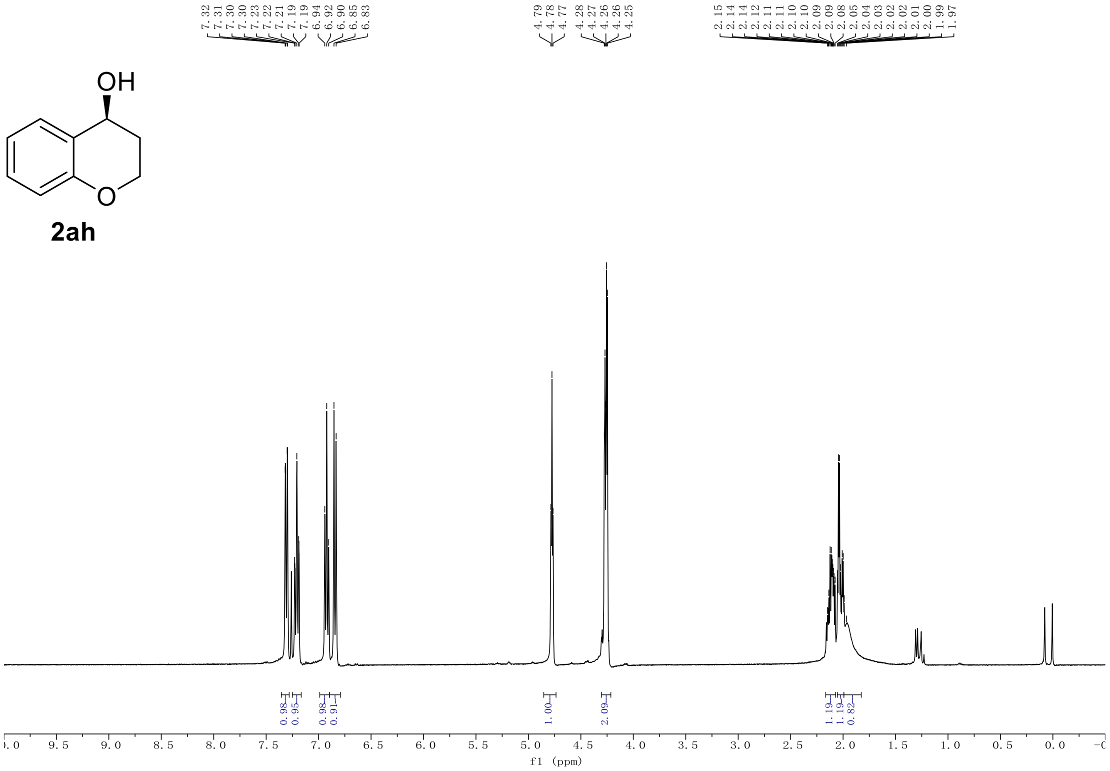

^{1}H NMR spectra (400 MHz, $CDCl_3$) of **2ah**

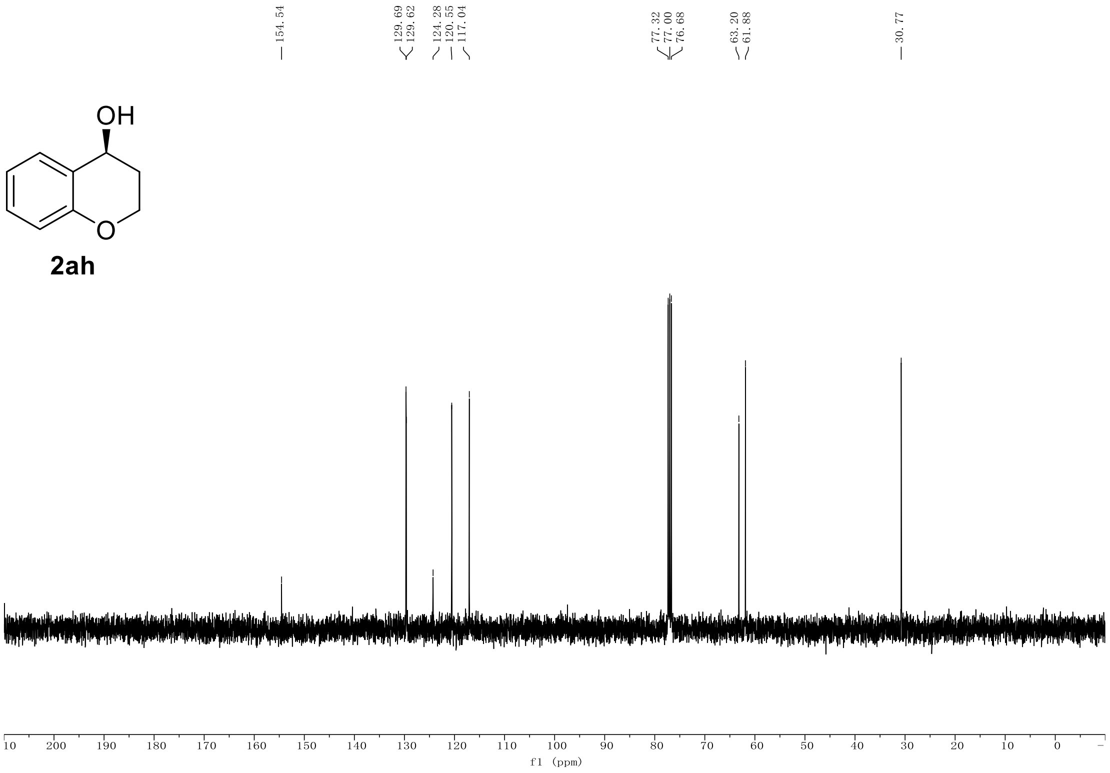

^{13}C NMR spectra (101 MHz, $CDCl_3$) of **2ah**

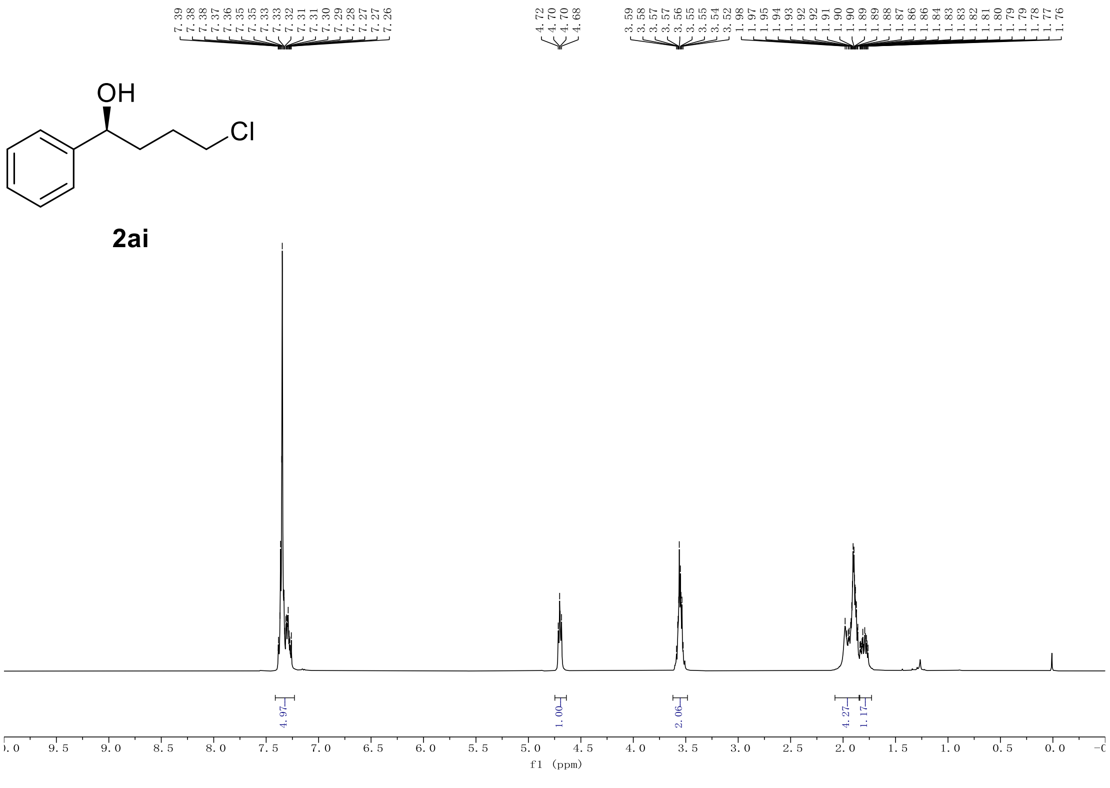

^{1}H NMR spectra (400 MHz, $CDCl_3$) of **2ai**

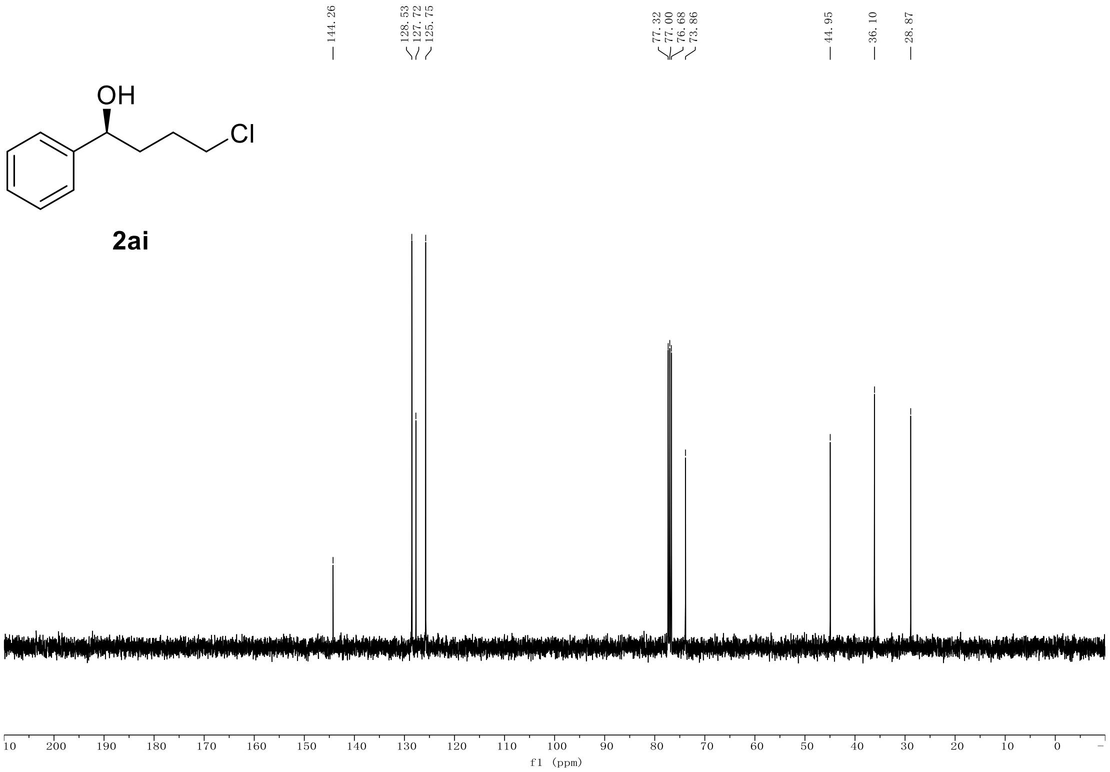

^{13}C NMR spectra (101 MHz, $CDCl_3$) of **2ai**

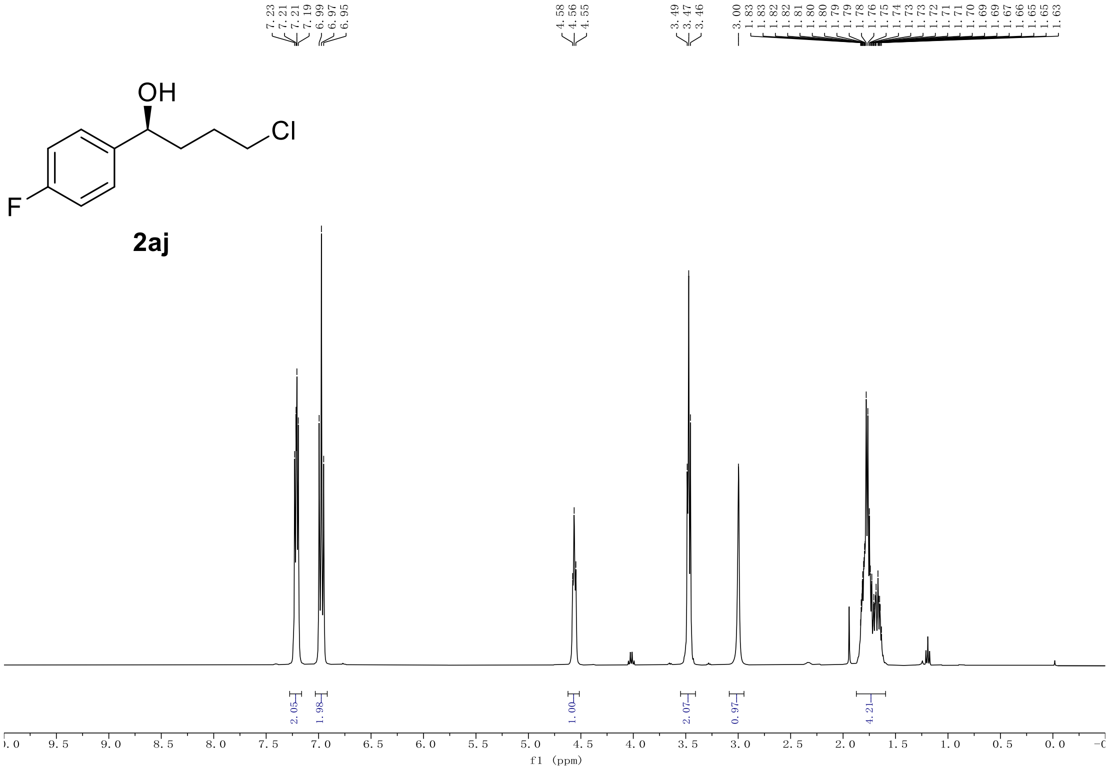

^{1}H NMR spectra (400 MHz, $CDCl_3$) of **2aj**

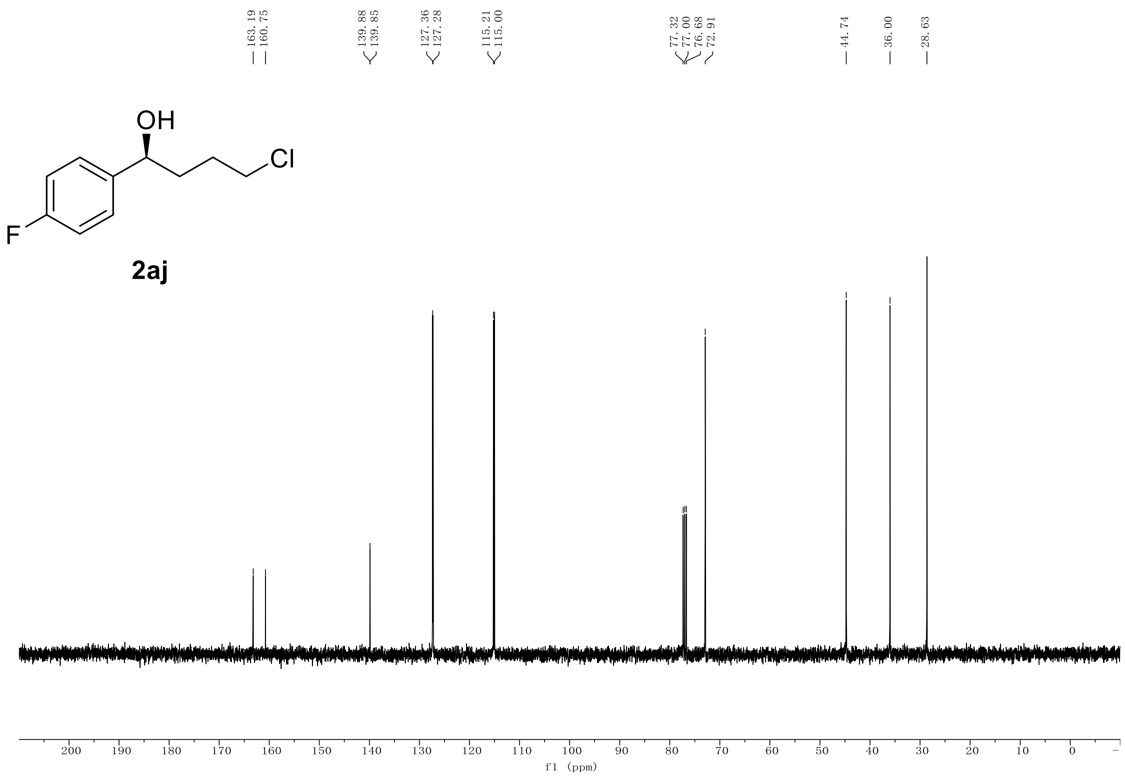

^{13}C NMR spectra (101 MHz, $CDCl_3$) of **2aj**

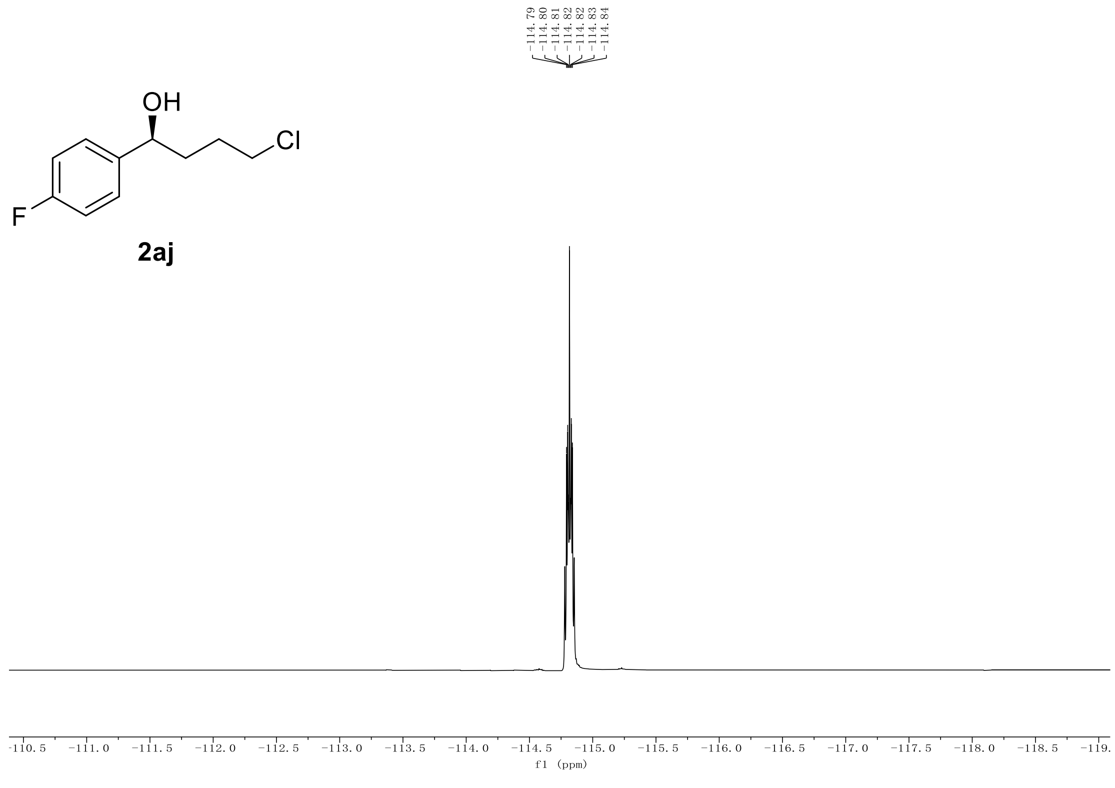

^{19}F NMR spectra (376 MHz, $CDCl_3$) of **2aj**

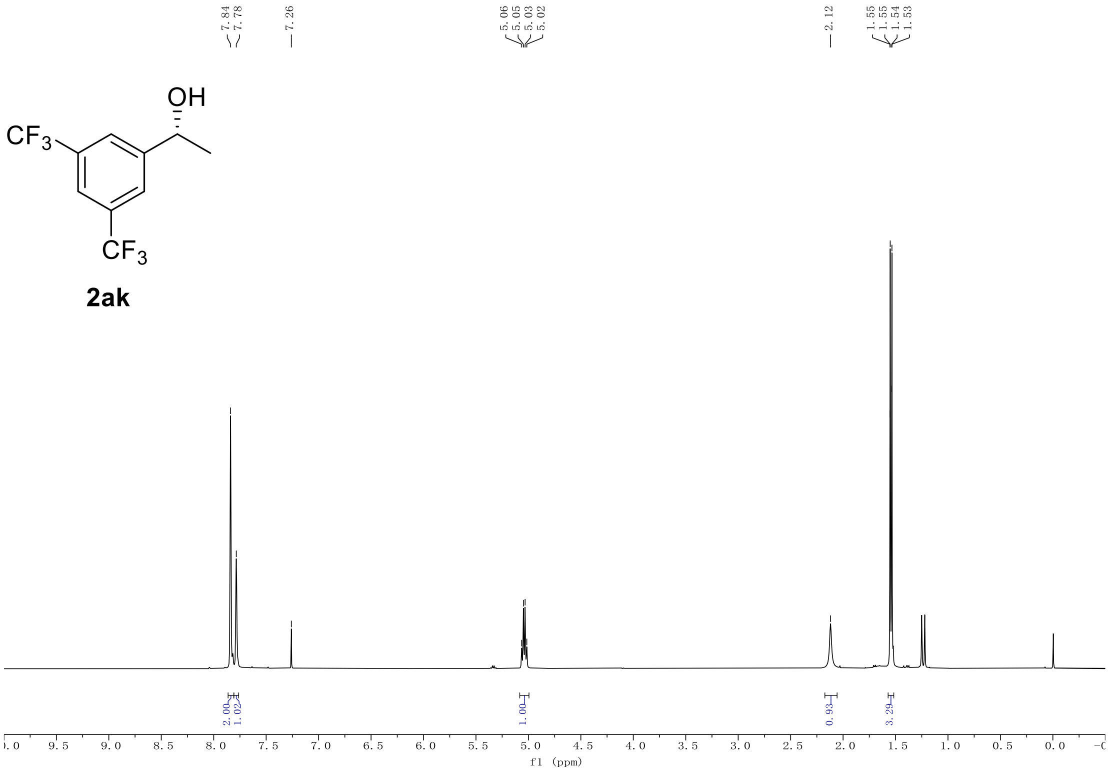

^{1}H NMR spectra (400 MHz, $CDCl_3$) of **2ak**

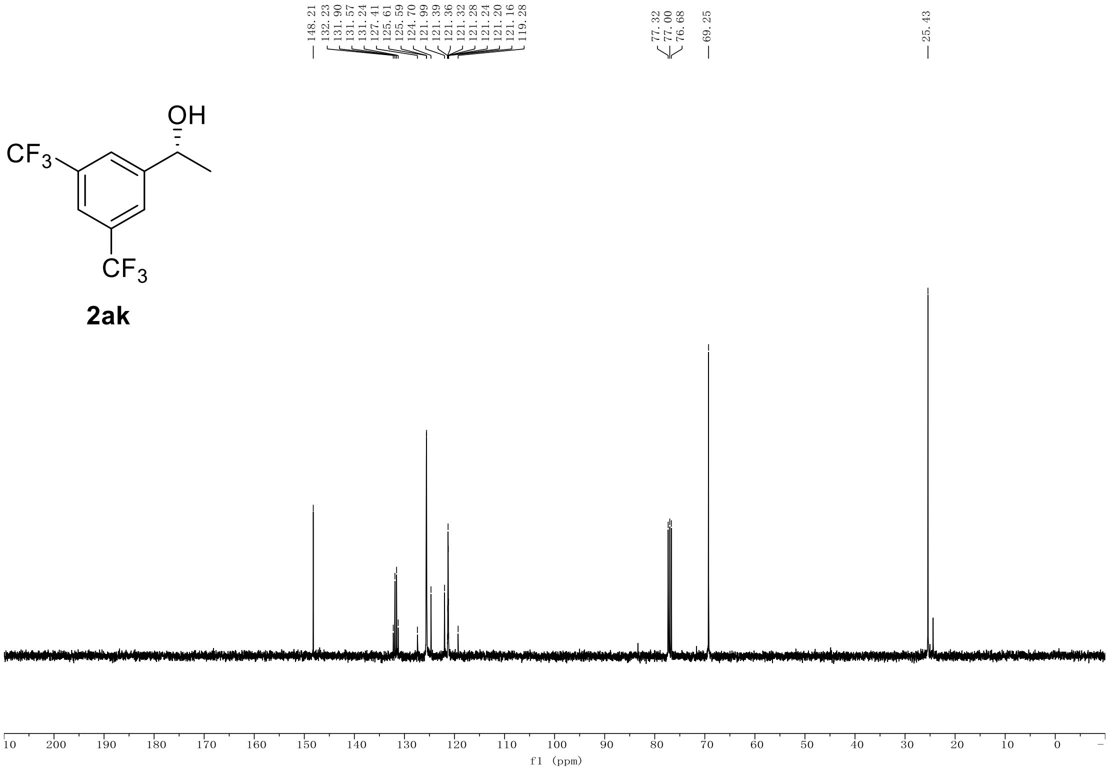

^{13}C NMR spectra (101 MHz, $CDCl_3$) of **2ak**

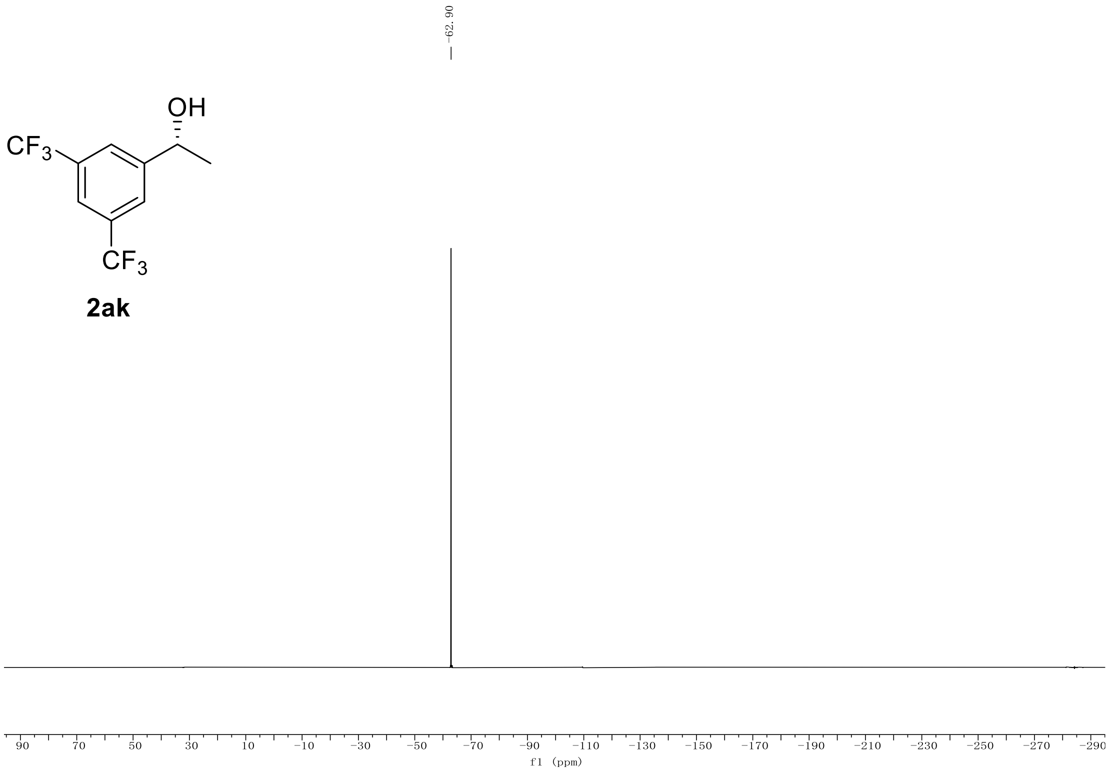

^{19}F NMR spectra (376 MHz, $CDCl_3$) of **2ak**

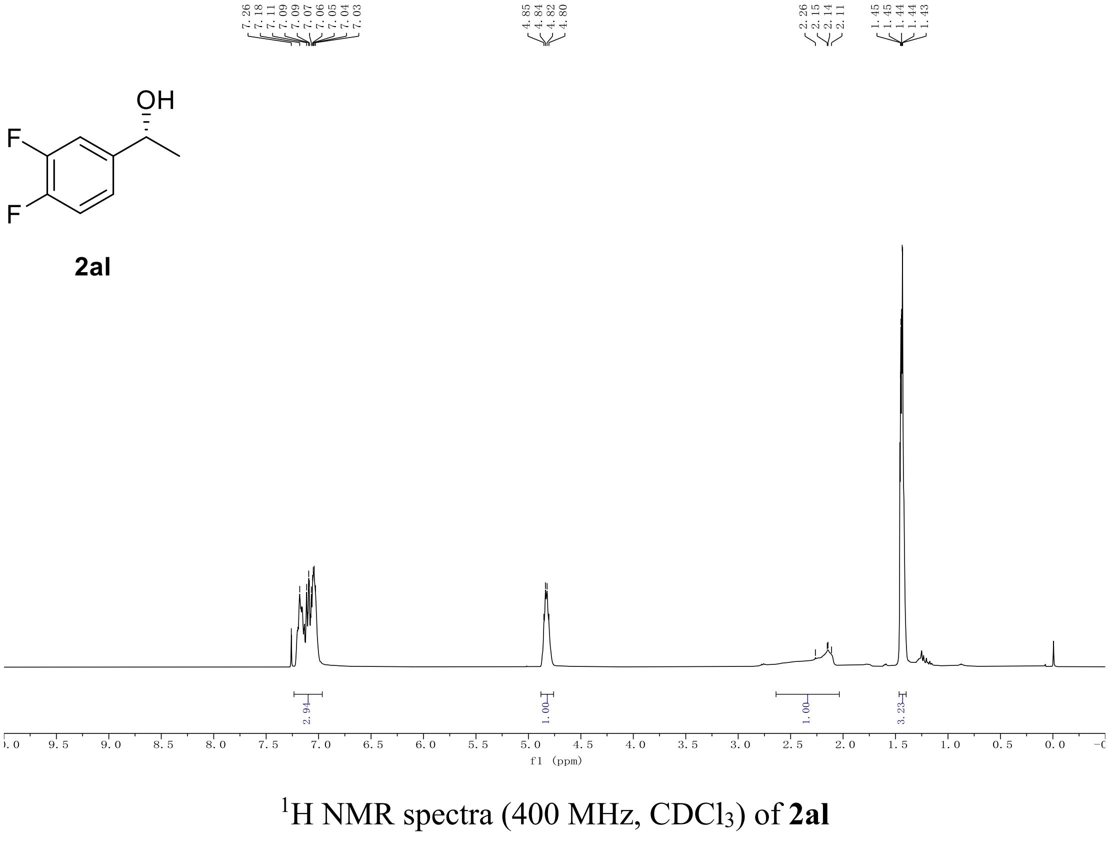

^{1}H NMR spectra (400 MHz, $CDCl_3$) of **2al**

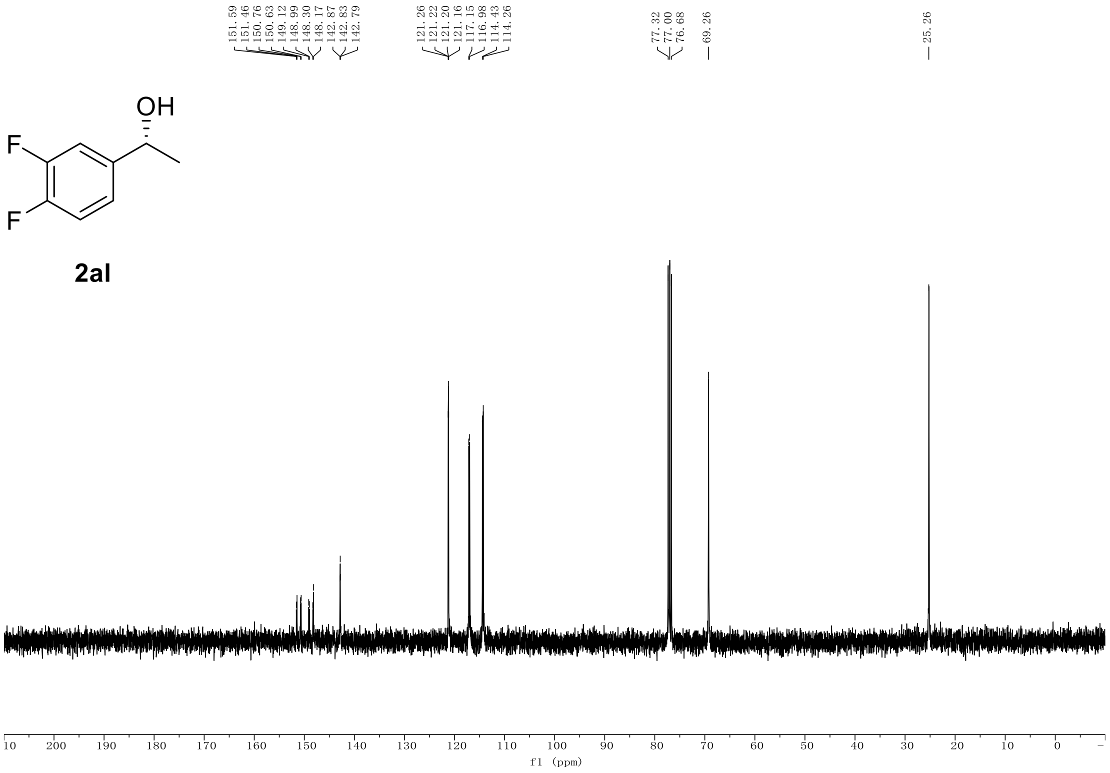

^{13}C NMR spectra (101 MHz, $CDCl_3$) of **2al**

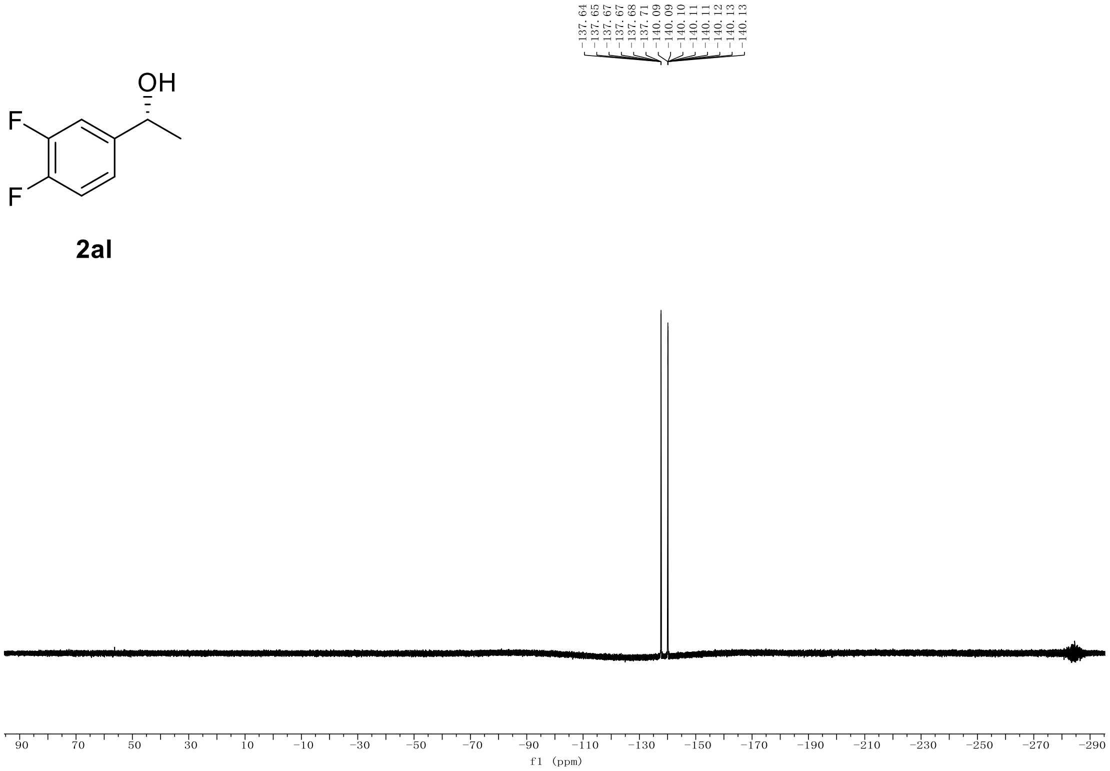

^{19}F NMR spectra (376 MHz, $CDCl_3$) of **2al**

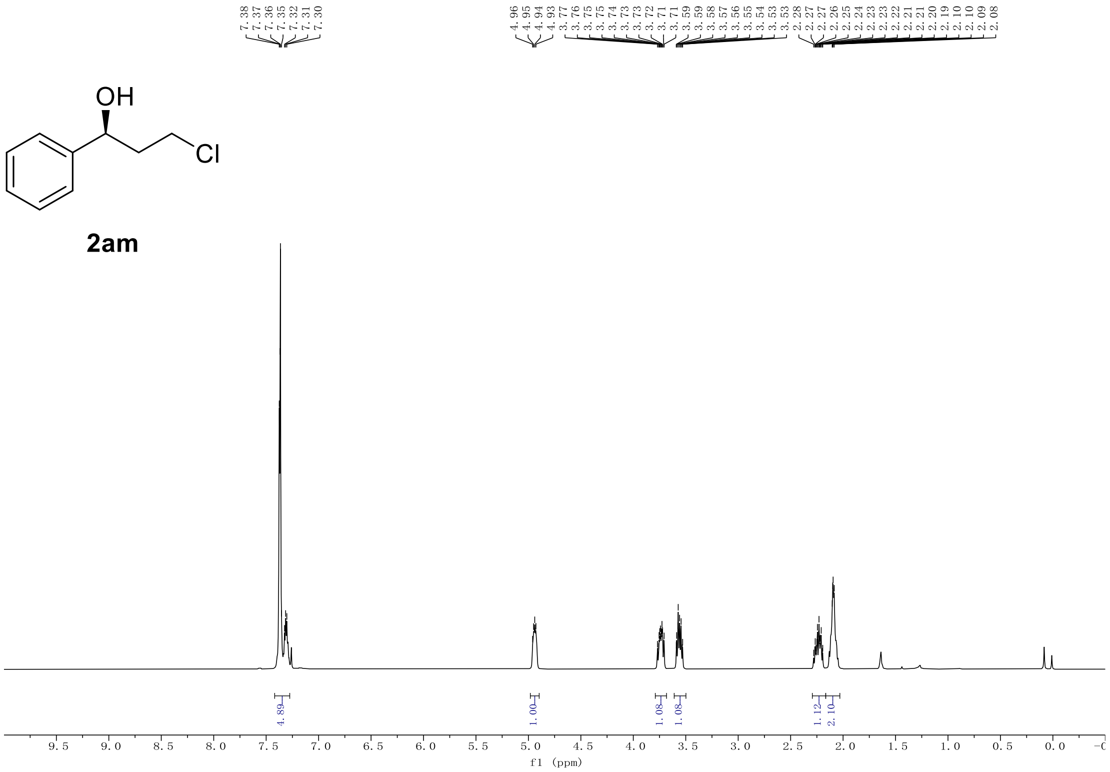

^{1}H NMR spectra (400 MHz, $CDCl_3$) of **2am**

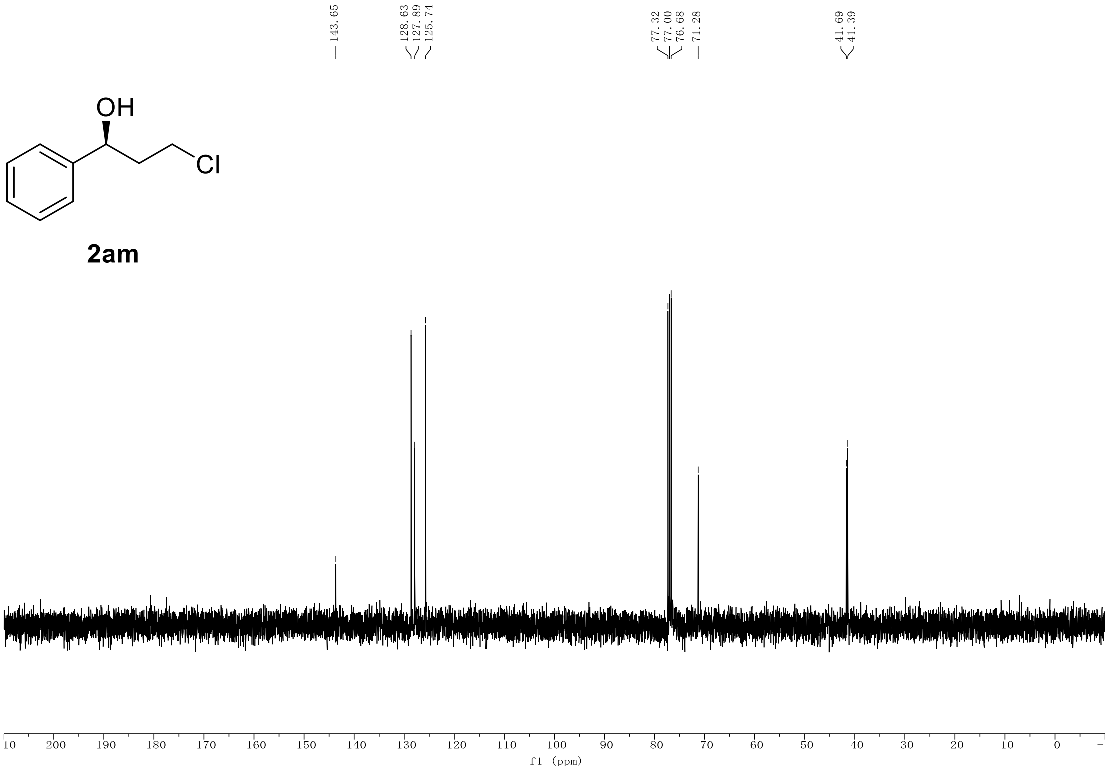

^{13}C NMR spectra (101 MHz, $CDCl_3$) of **2an**

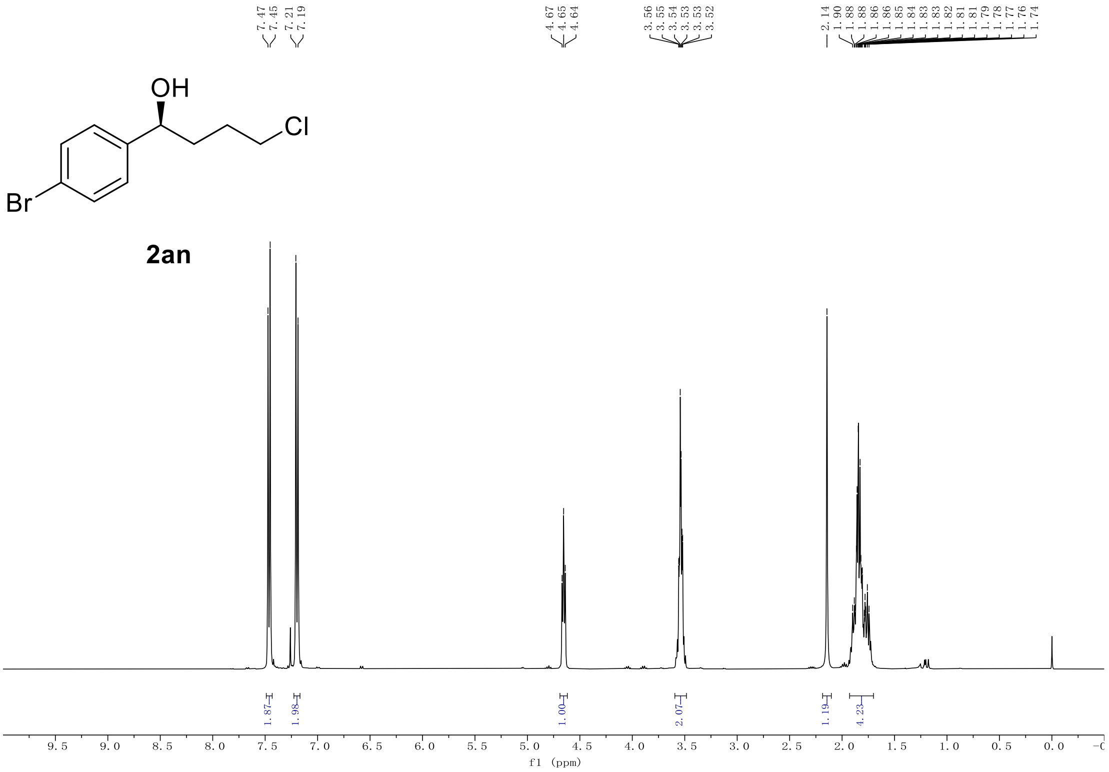

^{1}H NMR spectra (400 MHz, $CDCl_3$) of **2an**

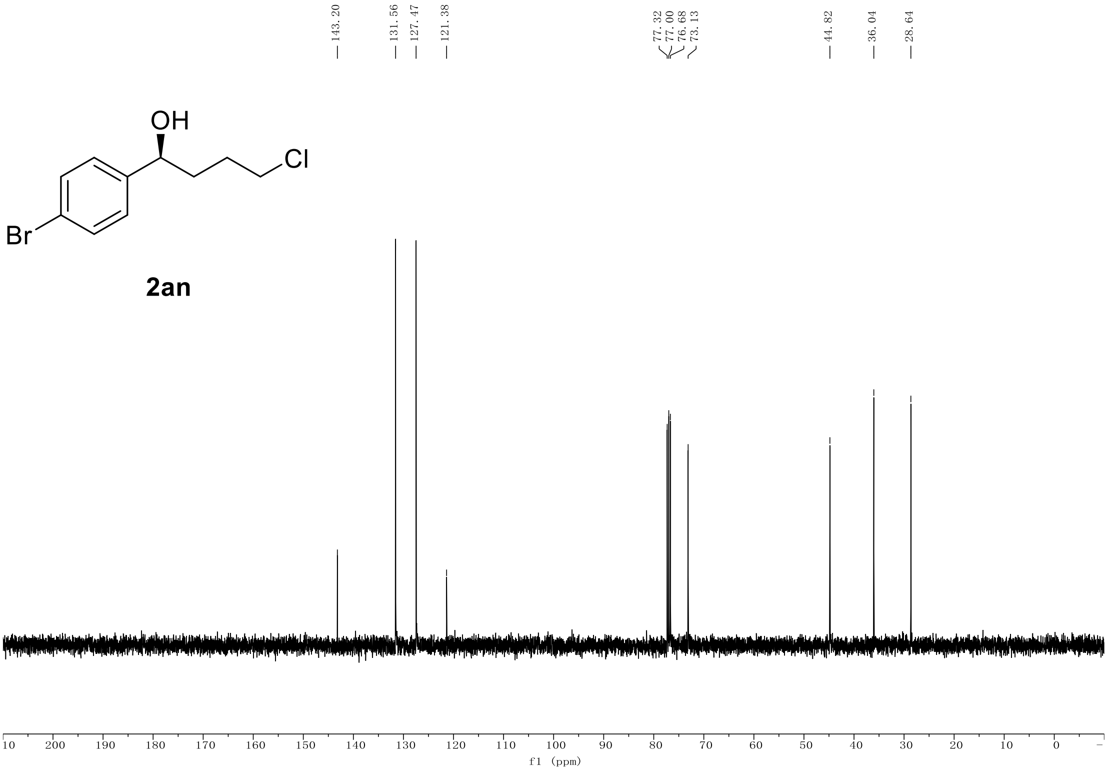

^{13}C NMR spectra (101 MHz, $CDCl_3$) of **2an**

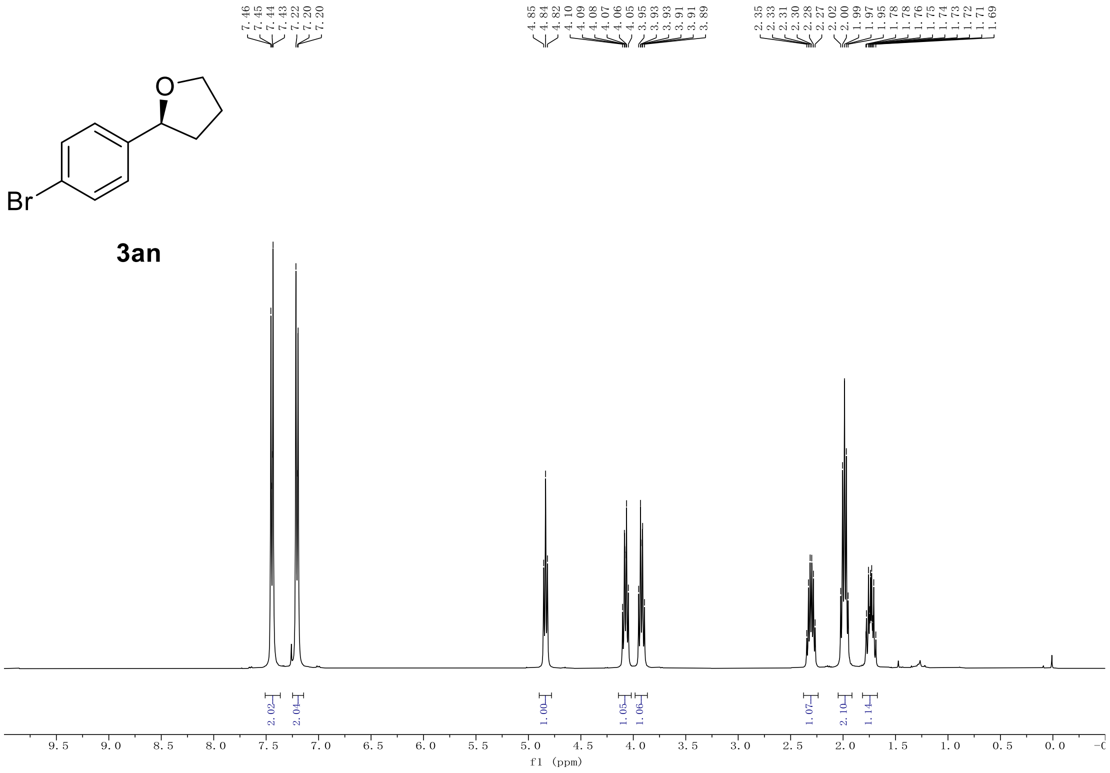

^{1}H NMR spectra (400 MHz, $CDCl_3$) of **3an**

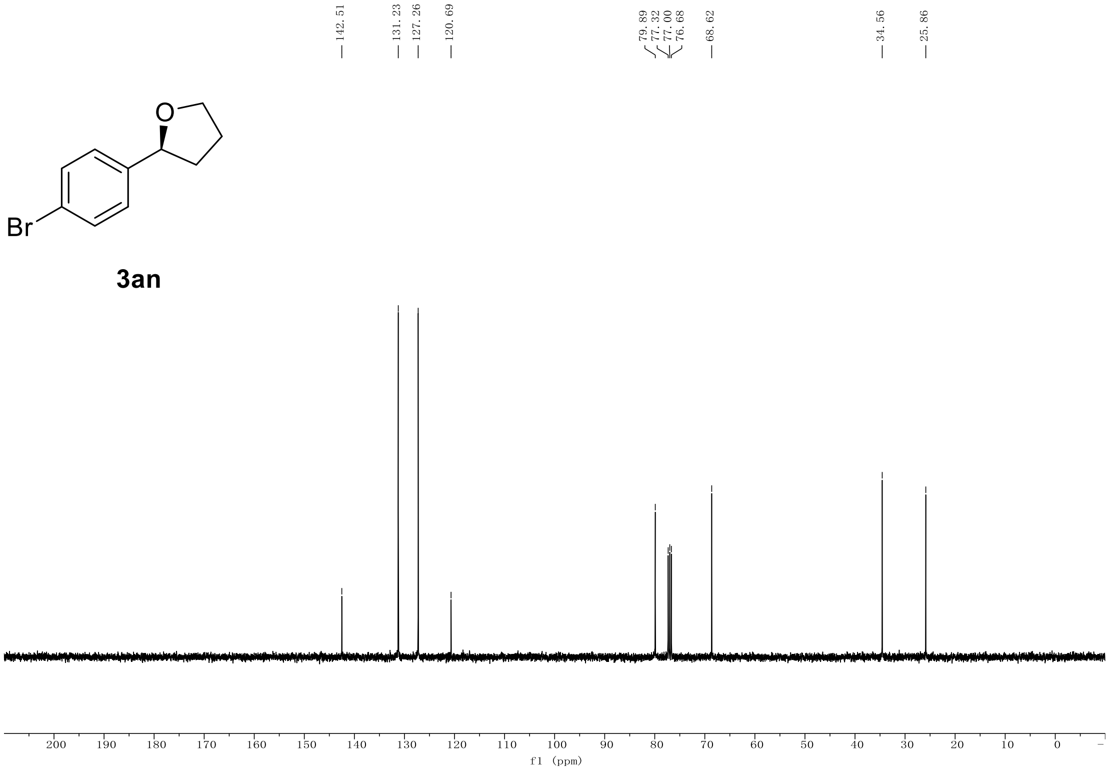

^{13}C NMR spectra (101 MHz, $CDCl_3$) of **3an**

6. HPLC Chromatograms

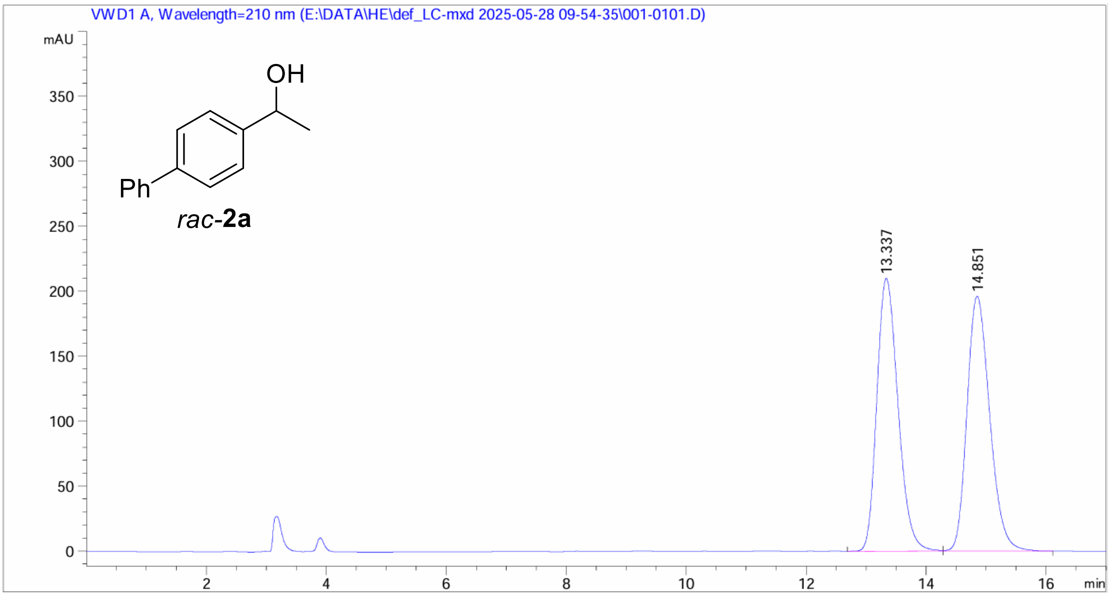

峰 #	保留时间 [min]	类型	峰宽 [min]	峰面积 [mAU*s]	峰高 [mAU]	峰面积 %
1	13.337	BV	0.3825	5155.87207	209.95738	49.9543
2	14.851	VB	0.4091	5165.31250	196.23672	50.0457

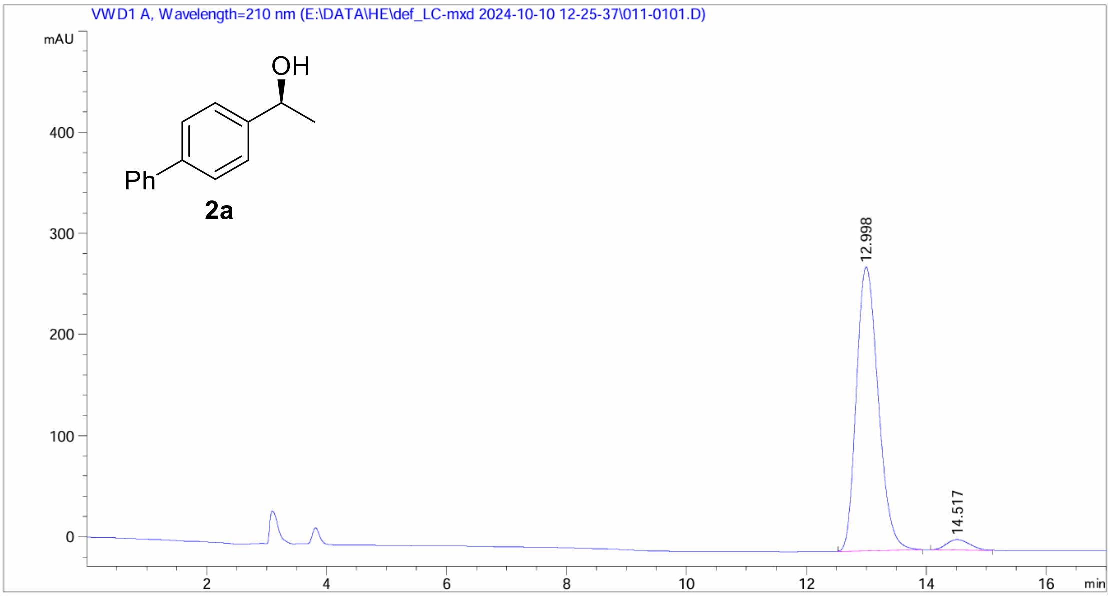

峰 #	保留时间 [min]	类型	峰宽 [min]	峰面积 [mAU*s]	峰高 [mAU]	峰面积 %
1	12.998	MM R	0.4219	7102.35352	280.55557	96.2617
2	14.517	MM R	0.4483	275.82050	10.25356	3.7383

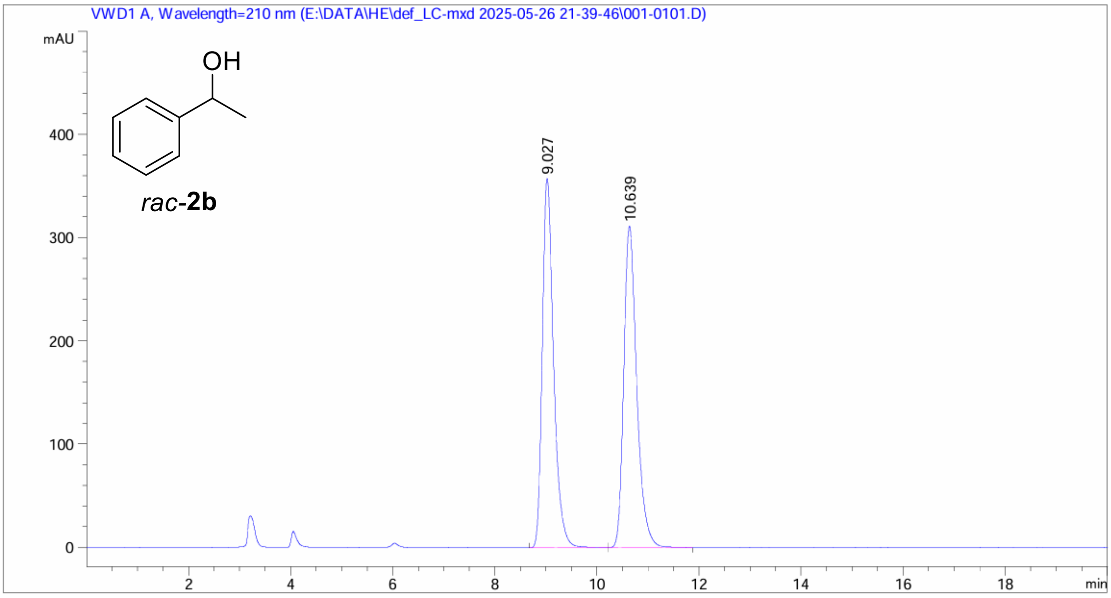

峰 #	保留时间 [min]	类型	峰宽 [min]	峰面积 [mAU*s]	峰高 [mAU]	峰面积 %
1	9.027	BB	0.2336	5505.20801	357.64523	50.0951
2	10.639	BB	0.2676	5484.31299	311.17288	49.9049

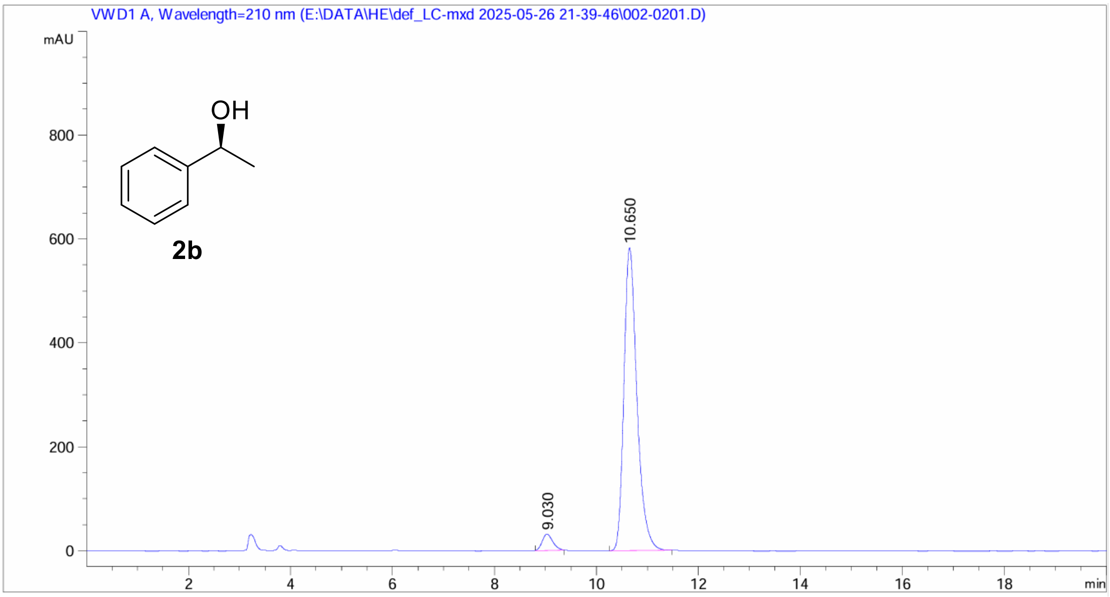

峰 #	保留时间 [min]	类型	峰宽 [min]	峰面积 [mAU*s]	峰高 [mAU]	峰面积 %
1	9.030	MM R	0.2378	444.79309	31.17155	4.1268
2	10.650	MM R	0.2956	1.03333e4	582.66302	95.8732

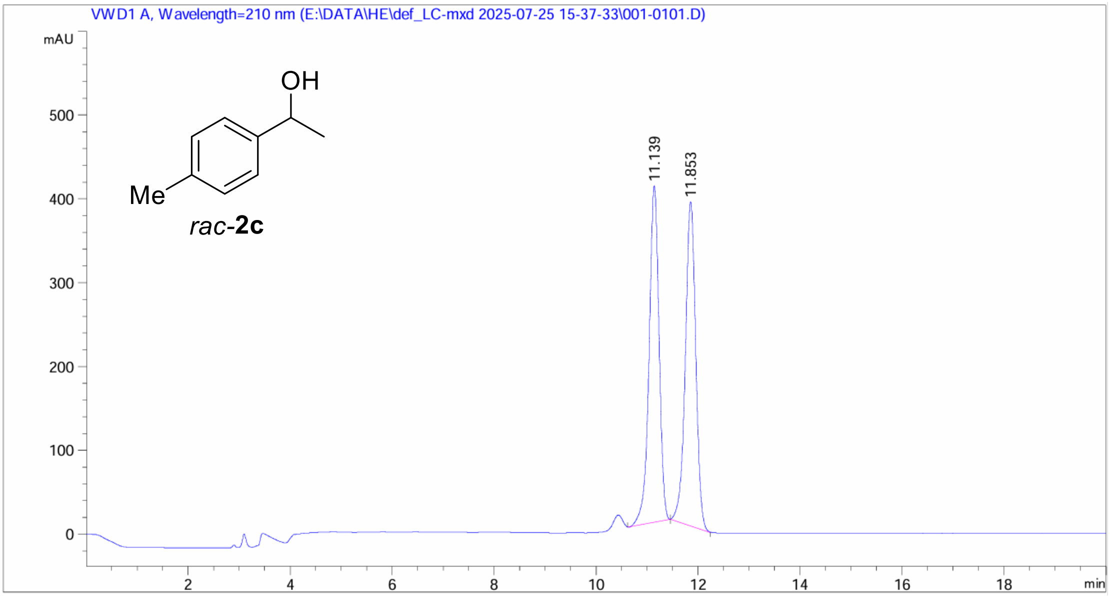

峰 #	保留时间 [min]	类型	峰宽 [min]	峰面积 [mAU*s]	峰高 [mAU]	峰面积 %
1	11.139	MM R	0.2264	5455.18457	401.56799	49.8404
2	11.853	MM R	0.2364	5490.12744	387.04291	50.1596

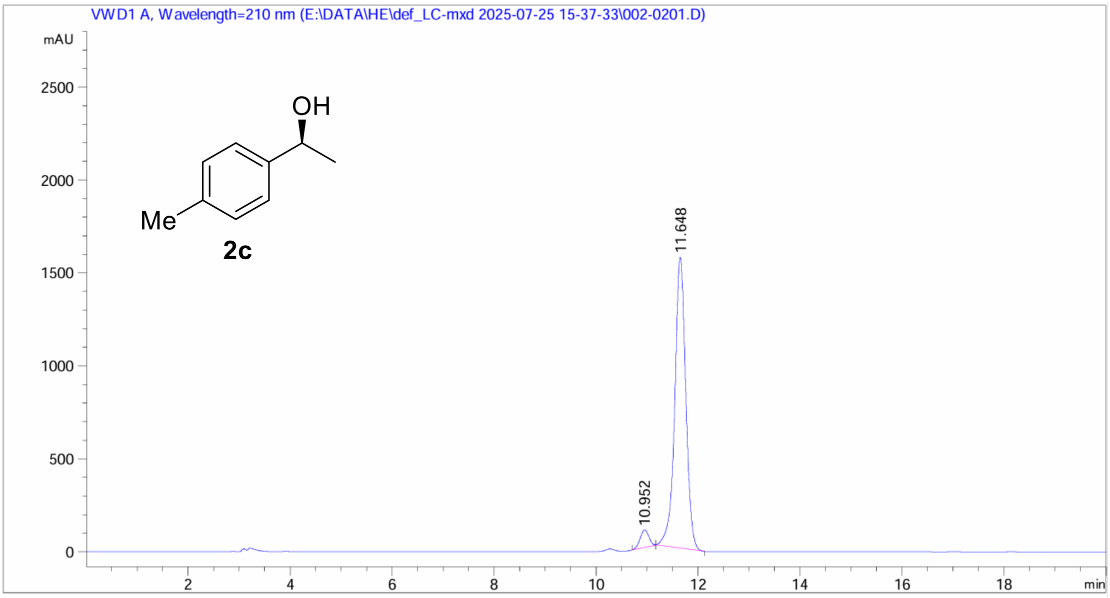

峰 #	保留时间 [min]	类型	峰宽 [min]	峰面积 [mAU*s]	峰高 [mAU]	峰面积 %
1	10.952	MM R	0.1969	1102.75940	93.33635	4.4635
2	11.648	MM R	0.2512	2.36037e4	1565.84180	95.5365

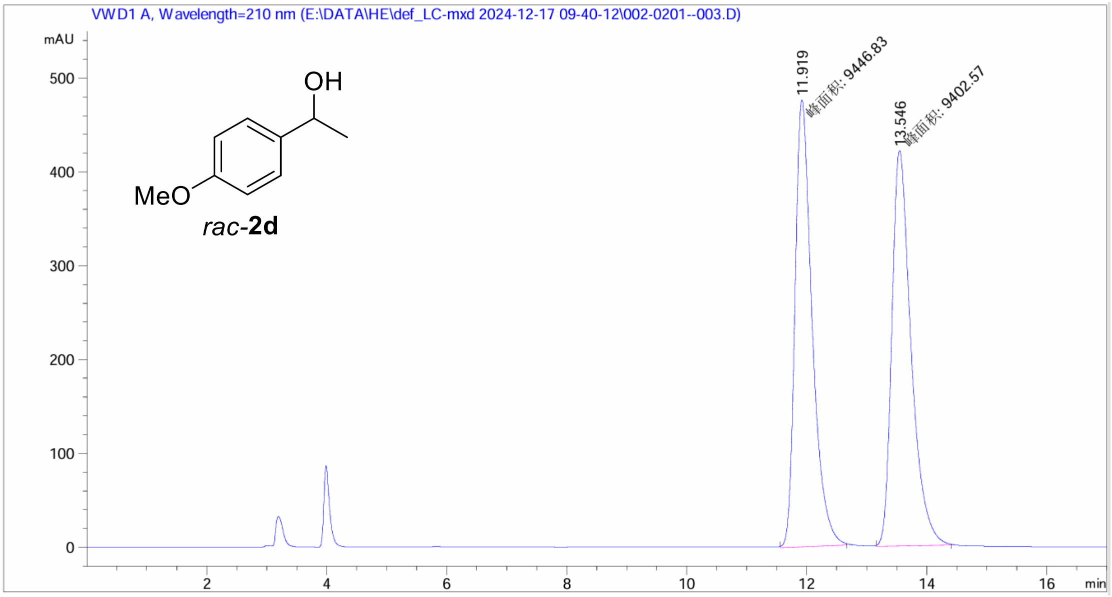

峰 #	保留时间 [min]	类型	峰宽 [min]	峰面积 [mAU*s]	峰高 [mAU]	峰面积 %
1	11.919	MM	0.3306	9446.82520	476.22711	50.1174
2	13.546	MM	0.3720	9402.56543	421.28061	49.8826

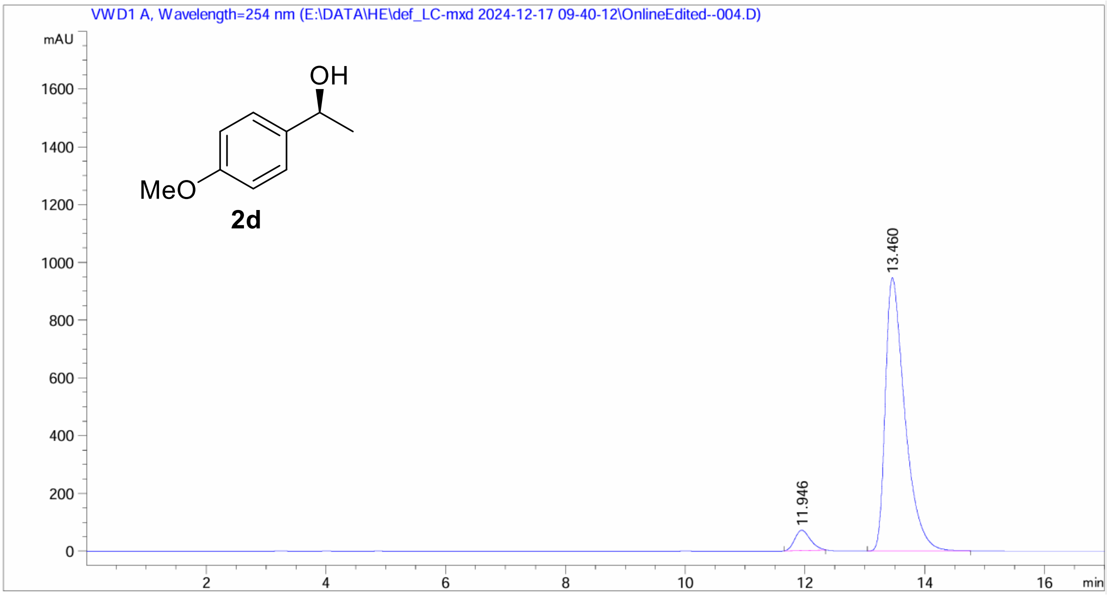

峰 #	保留时间 [min]	类型	峰宽 [min]	峰面积 [mAU*s]	峰高 [mAU]	峰面积 %
1	11.946	MM R	0.2996	1269.42334	70.62825	5.5763
2	13.460	MM R	0.3787	2.14954e4	946.04816	94.4237

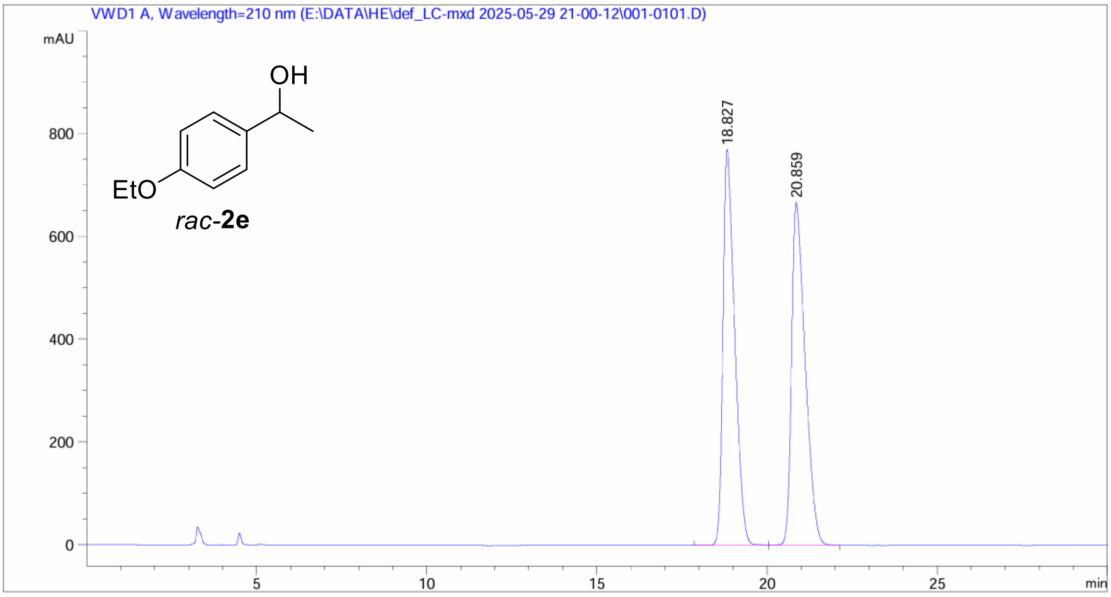

峰 #	保留时间 [min]	类型	峰宽 [min]	峰面积 [mAU*s]	峰高 [mAU]	峰面积 %
1	18.827	BB	0.3571	1.83503e4	769.70081	49.8482
2	20.859	BB	0.4138	1.84621e4	667.33990	50.1518

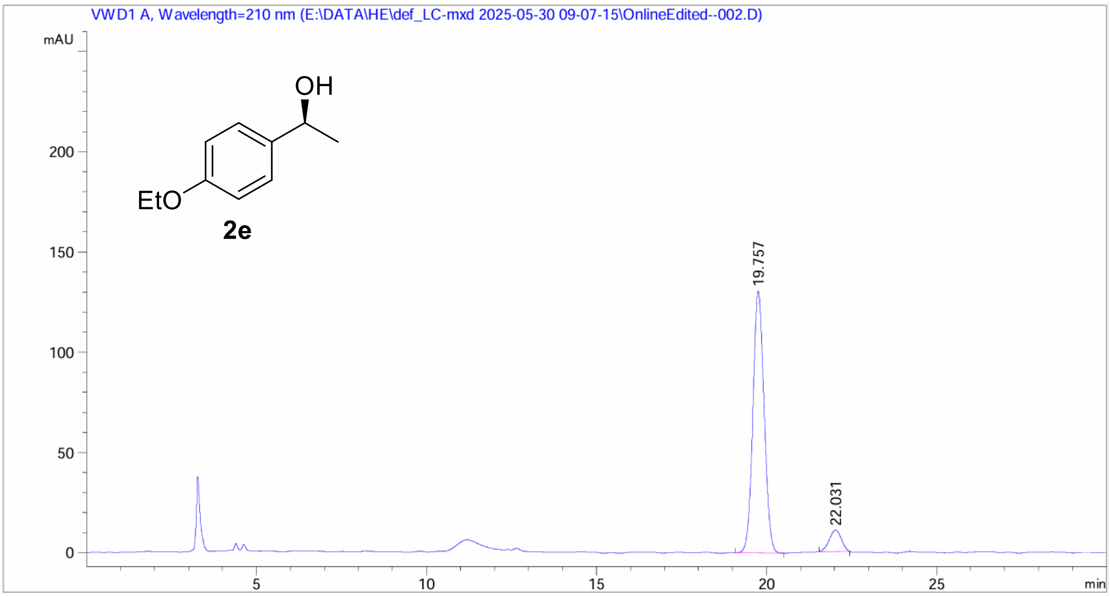

峰 #	保留时间 [min]	类型	峰宽 [min]	峰面积 [mAU*s]	峰高 [mAU]	峰面积 %
1	19.757	BB	0.3551	2991.23267	130.64845	91.9153
2	22.031	MM R	0.4093	263.10495	10.71238	8.0847

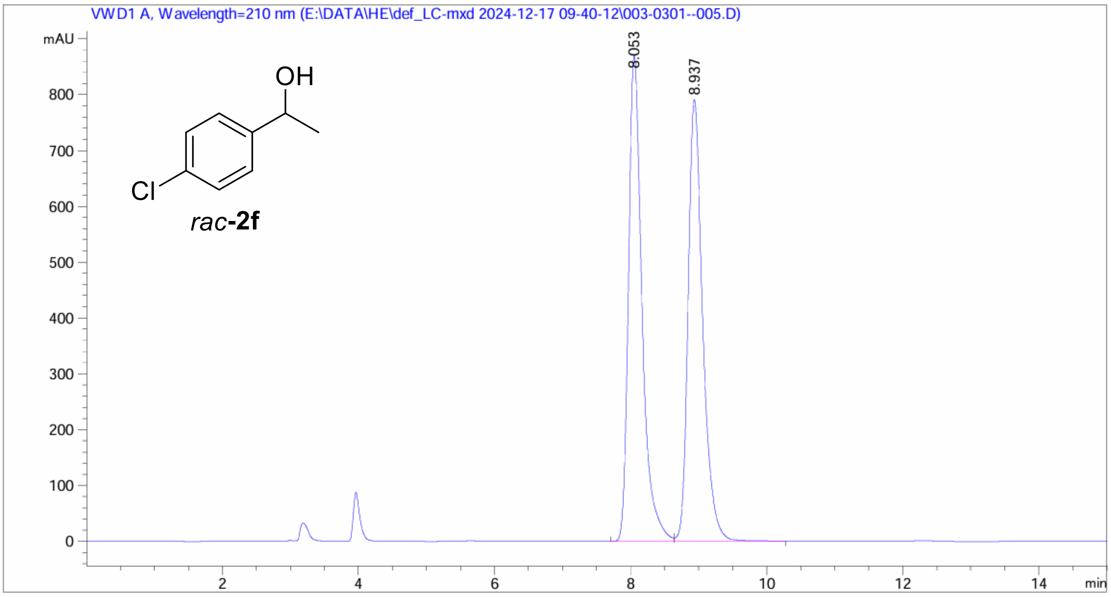

峰 #	保留时间 [min]	类型	峰宽 [min]	峰面积 [mAU*s]	峰高 [mAU]	峰面积 %
1	8.053	BV	0.2082	1.20254e4	870.32770	50.4775
2	8.937	VB	0.2250	1.17979e4	791.17279	49.5225

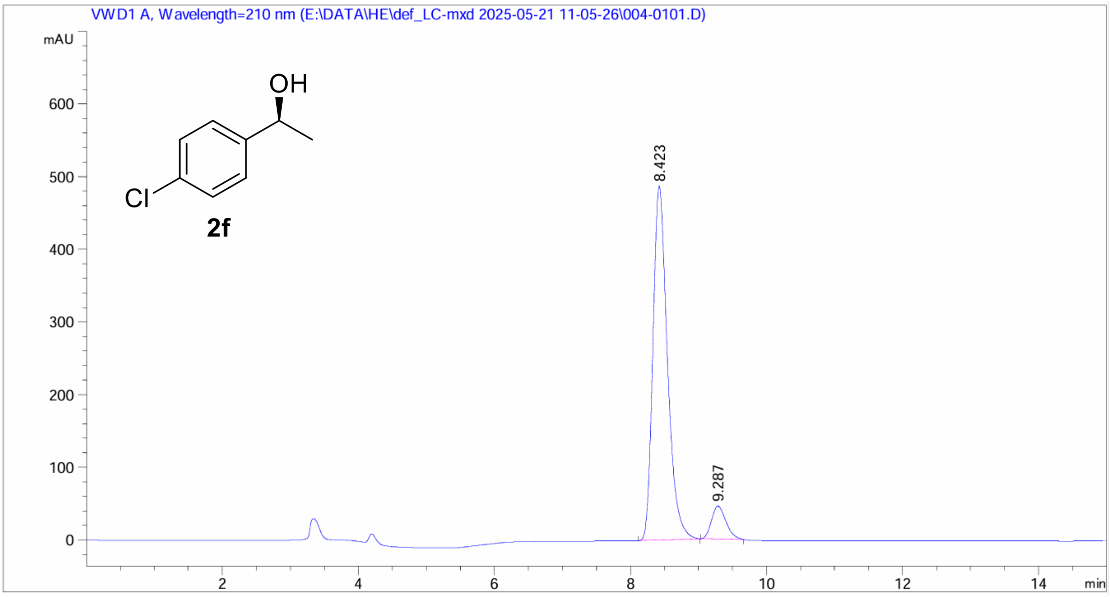

峰 #	保留时间 [min]	类型	峰宽 [min]	峰面积 [mAU*s]	峰高 [mAU]	峰面积 %
1	8.423	MM R	0.2470	7224.27539	487.39816	91.6148
2	9.287	MM R	0.2436	661.21753	45.23026	8.3852

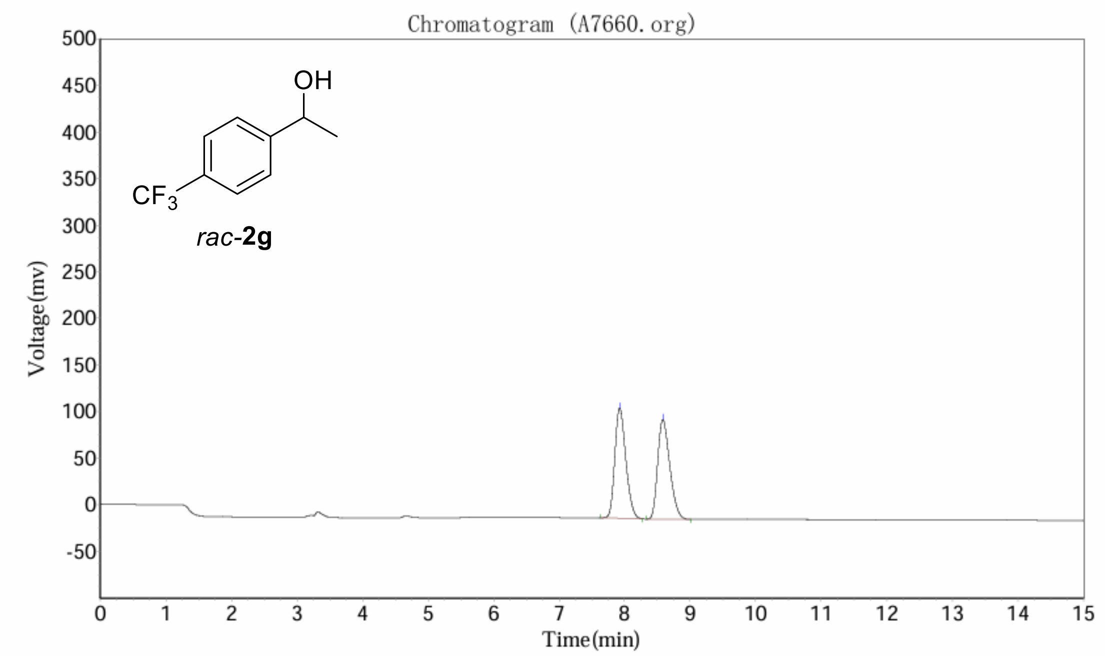

Results

Peak No.	Peak ID	Ret Time	Height	Area	Conc.
1		7.925	118278.602	1378576.625	50.1867
2		8.583	106709.828	1368320.375	49.8133
Total			224988.430	2746897.000	100.0000

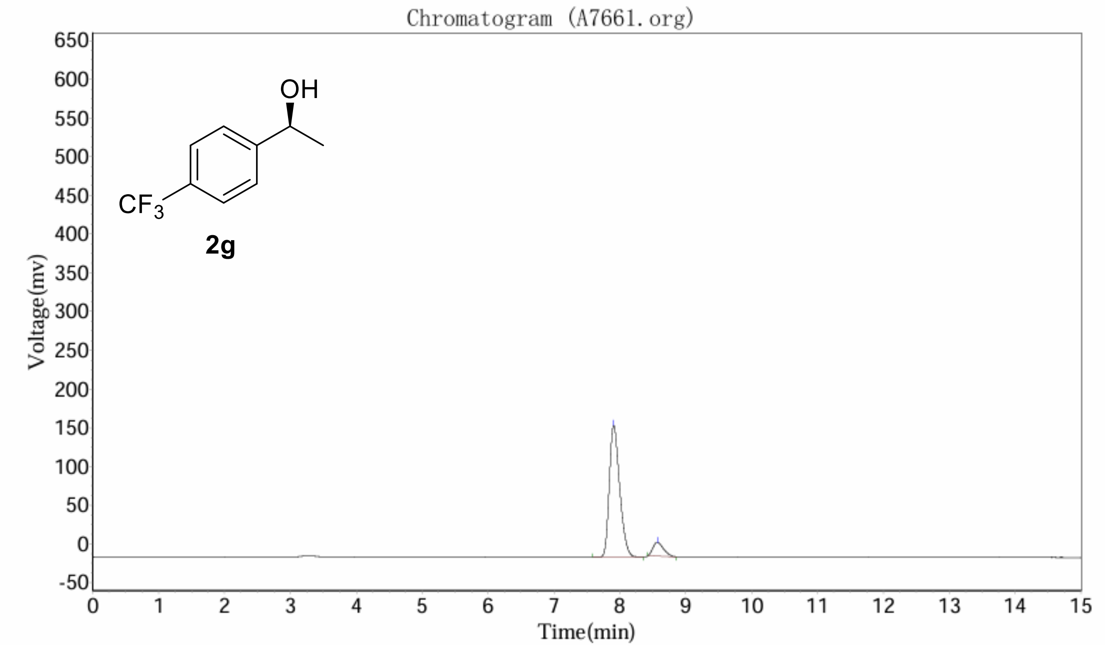

Results

Peak No.	Peak ID	Ret Time	Height	Area	Conc.
1		7.907	170176.672	1858822.750	90.1399
2		8.568	17955.352	203330.703	9.8601
Total			188132.023	2062153.453	100.0000

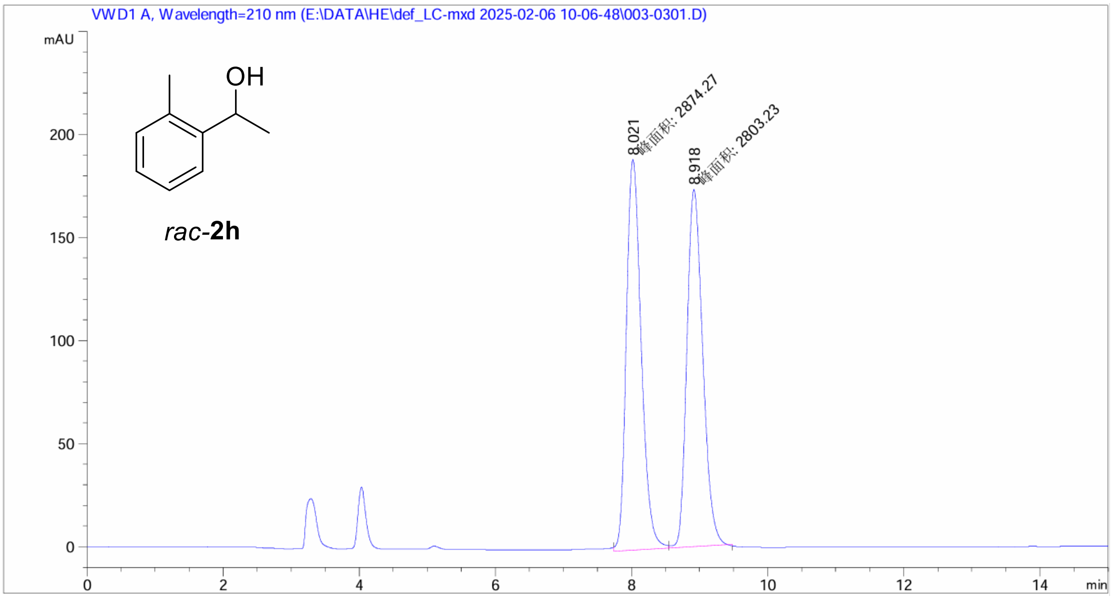

峰 #	保留时间 [min]	类型	峰宽 [min]	峰面积 [mAU*s]	峰高 [mAU]	峰面积 %
1	8.021	MF	0.2527	2874.27051	189.55008	50.6257
2	8.918	FM	0.2694	2803.22607	173.40982	49.3743

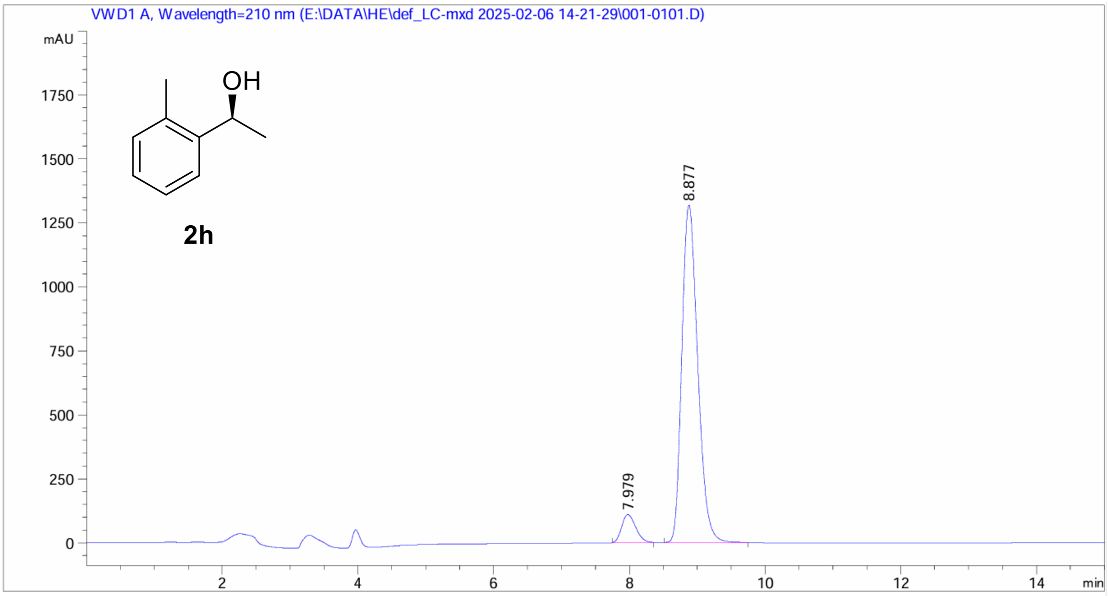

峰 #	保留时间 [min]	类型	峰宽 [min]	峰面积 [mAU*s]	峰高 [mAU]	峰面积 %
1	7.979	MM R	0.2395	1570.97534	109.34072	6.8330
2	8.877	MM R	0.2708	2.14199e4	1318.49023	93.1670

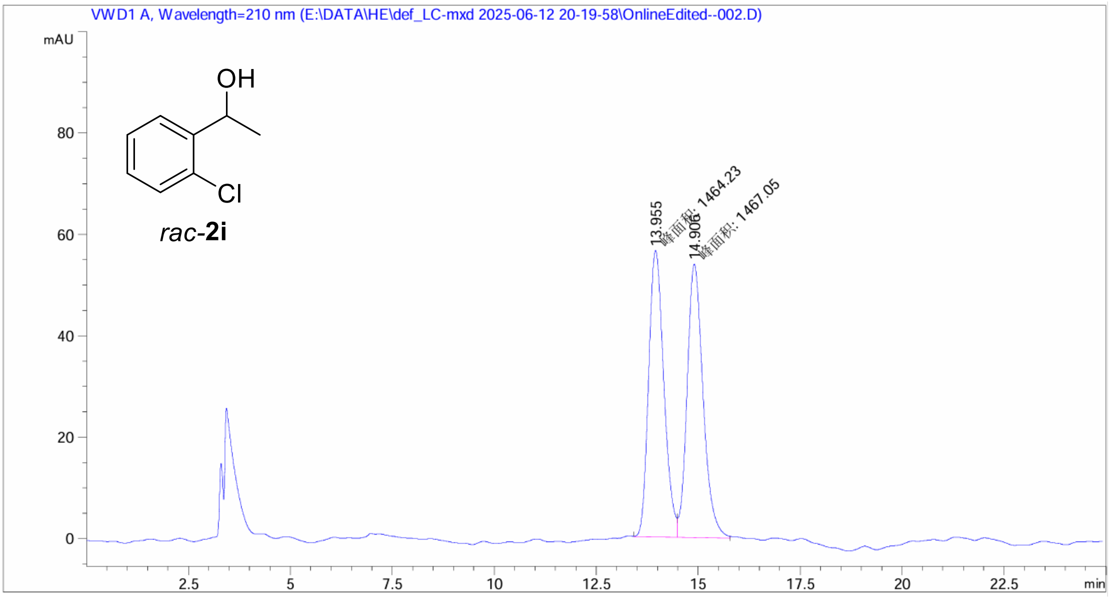

峰 #	保留时间 [min]	类型	峰宽 [min]	峰面积 [mAU*s]	峰高 [mAU]	峰面积 %
1	13.955	MF	0.4324	1464.23254	56.43230	49.9520
2	14.906	FM	0.4537	1467.04736	53.89335	50.0480

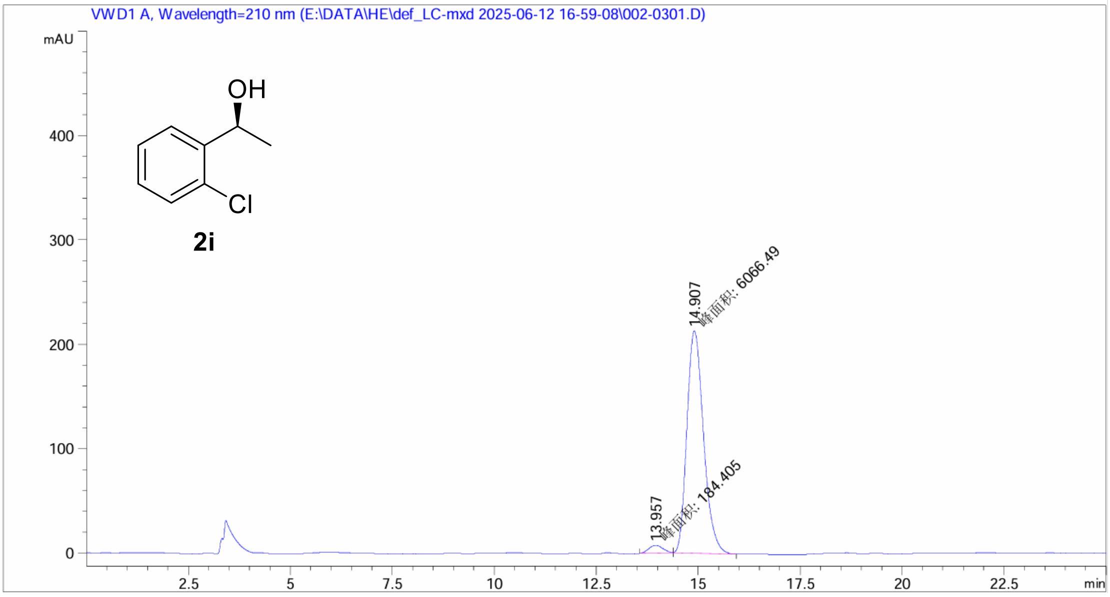

峰 #	保留时间 [min]	类型	峰宽 [min]	峰面积 [mAU*s]	峰高 [mAU]	峰面积 %
1	13.957	MM	0.4043	184.40454	7.60153	2.9501
2	14.907	MM	0.4743	6066.48975	213.18721	97.0499

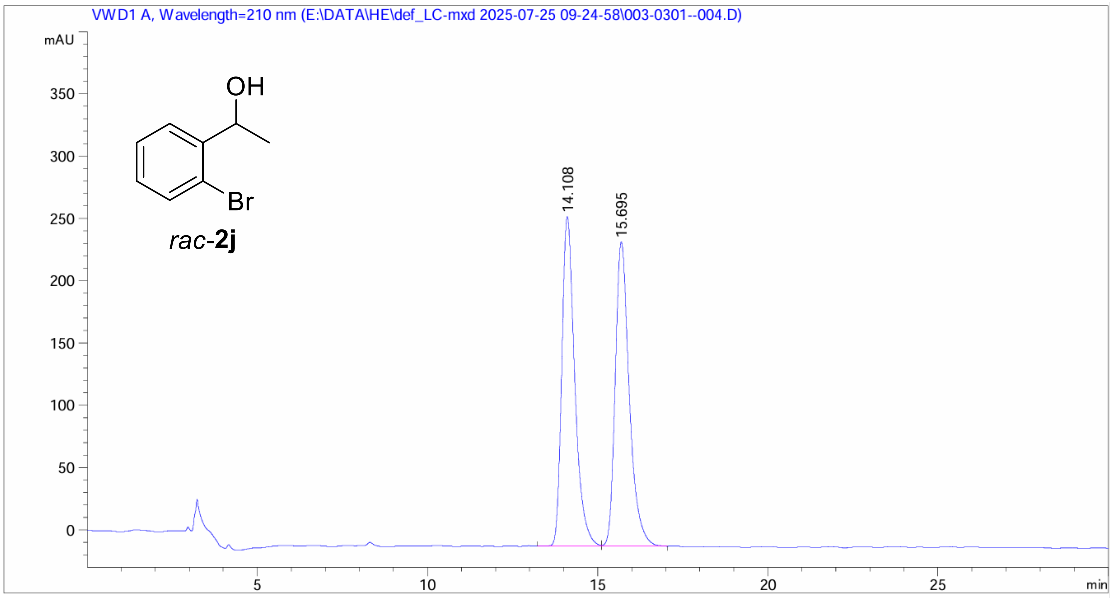

峰 #	保留时间 [min]	类型	峰宽 [min]	峰面积 [mAU*s]	峰高 [mAU]	峰面积 %
1	14.108	BV	0.4031	7010.81104	264.64066	49.9893
2	15.695	VB	0.4359	7013.80029	244.33531	50.0107

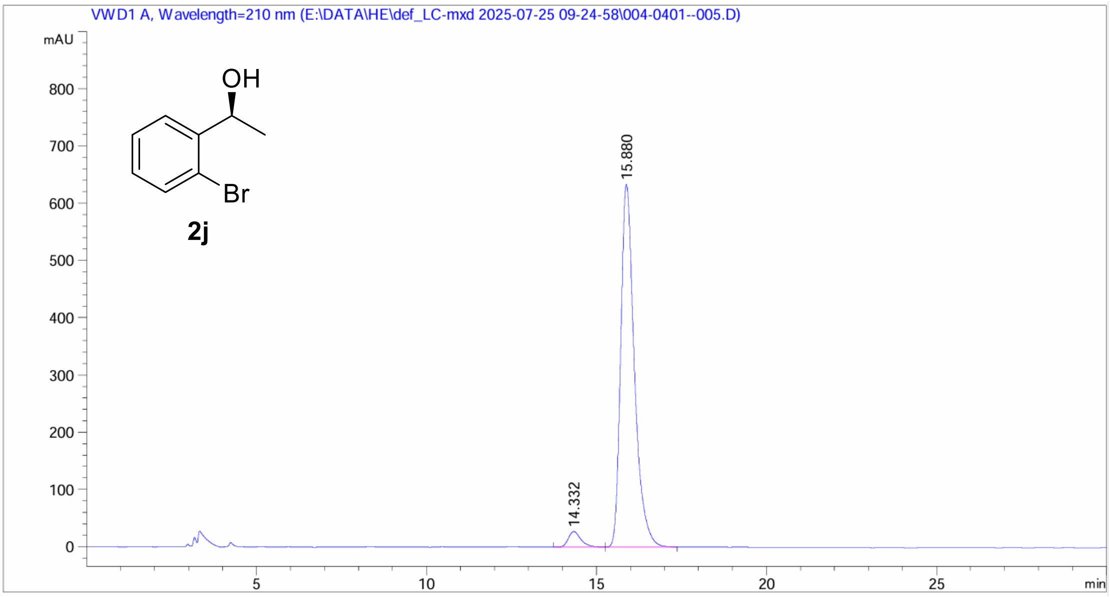

峰 #	保留时间 [min]	类型	峰宽 [min]	峰面积 [mAU*s]	峰高 [mAU]	峰面积 %
1	14.332	BB	0.3921	708.52393	27.35786	3.8566
2	15.880	BB	0.4222	1.76634e4	633.71393	96.1434

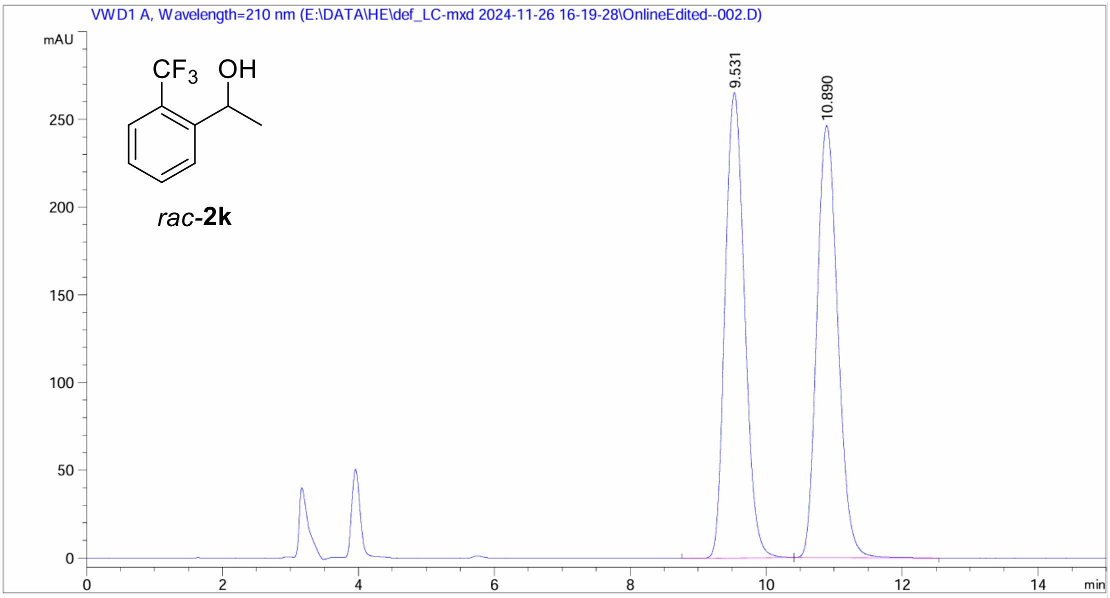

峰 #	保留时间 [min]	类型	峰宽 [min]	峰面积 [mAU*s]	峰高 [mAU]	峰面积 %
1	9.531	BB	0.3153	5274.19727	264.93365	50.0644
2	10.890	BB	0.3364	5260.63477	246.11716	49.9356

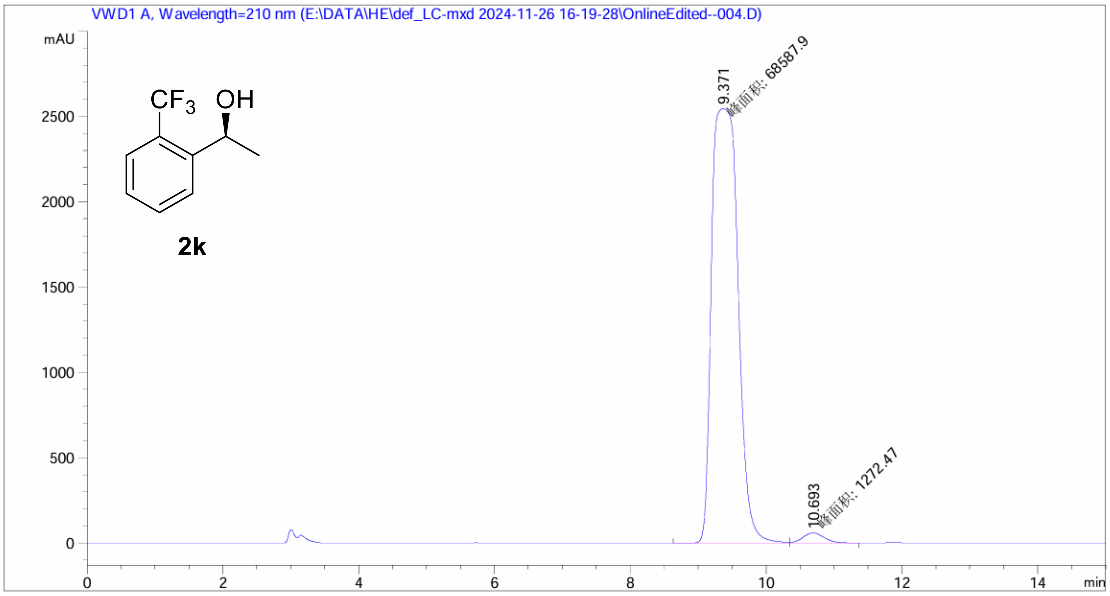

峰 #	保留时间 [min]	类型	峰宽 [min]	峰面积 [mAU*s]	峰高 [mAU]	峰面积 %
1	9.371	MM	0.4480	6.83943e4	2544.44824	98.3922
2	10.693	MM	0.3309	1117.58020	56.29349	1.6078

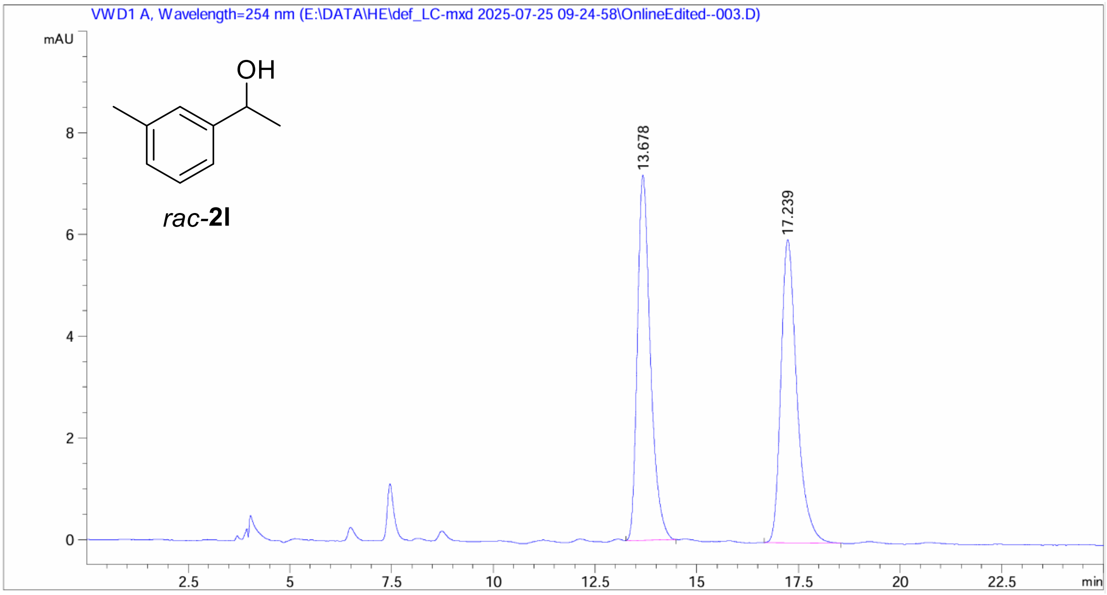

峰 #	保留时间 [min]	类型	峰宽 [min]	峰面积 [mAU*s]	峰高 [mAU]	峰面积 %
1	13.678	BB	0.3358	159.82521	7.17749	49.4447
2	17.239	BB	0.4140	163.41486	5.95871	50.5553

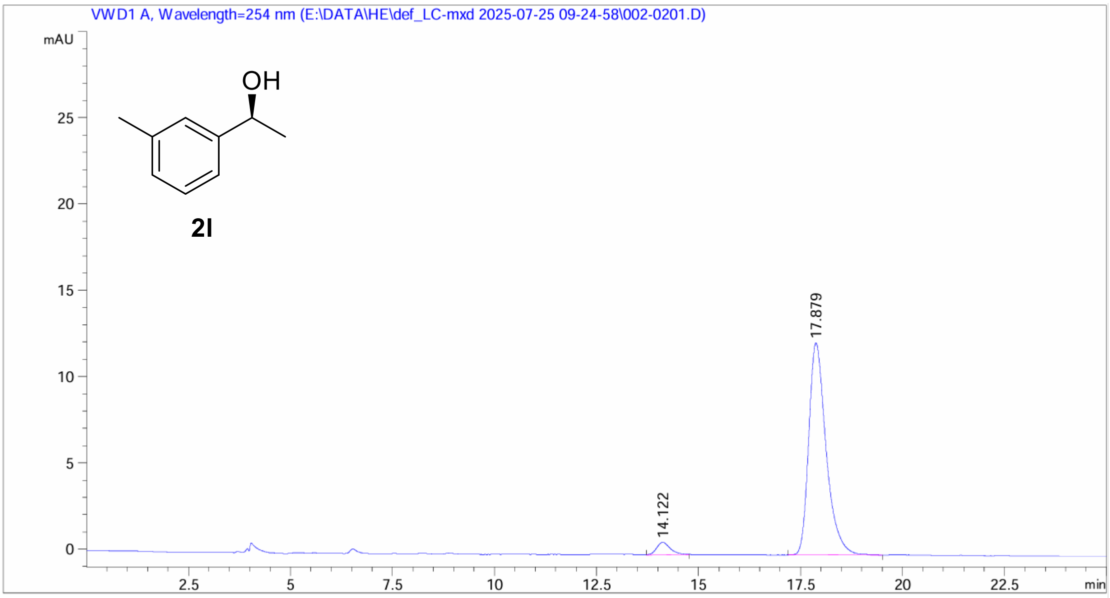

峰 #	保留时间 [min]	类型	峰宽 [min]	峰面积 [mAU*s]	峰高 [mAU]	峰面积 %
1	14.122	MM R	0.3888	16.83323	7.21603e-1	4.4327
2	17.879	BB	0.4466	362.91776	12.28576	95.5673

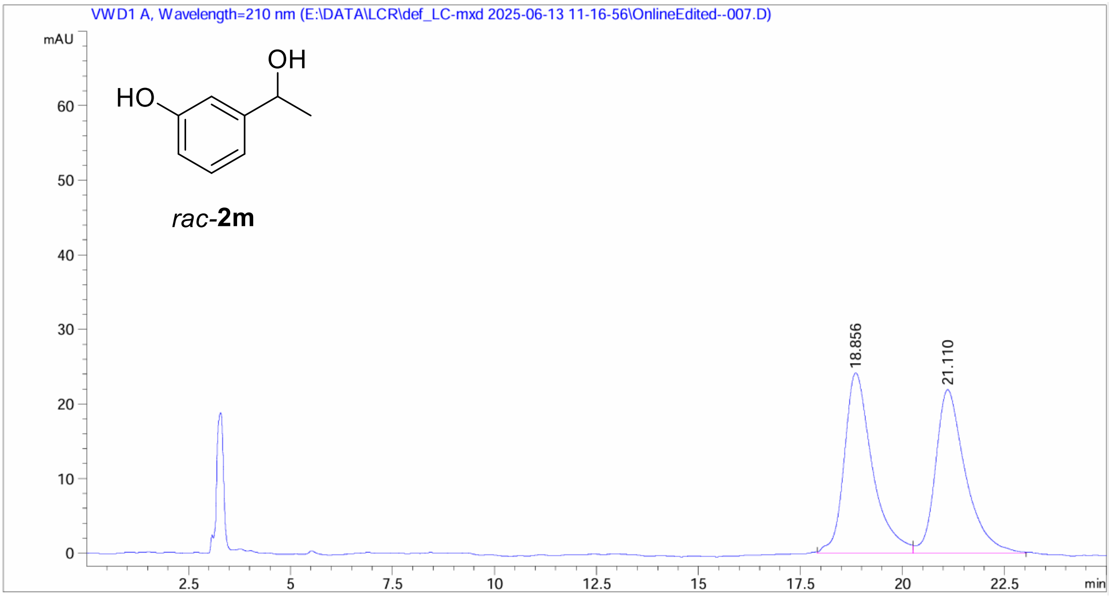

峰 #	保留时间 [min]	类型	峰宽 [min]	峰面积 [mAU*s]	峰高 [mAU]	峰面积 %
1	18.856	MF R	0.7983	1158.41858	24.18476	50.7796
2	21.110	FM R	0.8537	1122.84973	21.92080	49.2204

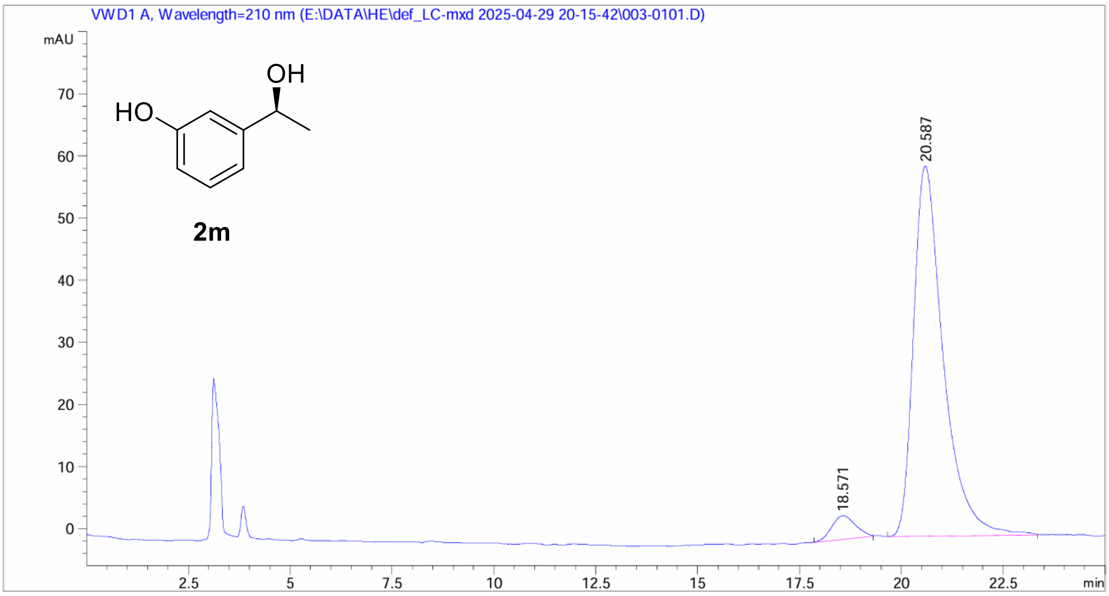

峰 #	保留时间 [min]	类型	峰宽 [min]	峰面积 [mAU*s]	峰高 [mAU]	峰面积 %
1	18.571	MM R	0.6799	155.31755	3.80710	4.8649
2	20.587	MM R	0.8498	3037.30151	59.56628	95.1351

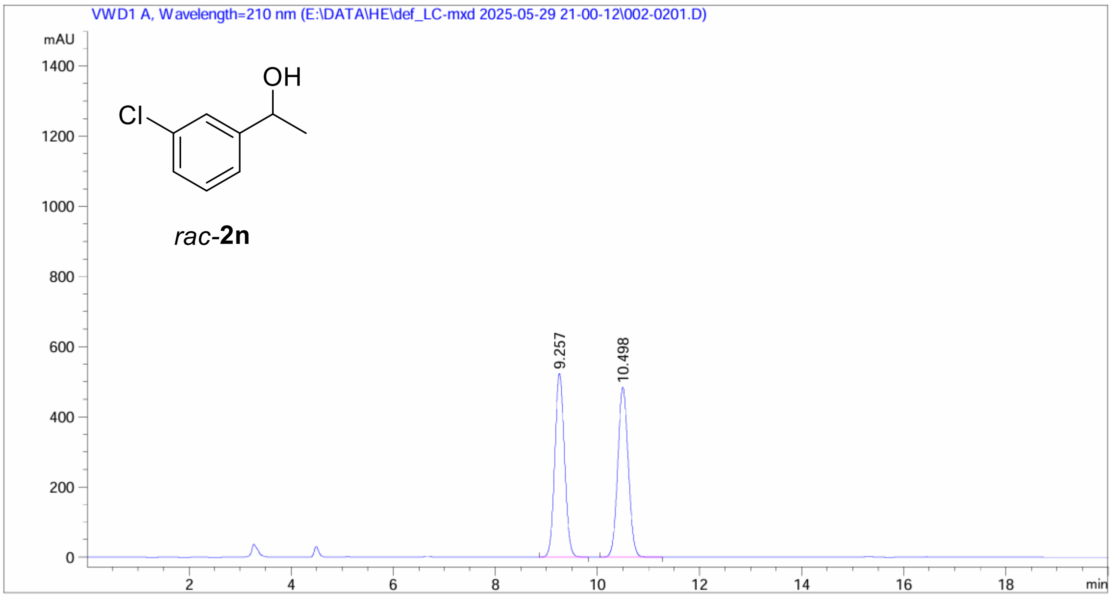

峰 #	保留时间 [min]	类型	峰宽 [min]	峰面积 [mAU*s]	峰高 [mAU]	峰面积 %
1	9.257	BB	0.2051	6923.88086	524.07208	49.9009
2	10.498	BB	0.2235	6951.39551	484.07822	50.0991

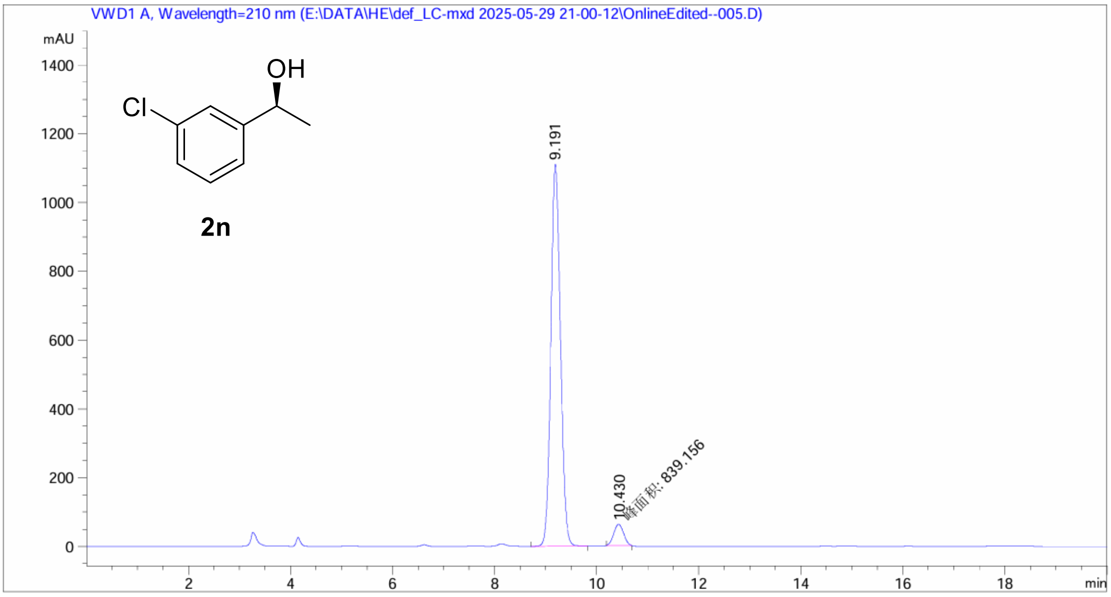

峰 #	保留时间 [min]	类型	峰宽 [min]	峰面积 [mAU*s]	峰高 [mAU]	峰面积 %
1	9.191	BB	0.2004	1.44142e4	1110.96643	94.4985
2	10.430	MM	0.2244	839.15601	62.33885	5.5015

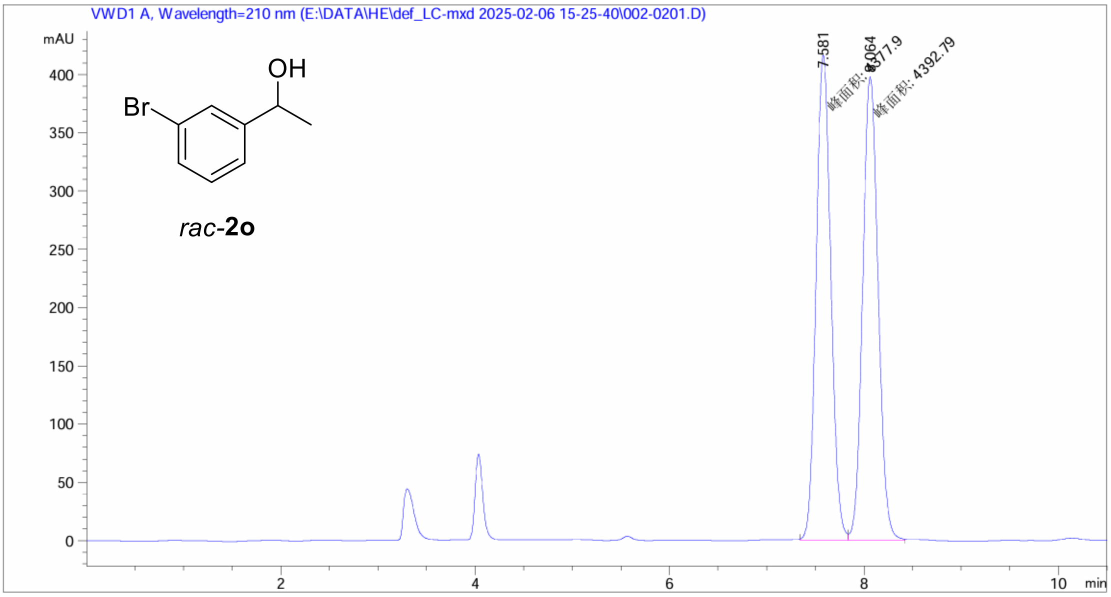

峰 #	保留时间 [min]	类型	峰宽 [min]	峰面积 [mAU*s]	峰高 [mAU]	峰面积 %
1	7.581	MF	0.1753	4377.89600	416.14798	49.9151
2	8.064	FM	0.1842	4392.78662	397.54236	50.0849

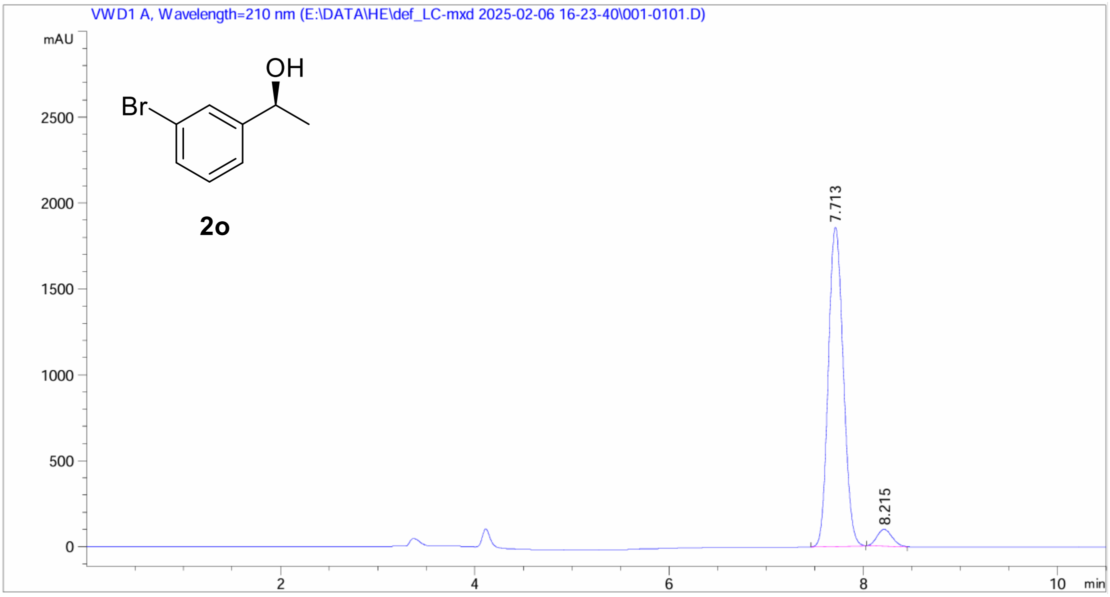

峰 #	保留时间 [min]	类型	峰宽 [min]	峰面积 [mAU*s]	峰高 [mAU]	峰面积 %
1	7.713	MM R	0.1782	1.98804e4	1858.96899	95.0147
2	8.215	MM R	0.1790	1043.10706	97.12874	4.9853

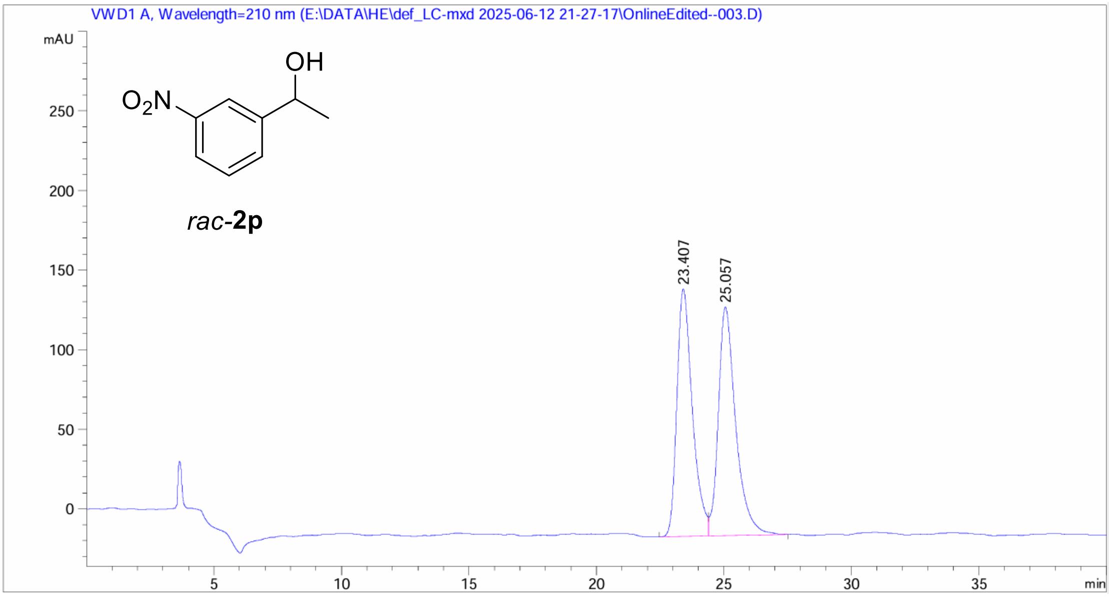

峰 #	保留时间 [min]	类型	峰宽 [min]	峰面积 [mAU*s]	峰高 [mAU]	峰面积 %
1	23.407	BV	0.6376	6578.54395	155.49054	49.0978
2	25.057	VB	0.7081	6820.30518	143.59830	50.9022

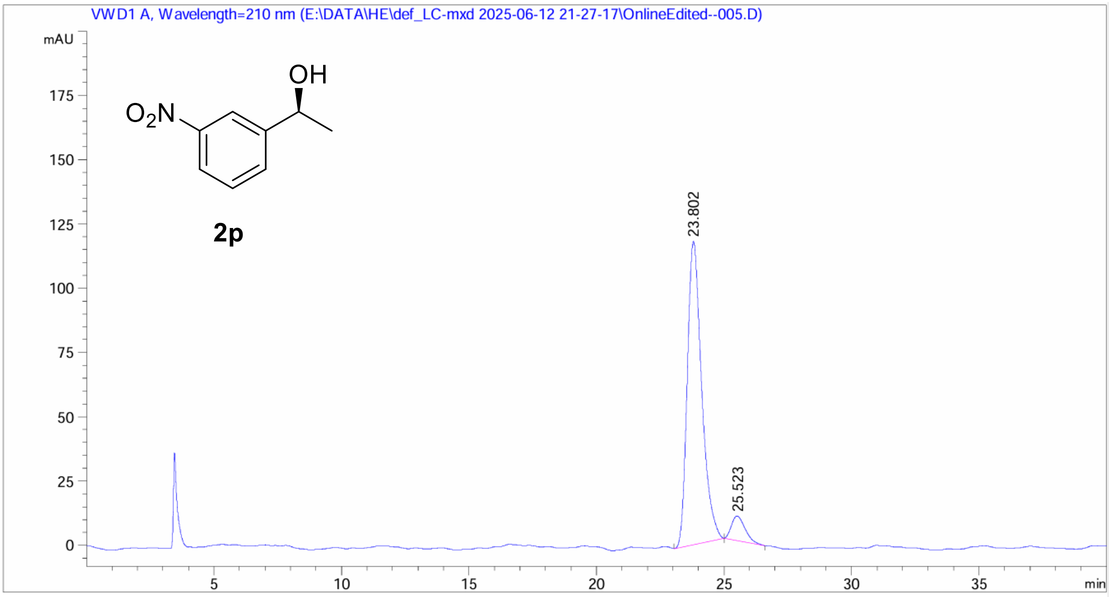

峰 #	保留时间 [min]	类型	峰宽 [min]	峰面积 [mAU*s]	峰高 [mAU]	峰面积 %
1	23.802	MM R	0.6549	4637.86475	118.02720	92.7052
2	25.523	MM R	0.6278	364.94437	9.68790	7.2948

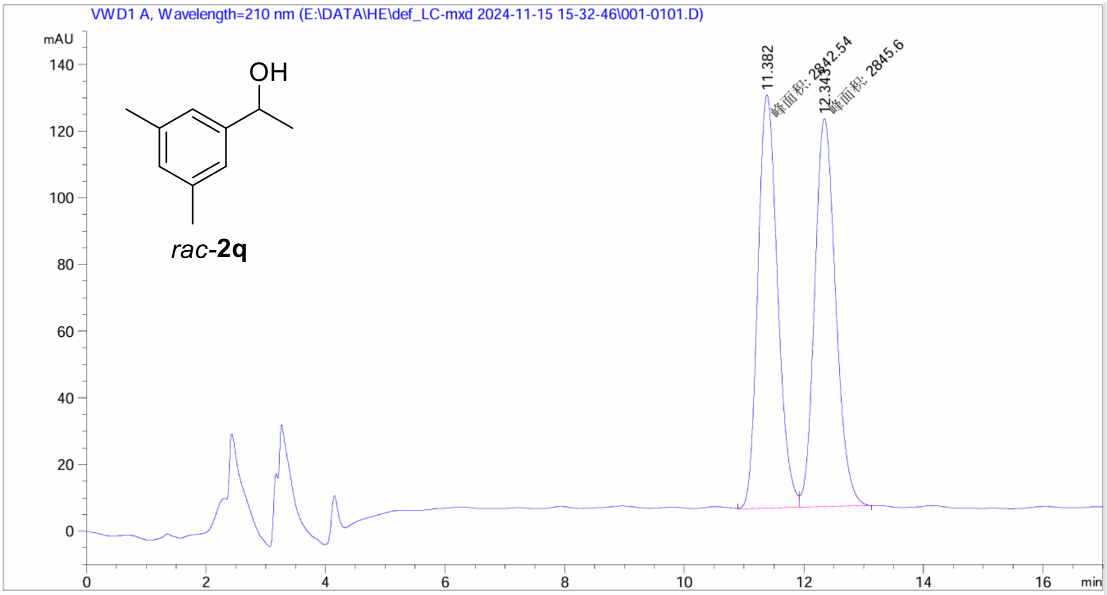

峰 #	保留时间 [min]	类型	峰宽 [min]	峰面积 [mAU*s]	峰高 [mAU]	峰面积 %
1	11.382	MF	0.3819	2842.54028	124.06493	49.9731
2	12.343	FM	0.4071	2845.59546	116.48859	50.0269

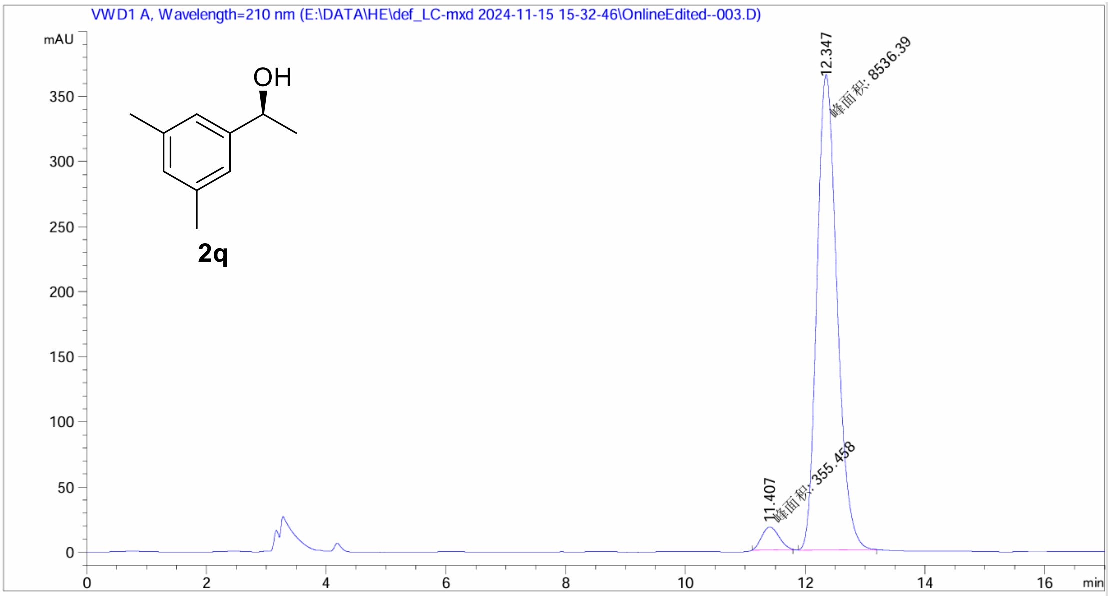

峰 #	保留时间 [min]	类型	峰宽 [min]	峰面积 [mAU*s]	峰高 [mAU]	峰面积 %
1	11.407	MM	0.3344	355.45840	17.71581	3.9976
2	12.347	MM	0.3896	8536.39063	365.13644	96.0024

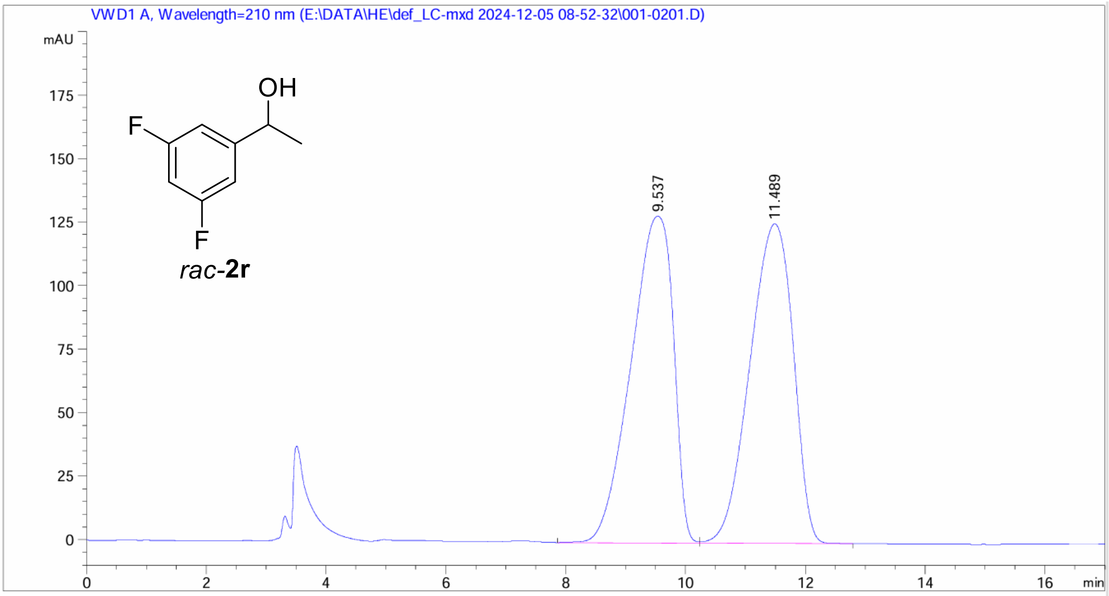

峰 #	保留时间 [min]	类型	峰宽 [min]	峰面积 [mAU*s]	峰高 [mAU]	峰面积 %
1	9.537	BV	0.7595	6319.50586	128.55331	50.0085
2	11.489	VB	0.8132	6317.34863	125.72824	49.9915

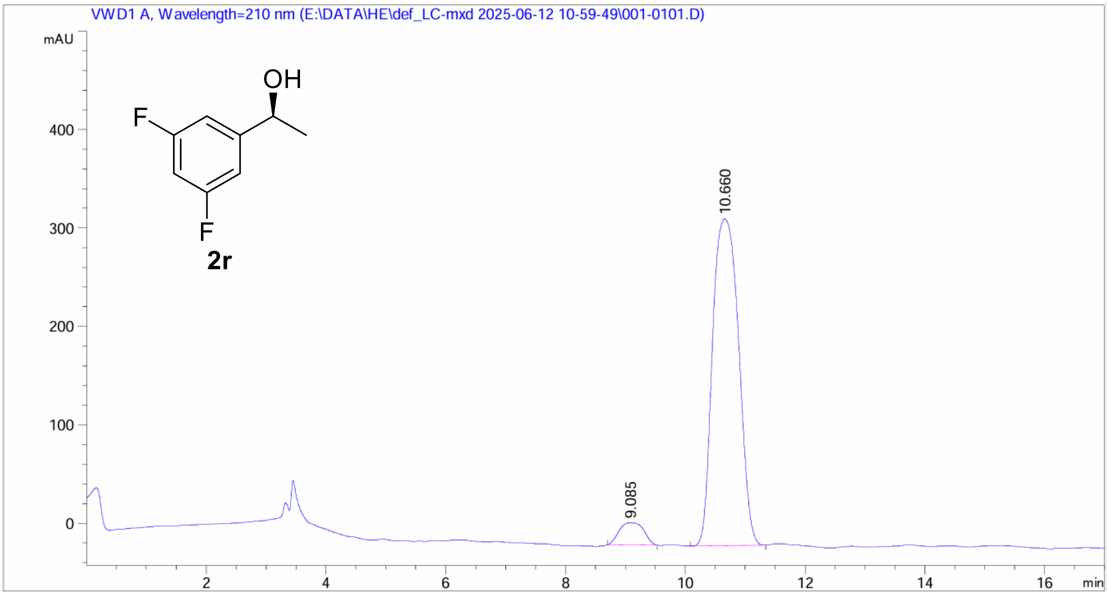

峰 #	保留时间 [min]	类型	峰宽 [min]	峰面积 [mAU*s]	峰高 [mAU]	峰面积 %
1	9.085	MM R	0.4758	644.17255	22.56380	6.0337
2	10.660	MM R	0.5034	1.00321e4	332.12015	93.9663

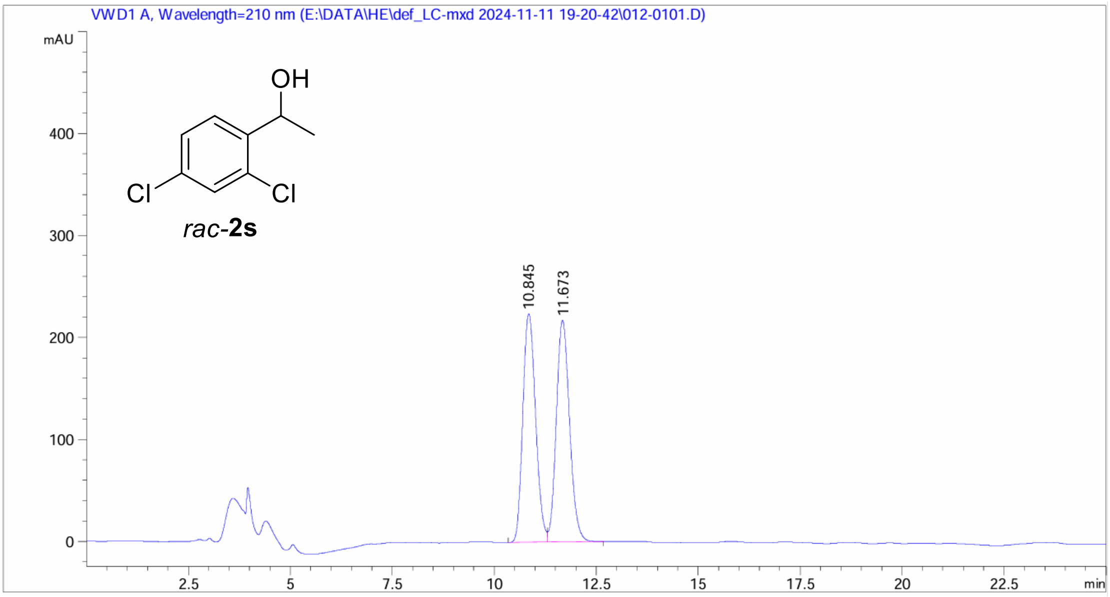

峰 #	保留时间 [min]	类型	峰宽 [min]	峰面积 [mAU*s]	峰高 [mAU]	峰面积 %
1	10.845	BV	0.3441	4877.83691	223.92191	49.7844
2	11.673	VB	0.3532	4920.07861	217.27675	50.2156

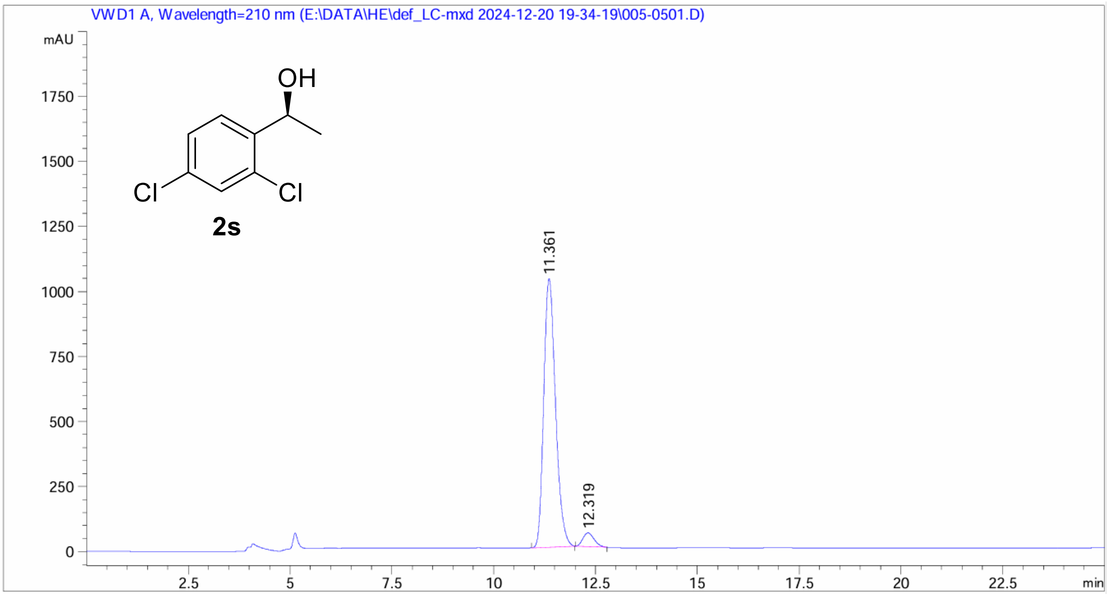

峰 #	保留时间 [min]	类型	峰宽 [min]	峰面积 [mAU*s]	峰高 [mAU]	峰面积 %
1	11.361	MM R	0.3342	2.07048e4	1032.41943	95.3492
2	12.319	MM R	0.3205	1009.90265	52.51021	4.6508

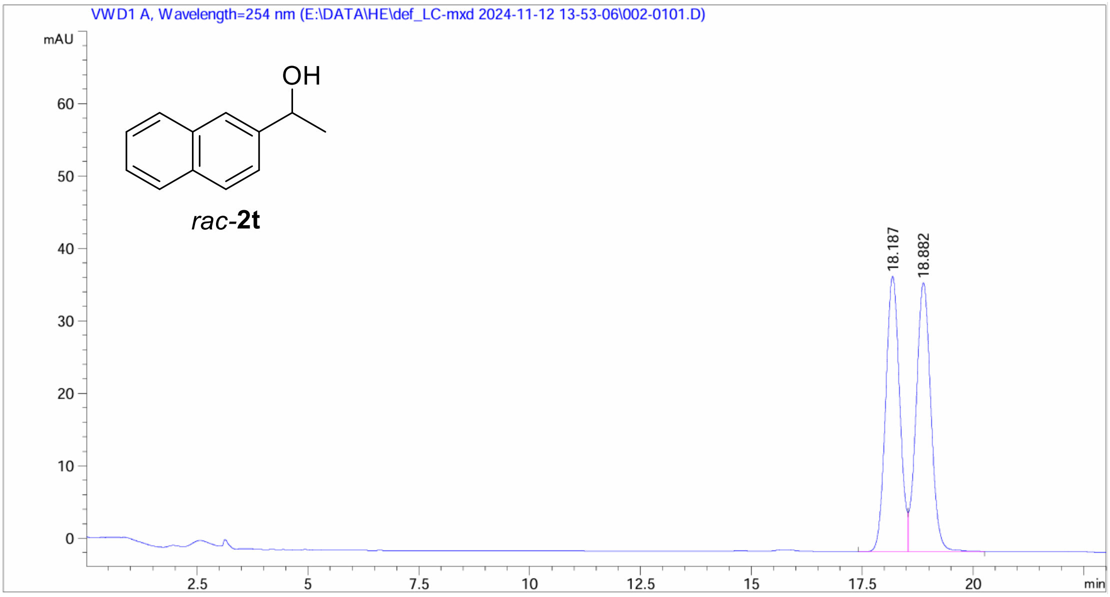

峰 #	保留时间 [min]	类型	峰宽 [min]	峰面积 [mAU*s]	峰高 [mAU]	峰面积 %
1	18.187	MF	0.3617	815.51984	37.58055	49.5745
2	18.882	FM	0.3765	829.51947	36.71760	50.4255

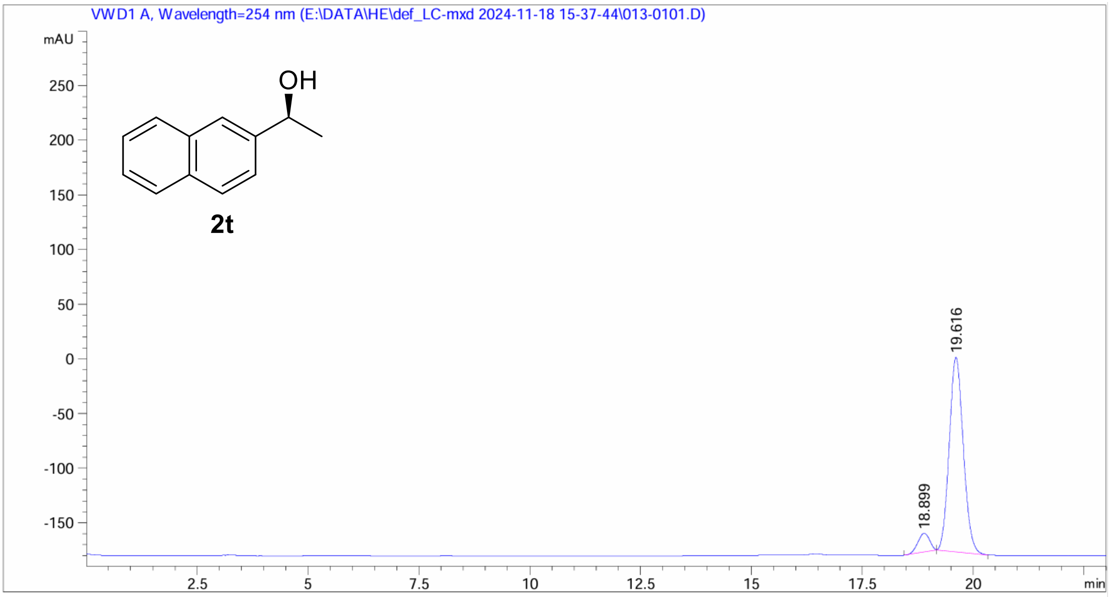

峰 #	保留时间 [min]	类型	峰宽 [min]	峰面积 [mAU*s]	峰高 [mAU]	峰面积 %
1	18.899	MM R	0.3064	314.22537	17.09499	7.3940
2	19.616	MM R	0.3682	3935.52466	178.13528	92.6060

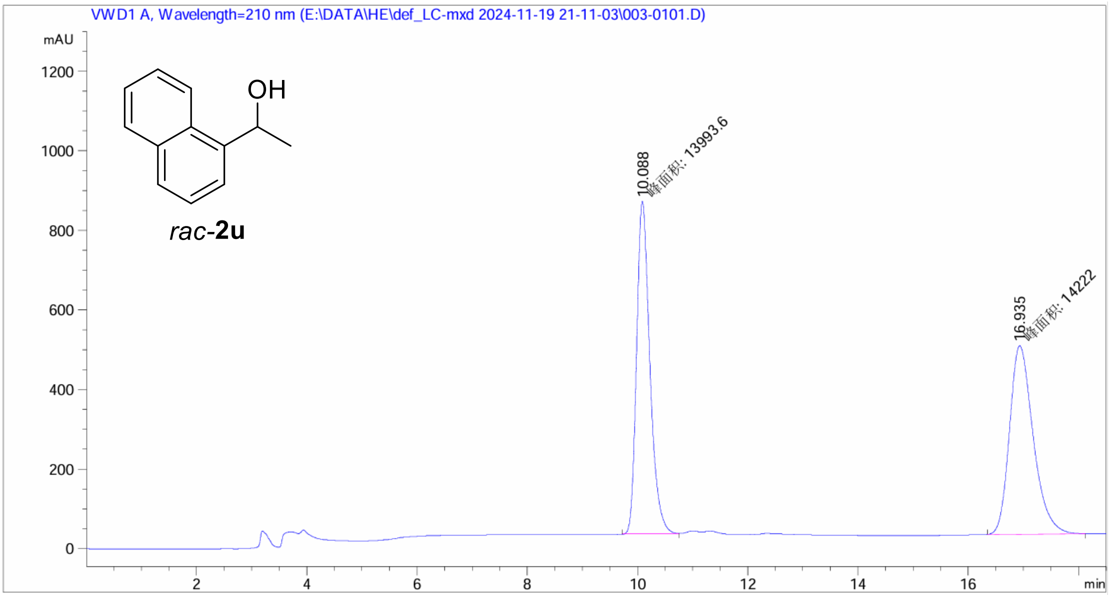

峰 #	保留时间 [min]	类型	峰宽 [min]	峰面积 [mAU*s]	峰高 [mAU]	峰面积 %
1	10.088	MM	0.2791	1.39936e4	835.70825	49.5952
2	16.935	MM	0.4998	1.42220e4	474.25992	50.4048

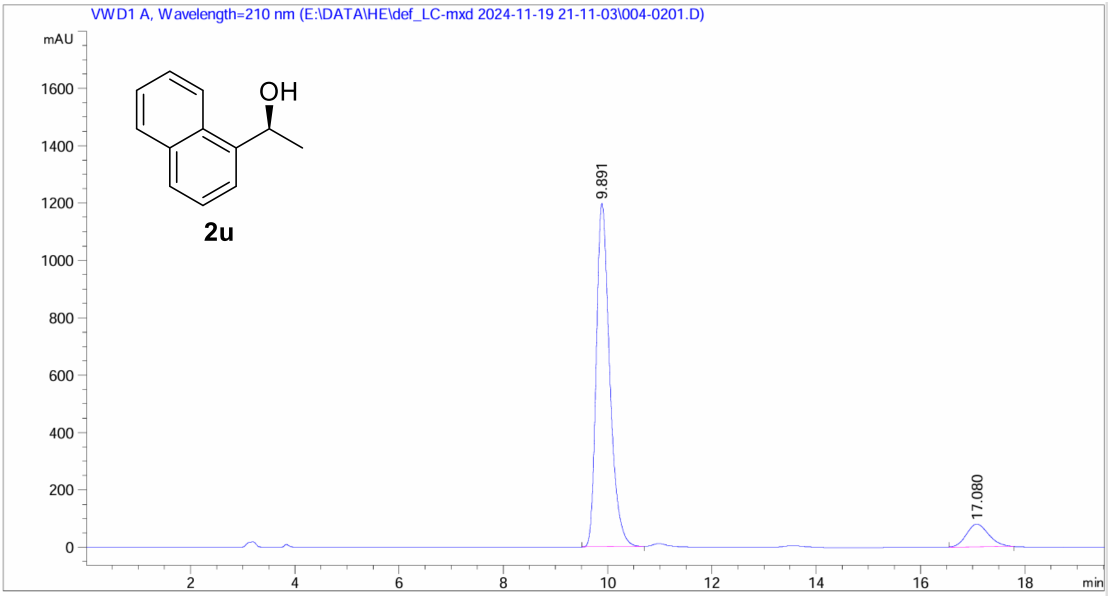

峰 #	保留时间 [min]	类型	峰宽 [min]	峰面积 [mAU*s]	峰高 [mAU]	峰面积 %
1	9.891	MM R	0.2974	2.13702e4	1197.65527	90.0933
2	17.080	MM R	0.4945	2349.87231	79.20544	9.9067

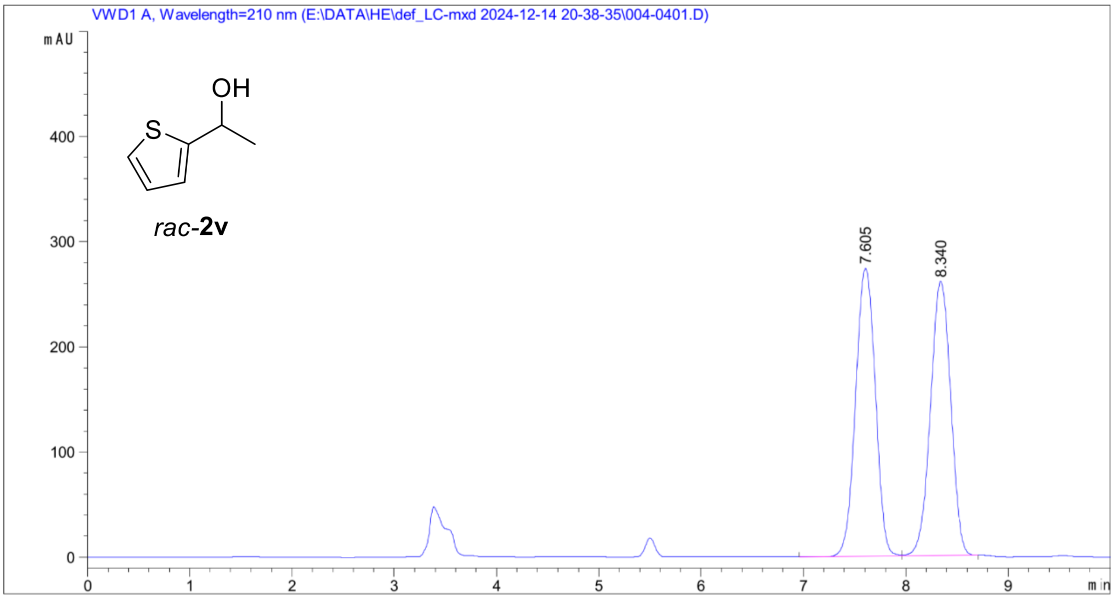

峰 #	保留时间 [min]	类型	峰宽 [min]	峰面积 [mAU*s]	峰高 [mAU]	峰面积 %
1	7.605	BV	0.2110	3636.94775	273.68286	50.3137
2	8.340	VB	0.2179	3591.59180	260.44226	49.6863

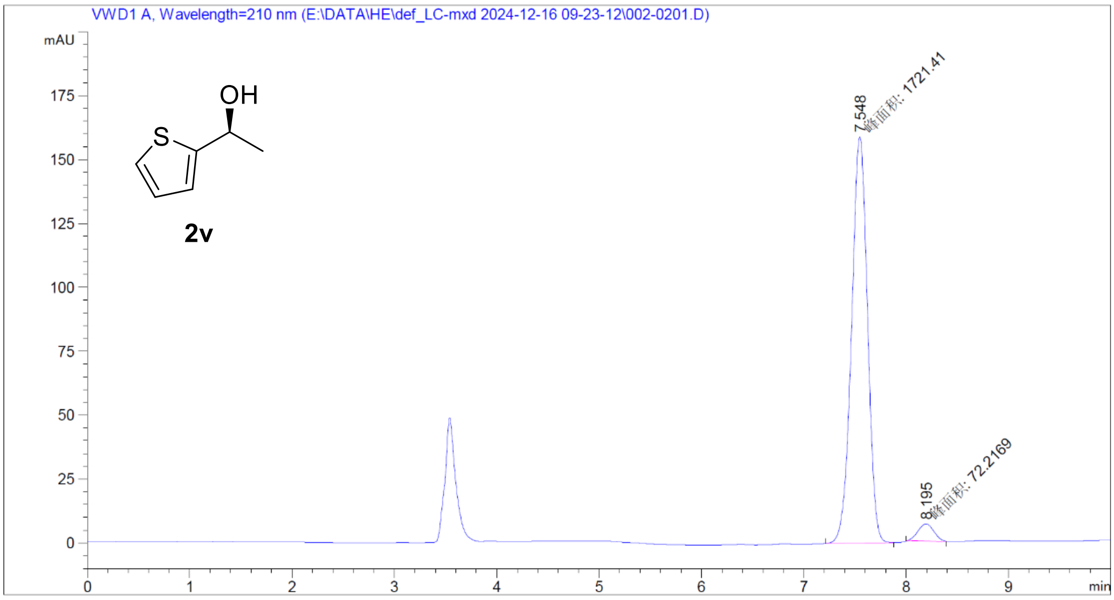

峰 #	保留时间 [min]	类型	峰宽 [min]	峰面积 [mAU*s]	峰高 [mAU]	峰面积 %
1	7.548	MM	0.1805	1721.40613	158.90474	95.9737
2	8.195	MM	0.1797	72.21690	6.69912	4.0263

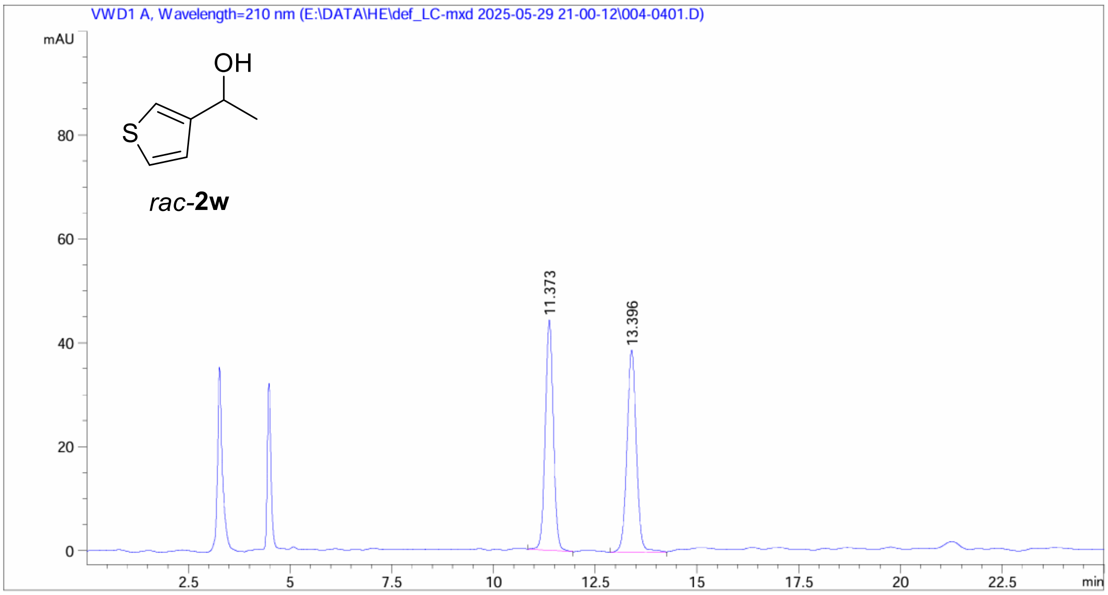

峰 #	保留时间 [min]	类型	峰宽 [min]	峰面积 [mAU*s]	峰高 [mAU]	峰面积 %
1	11.373	BB	0.2127	610.82050	44.35609	49.4278
2	13.396	BB	0.2476	624.96289	38.87222	50.5722

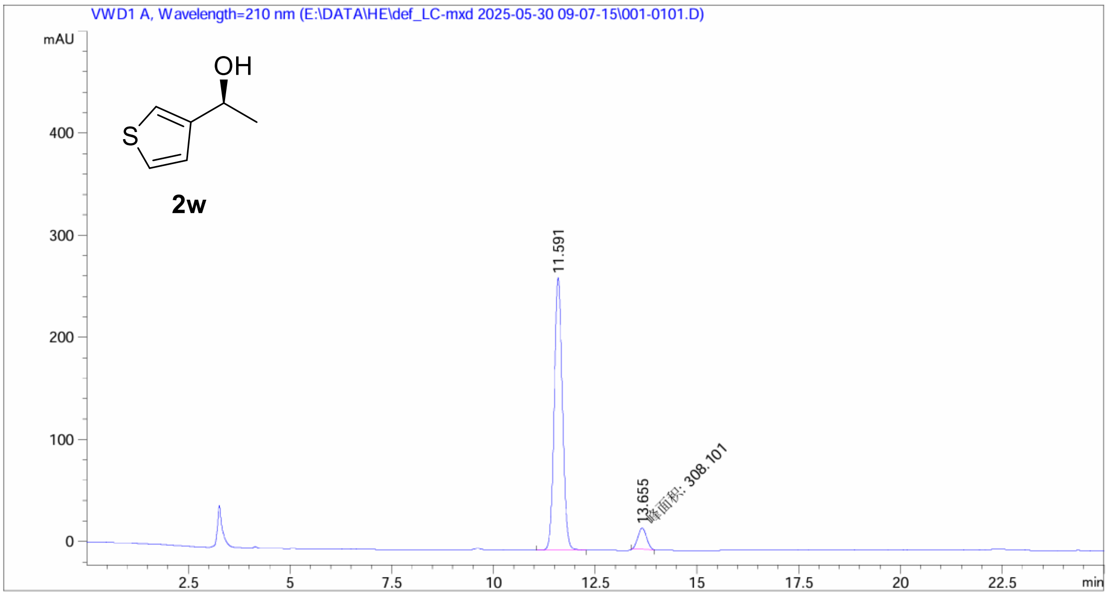

峰 #	保留时间 [min]	类型	峰宽 [min]	峰面积 [mAU*s]	峰高 [mAU]	峰面积 %
1	11.591	BB	0.2183	3758.05908	266.90884	92.4228
2	13.655	MM	0.2465	308.10086	20.83524	7.5772

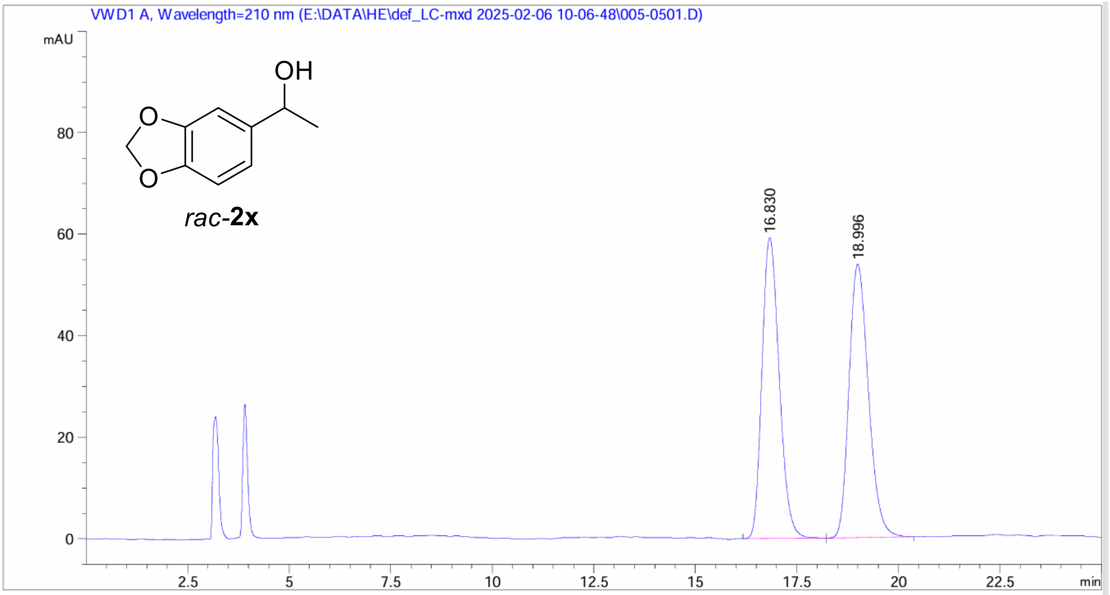

峰 #	保留时间 [min]	类型	峰宽 [min]	峰面积 [mAU*s]	峰高 [mAU]	峰面积 %
1	16.830	BB	0.4637	1752.20850	59.29804	49.4547
2	18.996	BB	0.5164	1790.84949	53.93598	50.5453

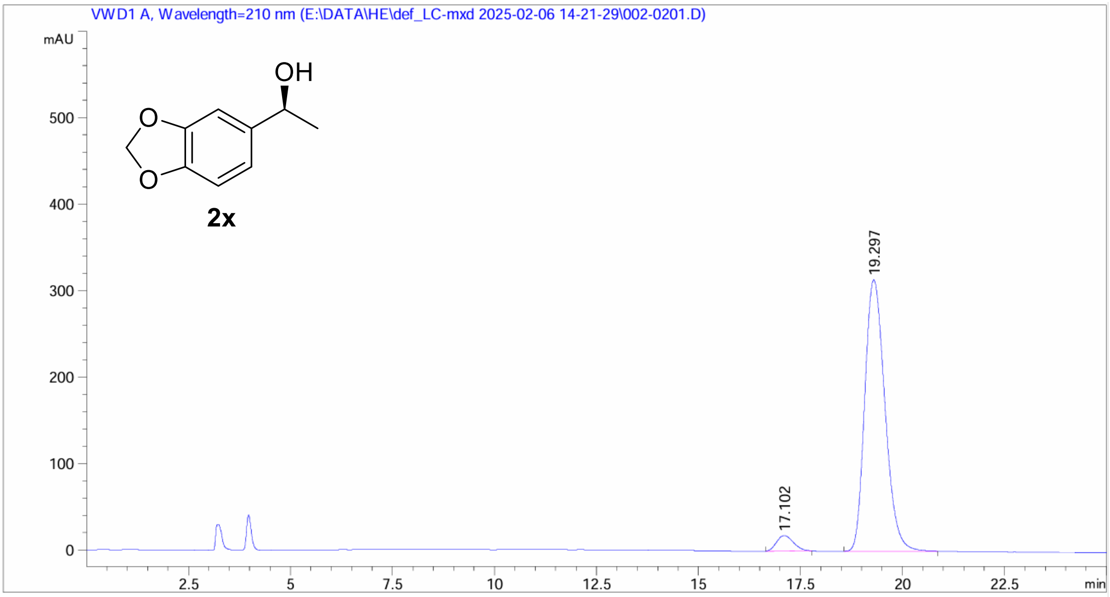

峰 #	保留时间 [min]	类型	峰宽 [min]	峰面积 [mAU*s]	峰高 [mAU]	峰面积 %
1	17.102	MM R	0.4827	521.71057	18.01327	4.6027
2	19.297	MM R	0.5737	1.08133e4	314.12936	95.3973

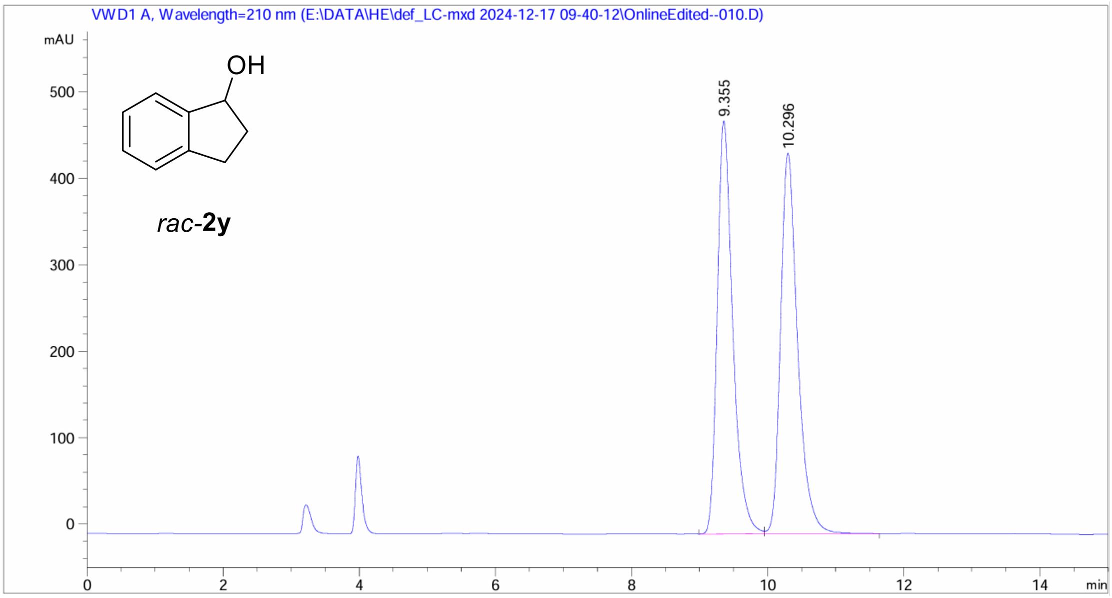

峰 #	保留时间 [min]	类型	峰宽 [min]	峰面积 [mAU*s]	峰高 [mAU]	峰面积 %
1	9.355	BV	0.2389	7574.43799	478.05179	49.6630
2	10.296	VB	0.2624	7677.22803	440.31909	50.3370

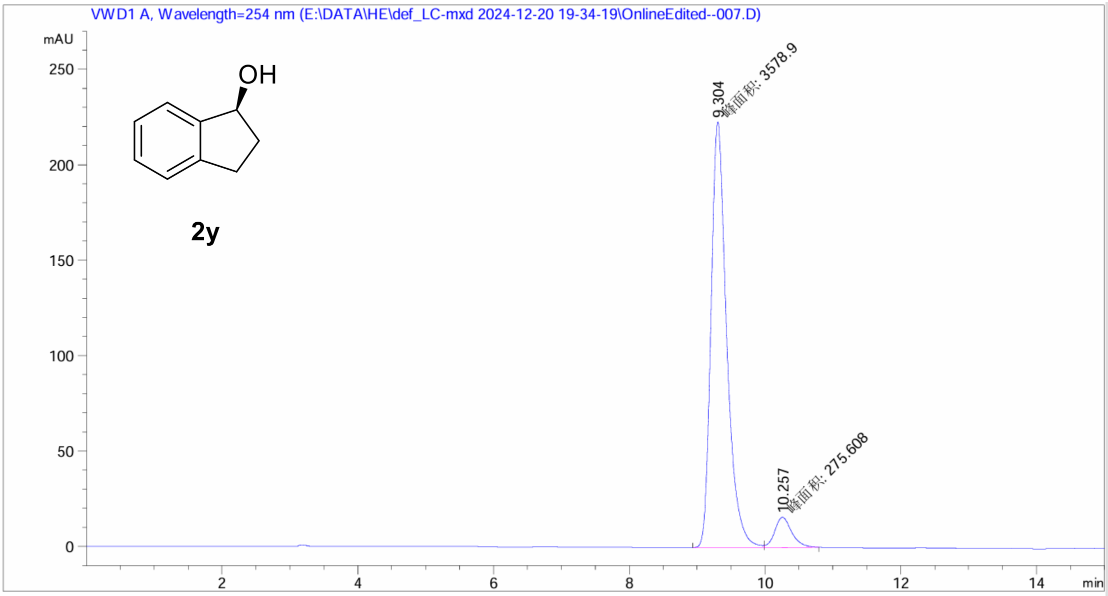

峰 #	保留时间 [min]	类型	峰宽 [min]	峰面积 [mAU*s]	峰高 [mAU]	峰面积 %
1	9.304	MF	0.2674	3578.90112	223.07990	92.8497
2	10.257	FM	0.2901	275.60760	15.83365	7.1503

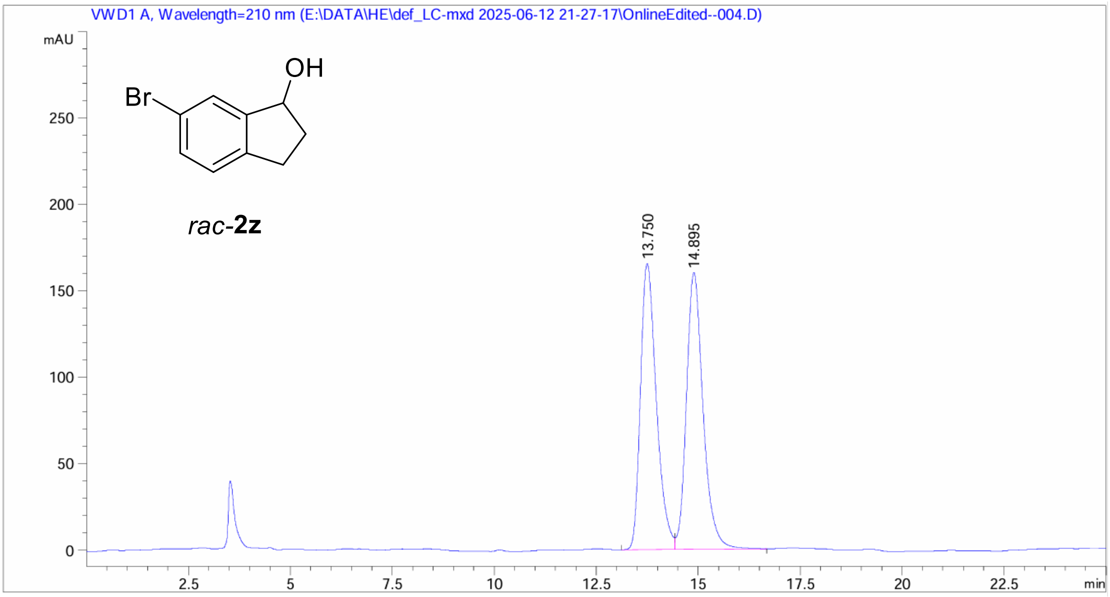

峰 #	保留时间 [min]	类型	峰宽 [min]	峰面积 [mAU*s]	峰高 [mAU]	峰面积 %
1	13.750	BV	0.4145	4473.88135	165.45609	49.5468
2	14.895	VB	0.4322	4555.71973	159.99370	50.4532

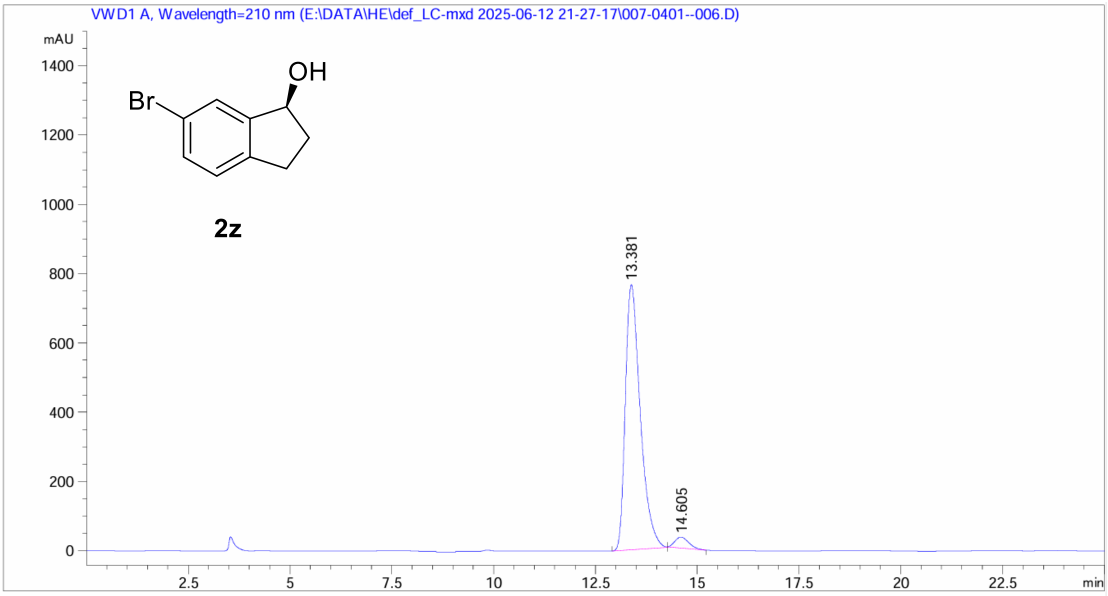

峰 #	保留时间 [min]	类型	峰宽 [min]	峰面积 [mAU*s]	峰高 [mAU]	峰面积 %
1	13.381	MM R	0.4247	1.94904e4	764.88232	96.1927
2	14.605	MM R	0.4007	771.42444	32.08864	3.8073

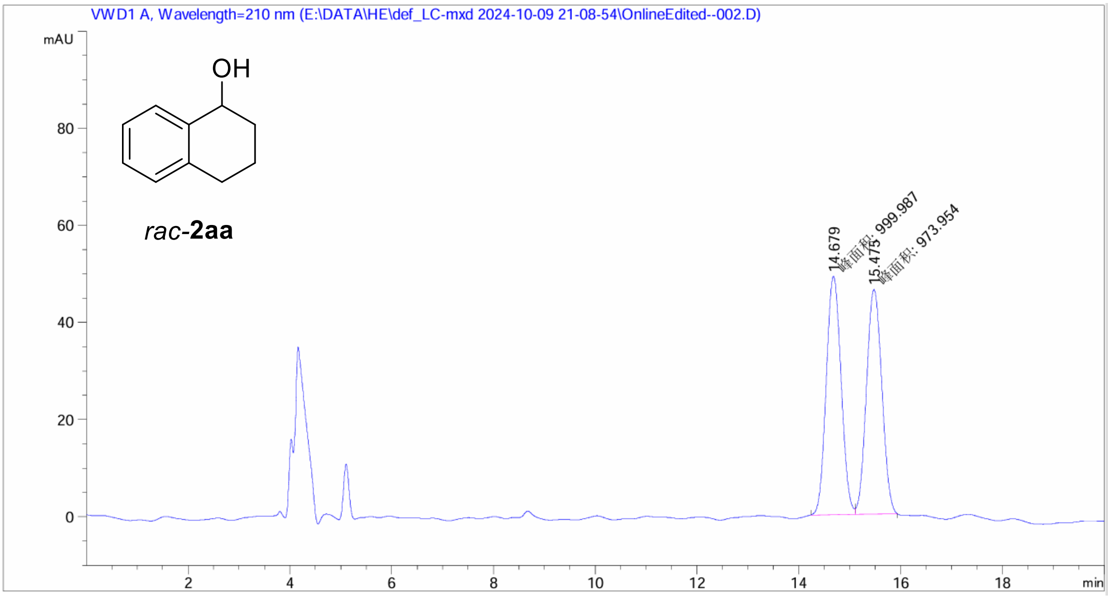

峰 #	保留时间 [min]	类型	峰宽 [min]	峰面积 [mAU*s]	峰高 [mAU]	峰面积 %
1	14.679	MF	0.3391	999.98718	49.14677	50.6594
2	15.475	FM	0.3506	973.95392	46.30362	49.3406

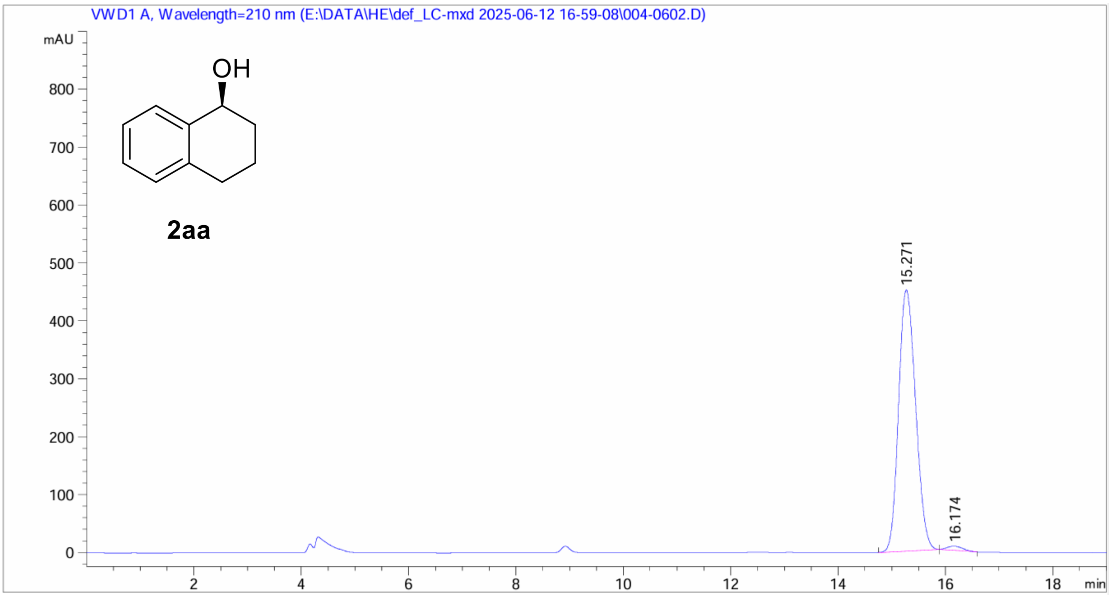

峰 #	保留时间 [min]	类型	峰宽 [min]	峰面积 [mAU*s]	峰高 [mAU]	峰面积 %
1	15.271	MM R	0.3672	9936.21680	450.99341	98.5557
2	16.174	MM R	0.3312	145.61525	7.32865	1.4443

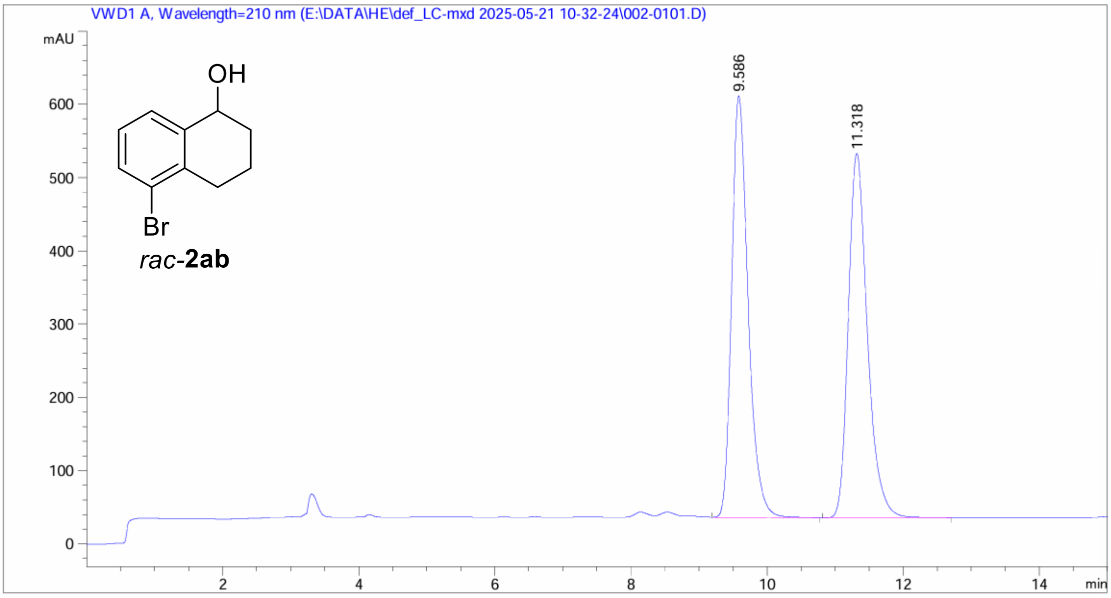

峰 #	保留时间 [min]	类型	峰宽 [min]	峰面积 [mAU*s]	峰高 [mAU]	峰面积 %
1	9.586	BB	0.2560	9660.80078	575.06830	49.9448
2	11.318	BB	0.2972	9682.15137	497.23087	50.0552

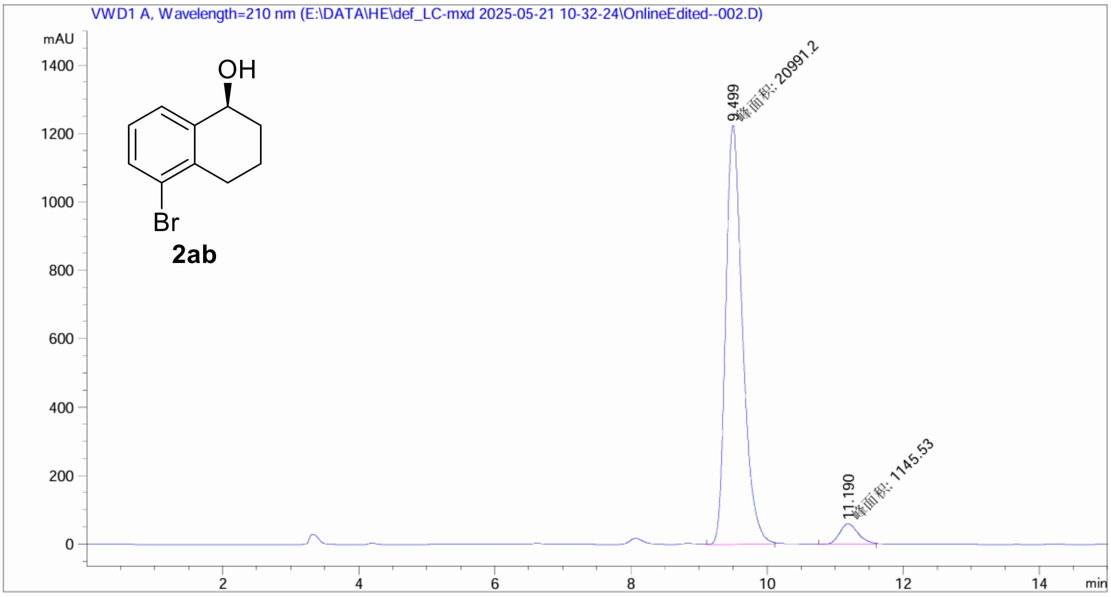

峰 #	保留时间 [min]	类型	峰宽 [min]	峰面积 [mAU*s]	峰高 [mAU]	峰面积 %
1	9.499	MF	0.2857	2.09912e4	1224.73633	94.8252
2	11.190	MF	0.3180	1145.53101	60.03634	5.1748

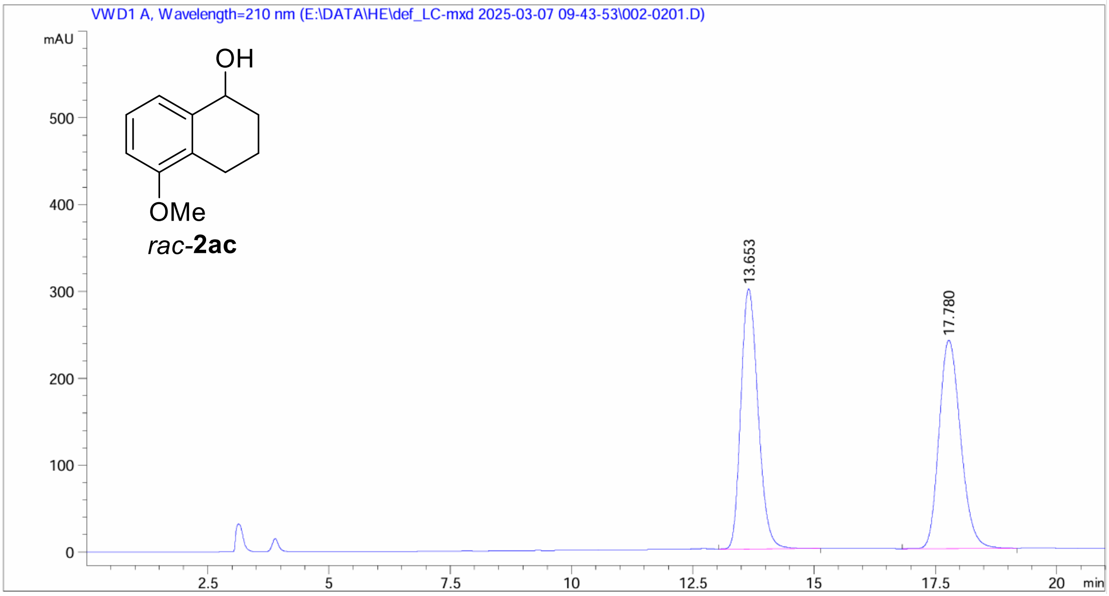

峰 #	保留时间 [min]	类型	峰宽 [min]	峰面积 [mAU*s]	峰高 [mAU]	峰面积 %
1	13.653	BB	0.3869	7392.77393	299.54089	49.8990
2	17.780	BB	0.4829	7422.69775	239.96245	50.1010

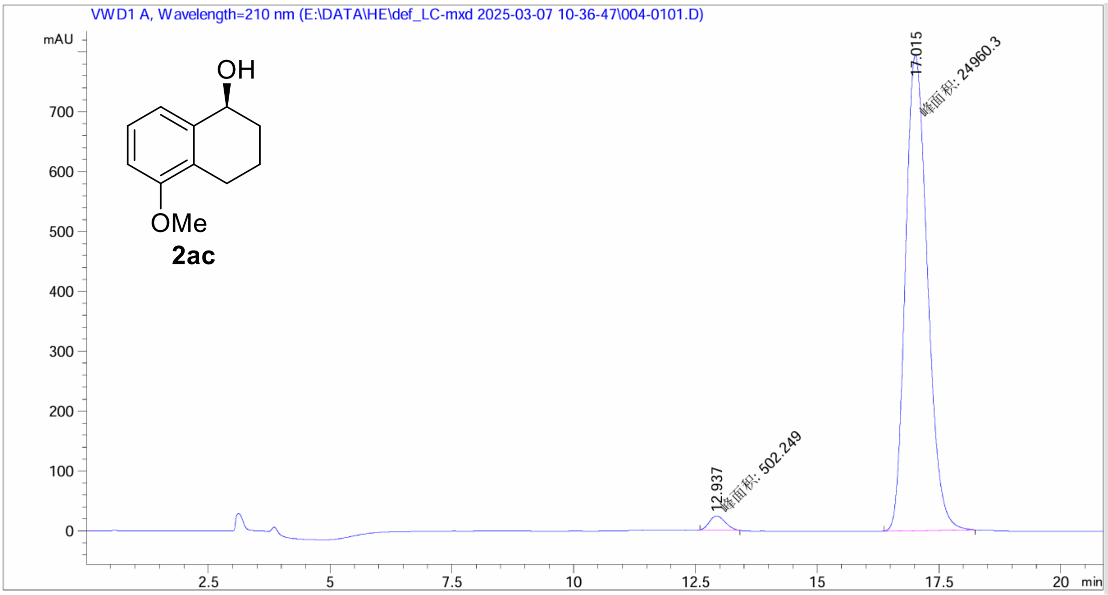

峰 #	保留时间 [min]	类型	峰宽 [min]	峰面积 [mAU*s]	峰高 [mAU]	峰面积 %
1	12.937	MM	0.3637	502.24948	23.01377	1.9725
2	17.015	MM	0.5239	2.49603e4	794.06281	98.0275

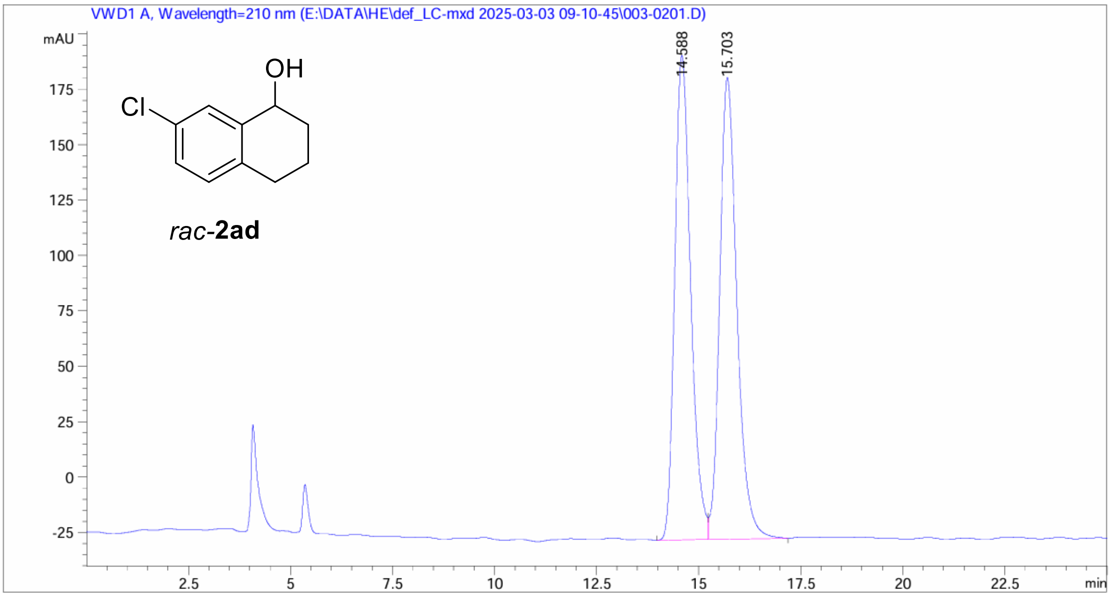

峰 #	保留时间 [min]	类型	峰宽 [min]	峰面积 [mAU*s]	峰高 [mAU]	峰面积 %
1	14.588	BV	0.4175	5932.45410	218.64725	49.2965
2	15.703	VB	0.4465	6101.77100	208.44101	50.7035

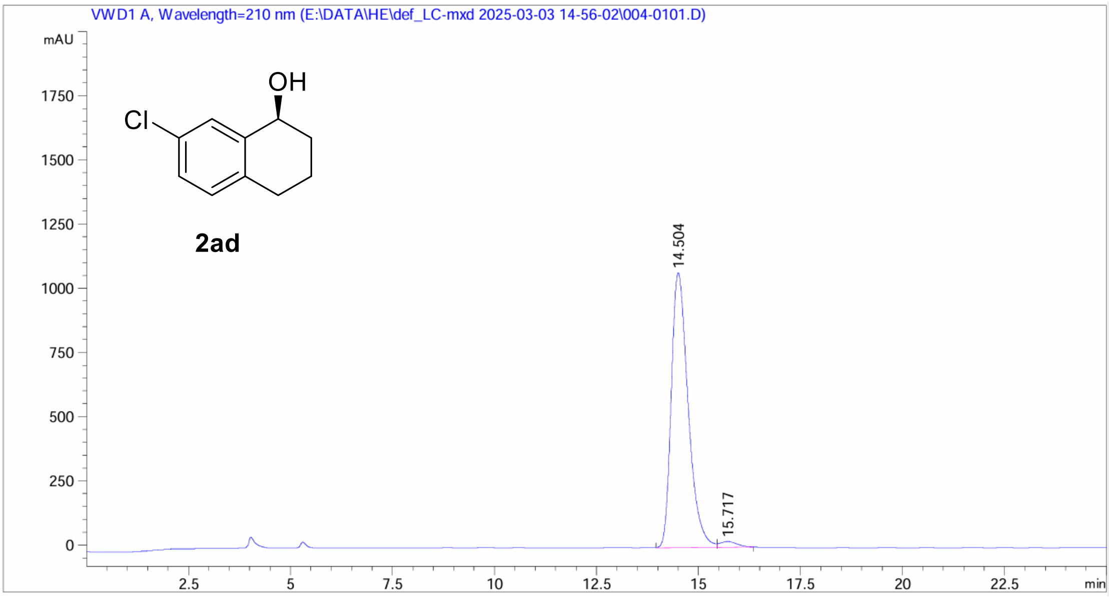

峰 #	保留时间 [min]	类型	峰宽 [min]	峰面积 [mAU*s]	峰高 [mAU]	峰面积 %
1	14.504	MF R	0.4789	3.07321e4	1069.51892	97.9904
2	15.717	FM R	0.4807	630.26062	21.85124	2.0096

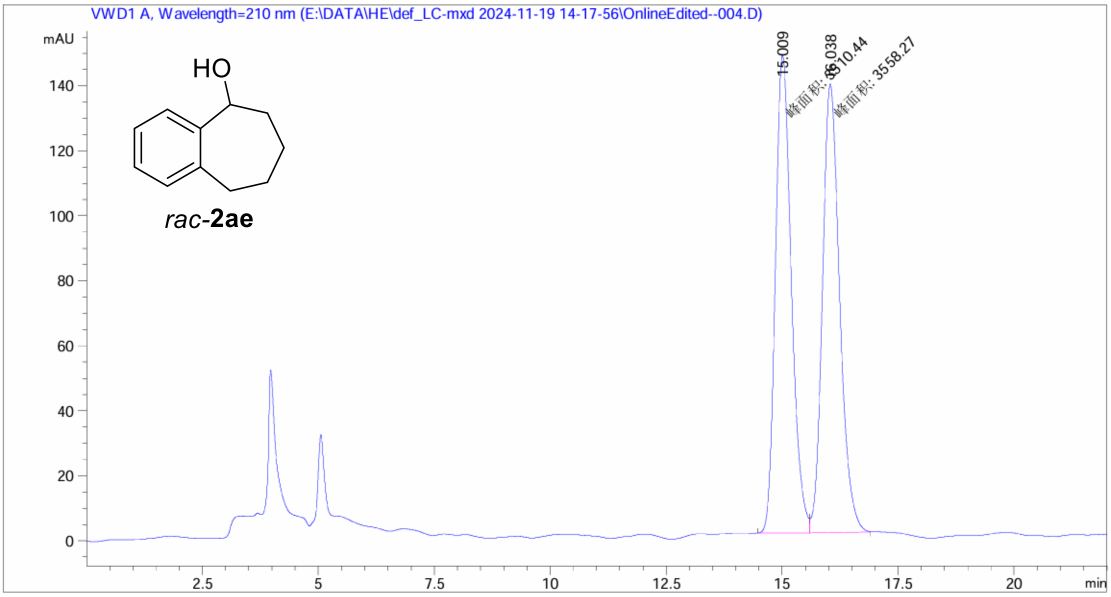

峰 #	保留时间 [min]	类型	峰宽 [min]	峰面积 [mAU*s]	峰高 [mAU]	峰面积 %
1	15.009	MF	0.3982	3510.43921	146.93887	49.6617
2	16.038	FM	0.4296	3558.27271	138.03850	50.3383

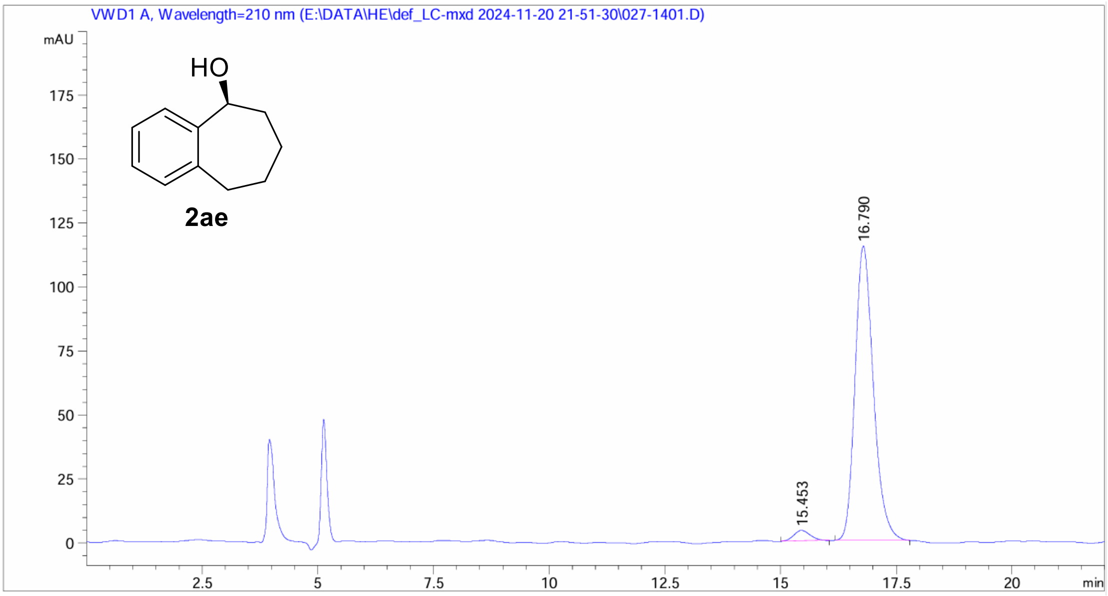

峰 #	保留时间 [min]	类型	峰宽 [min]	峰面积 [mAU*s]	峰高 [mAU]	峰面积 %
1	15.453	MM R	0.4052	98.86916	4.06671	3.0226
2	16.790	MM R	0.4600	3172.17969	114.94176	96.9774

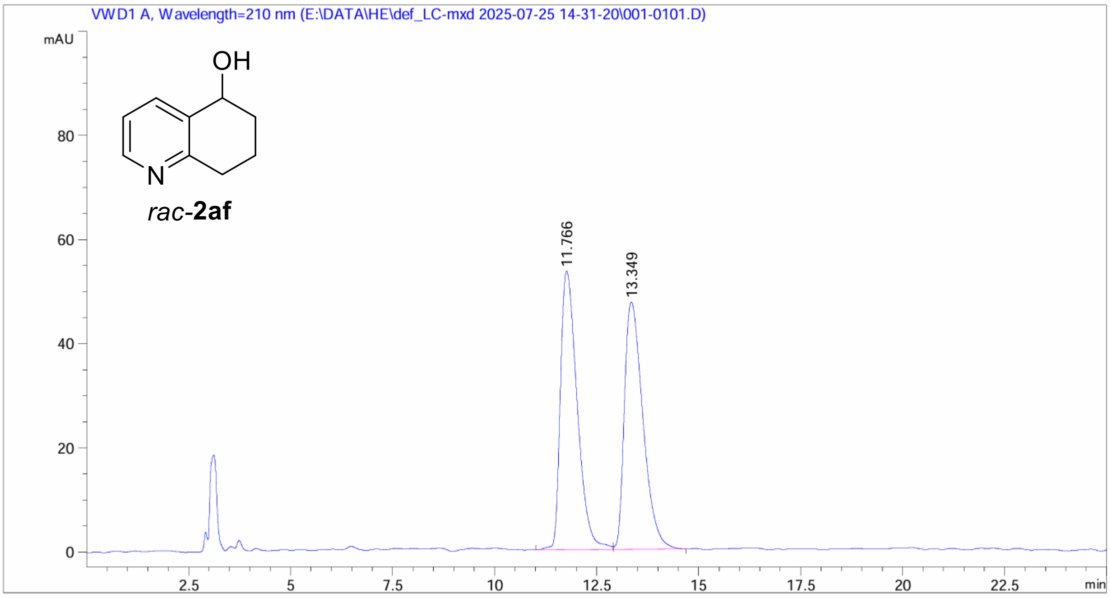

峰 #	保留时间 [min]	类型	峰宽 [min]	峰面积 [mAU*s]	峰高 [mAU]	峰面积 %
1	11.766	BV	0.4440	1532.11890	53.52262	50.4989
2	13.349	VB	0.4937	1501.84668	47.52842	49.5011

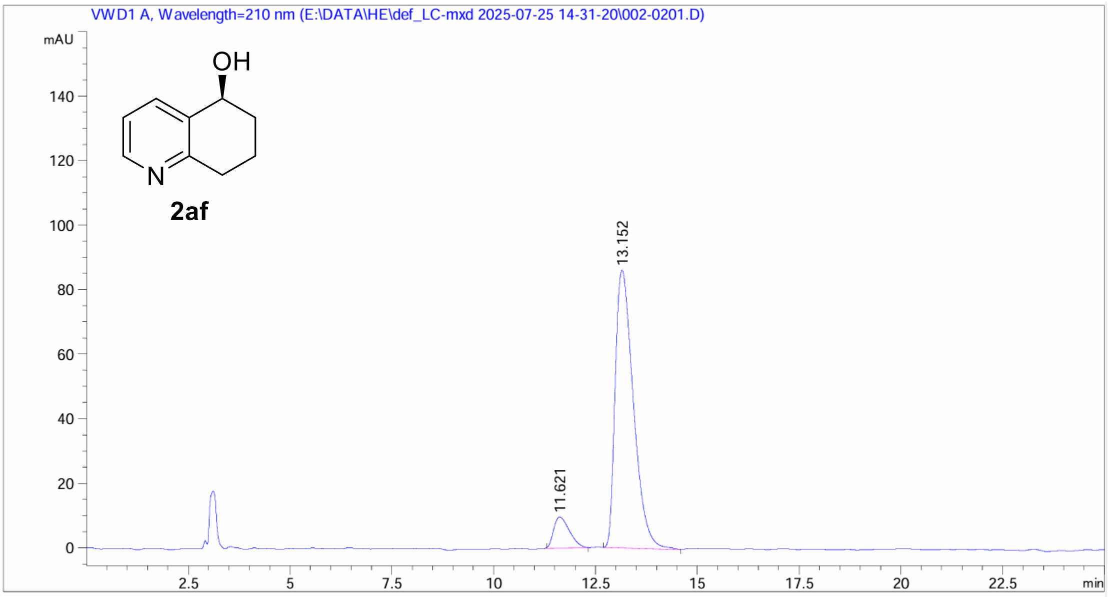

峰 #	保留时间 [min]	类型	峰宽 [min]	峰面积 [mAU*s]	峰高 [mAU]	峰面积 %
1	11.621	MM R	0.4507	260.76865	9.64365	8.8915
2	13.152	MM R	0.5173	2672.02100	86.09533	91.1085

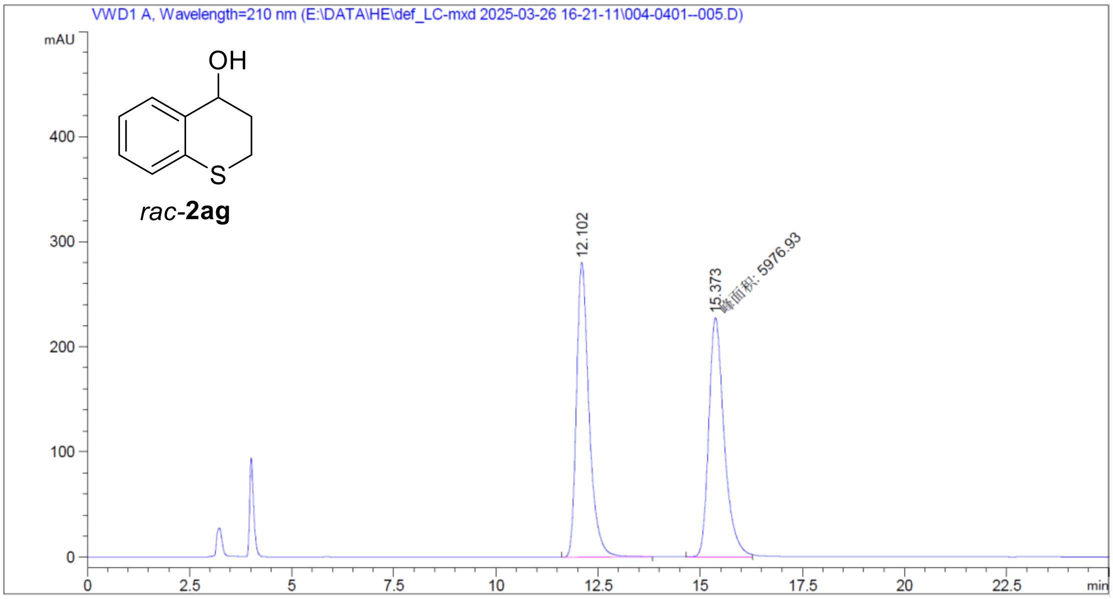

峰 #	保留时间 [min]	类型	峰宽 [min]	峰面积 [mAU*s]	峰高 [mAU]	峰面积 %
1	12.102	BB	0.3226	5998.01123	280.38669	50.0880
2	15.373	MF	0.4380	5976.92627	227.43719	49.9120

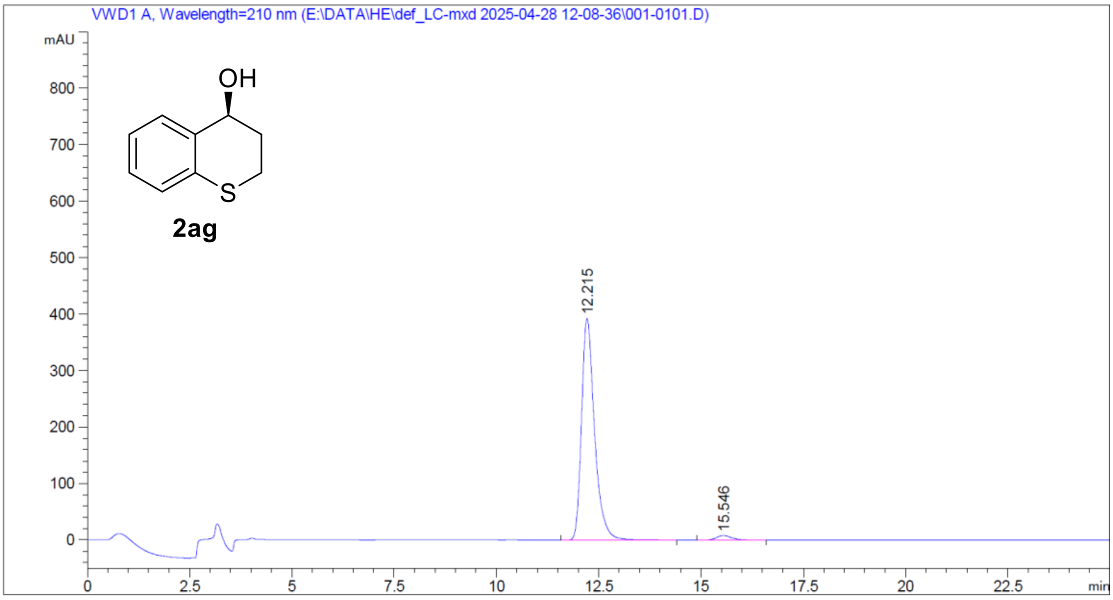

峰 #	保留时间 [min]	类型	峰宽 [min]	峰面积 [mAU*s]	峰高 [mAU]	峰面积 %
1	12.215	BB	0.3341	8675.68457	392.20071	97.4512
2	15.546	BB	0.4126	226.91173	8.25828	2.5488

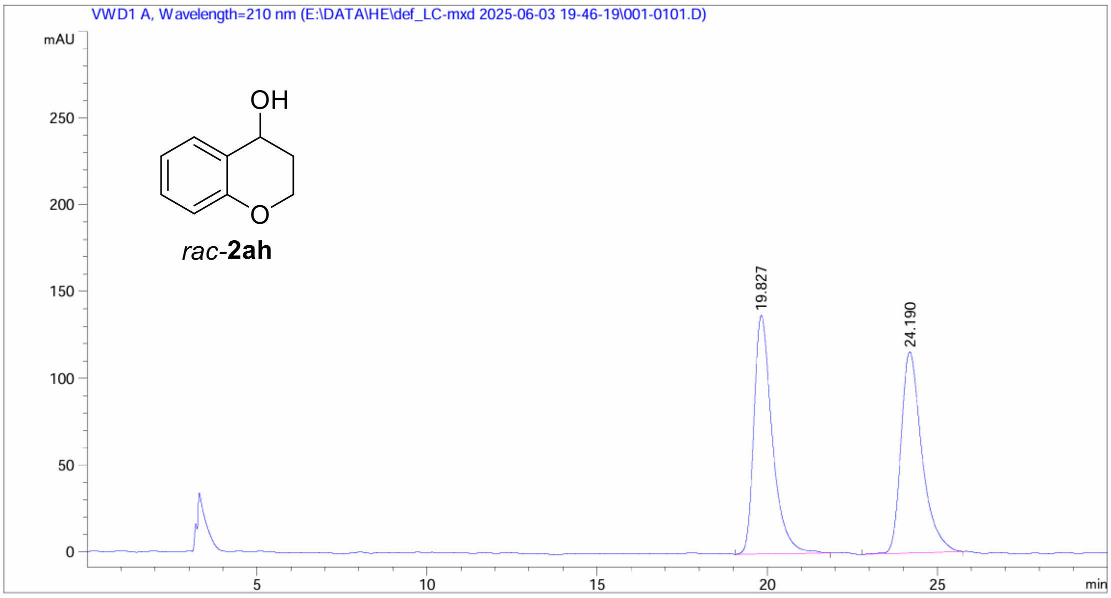

峰 #	保留时间 [min]	类型	峰宽 [min]	峰面积 [mAU*s]	峰高 [mAU]	峰面积 %
1	19.827	BB	0.5657	5145.65283	137.47089	50.9590
2	24.190	BB	0.6447	4951.98047	115.84496	49.0410

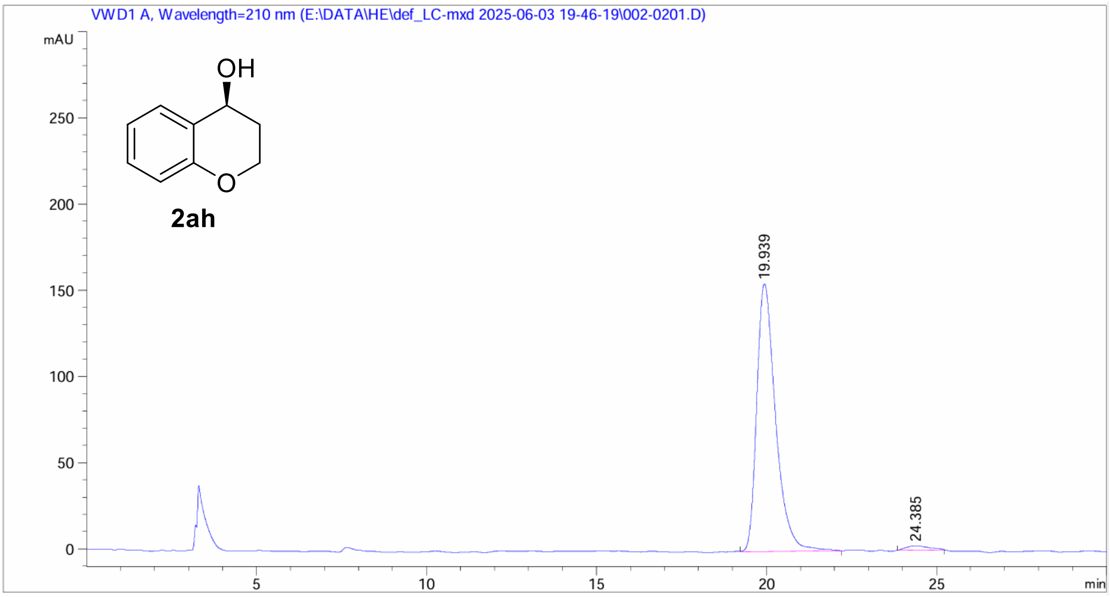

峰 #	保留时间 [min]	类型	峰宽 [min]	峰面积 [mAU*s]	峰高 [mAU]	峰面积 %
1	19.939	MM R	0.6426	5981.56152	155.15121	98.0281
2	24.385	MM R	0.7723	120.32347	2.59667	1.9719

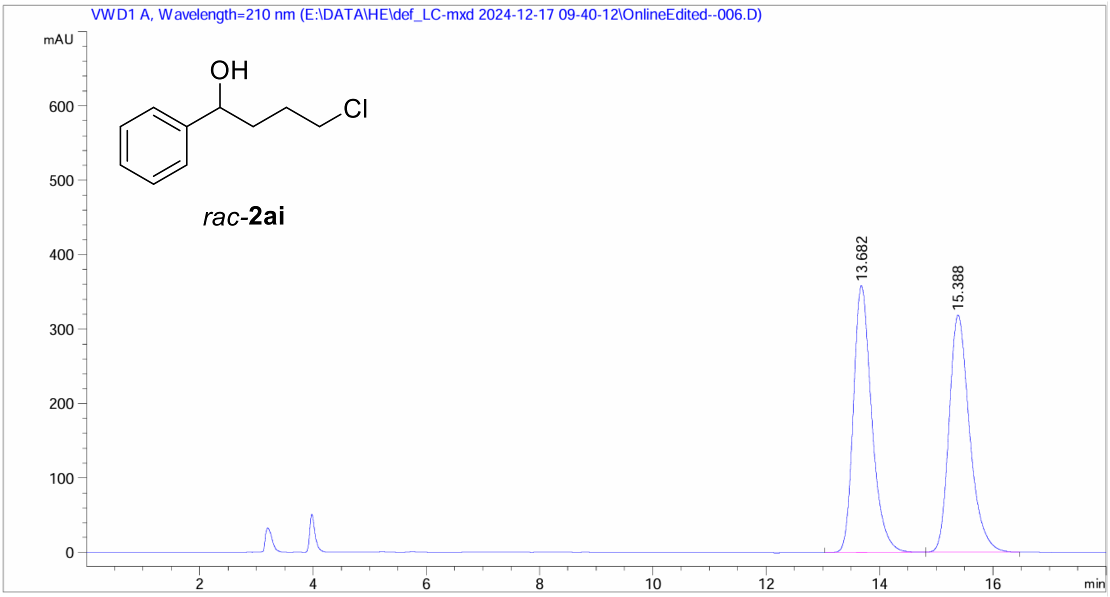

峰 #	保留时间 [min]	类型	峰宽 [min]	峰面积 [mAU*s]	峰高 [mAU]	峰面积 %
1	13.682	BB	0.3421	8056.69971	358.56168	50.1350
2	15.388	BB	0.3835	8013.29736	318.57895	49.8650

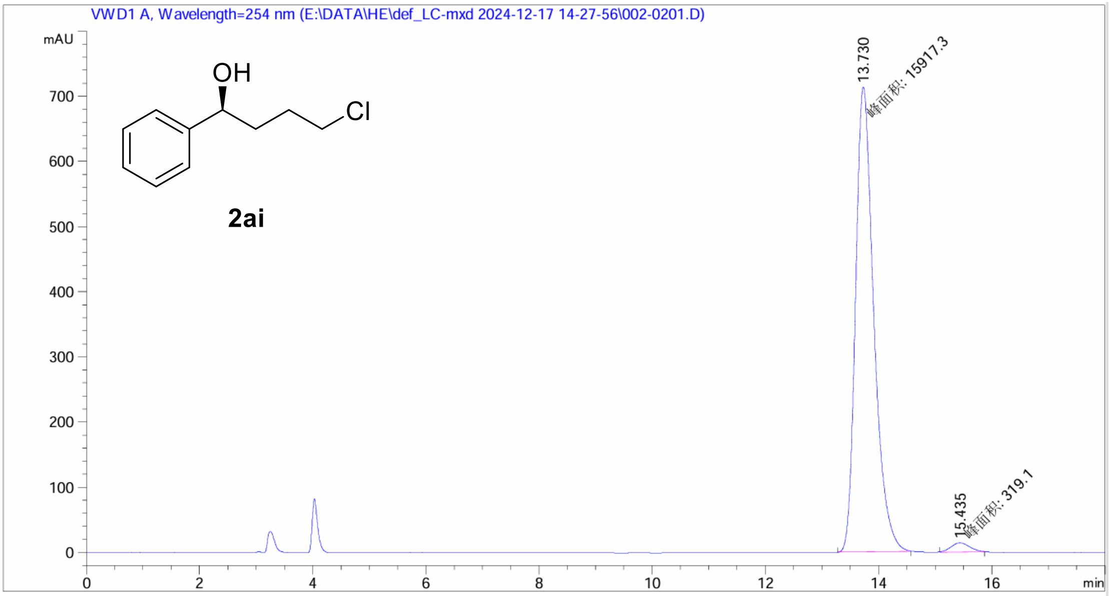

峰 #	保留时间 [min]	类型	峰宽 [min]	峰面积 [mAU*s]	峰高 [mAU]	峰面积 %
1	13.730	MM	0.3719	1.59173e4	713.33435	98.0347
2	15.435	MM	0.3771	319.09982	14.10435	1.9653

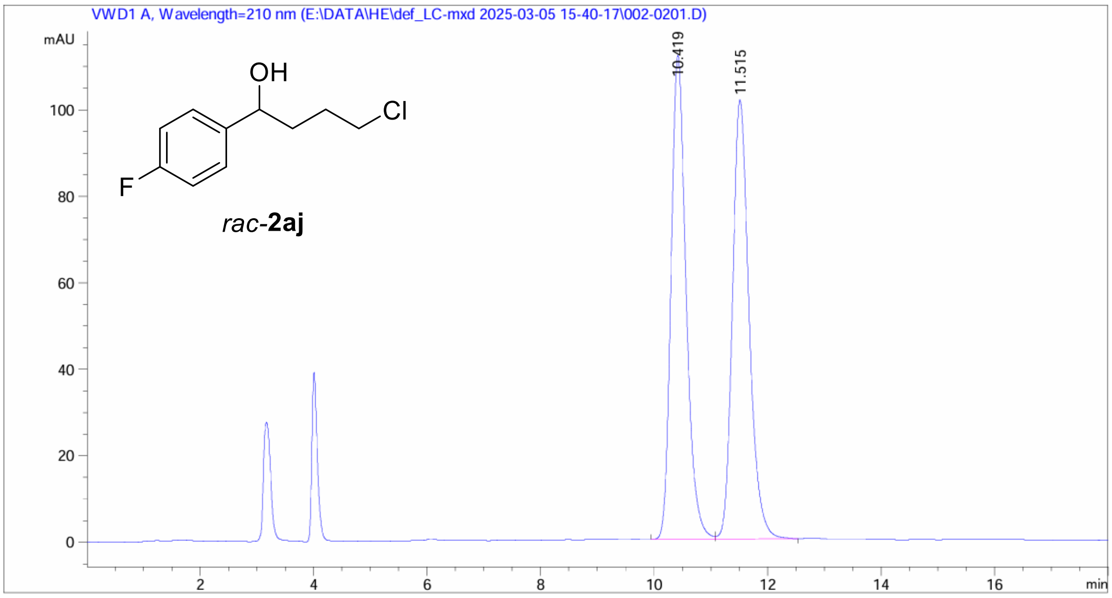

峰 #	保留时间 [min]	类型	峰宽 [min]	峰面积 [mAU*s]	峰高 [mAU]	峰面积 %
1	10.419	BV	0.2805	2055.99170	111.82536	49.8697
2	11.515	VB	0.3103	2066.73438	101.61290	50.1303

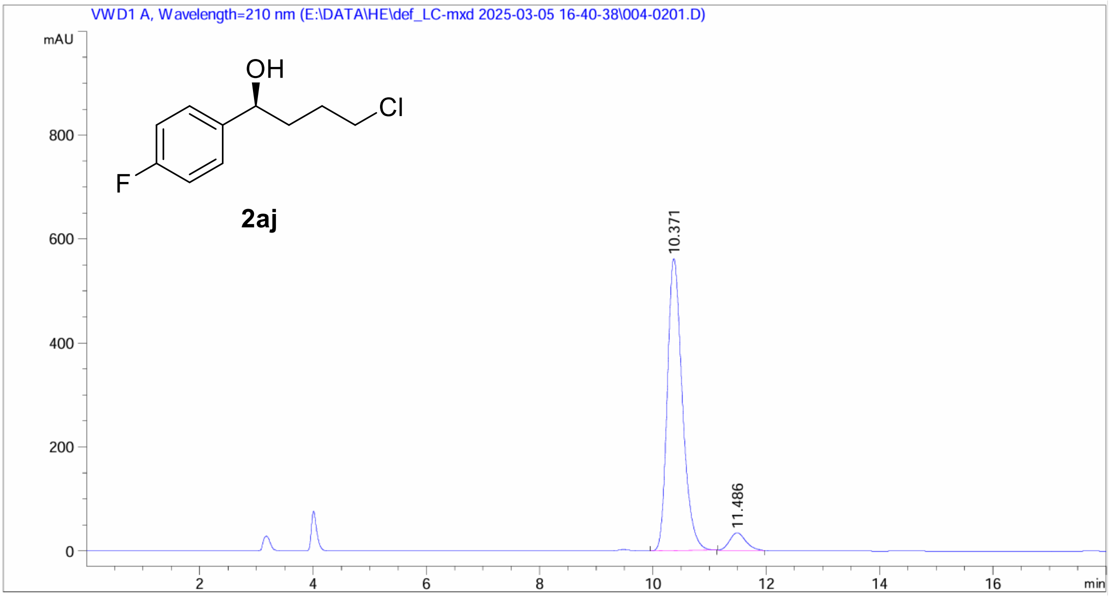

峰 #	保留时间 [min]	类型	峰宽 [min]	峰面积 [mAU*s]	峰高 [mAU]	峰面积 %
1	10.371	MM R	0.3071	1.03612e4	562.35651	93.9808
2	11.486	MM R	0.3282	663.61005	33.69731	6.0192

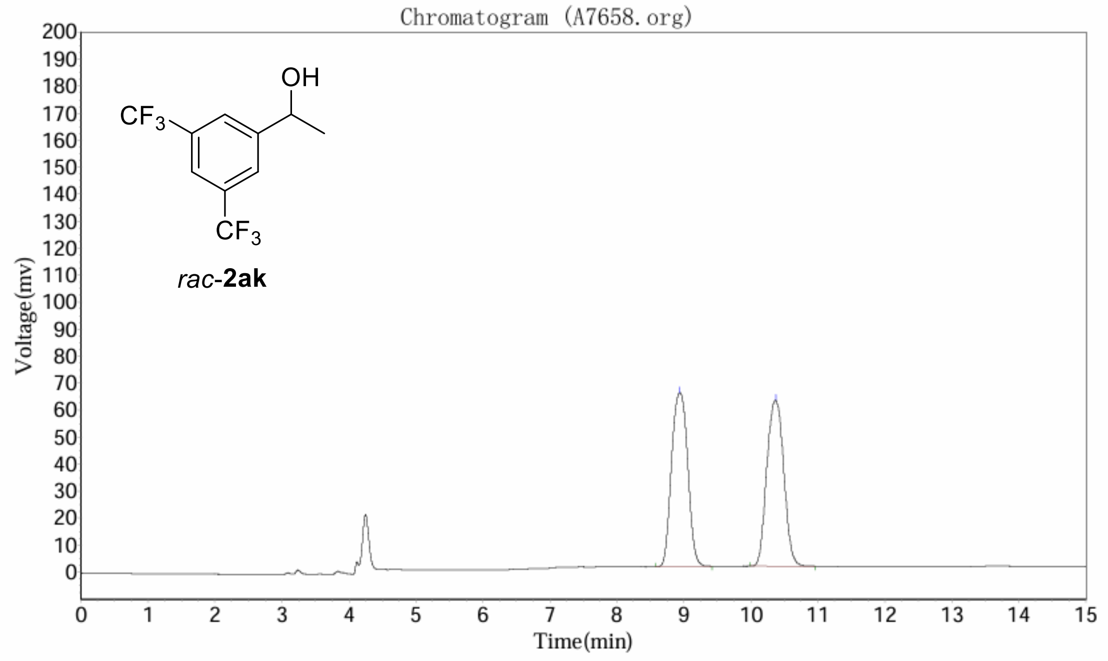

Results

Peak No.	Peak ID	Ret Time	Height	Area	Conc.
1		8.940	64417.801	1079252.500	49.9193
2		10.367	61518.242	1082740.125	50.0807
Total			125936.043	2161992.625	100.0000

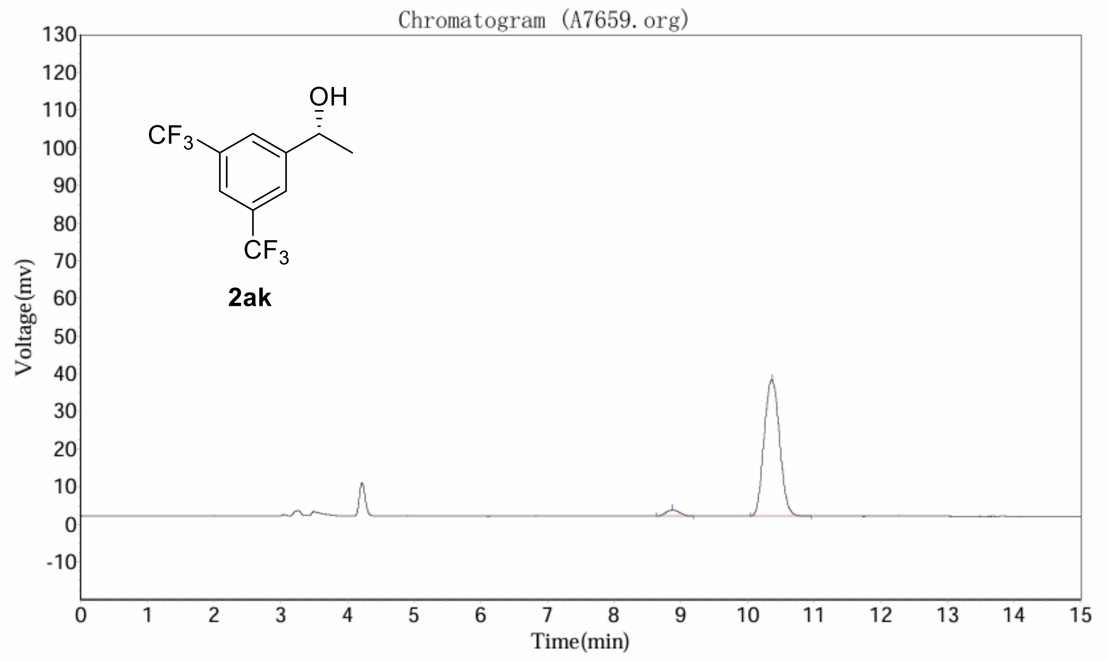

Results

Peak No.	Peak ID	Ret Time	Height	Area	Conc.
1		8.883	1561.287	22947.902	3.7808
2		10.367	36152.281	584007.000	96.2192
Total			37713.568	606954.902	100.0000

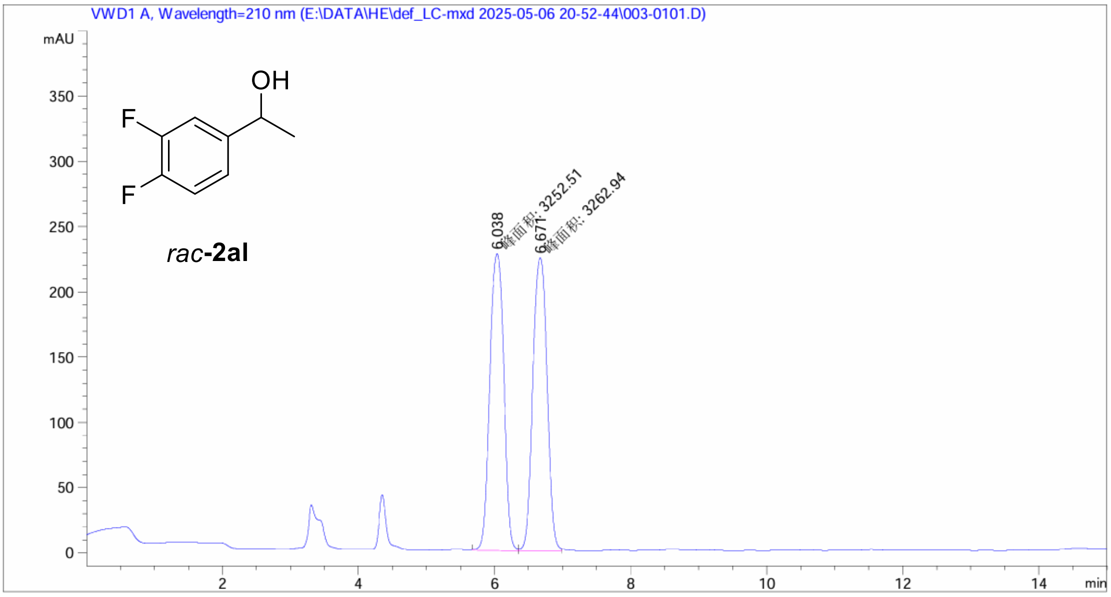

峰 #	保留时间 [min]	类型	峰宽 [min]	峰面积 [mAU*s]	峰高 [mAU]	峰面积 %
1	6.038	FM	0.2386	3252.50977	227.19147	49.9200
2	6.671	MF	0.2423	3262.93506	224.42154	50.0800

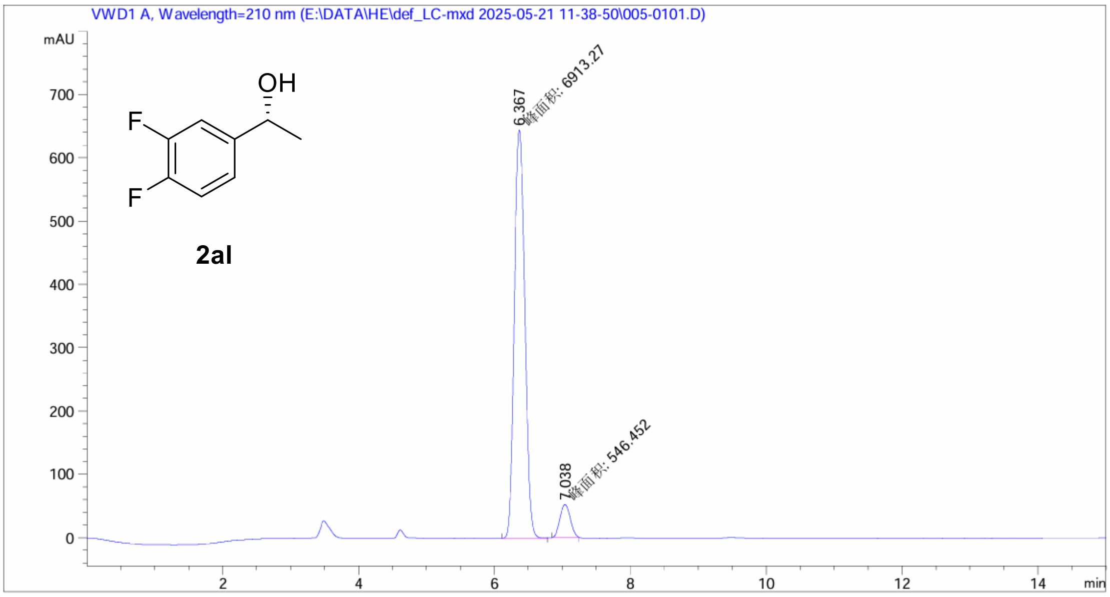

峰 #	保留时间 [min]	类型	峰宽 [min]	峰面积 [mAU*s]	峰高 [mAU]	峰面积 %
1	6.367	FM	0.1786	6913.26563	645.08069	92.6746
2	7.038	MM	0.1761	546.45239	51.72557	7.3254

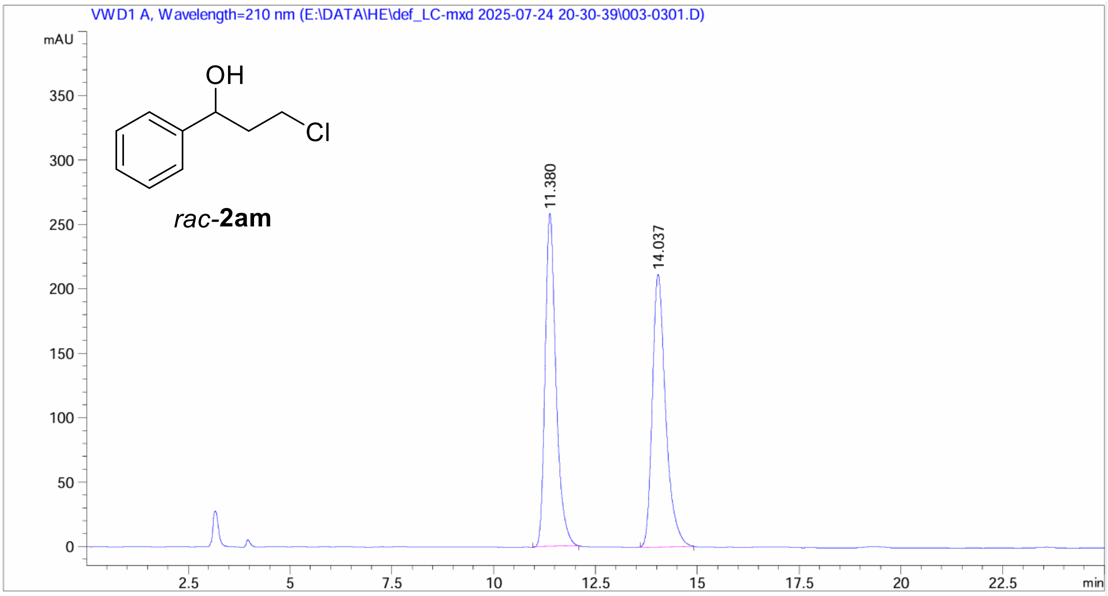

峰 #	保留时间 [min]	类型	峰宽 [min]	峰面积 [mAU*s]	峰高 [mAU]	峰面积 %
1	11.380	MM R	0.3086	4787.36670	258.52203	49.7320
2	14.037	MM R	0.3805	4838.95654	211.94691	50.2680

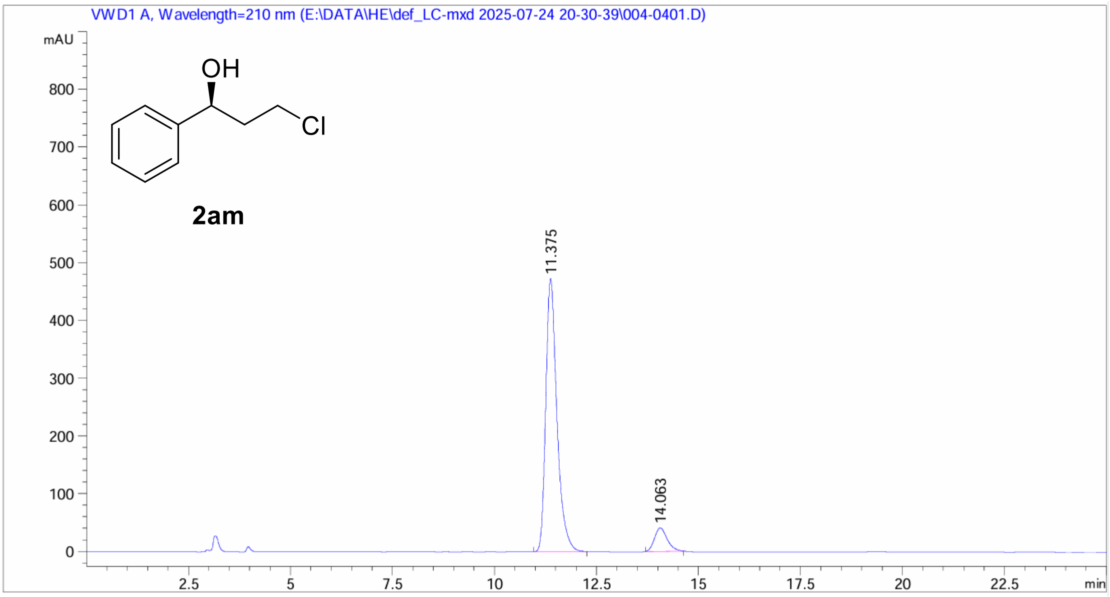

峰 #	保留时间 [min]	类型	峰宽 [min]	峰面积 [mAU*s]	峰高 [mAU]	峰面积 %
1	11.375	MM R	0.3164	8970.18555	472.57663	91.1200
2	14.063	MM R	0.3588	874.17438	40.60527	8.8800

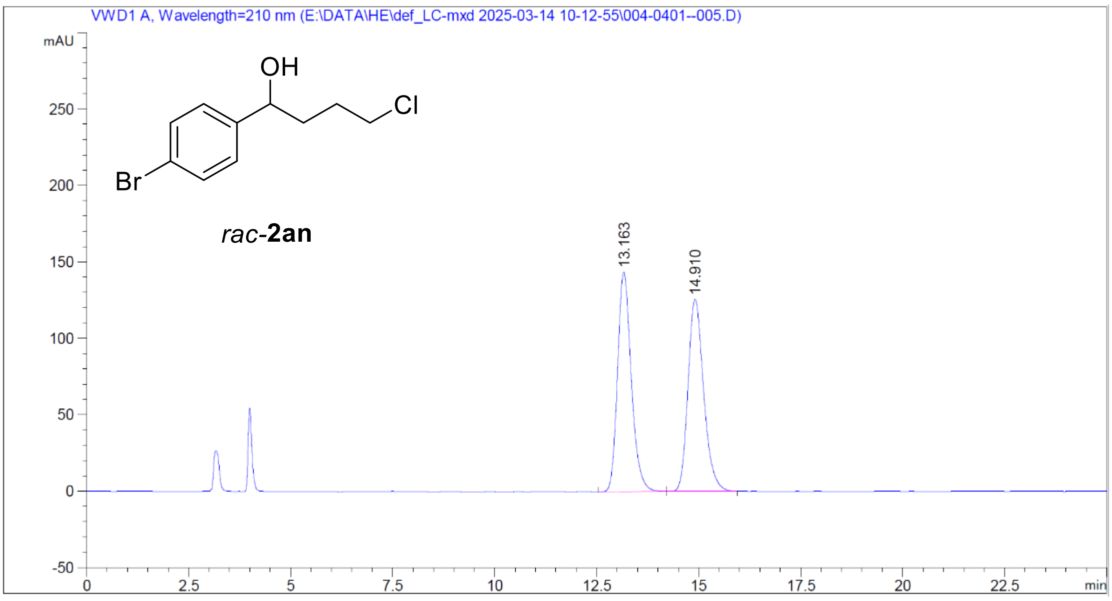

峰 #	保留时间 [min]	类型	峰宽 [min]	峰面积 [mAU*s]	峰高 [mAU]	峰面积 %
1	13.163	BB	0.3591	3366.57544	143.83479	50.1028
2	14.910	BB	0.4090	3352.76489	125.77606	49.8972

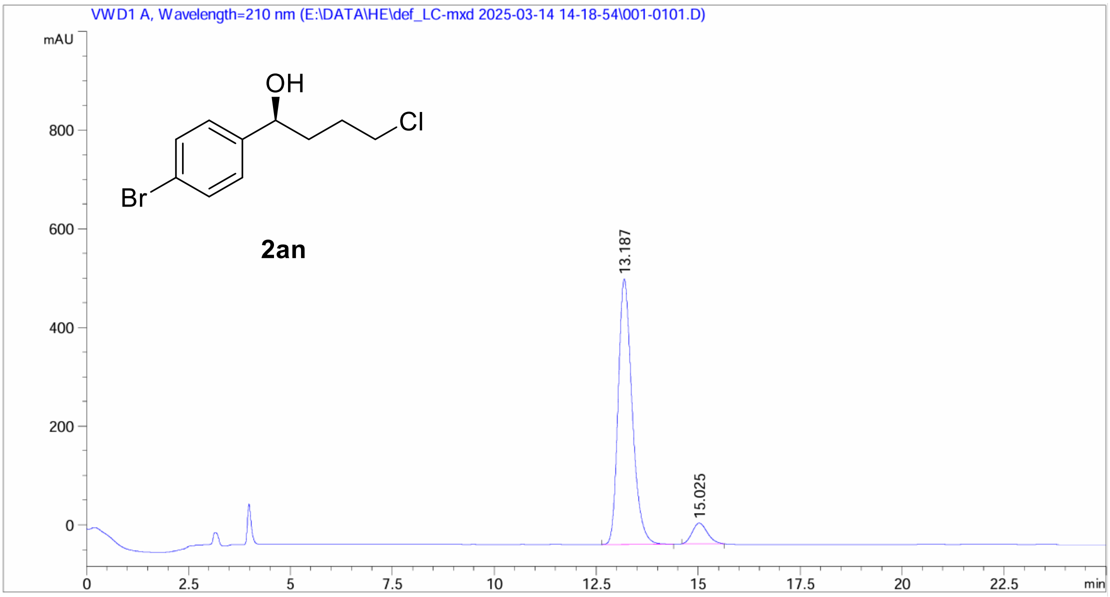

峰 #	保留时间 [min]	类型	峰宽 [min]	峰面积 [mAU*s]	峰高 [mAU]	峰面积 %
1	13.187	MM R	0.3964	1.27957e4	537.95526	92.2278
2	15.025	MM R	0.4271	1078.31958	42.08335	7.7722

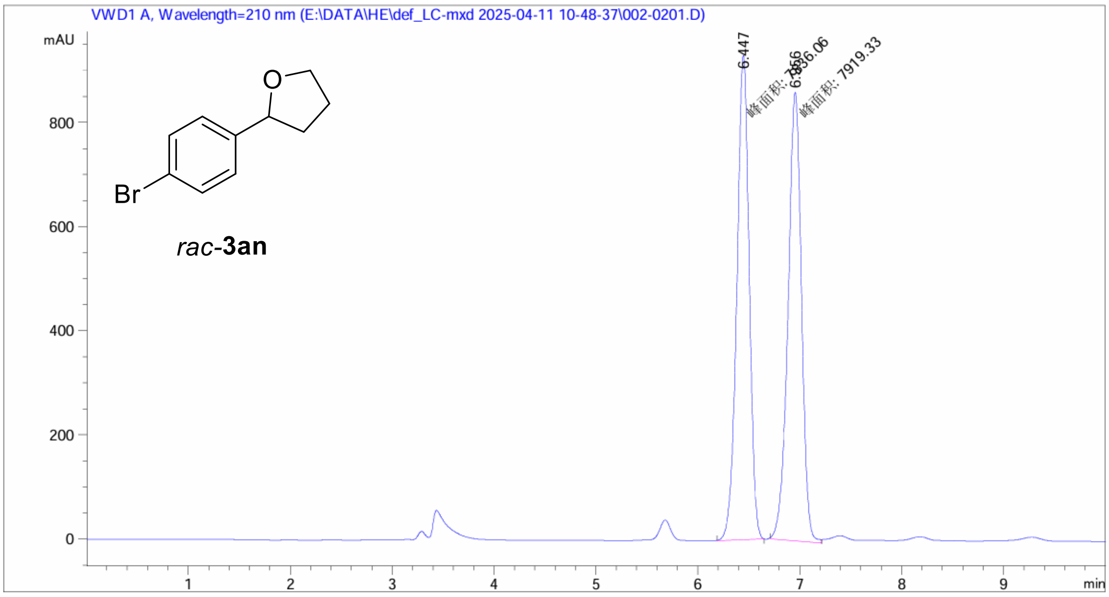

峰 #	保留时间 [min]	类型	峰宽 [min]	峰面积 [mAU*s]	峰高 [mAU]	峰面积 %
1	6.447	MM	0.1404	7836.05811	930.01404	49.7357
2	6.956	MM	0.1532	7919.33496	861.41425	50.2643

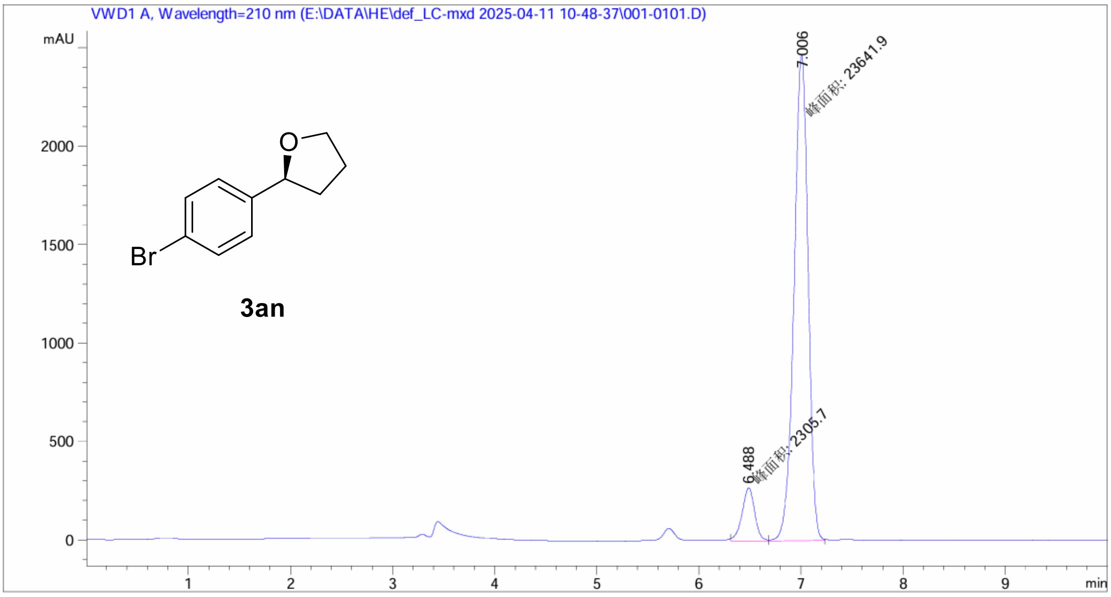

峰 #	保留时间 [min]	类型	峰宽 [min]	峰面积 [mAU*s]	峰高 [mAU]	峰面积 %
1	6.488	FM	0.1429	2305.69800	268.82776	8.8860
2	7.006	MF	0.1597	2.36419e4	2466.73975	91.1140
