# Supplementary material for: Artificial Chiral Trinuclear Zn Catalysts: Design, Self-Assembly and Unprecedented Efficiency in Asymmetric Hydroboration of Ketones
Source: ACS Cent Sci. 2025 Aug 12;11(9):1773–83. doi: 10.1021/acscentsci.5c01067 (PMC12464761; doi:10.1021/acscentsci.5c01067)

checkCIF/PLATON report

Structure factors have been supplied for datablock(s) mj23715_0m

THIS REPORT IS FOR GUIDANCE ONLY. IF USED AS PART OF A REVIEW PROCEDURE FOR PUBLICATION, IT SHOULD NOT REPLACE THE EXPERTISE OF AN EXPERIENCED CRYSTALLOGRAPHIC REFEREE.

No syntax errors found. CIF dictionary Interpreting this report

Datablock: mj23715_0m

Bond precision: C-C = 0.0075 A Wavelength=1.34139

Cell:	a=23.1920(6)	b=23.5097(8)	c=25.2306(7)
	alpha=90	beta=90	gamma=90
Temperature:	173 K		

	Calculated	Reported
Volume	13756.7(7)	13756.7(7)
Space group	P 21 21 21	P 21 21 21
Hall group	P 2ac 2ab	P 2ac 2ab
Moiety formula	C44 H42 N4 O4 [+ solvent]	C44 H42 N4 O4
Sum formula	C44 H42 N4 O4 [+ solvent]	C44 H42 N4 O4
Mr	690.82	690.81
Dx,g cm-3	1.001	1.001
Z	12	12
Mu (mm-1)	0.327	0.327
F000	4392.0	4392.0
F000’	4401.53	
h,k,lmax		28,28,30
Nref		26194
Tmin,Tmax	0.946,0.984	0.616,0.751
Tmin’	0.946	

Correction method= # Reported T Limits: Tmin=0.616 Tmax=0.751
AbsCorr = MULTI-SCAN

Data completeness= Theta(max)= 55.030

R(reflections)= 0.0677(20633) wR2(reflections)= 0.2037(26194)

S = 1.019 Npar= 1429

The following ALERTS were generated. Each ALERT has the format
test-name_ALERT_alert-type_alert-level.
Click on the hyperlinks for more details of the test.

Alert level C

```
DIFMX02_ALERT_1_C  The maximum difference density is > 0.1*ZMAX*0.75
            The relevant atom site should be identified.
PLAT094_ALERT_2_C Ratio of Maximum / Minimum Residual Density ....       2.02 Report
PLAT097_ALERT_2_C Large Reported Max.  (Positive) Residual Density       0.67 eA-3
PLAT220_ALERT_2_C NonSolvent   Resd 2  C   Ueq(max)/Ueq(min) Range        3.3 Ratio
PLAT220_ALERT_2_C NonSolvent   Resd 3  C   Ueq(max)/Ueq(min) Range        3.3 Ratio
PLAT230_ALERT_2_C Hirshfeld Test Diff for    N2        --C38      .       6.0 s.u.
PLAT230_ALERT_2_C Hirshfeld Test Diff for    C40       --C41      .       5.7 s.u.
PLAT234_ALERT_4_C Large Hirshfeld Difference C41       --C44      .      0.17 Ang.
PLAT234_ALERT_4_C Large Hirshfeld Difference C41A      --C43A     .      0.16 Ang.
PLAT241_ALERT_2_C High   'MainMol' Ueq as Compared to Neighbors of       C39B Check
PLAT242_ALERT_2_C Low    'MainMol' Ueq as Compared to Neighbors of       C29A Check
PLAT242_ALERT_2_C Low    'MainMol' Ueq as Compared to Neighbors of       C29B Check
PLAT242_ALERT_2_C Low    'MainMol' Ueq as Compared to Neighbors of       C41B Check
PLAT340_ALERT_3_C Low Bond Precision on  C-C Bonds ..............    0.00745 Ang.
PLAT413_ALERT_2_C Short Inter XH3 .. XHn     H44C      ..H44F     .      2.13 Ang.
                                           1-x,1/2+y,3/2-z  =      3_656 Check
PLAT911_ALERT_3_C Missing FCF Refl Between Thmin & STh/L=    0.600          7 Report
                7  2  0,   1  4  0,   5  0  1,   1  0  2,   0  2  2,   4  0  3,
                0  2  4,
PLAT918_ALERT_3_C Reflection(s) with I(obs) much Smaller I(calc) .          7 Check
```

Alert level G

```
ABSMU01_ALERT_1_G  Calculation of _exptl_absorpt_correction_mu
                not performed for this radiation type.
PLAT007_ALERT_5_G Number of Unrefined Donor-H Atoms .............           6 Report
              H1    H2    H1A   H2A   H1B   H2B
PLAT072_ALERT_2_G SHELXL First  Parameter in WGHT  Unusually Large       0.15 Report
PLAT398_ALERT_2_G Deviating  C-O-C   Angle From 120 for O3        .     104.6 Degree
PLAT398_ALERT_2_G Deviating  C-O-C   Angle From 120 for O4        .     104.8 Degree
PLAT398_ALERT_2_G Deviating  C-O-C   Angle From 120 for O3A       .     105.4 Degree
PLAT398_ALERT_2_G Deviating  C-O-C   Angle From 120 for O4A       .     105.3 Degree
PLAT398_ALERT_2_G Deviating  C-O-C   Angle From 120 for O3B       .     105.4 Degree
PLAT398_ALERT_2_G Deviating  C-O-C   Angle From 120 for O4B       .     107.0 Degree
PLAT606_ALERT_4_G Solvent Accessible VOID(S) in Structure ........          ! Info
PLAT868_ALERT_4_G ALERTS Due to the Use of _smtbx_masks Suppressed          ! Info
PLAT910_ALERT_3_G Missing # of FCF Reflection(s) Below Theta(Min).          3 Note
                1  1  0,   1  0  1,   0  1  1,
PLAT912_ALERT_4_G Missing # of FCF Reflections Above STh/L=  0.600         37 Note
PLAT913_ALERT_3_G Missing # of Very Strong Reflections in FCF ....          3 Note
                1  1  0,   1  0  1,   0  1  1,
PLAT933_ALERT_2_G Number of HKL-OMIT Records in Embedded .res File          6 Note
                0  2  2,   0  2  4,   1  0  2,   1  4  0,   4  0  3,   7  2  0,
PLAT978_ALERT_2_G Number C-C Bonds with Positive Residual Density.          0 Info
```

0 **ALERT level A** = Most likely a serious problem - resolve or explain
0 **ALERT level B** = A potentially serious problem, consider carefully
17 **ALERT level C** = Check. Ensure it is not caused by an omission or oversight

```
16 ALERT level G = General information/check it is not something unexpected

 2 ALERT type 1 CIF construction/syntax error, inconsistent or missing data
20 ALERT type 2 Indicator that the structure model may be wrong or deficient
 5 ALERT type 3 Indicator that the structure quality may be low
 5 ALERT type 4 Improvement, methodology, query or suggestion
 1 ALERT type 5 Informative message, check
```

It is advisable to attempt to resolve as many as possible of the alerts in all categories. Often the minor alerts point to easily fixed oversights, errors and omissions in your CIF or refinement strategy, so attention to these fine details can be worthwhile. In order to resolve some of the more serious problems it may be necessary to carry out additional measurements or structure refinements. However, the purpose of your study may justify the reported deviations and the more serious of these should normally be commented upon in the discussion or experimental section of a paper or in the "special_details" fields of the CIF. checkCIF was carefully designed to identify outliers and unusual parameters, but every test has its limitations and alerts that are not important in a particular case may appear. Conversely, the absence of alerts does not guarantee there are no aspects of the results needing attention. It is up to the individual to critically assess their own results and, if necessary, seek expert advice.

Publication of your CIF in IUCr journals

A basic structural check has been run on your CIF. These basic checks will be run on all CIFs submitted for publication in IUCr journals (*Acta Crystallographica*, *Journal of Applied Crystallography*, *Journal of Synchrotron Radiation*); however, if you intend to submit to *Acta Crystallographica Section C* or *E* or *IUCrData*, you should make sure that full publication checks are run on the final version of your CIF prior to submission.

Publication of your CIF in other journals

Please refer to the *Notes for Authors* of the relevant journal for any special instructions relating to CIF submission.

PLATON version of 29/11/2023; check.def file version of 14/09/2023

Datablock mj23715_0m - ellipsoid plot

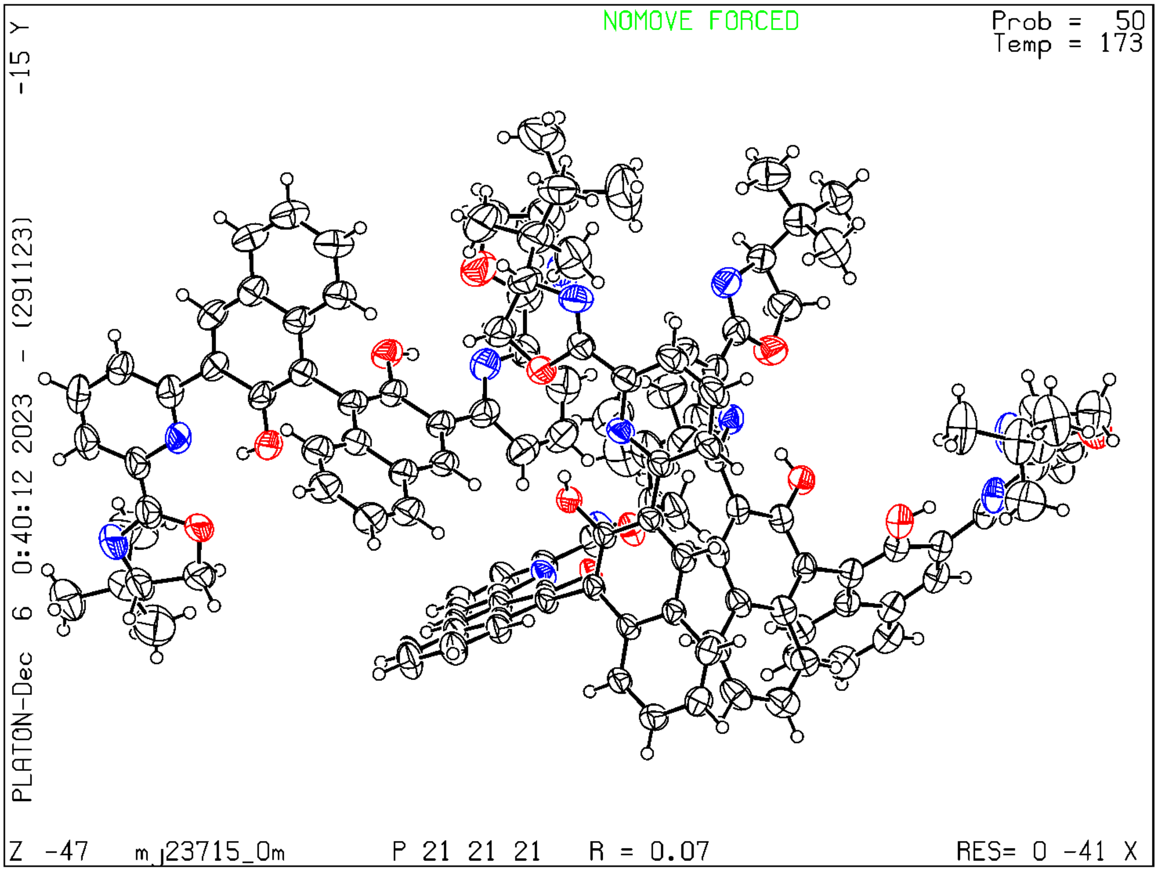

Supplement: Supplementary file 2 [file oc5c01067_si_002.pdf]
