# Supplementary material for: Artificial Chiral Trinuclear Zn Catalysts: Design, Self-Assembly and Unprecedented Efficiency in Asymmetric Hydroboration of Ketones
Source: ACS Cent Sci. 2025 Aug 12;11(9):1773–83. doi: 10.1021/acscentsci.5c01067 (PMC12464761; doi:10.1021/acscentsci.5c01067)

checkCIF/PLATON report

Structure factors have been supplied for datablock(s) exp_4540_auto

THIS REPORT IS FOR GUIDANCE ONLY. IF USED AS PART OF A REVIEW PROCEDURE FOR PUBLICATION, IT SHOULD NOT REPLACE THE EXPERTISE OF AN EXPERIENCED CRYSTALLOGRAPHIC REFEREE.

No syntax errors found. CIF dictionary Interpreting this report

Datablock: exp_4540_auto

Bond precision:	C-C = 0.0296 A		Wavelength=1.54184
Cell:	a=20.0152(5)	b=20.0152(5)	c=37.6884(14)
	alpha=90	beta=90	gamma=90
Temperature:	173 K		

	Calculated	Reported
Volume	15098.3(9)	15098.3(9)
Space group	P 41	P 41
Hall group	P 4w	P 4w
Moiety formula	C52 H51 N4 O12 Zn3, C52 H52 N4 O12 Zn3, C4 H8 O2, C2 H3 N	C52 H52 N4 O12 Zn3, C52 H51 N4 O12 Zn3, C2 H3 N, C4 H8 O2
Sum formula	C110 H114 N9 O26 Zn6	C110 H114 N9 O26 Zn6
Mr	2370.45	2370.32
Dx,g cm-3	1.043	1.043
Z	4	4
Mu (mm-1)	1.514	1.514
F000	4900.0	4900.0
F000'	4876.10	
h,k,lmax	23,23,45	23,23,45
Nref	26939[13693]	26916
Tmin,Tmax	0.698,0.847	0.728,1.000
Tmin'	0.633	

Correction method= # Reported T Limits: Tmin=0.728 Tmax=1.000
AbsCorr = MULTI-SCAN

Data completeness= 1.97/1.00 Theta(max)= 67.045

R(reflections)= 0.1109(17076)   wR2(reflections)= 0.3206(26916)

S = 1.050   Npar= 1381

The following ALERTS were generated. Each ALERT has the format
test-name_ALERT_alert-type_alert-level.
Click on the hyperlinks for more details of the test.

Alert level A

PLAT602_ALERT_2_A Solvent Accessible VOID(S) in Structure .... ! Check

---

Alert level B

PLAT234_ALERT_4_B Large Hirshfeld Difference C14 --C15 . 0.28 Ang.
PLAT234_ALERT_4_B Large Hirshfeld Difference C27 --C28 . 0.30 Ang.
PLAT234_ALERT_4_B Large Hirshfeld Difference C31 --C32 . 0.26 Ang.
PLAT234_ALERT_4_B Large Hirshfeld Difference O21 --C95 . 0.28 Ang.
PLAT242_ALERT_2_B Low 'MainMol' Ueq as Compared to Neighbors of C47 Check
PLAT341_ALERT_3_B Low Bond Precision on C-C Bonds .... 0.0296 Ang.

Alert level C

RINTA01_ALERT_3_C The value of Rint is greater than 0.12
Rint given 0.150
PLAT020_ALERT_3_C The Value of Rint is Greater Than 0.12 .... 0.150 Report
PLAT042_ALERT_1_C Calc. and Reported MoietyFormula Strings Differ Please Check
Calc: C52 H51 N4 O12 Zn3, C52 H52 N4 O12 Zn3, C4 H8 O2, C2 H3 N
Rep.: C52 H52 N4 O12 Zn3, C52 H51 N4 O12 Zn3, C2 H3 N, C4 H8 O2
PLAT082_ALERT_2_C High R1 Value .... 0.11 Report
PLAT084_ALERT_3_C High wR2 Value (i.e. > 0.25) .... 0.32 Report
PLAT094_ALERT_2_C Ratio of Maximum / Minimum Residual Density .... 2.86 Report
PLAT220_ALERT_2_C NonSolvent Resd 1 C Ueq(max)/Ueq(min) Range 4.1 Ratio
PLAT220_ALERT_2_C NonSolvent Resd 1 O Ueq(max)/Ueq(min) Range 3.5 Ratio
PLAT222_ALERT_3_C NonSolvent Resd 1 H Uiso(max)/Uiso(min) Range 4.5 Ratio
PLAT230_ALERT_2_C Hirshfeld Test Diff for C61 --C62 . 5.1 s.u.
PLAT234_ALERT_4_C Large Hirshfeld Difference O2 --C49 . 0.18 Ang.
PLAT234_ALERT_4_C Large Hirshfeld Difference O3 --C49 . 0.22 Ang.
PLAT234_ALERT_4_C Large Hirshfeld Difference O5 --C51 . 0.23 Ang.
PLAT234_ALERT_4_C Large Hirshfeld Difference O6 --C51 . 0.21 Ang.
PLAT234_ALERT_4_C Large Hirshfeld Difference N1 --C3 . 0.21 Ang.
PLAT234_ALERT_4_C Large Hirshfeld Difference N2 --C4 . 0.17 Ang.
PLAT234_ALERT_4_C Large Hirshfeld Difference C3 --C4 . 0.20 Ang.
PLAT234_ALERT_4_C Large Hirshfeld Difference C19 --C20 . 0.19 Ang.
PLAT234_ALERT_4_C Large Hirshfeld Difference C19 --C28 . 0.25 Ang.
PLAT234_ALERT_4_C Large Hirshfeld Difference C20 --C21 . 0.18 Ang.
PLAT234_ALERT_4_C Large Hirshfeld Difference C22 --C23 . 0.20 Ang.
PLAT234_ALERT_4_C Large Hirshfeld Difference O16 --C97 . 0.17 Ang.
PLAT234_ALERT_4_C Large Hirshfeld Difference O17 --C62 . 0.17 Ang.
PLAT234_ALERT_4_C Large Hirshfeld Difference N8 --C86 . 0.17 Ang.
PLAT234_ALERT_4_C Large Hirshfeld Difference C62 --C63 . 0.23 Ang.
PLAT234_ALERT_4_C Large Hirshfeld Difference C64 --C65 . 0.20 Ang.
PLAT234_ALERT_4_C Large Hirshfeld Difference C69 --C70 . 0.22 Ang.
PLAT234_ALERT_4_C Large Hirshfeld Difference C71 --C72 . 0.17 Ang.

```
PLAT241_ALERT_2_C High   ’MainMol’ Ueq as Compared to Neighbors of         O1 Check
PLAT241_ALERT_2_C High   ’MainMol’ Ueq as Compared to Neighbors of        C15 Check
PLAT241_ALERT_2_C High   ’MainMol’ Ueq as Compared to Neighbors of        C19 Check
PLAT241_ALERT_2_C High   ’MainMol’ Ueq as Compared to Neighbors of        C25 Check
PLAT241_ALERT_2_C High   ’MainMol’ Ueq as Compared to Neighbors of        C27 Check
PLAT241_ALERT_2_C High   ’MainMol’ Ueq as Compared to Neighbors of        C31 Check
PLAT241_ALERT_2_C High   ’MainMol’ Ueq as Compared to Neighbors of        C36 Check
PLAT241_ALERT_2_C High   ’MainMol’ Ueq as Compared to Neighbors of        C41 Check
PLAT241_ALERT_2_C High   ’MainMol’ Ueq as Compared to Neighbors of        O16 Check
PLAT241_ALERT_2_C High   ’MainMol’ Ueq as Compared to Neighbors of        O19 Check
PLAT241_ALERT_2_C High   ’MainMol’ Ueq as Compared to Neighbors of        O22 Check
PLAT241_ALERT_2_C High   ’MainMol’ Ueq as Compared to Neighbors of        C54 Check
PLAT241_ALERT_2_C High   ’MainMol’ Ueq as Compared to Neighbors of        C70 Check
PLAT242_ALERT_2_C Low    ’MainMol’ Ueq as Compared to Neighbors of         N3 Check
PLAT242_ALERT_2_C Low    ’MainMol’ Ueq as Compared to Neighbors of        C28 Check
PLAT242_ALERT_2_C Low    ’MainMol’ Ueq as Compared to Neighbors of        C35 Check
PLAT242_ALERT_2_C Low    ’MainMol’ Ueq as Compared to Neighbors of        C37 Check
PLAT242_ALERT_2_C Low    ’MainMol’ Ueq as Compared to Neighbors of        C89 Check
PLAT242_ALERT_2_C Low    ’MainMol’ Ueq as Compared to Neighbors of        C93 Check
PLAT242_ALERT_2_C Low    ’MainMol’ Ueq as Compared to Neighbors of        C97 Check
PLAT242_ALERT_2_C Low    ’MainMol’ Ueq as Compared to Neighbors of        C99 Check
PLAT242_ALERT_2_C Low    ’MainMol’ Ueq as Compared to Neighbors of       C101 Check
PLAT244_ALERT_4_C Low    ’Solvent’ Ueq as Compared to Neighbors of       C202 Check
PLAT260_ALERT_2_C Large Average Ueq of Residue Including      O25      0.163 Check
PLAT260_ALERT_2_C Large Average Ueq of Residue Including       N9      0.179 Check
PLAT361_ALERT_2_C Long   C(sp3)-C(sp3) Bond  C36      - C41      .      1.65 Ang.
PLAT369_ALERT_2_C Long   C(sp2)-C(sp2) Bond  C60      - C63      .      1.53 Ang.
PLAT906_ALERT_3_C Large K Value in the Analysis of Variance ......     5.597 Check
PLAT906_ALERT_3_C Large K Value in the Analysis of Variance ......     2.174 Check
PLAT911_ALERT_3_C Missing FCF Refl Between Thmin & STh/L=    0.597         9 Report
                3  4  0,   1  1  1,   0  3  1,   0  4  1,   1  1  2,   1  2  2,
                1  3  2,   1  2  5,  -1  3  5,
```

Alert level G

```
PLAT002_ALERT_2_G Number of Distance or Angle Restraints on AtSite        10 Note
PLAT003_ALERT_2_G Number of Uiso or U(i,j) Restrained non-H Atoms          6 Report
PLAT072_ALERT_2_G SHELXL First  Parameter in WGHT  Unusually Large      0.20 Report
PLAT172_ALERT_4_G The CIF-Embedded .res File Contains DFIX Records         3 Report
PLAT177_ALERT_4_G The CIF-Embedded .res File Contains DELU Records         1 Report
PLAT178_ALERT_4_G The CIF-Embedded .res File Contains SIMU Records         1 Report
PLAT188_ALERT_3_G A Non-default SIMU Restraint Value has been used    0.0100 Report
PLAT192_ALERT_3_G A Non-default DELU Restraint Value for SecondPar    0.0200 Report
PLAT333_ALERT_2_G Large Aver C6-Ring C-C Dist C19      -C24      .      1.42 Ang.
PLAT335_ALERT_2_G Check Large C6 Ring C-C Range C12      -C17           0.18 Ang.
PLAT335_ALERT_2_G Check Large C6 Ring C-C Range C65      -C70           0.27 Ang.
PLAT343_ALERT_2_G Unusual sp?      Angle Range in Main Residue for       C31 Check
PLAT398_ALERT_2_G Deviating  C-O-C   Angle From 120 for O1       .      106.9 Degree
PLAT398_ALERT_2_G Deviating  C-O-C   Angle From 120 for O12      .      106.5 Degree
PLAT398_ALERT_2_G Deviating  C-O-C   Angle From 120 for O13      .      104.2 Degree
PLAT398_ALERT_2_G Deviating  C-O-C   Angle From 120 for O24      .      104.5 Degree
PLAT721_ALERT_1_G Bond    Calc     0.99000, Rep     0.97990 Dev...      0.01 Ang.
              C44      -H44A           1_555   1_555 ........     #  272 Check
PLAT721_ALERT_1_G Bond    Calc     0.97000, Rep     0.98010 Dev...      0.01 Ang.
              C44      -H44C           1_555   1_555 ........     #  274 Check
PLAT790_ALERT_4_G Centre of Gravity not Within Unit Cell: Resd.  #         4 Note
              C2 H3 N
```

```
PLAT791_ALERT_4_G Model has Chirality at C1         (Sohncke SpGr)           S Verify
PLAT791_ALERT_4_G Model has Chirality at C41        (Sohncke SpGr)           S Verify
PLAT791_ALERT_4_G Model has Chirality at C53        (Sohncke SpGr)           S Verify
PLAT791_ALERT_4_G Model has Chirality at C87        (Sohncke SpGr)           S Verify
PLAT794_ALERT_5_G Tentative Bond Valency for Zn1       (II)      .       2.04 Info
PLAT794_ALERT_5_G Tentative Bond Valency for Zn2       (II)      .       2.13 Info
PLAT794_ALERT_5_G Tentative Bond Valency for Zn3       (II)      .       1.93 Info
PLAT794_ALERT_5_G Tentative Bond Valency for Zn4       (II)      .       1.97 Info
PLAT794_ALERT_5_G Tentative Bond Valency for Zn5       (II)      .       2.09 Info
PLAT794_ALERT_5_G Tentative Bond Valency for Zn6       (II)      .       2.00 Info
PLAT860_ALERT_3_G Number of Least-Squares Restraints ............          49 Note
PLAT910_ALERT_3_G Missing # of FCF Reflection(s) Below Theta(Min).          2 Note
                0  1  0,   0  1  1,
PLAT913_ALERT_3_G Missing # of Very Strong Reflections in FCF ....          1 Note
                0  1  0,
PLAT933_ALERT_2_G Number of HKL-OMIT Records in Embedded .res File         16 Note
               -1  3 -5,  -1  3  5,   0  3 -1,   0  3  1,   0  4 -1,   0  4  1,
                1  1 -2,   1  1  1,   1  1  2,   1  2 -5,   1  2 -2,   1  2  2,
                1  2  5,   1  3 -2,   1  3  2,   3  4  0,
PLAT955_ALERT_1_G Reported (CIF) and Actual (FCF) Lmax Differ by .          1 Units
PLAT969_ALERT_5_G The 'Henn et al.' R-Factor-gap value ..........       3.225 Note
              Predicted wR2: Based on SigI**2  9.94 or SHELX Weight 30.53
PLAT978_ALERT_2_G Number C-C Bonds with Positive Residual Density.          0 Info
```

```
  1 ALERT level A = Most likely a serious problem - resolve or explain
  6 ALERT level B = A potentially serious problem, consider carefully
 58 ALERT level C = Check. Ensure it is not caused by an omission or oversight
 36 ALERT level G = General information/check it is not something unexpected

  4 ALERT type 1 CIF construction/syntax error, inconsistent or missing data
 46 ALERT type 2 Indicator that the structure model may be wrong or deficient
 13 ALERT type 3 Indicator that the structure quality may be low
 31 ALERT type 4 Improvement, methodology, query or suggestion
  7 ALERT type 5 Informative message, check
```

It is advisable to attempt to resolve as many as possible of the alerts in all categories. Often the minor alerts point to easily fixed oversights, errors and omissions in your CIF or refinement strategy, so attention to these fine details can be worthwhile. In order to resolve some of the more serious problems it may be necessary to carry out additional measurements or structure refinements. However, the purpose of your study may justify the reported deviations and the more serious of these should normally be commented upon in the discussion or experimental section of a paper or in the "special_details" fields of the CIF. checkCIF was carefully designed to identify outliers and unusual parameters, but every test has its limitations and alerts that are not important in a particular case may appear. Conversely, the absence of alerts does not guarantee there are no aspects of the results needing attention. It is up to the individual to critically assess their own results and, if necessary, seek expert advice.

Publication of your CIF in IUCr journals

A basic structural check has been run on your CIF. These basic checks will be run on all CIFs submitted for publication in IUCr journals (*Acta Crystallographica*, *Journal of Applied Crystallography*, *Journal of Synchrotron Radiation*); however, if you intend to submit to *Acta Crystallographica Section C* or *E* or *IUCrData*, you should make sure that full publication checks are run on the final version of your CIF prior to submission.

Publication of your CIF in other journals

Please refer to the *Notes for Authors* of the relevant journal for any special instructions relating to CIF submission.

Validation response form

Please find below a validation response form (VRF) that can be filled in and pasted into your CIF.

```
# start Validation Reply Form
_vrf_PLAT602_exp_4540_auto
;
PROBLEM: Solvent Accessible VOID(S) in Structure ........          ! Check
RESPONSE: ...
;
# end Validation Reply Form
```

PLATON version of 22/08/2024; check.def file version of 21/08/2024

Datablock exp_4540_auto - ellipsoid plot

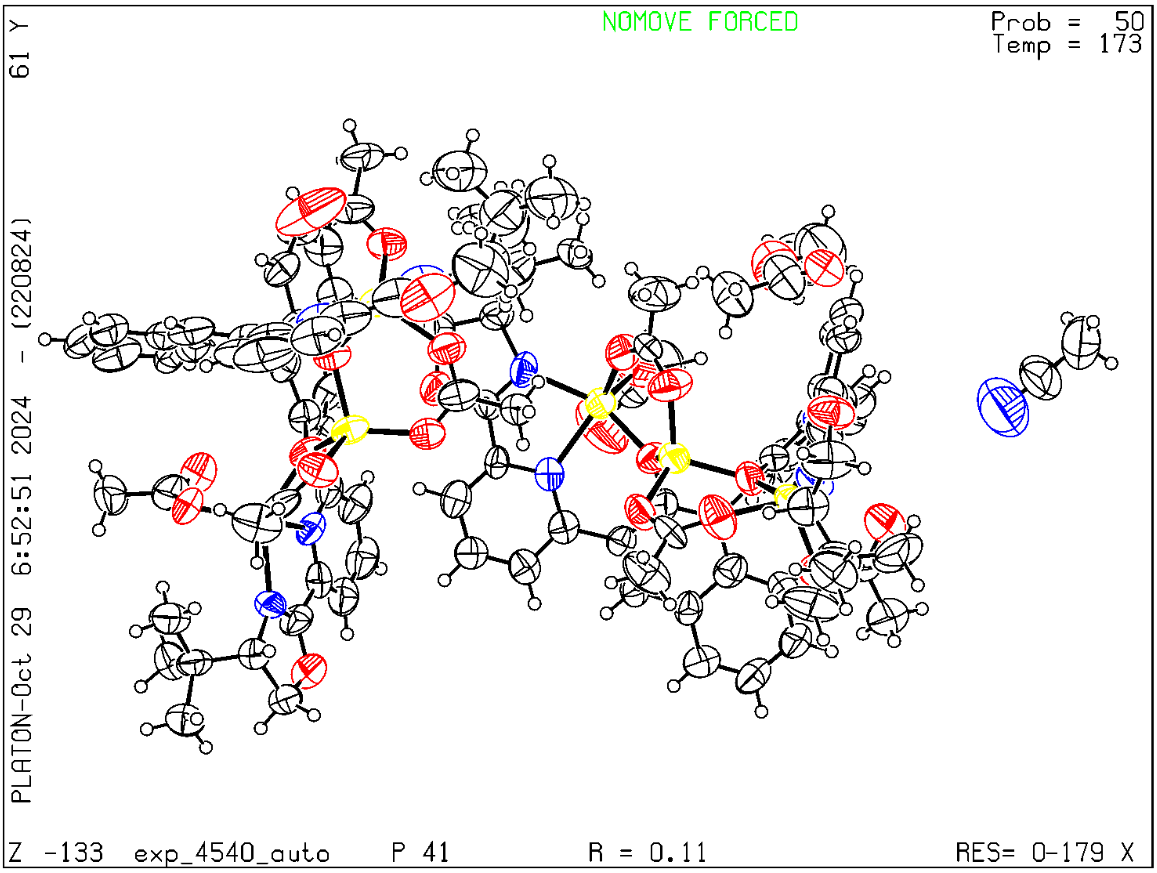

Supplement: Supplementary file 3 [file oc5c01067_si_003.pdf]
